# Supplementary material for: Blocking SHP2 benefits FGFR2 inhibitor and overcomes its resistance in FGFR2-amplified gastric cancer
Source: eLife. 2026 Mar 23;14:RP104060. doi: 10.7554/eLife.104060 (PMC13008354; doi:10.7554/eLife.104060)
Supplement: Figure 2—source data 1. [file elife-104060-fig2-data1.pdf]

Figure 2

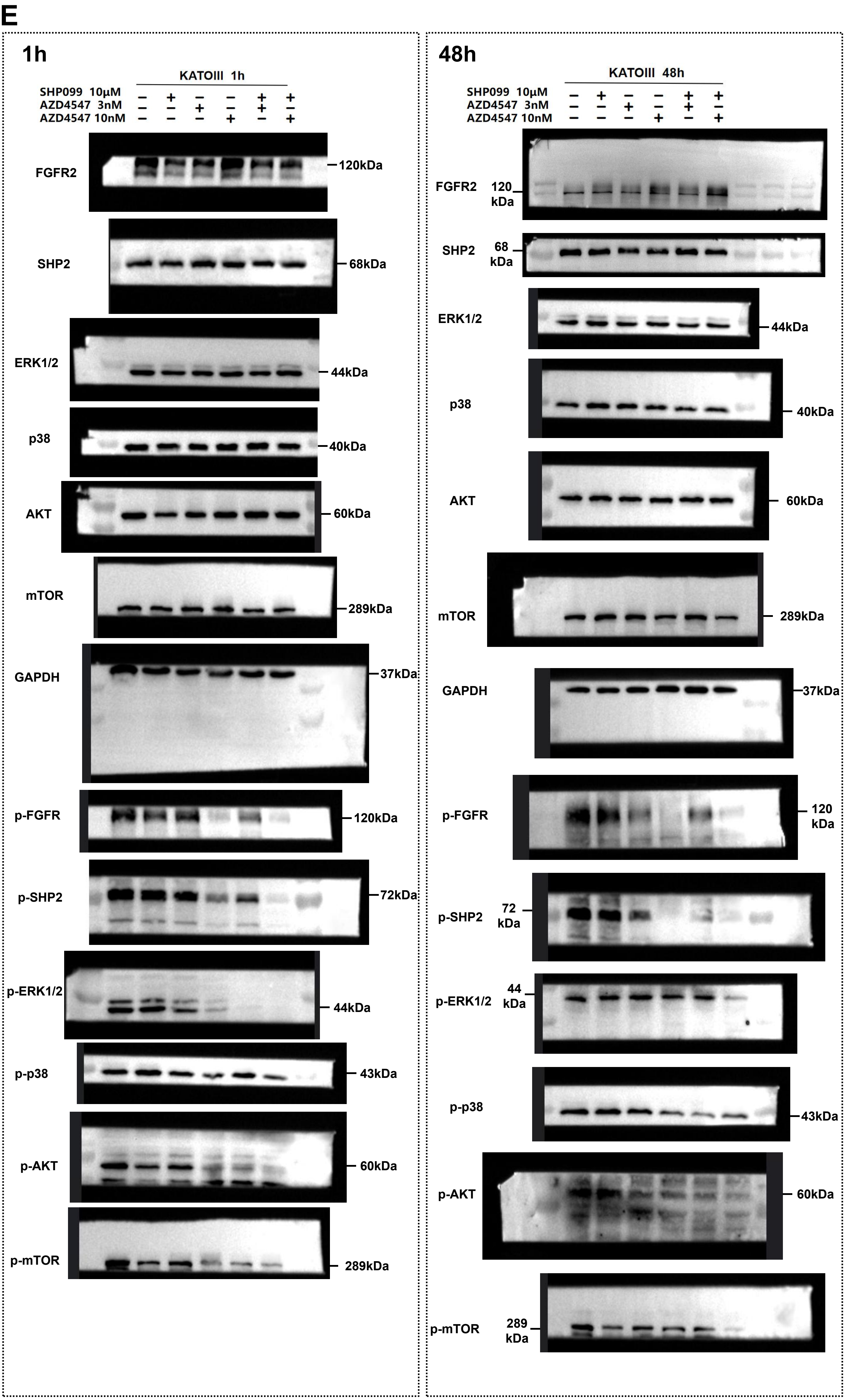
E
1h
KATOIII 1h
SHP099 10μM
AZD4547 3nM
AZD4547 10nM
FGFR2
120kDa
SHP2
68kDa
ERK1/2
44kDa
p38
40kDa
AKT
60kDa
mTOR
289kDa
GAPDH
37kDa
p-FGFR
120kDa
p-SHP2
72kDa
p-ERK1/2
44kDa
p-p38
43kDa
p-AKT
60kDa
p-mTOR
289kDa
48h
KATOIII 48h
SHP099 10μM
AZD4547 3nM
AZD4547 10nM
FGFR2
120 kDa
SHP2
68 kDa
ERK1/2
44kDa
p38
40kDa
AKT
60kDa
mTOR
289kDa
GAPDH
37kDa
p-FGFR
120 kDa
p-SHP2
72 kDa
p-ERK1/2
44 kDa
p-p38
43kDa
p-AKT
60kDa
p-mTOR
289 kDa

Figure 2

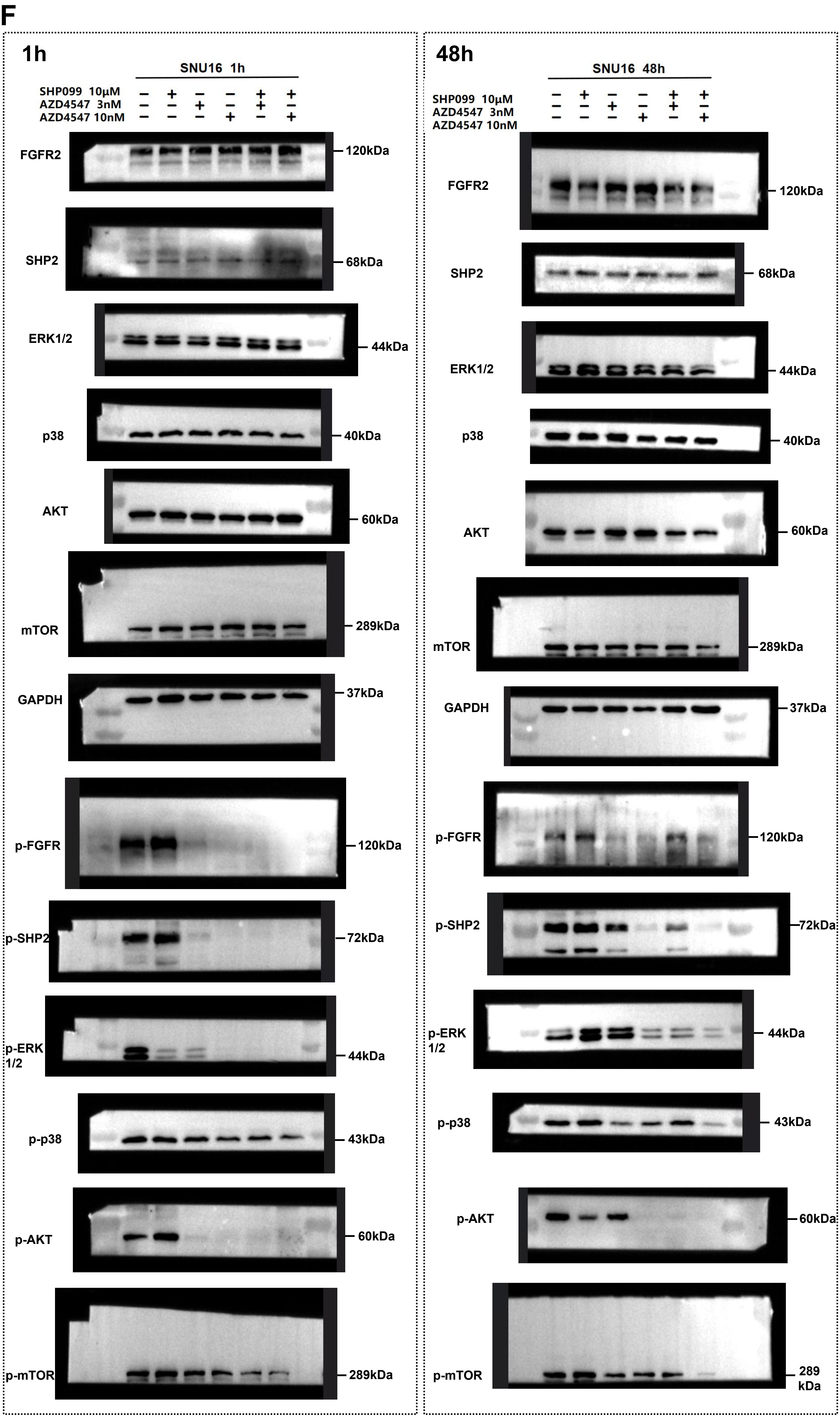
F
1h
SNU16 1h
SHP099 10μM
AZD4547 3nM
AZD4547 10nM
FGFR2
120kDa
SHP2
68kDa
ERK1/2
44kDa
p38
40kDa
AKT
60kDa
mTOR
289kDa
GAPDH
37kDa
p-FGFR
120kDa
p-SHP2
72kDa
p-ERK 1/2
44kDa
p-p38
43kDa
p-AKT
60kDa
p-mTOR
289kDa
48h
SNU16 48h
SHP099 10μM
AZD4547 3nM
AZD4547 10nM
FGFR2
120kDa
SHP2
68kDa
ERK1/2
44kDa
p38
40kDa
AKT
60kDa
mTOR
289kDa
GAPDH
37kDa
p-FGFR
120kDa
p-SHP2
72kDa
p-ERK 1/2
44kDa
p-p38
43kDa
p-AKT
60kDa
p-mTOR
289 kDa
