# Supplementary figures and images for: Blocking SHP2 benefits FGFR2 inhibitor and overcomes its resistance in FGFR2-amplified gastric cancer (part 2 of 3)
Source: eLife. 2026 Mar 23;14:RP104060. doi: 10.7554/eLife.104060 (PMC13008354; doi:10.7554/eLife.104060)

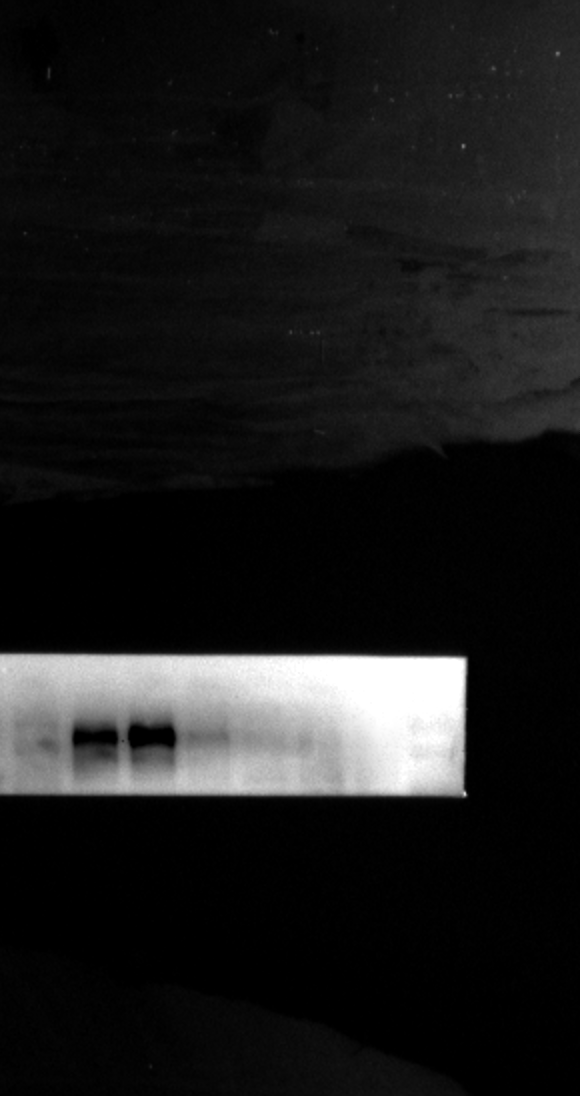

Supplement: Figure 2—source data 2. [file elife-104060-fig2-data2.zip › Figure 2-source data 2/2F/1h/p-FGFR/1 pfgfr merge.Tif]

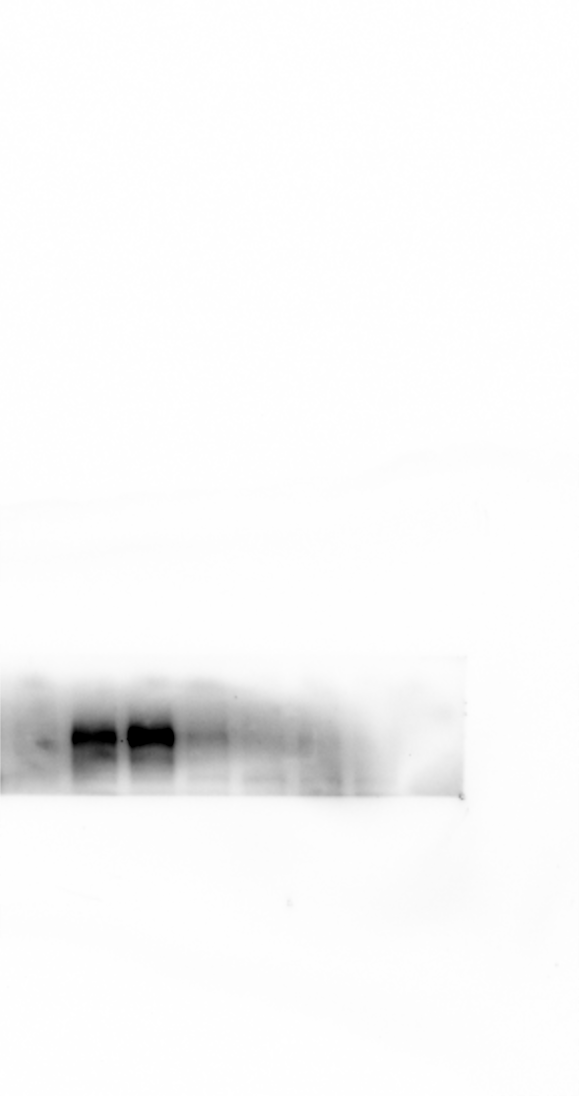

Supplement: Figure 2—source data 2. [file elife-104060-fig2-data2.zip › Figure 2-source data 2/2F/1h/p-FGFR/1 pfgfr.png]

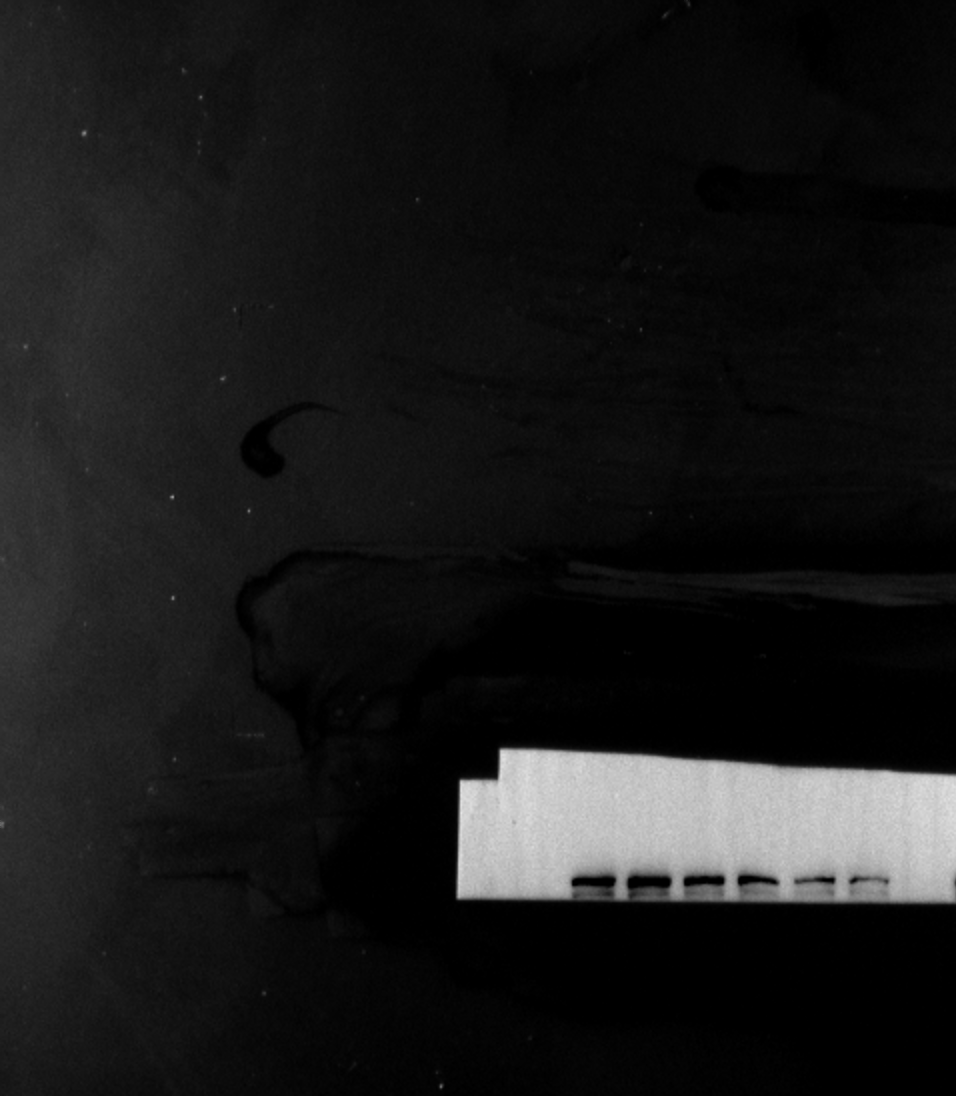

Supplement: Figure 2—source data 2. [file elife-104060-fig2-data2.zip › Figure 2-source data 2/2F/1h/p-mTOR/1 PMTOR MERGE.Tif]

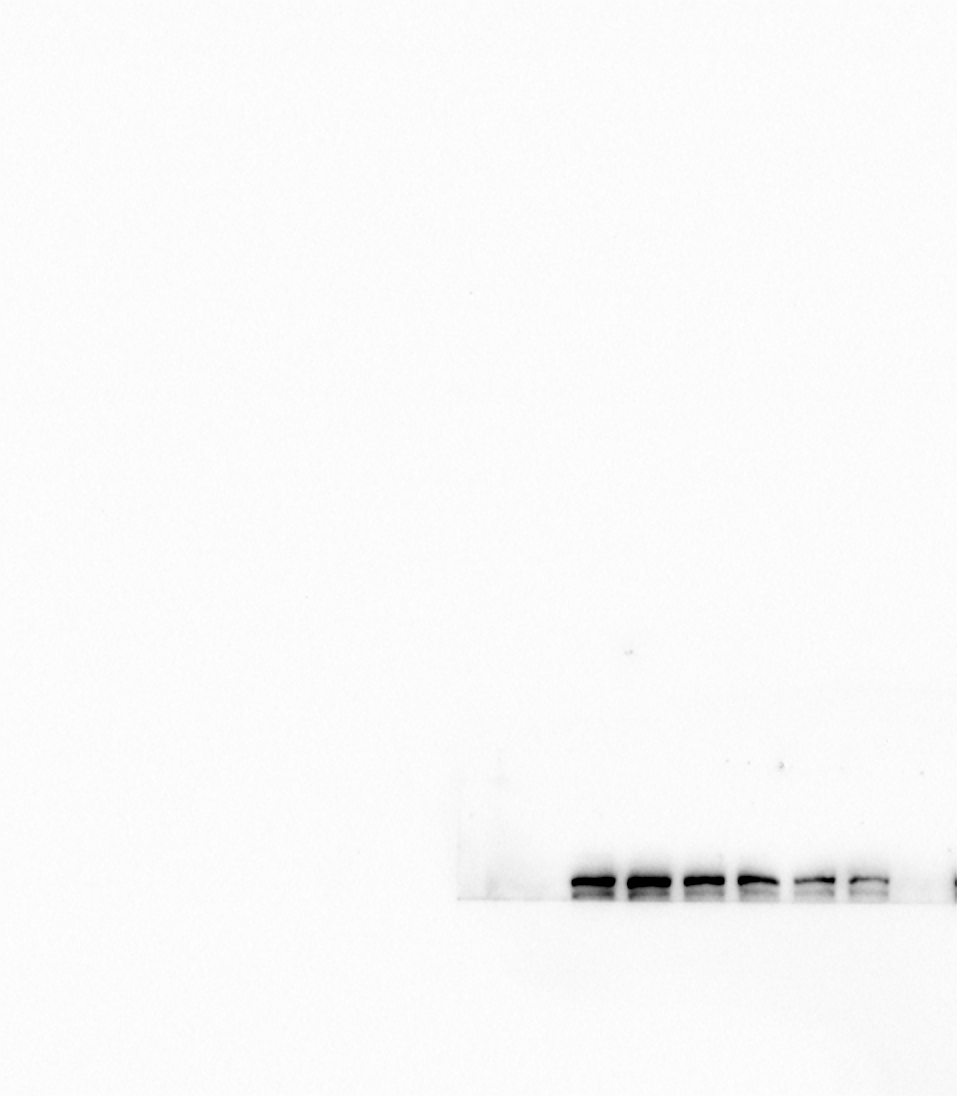

Supplement: Figure 2—source data 2. [file elife-104060-fig2-data2.zip › Figure 2-source data 2/2F/1h/p-mTOR/1 PMTOR.png]

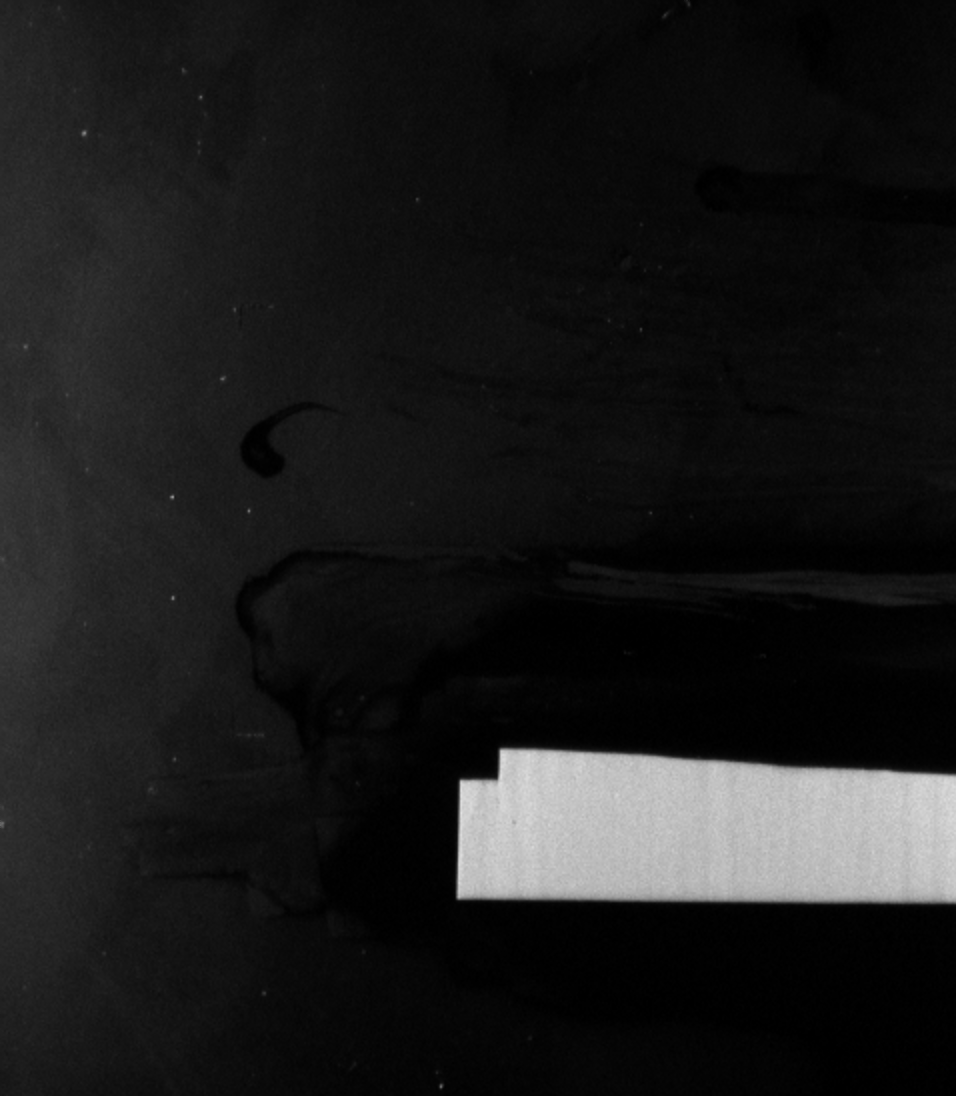

Supplement: Figure 2—source data 2. [file elife-104060-fig2-data2.zip › Figure 2-source data 2/2F/1h/p-mTOR/2 PMTOR WHITE.Tif]

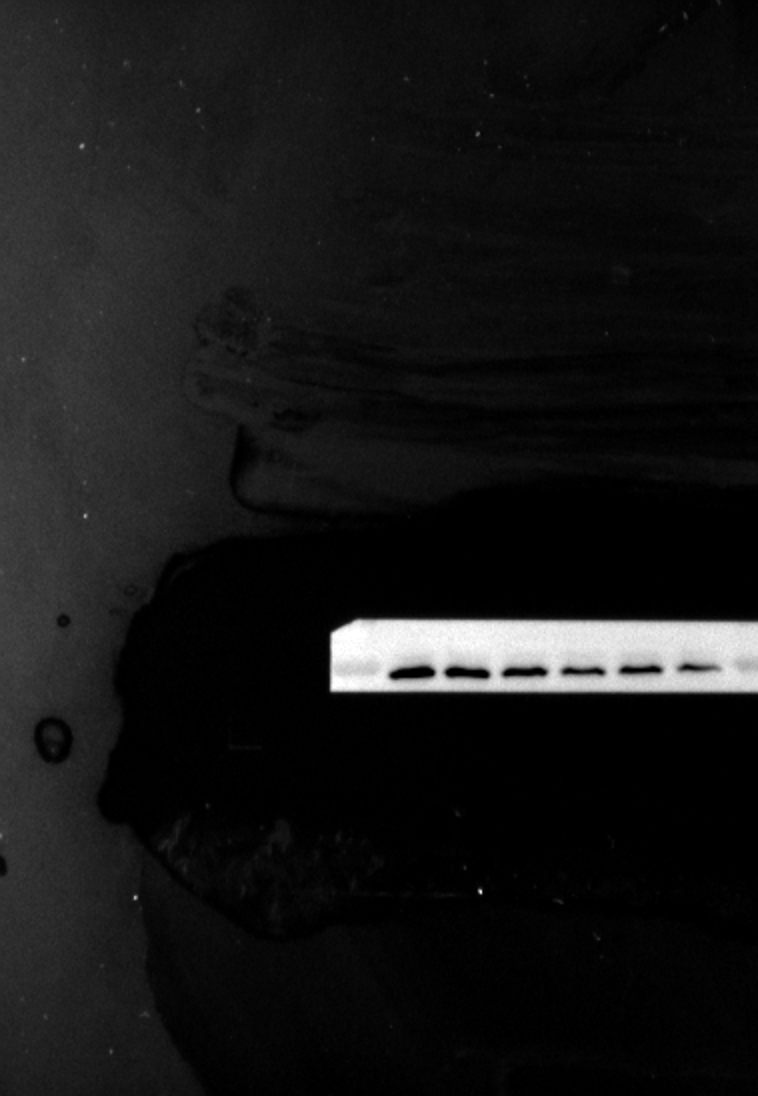

Supplement: Figure 2—source data 2. [file elife-104060-fig2-data2.zip › Figure 2-source data 2/2F/1h/p-p38/1 p-p38 merge.Tif]

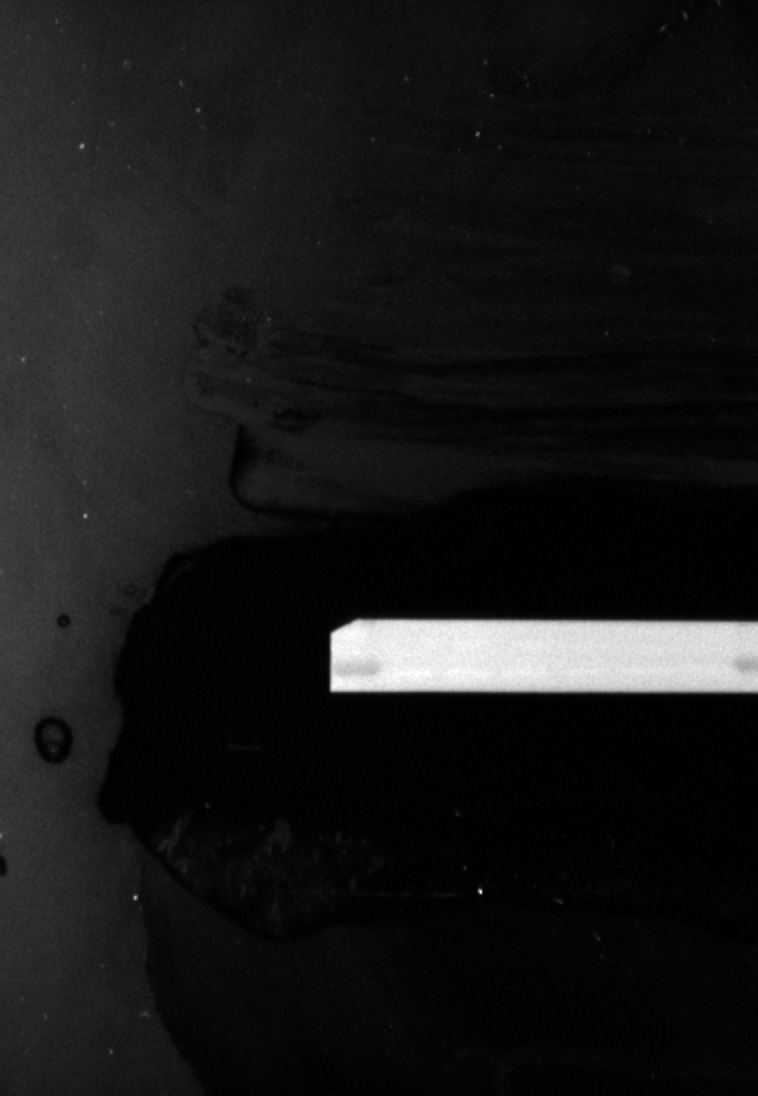

Supplement: Figure 2—source data 2. [file elife-104060-fig2-data2.zip › Figure 2-source data 2/2F/1h/p-p38/1 p-p38 white=.Tif]

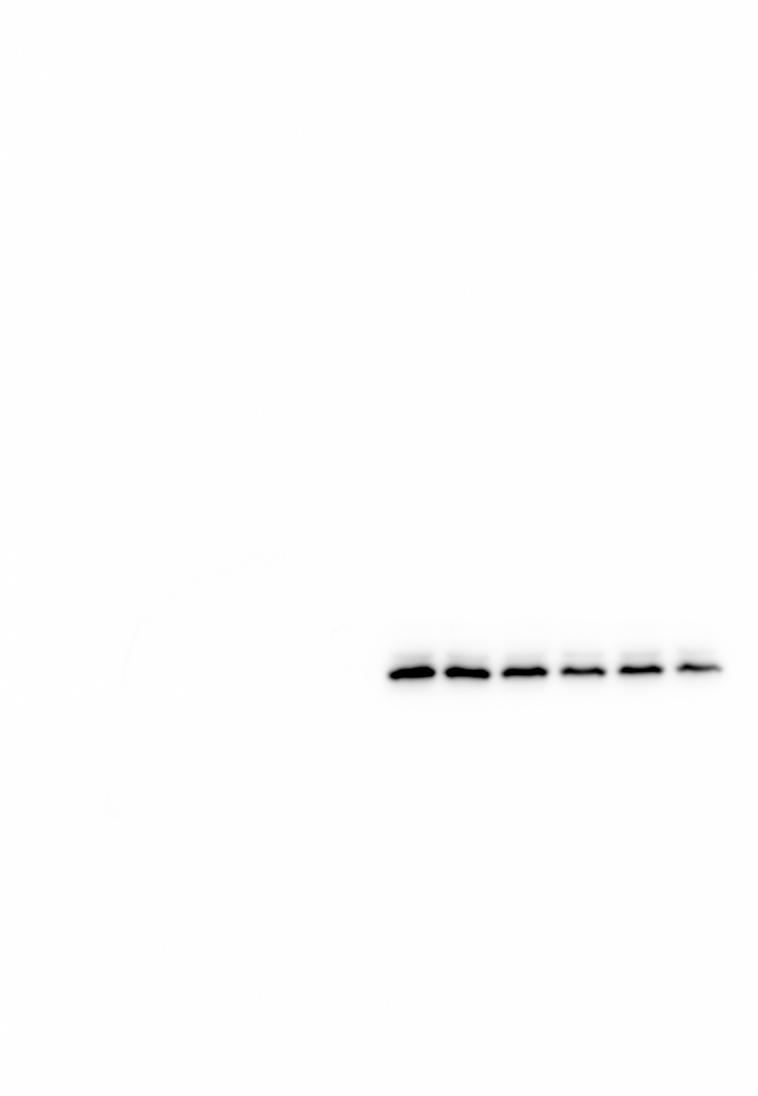

Supplement: Figure 2—source data 2. [file elife-104060-fig2-data2.zip › Figure 2-source data 2/2F/1h/p-p38/1 p-p38=.Tif]

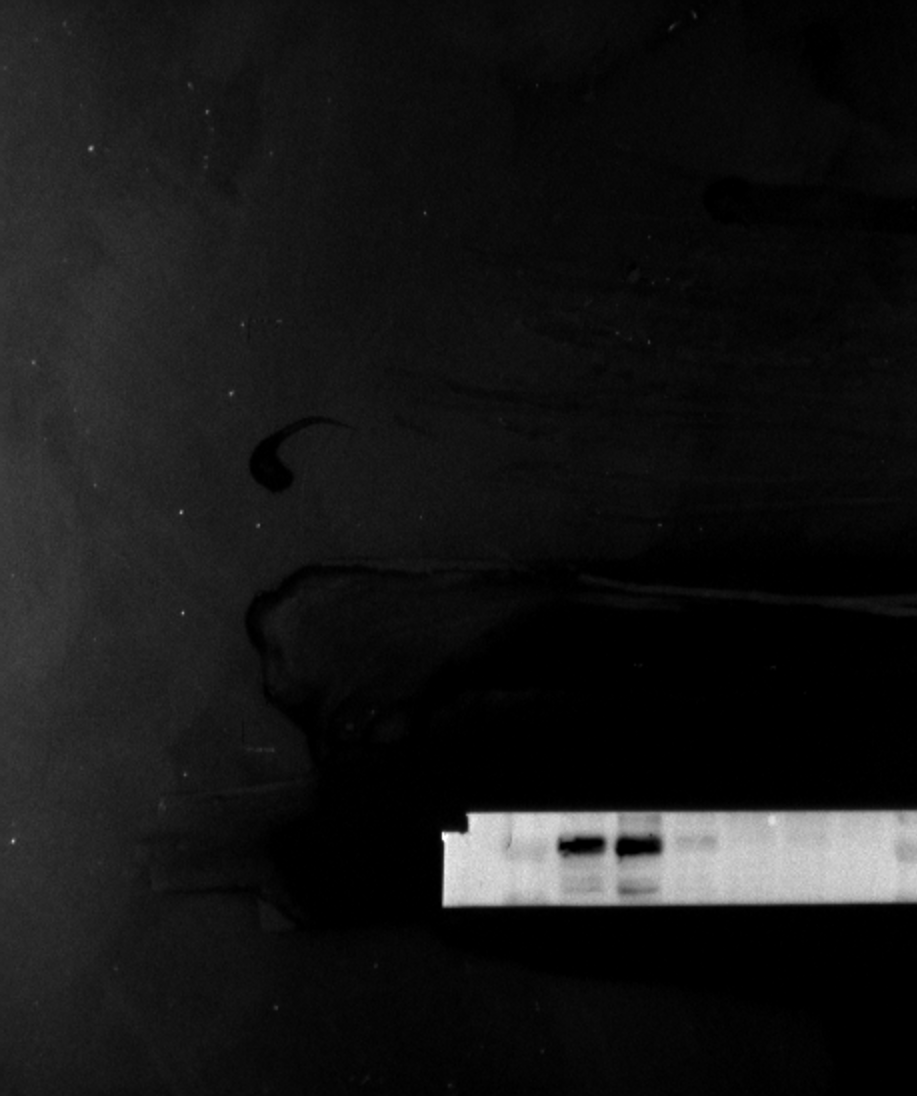

Supplement: Figure 2—source data 2. [file elife-104060-fig2-data2.zip › Figure 2-source data 2/2F/1h/p-SHP2/1 PSHP2 MERGE.Tif]

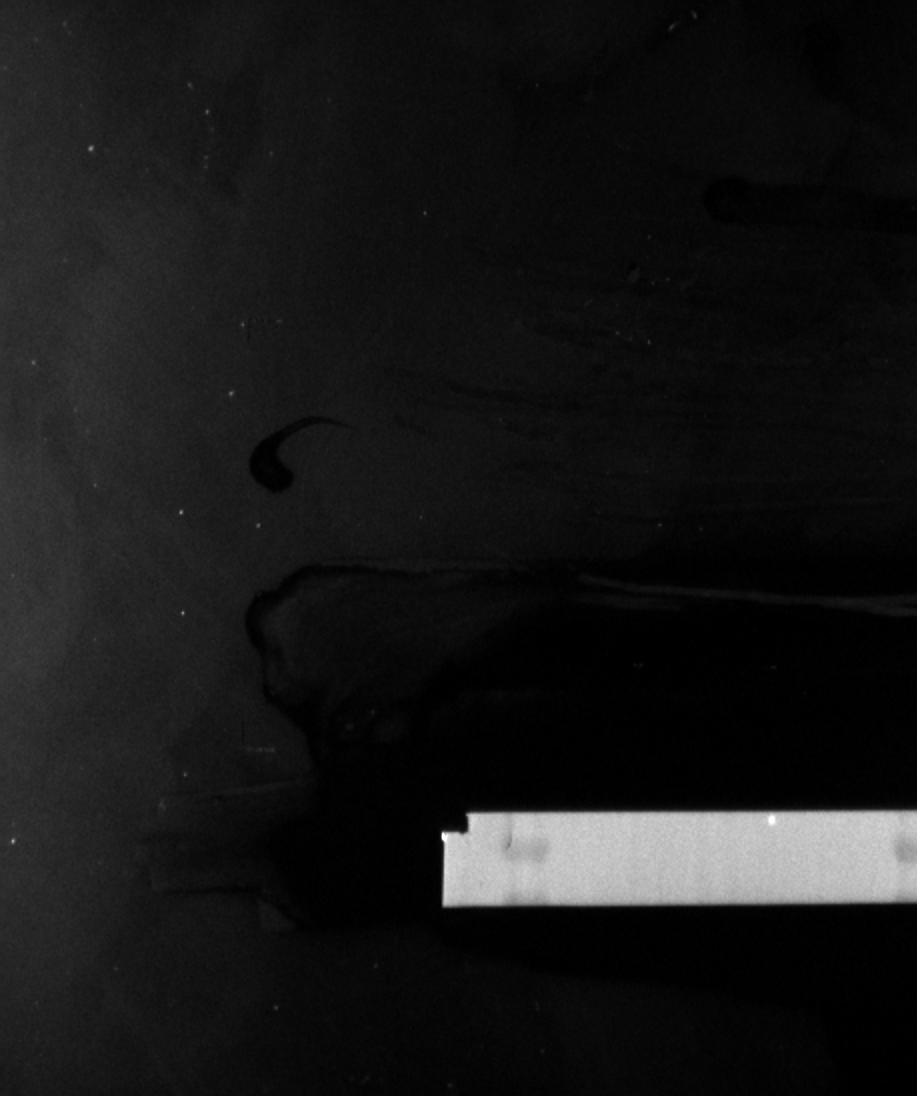

Supplement: Figure 2—source data 2. [file elife-104060-fig2-data2.zip › Figure 2-source data 2/2F/1h/p-SHP2/2 PSHP2 WHITE=.Tif]

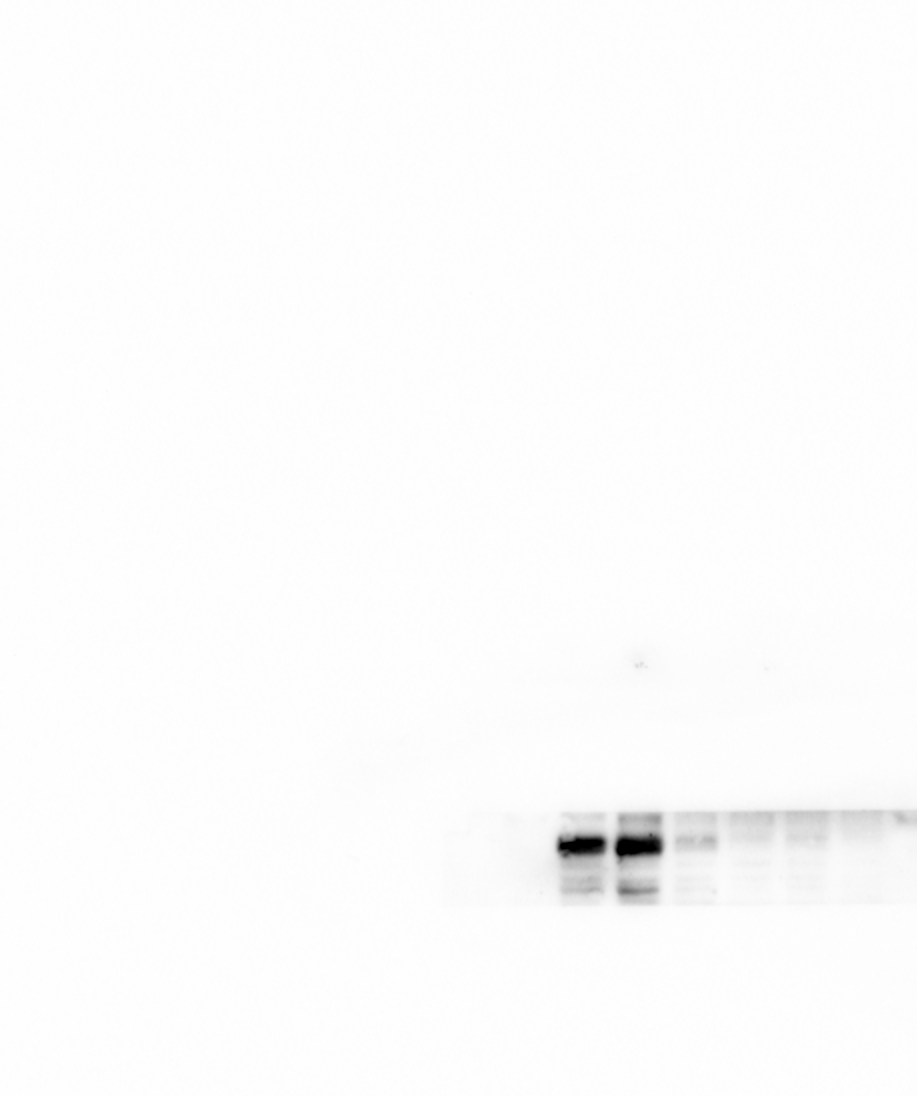

Supplement: Figure 2—source data 2. [file elife-104060-fig2-data2.zip › Figure 2-source data 2/2F/1h/p-SHP2/2 PSHP2=.Tif]

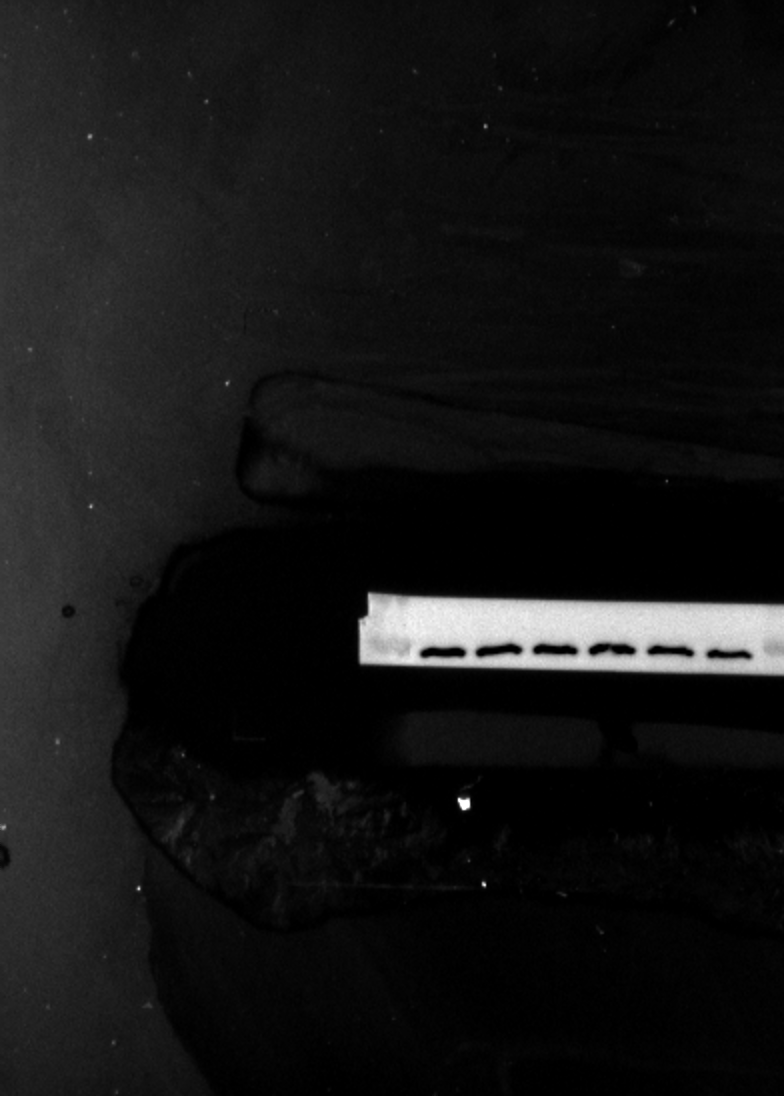

Supplement: Figure 2—source data 2. [file elife-104060-fig2-data2.zip › Figure 2-source data 2/2F/1h/p38/2 p38 merge.Tif]

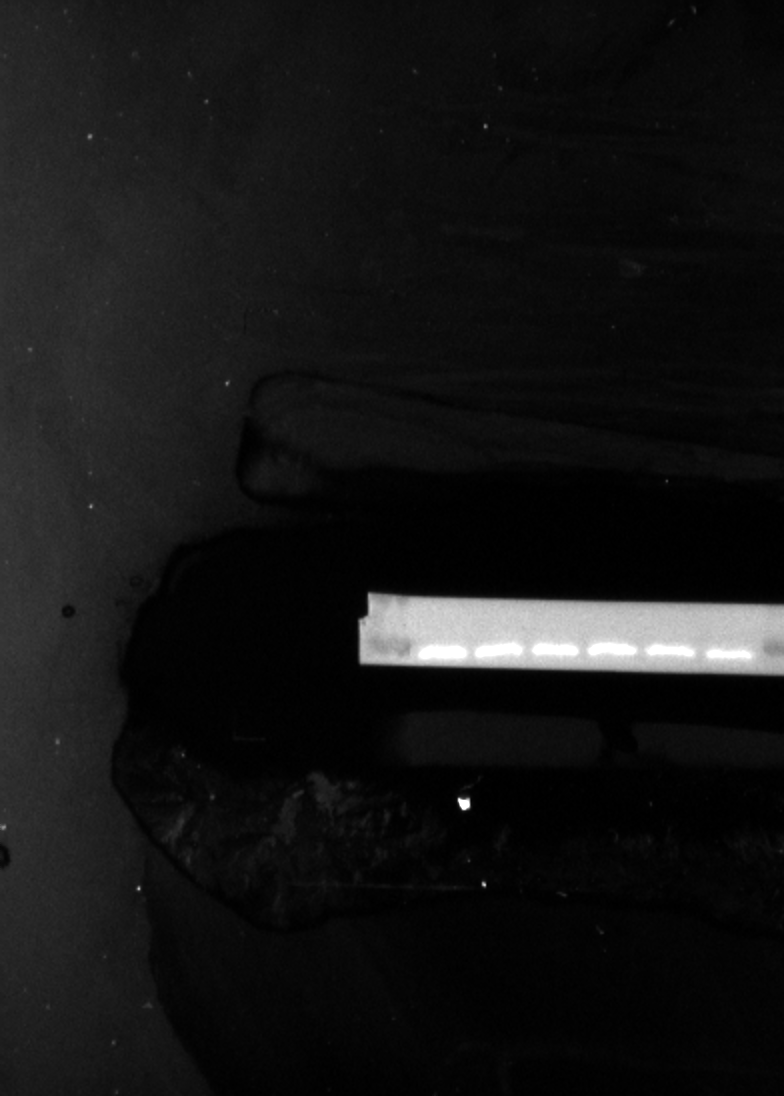

Supplement: Figure 2—source data 2. [file elife-104060-fig2-data2.zip › Figure 2-source data 2/2F/1h/p38/2 p38 white=.Tif]

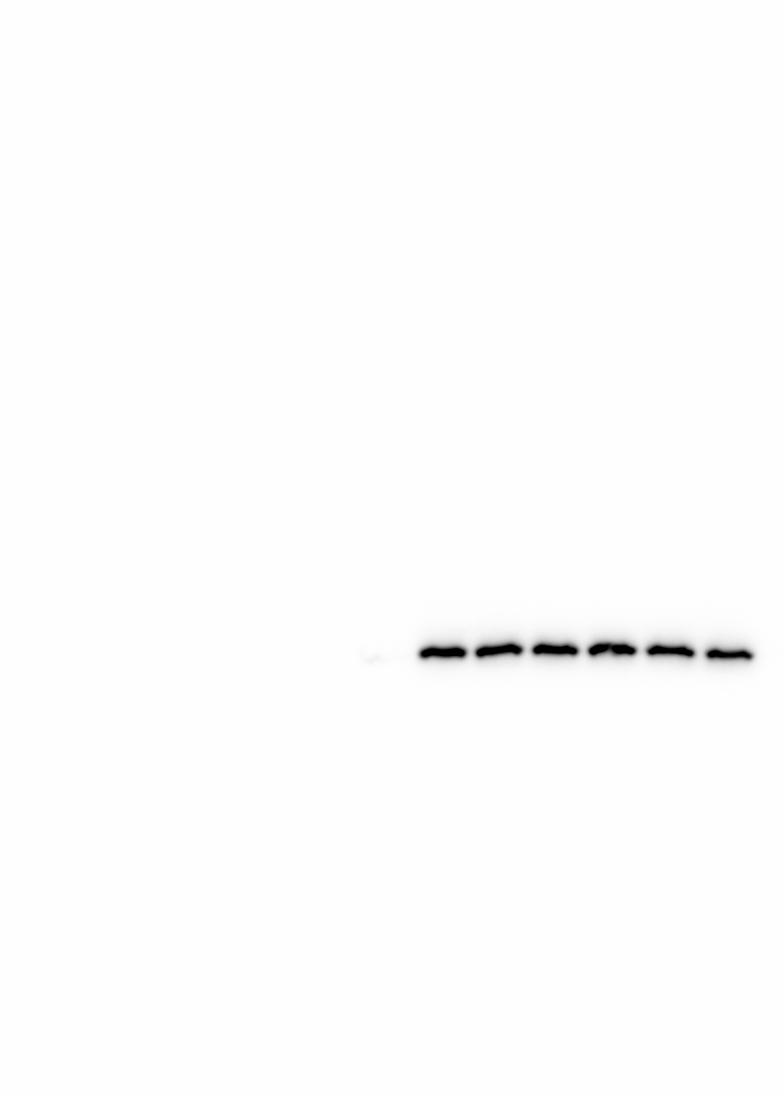

Supplement: Figure 2—source data 2. [file elife-104060-fig2-data2.zip › Figure 2-source data 2/2F/1h/p38/2 p38=.Tif]

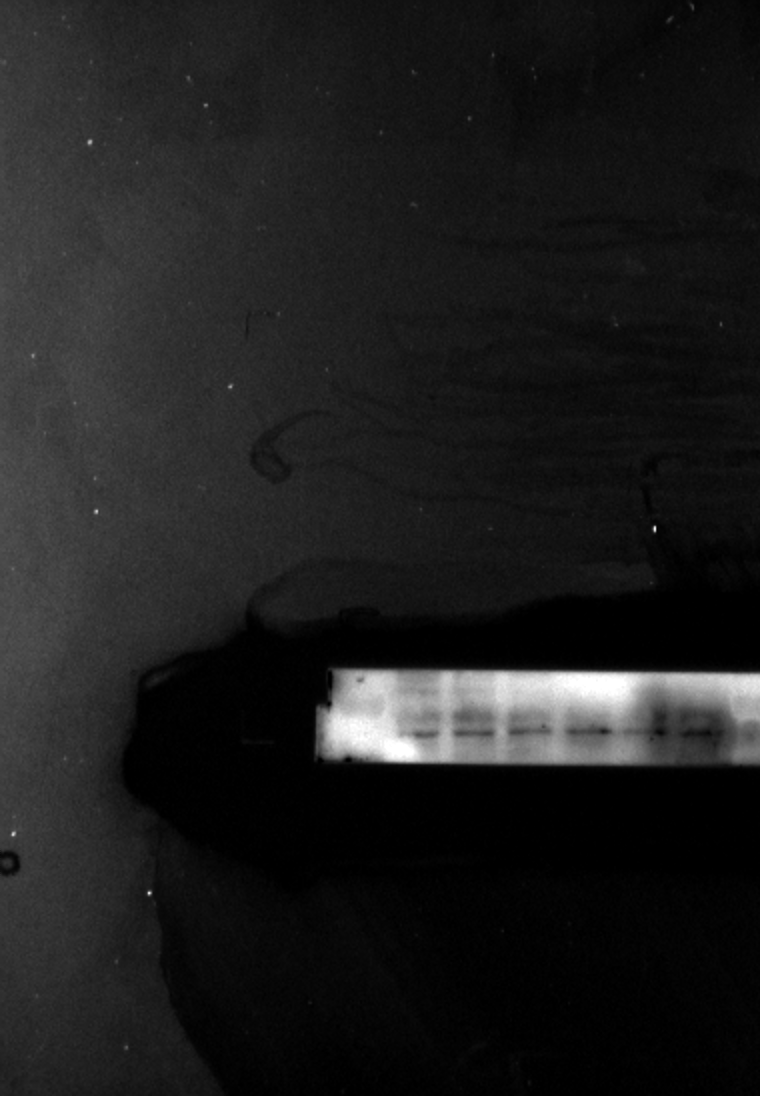

Supplement: Figure 2—source data 2. [file elife-104060-fig2-data2.zip › Figure 2-source data 2/2F/1h/SHP2/2 SHP2 MERGE.Tif]

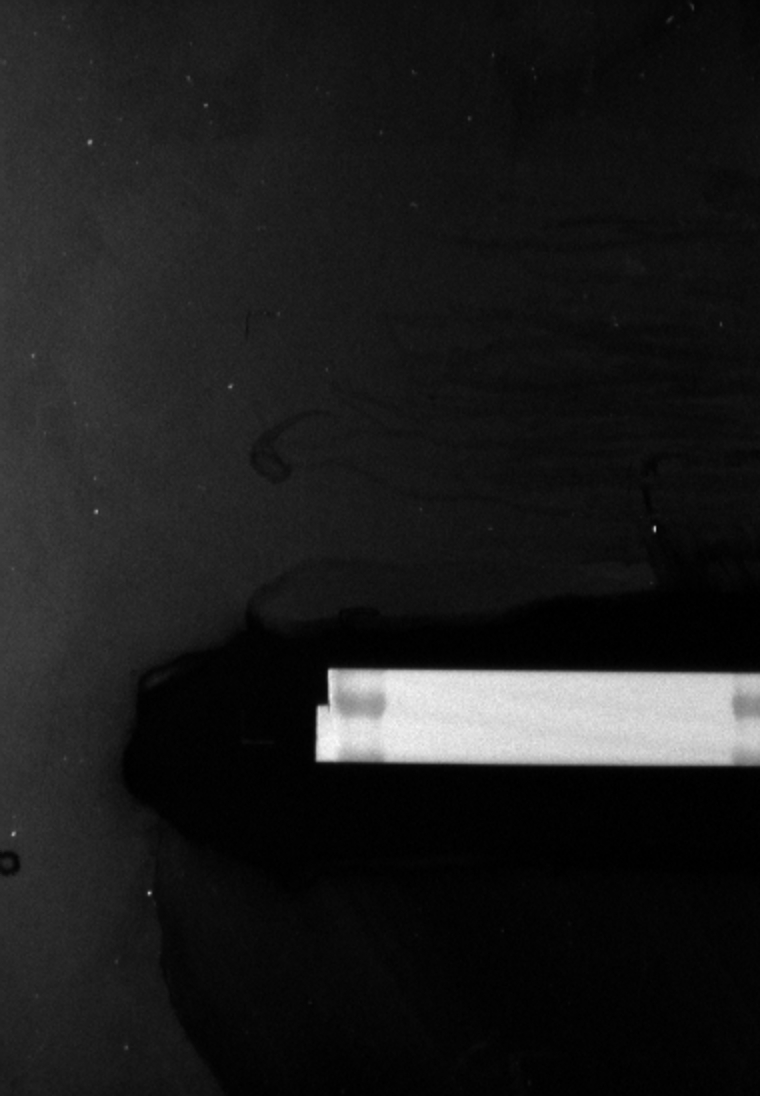

Supplement: Figure 2—source data 2. [file elife-104060-fig2-data2.zip › Figure 2-source data 2/2F/1h/SHP2/2 SHP2 WHITE.Tif]

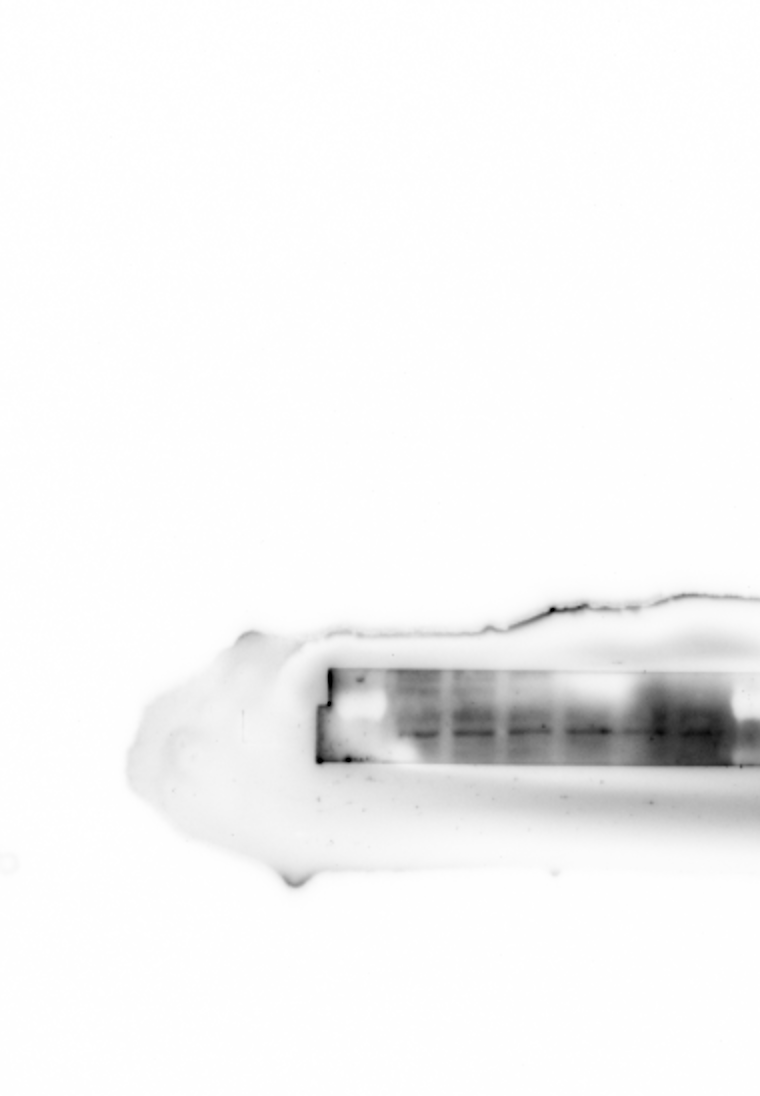

Supplement: Figure 2—source data 2. [file elife-104060-fig2-data2.zip › Figure 2-source data 2/2F/1h/SHP2/2 SHP2.Tif]

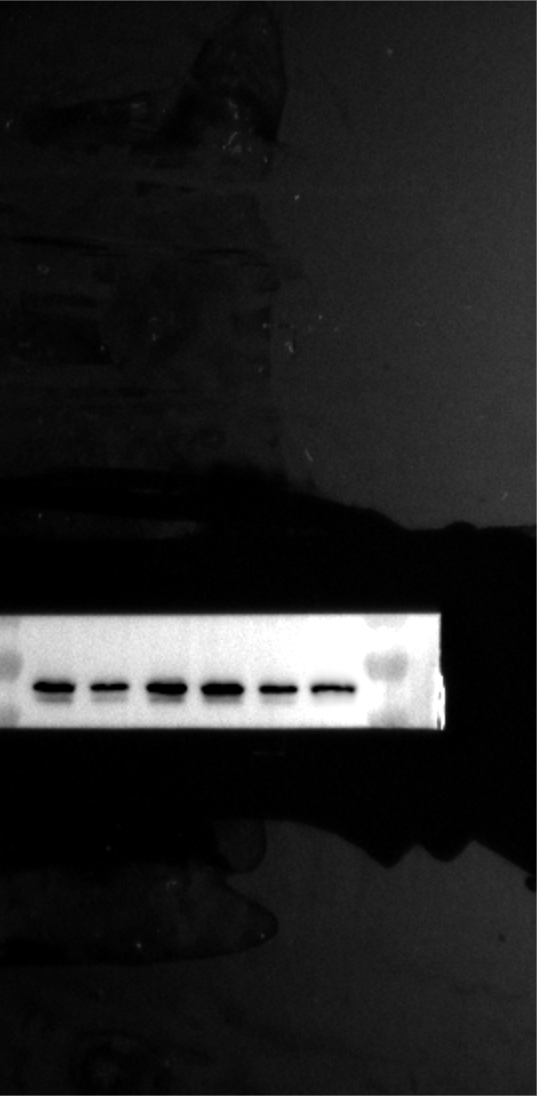

Supplement: Figure 2—source data 2. [file elife-104060-fig2-data2.zip › Figure 2-source data 2/2F/48h/AKT/akt merge=.png]

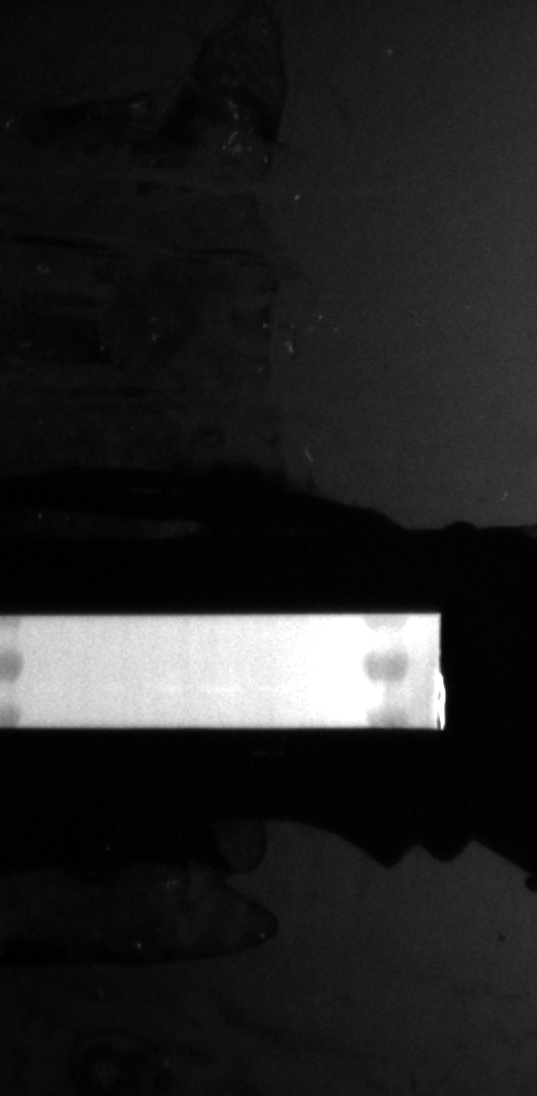

Supplement: Figure 2—source data 2. [file elife-104060-fig2-data2.zip › Figure 2-source data 2/2F/48h/AKT/akt white=.Tif]

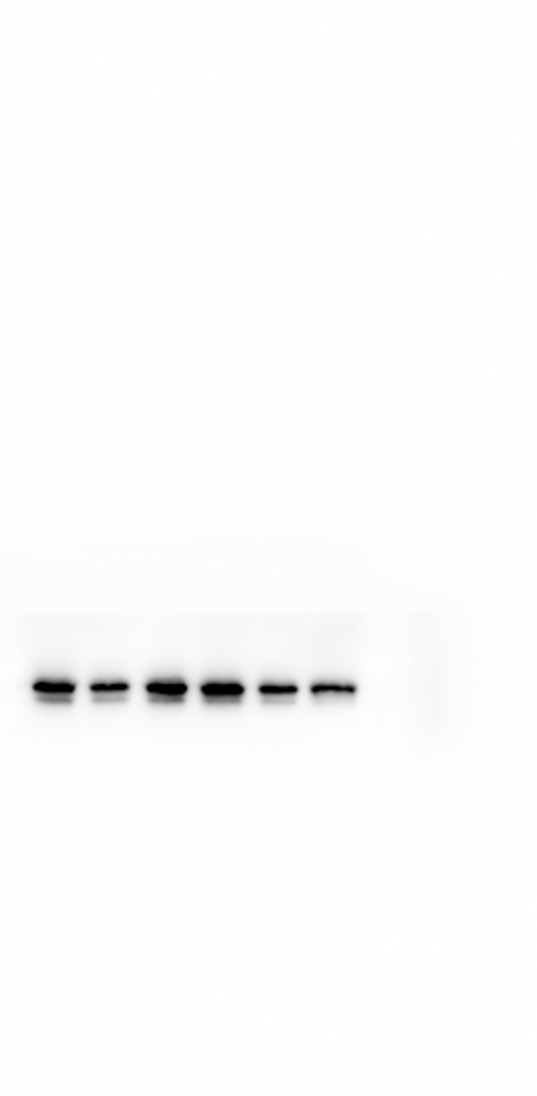

Supplement: Figure 2—source data 2. [file elife-104060-fig2-data2.zip › Figure 2-source data 2/2F/48h/AKT/akt=.Tif]

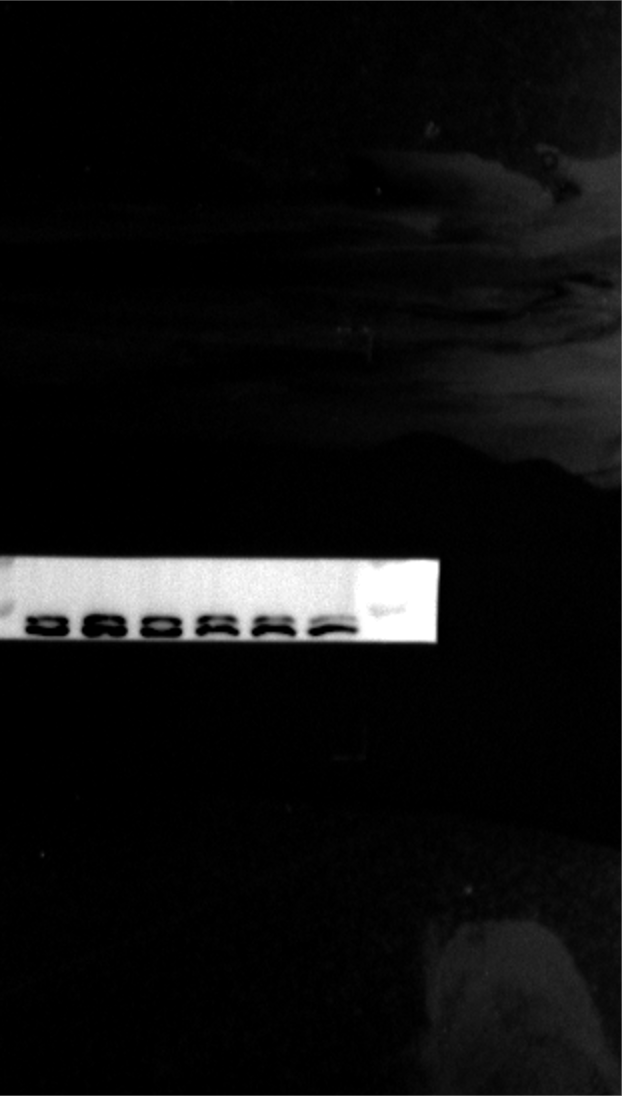

Supplement: Figure 2—source data 2. [file elife-104060-fig2-data2.zip › Figure 2-source data 2/2F/48h/ERK/2 ERK MERGE=.png]

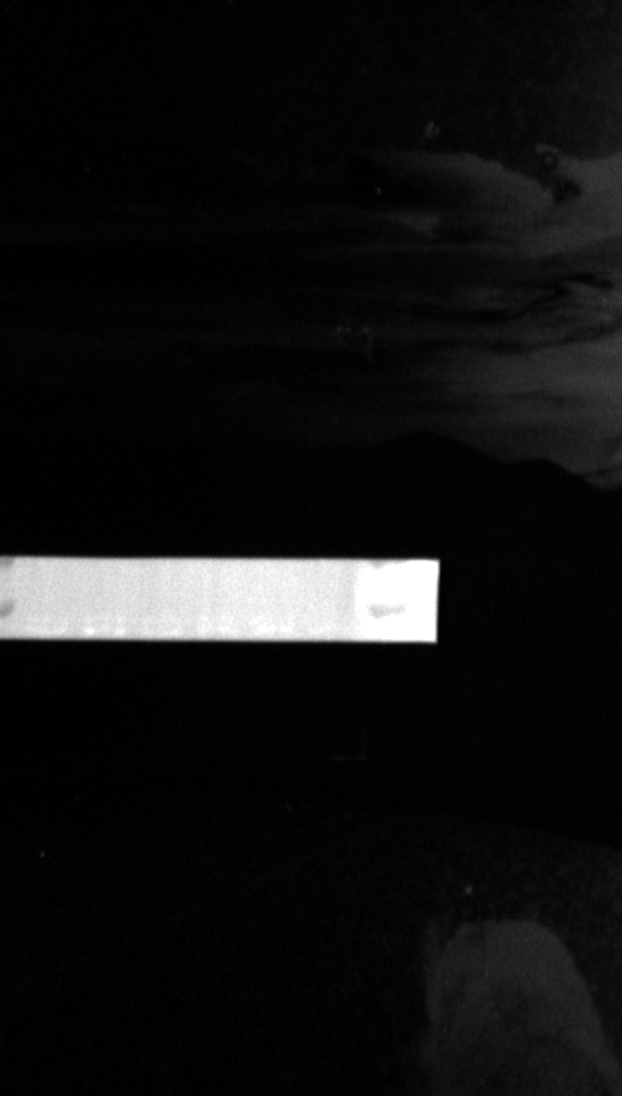

Supplement: Figure 2—source data 2. [file elife-104060-fig2-data2.zip › Figure 2-source data 2/2F/48h/ERK/2 ERK WHITE=.Tif]

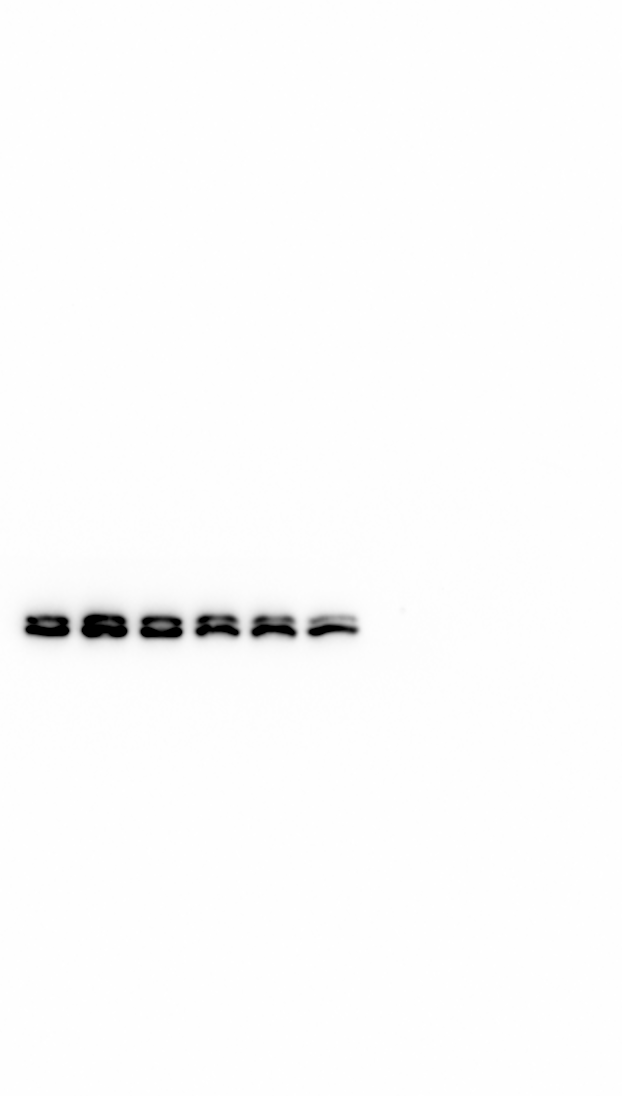

Supplement: Figure 2—source data 2. [file elife-104060-fig2-data2.zip › Figure 2-source data 2/2F/48h/ERK/2 ERK=.Tif]

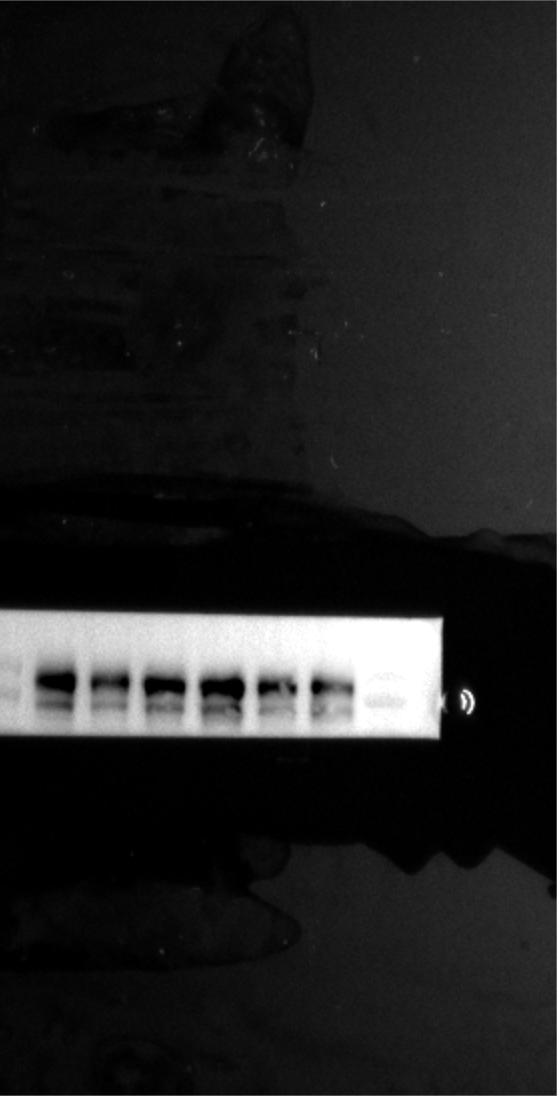

Supplement: Figure 2—source data 2. [file elife-104060-fig2-data2.zip › Figure 2-source data 2/2F/48h/FGFR2/fgfr2 merge=.png]

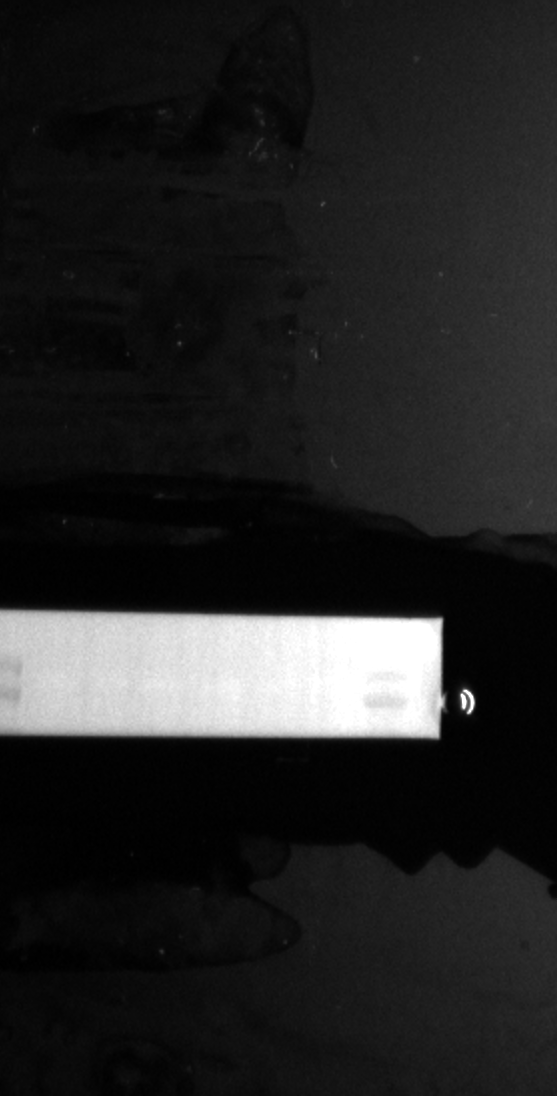

Supplement: Figure 2—source data 2. [file elife-104060-fig2-data2.zip › Figure 2-source data 2/2F/48h/FGFR2/fgfr2 white=.Tif]

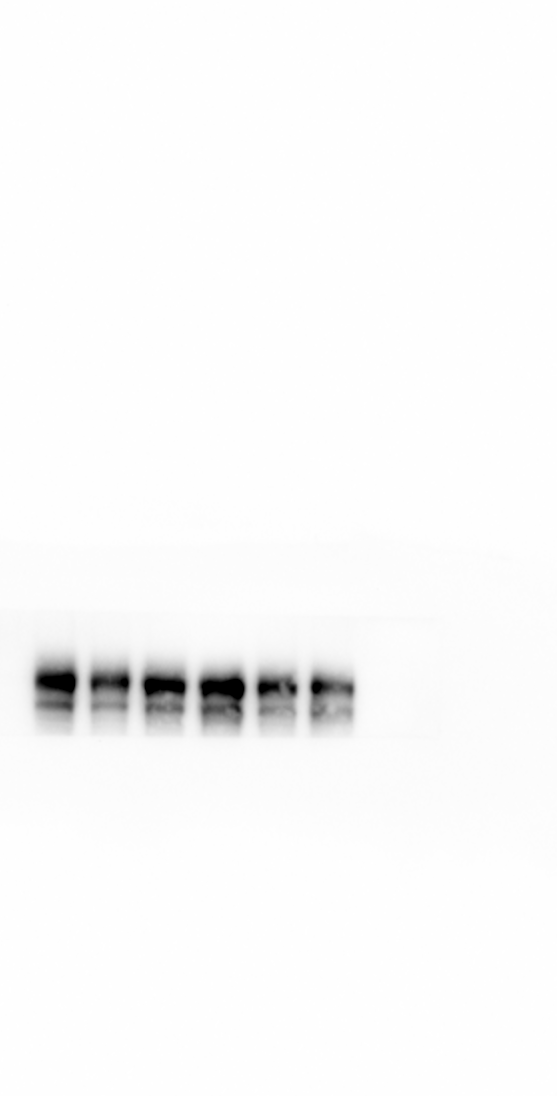

Supplement: Figure 2—source data 2. [file elife-104060-fig2-data2.zip › Figure 2-source data 2/2F/48h/FGFR2/fgfr2=.Tif]

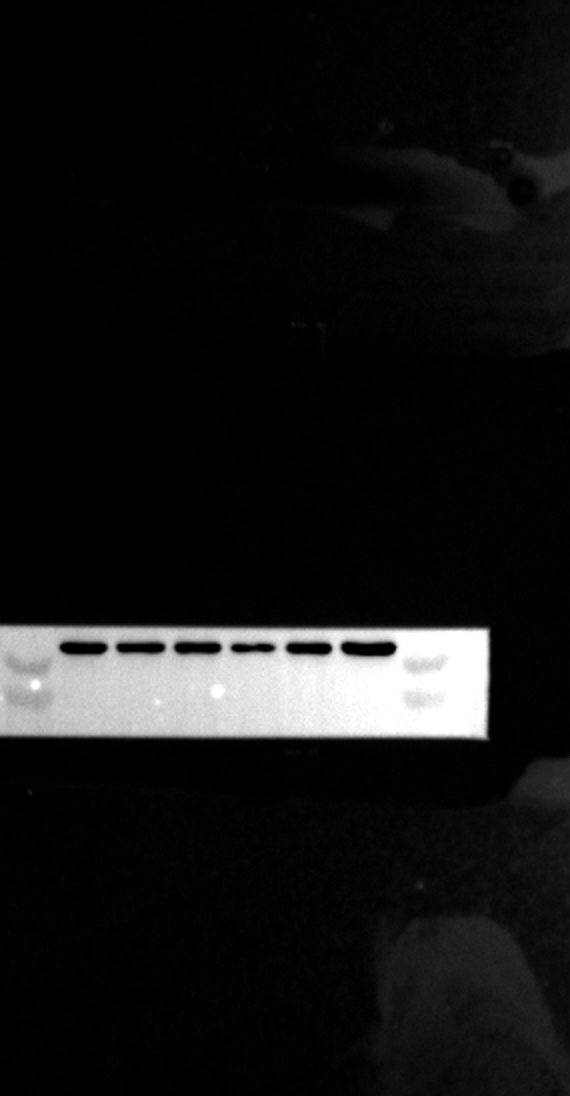

Supplement: Figure 2—source data 2. [file elife-104060-fig2-data2.zip › Figure 2-source data 2/2F/48h/GAPDH/1 gap MERGE.Tif]

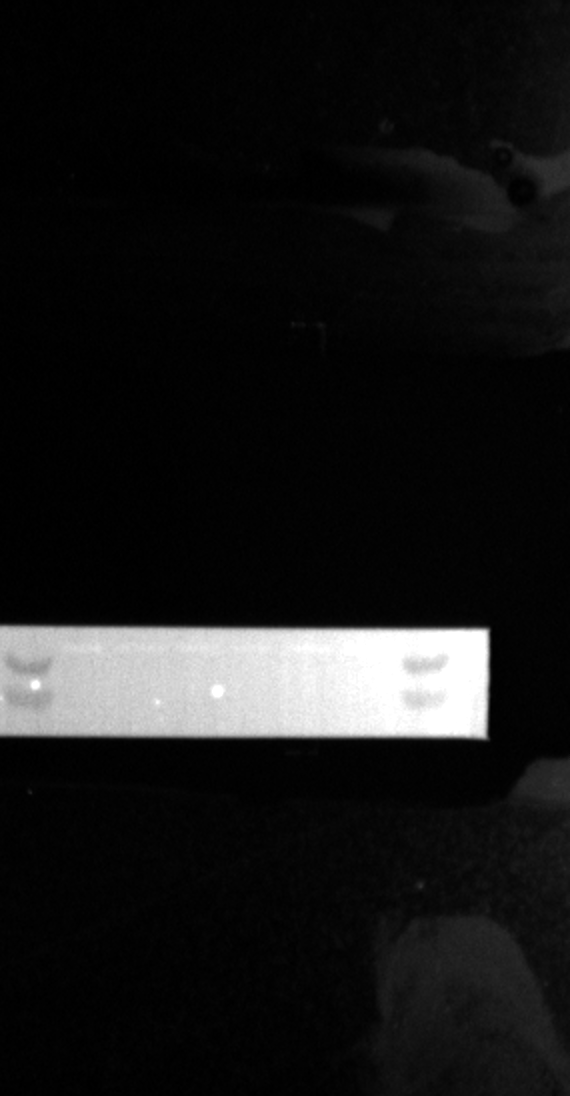

Supplement: Figure 2—source data 2. [file elife-104060-fig2-data2.zip › Figure 2-source data 2/2F/48h/GAPDH/1 gap WHITE.Tif]

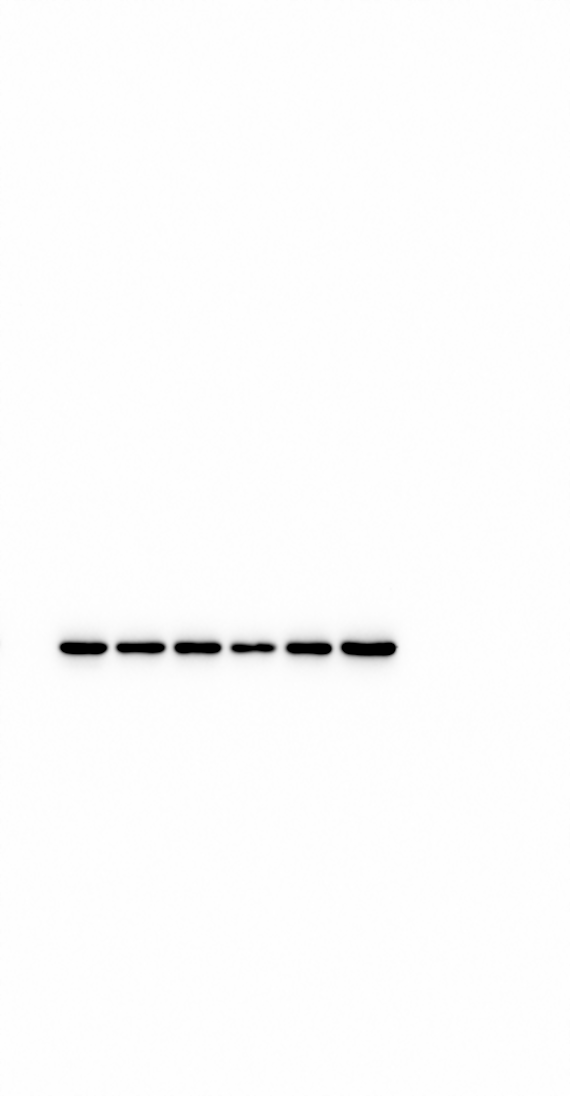

Supplement: Figure 2—source data 2. [file elife-104060-fig2-data2.zip › Figure 2-source data 2/2F/48h/GAPDH/1 gap.png]

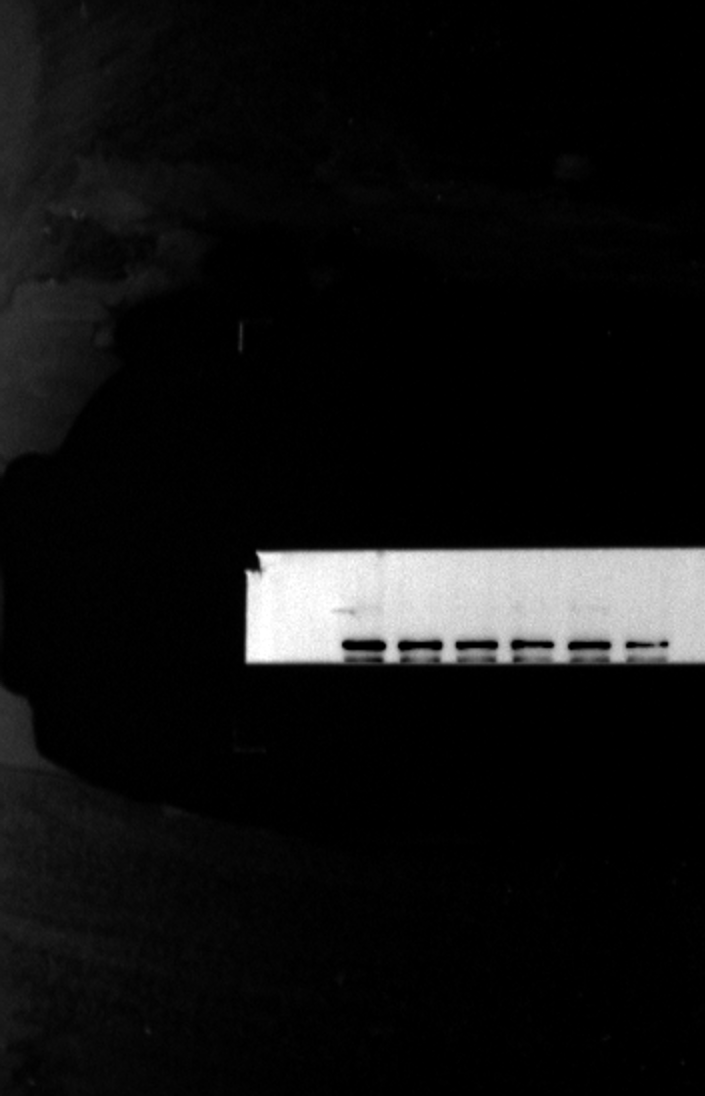

Supplement: Figure 2—source data 2. [file elife-104060-fig2-data2.zip › Figure 2-source data 2/2F/48h/mTOR/2 MTOR MERGE.Tif]

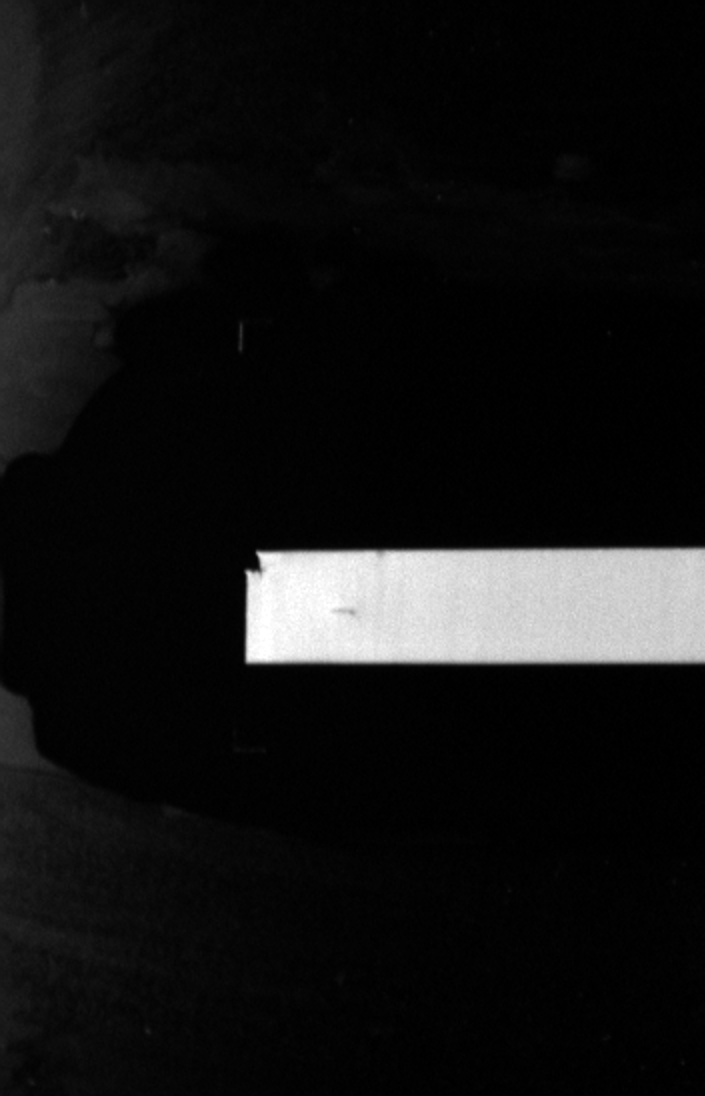

Supplement: Figure 2—source data 2. [file elife-104060-fig2-data2.zip › Figure 2-source data 2/2F/48h/mTOR/2 MTOR WHITE=.Tif]

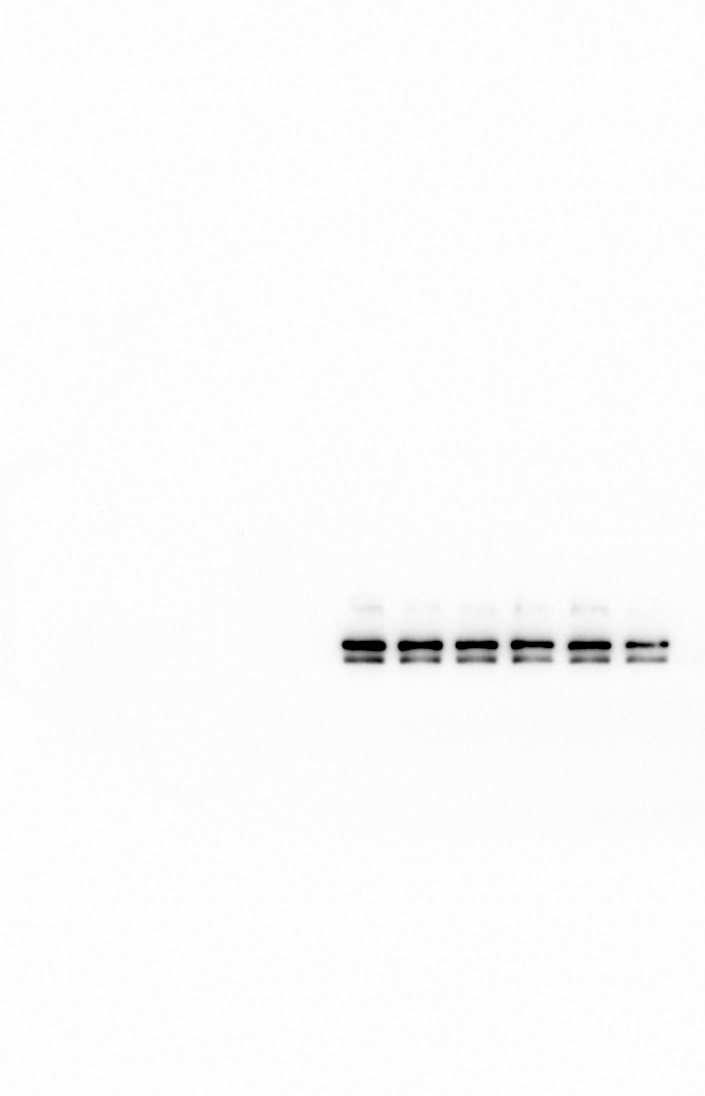

Supplement: Figure 2—source data 2. [file elife-104060-fig2-data2.zip › Figure 2-source data 2/2F/48h/mTOR/2 MTOR=.Tif]

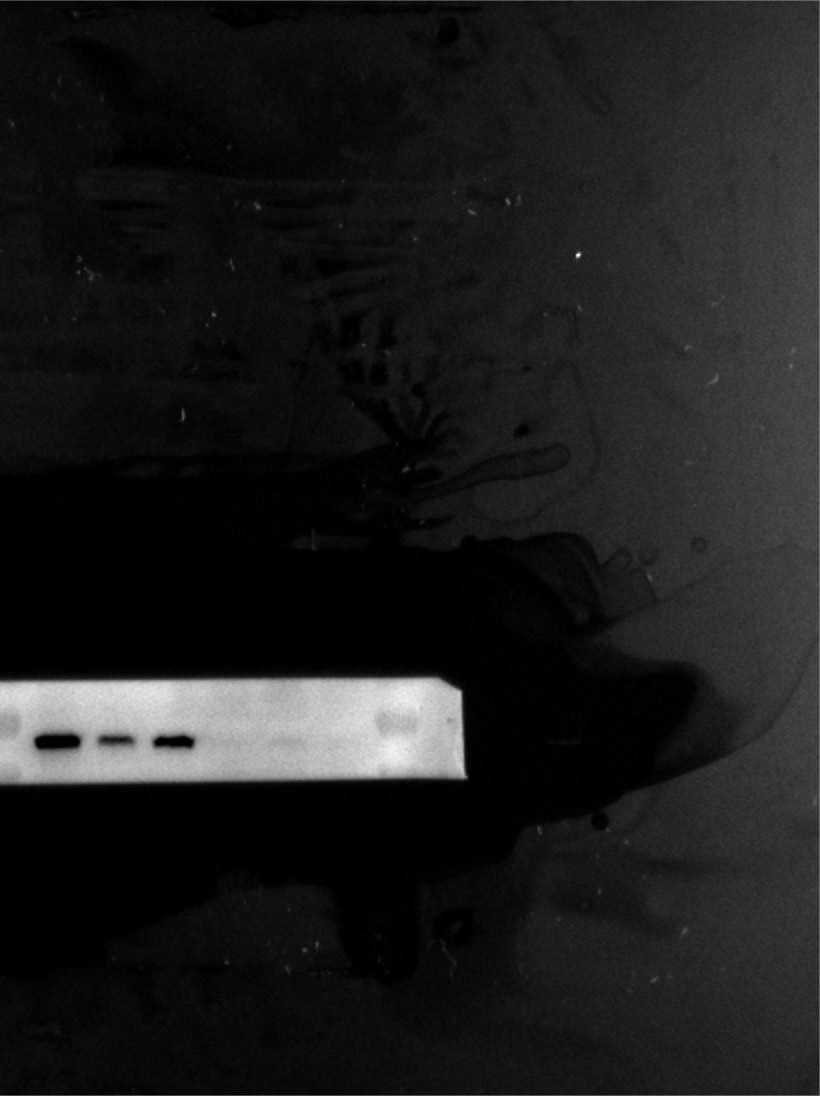

Supplement: Figure 2—source data 2. [file elife-104060-fig2-data2.zip › Figure 2-source data 2/2F/48h/p-AKT/p-akt merge=.jpg]

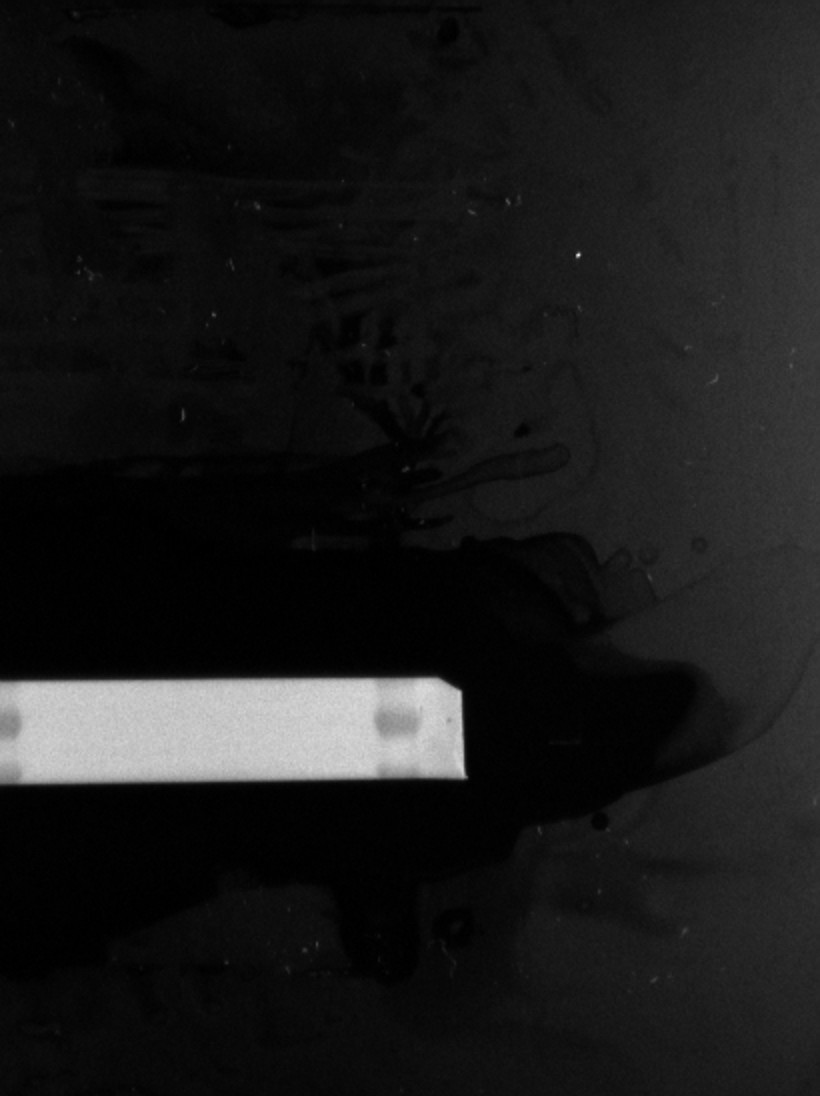

Supplement: Figure 2—source data 2. [file elife-104060-fig2-data2.zip › Figure 2-source data 2/2F/48h/p-AKT/p-akt white=.jpg]

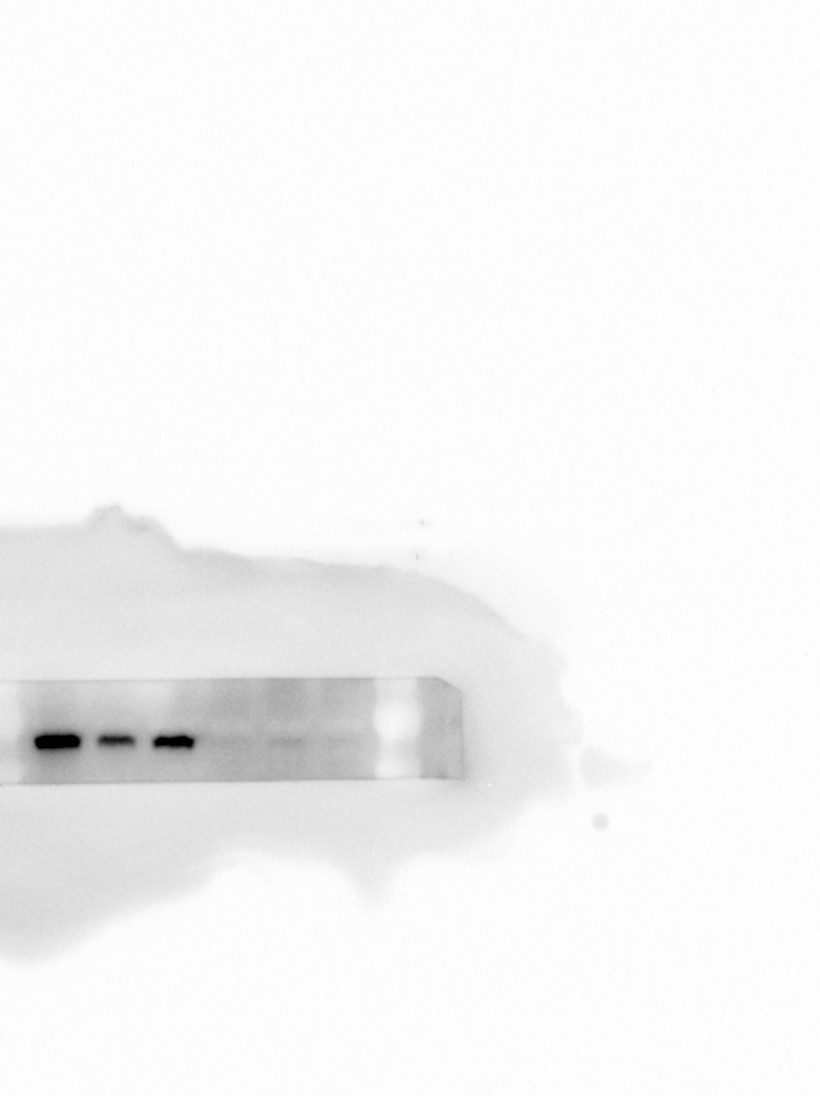

Supplement: Figure 2—source data 2. [file elife-104060-fig2-data2.zip › Figure 2-source data 2/2F/48h/p-AKT/p-akt=.jpg]

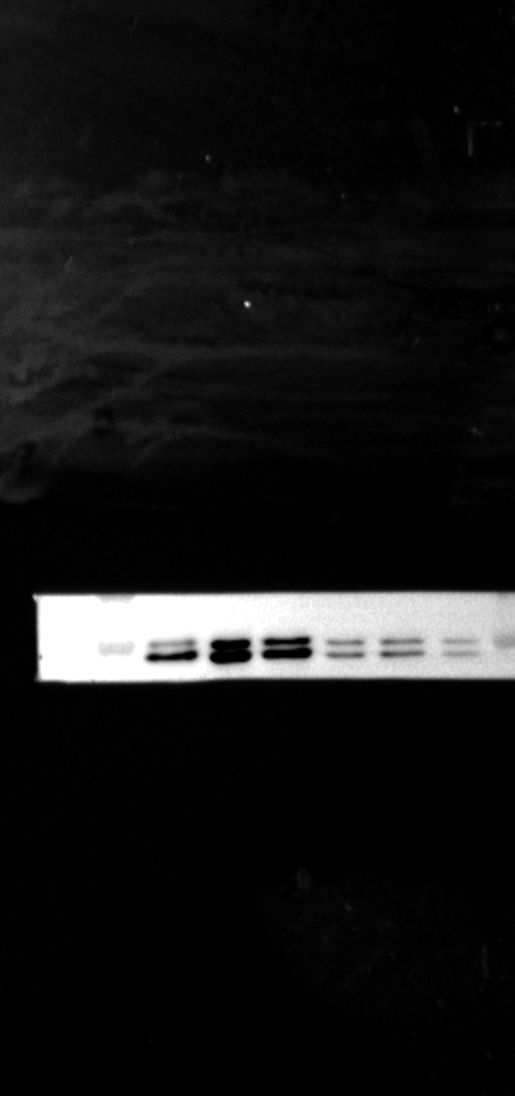

Supplement: Figure 2—source data 2. [file elife-104060-fig2-data2.zip › Figure 2-source data 2/2F/48h/p-ERK/1 perk merge.Tif]

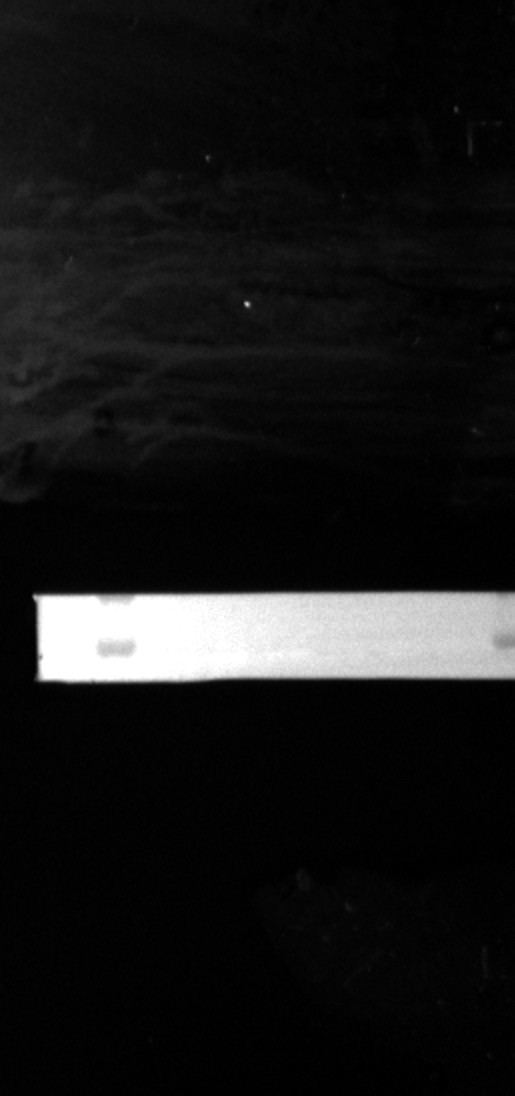

Supplement: Figure 2—source data 2. [file elife-104060-fig2-data2.zip › Figure 2-source data 2/2F/48h/p-ERK/p-erk white=.jpg]

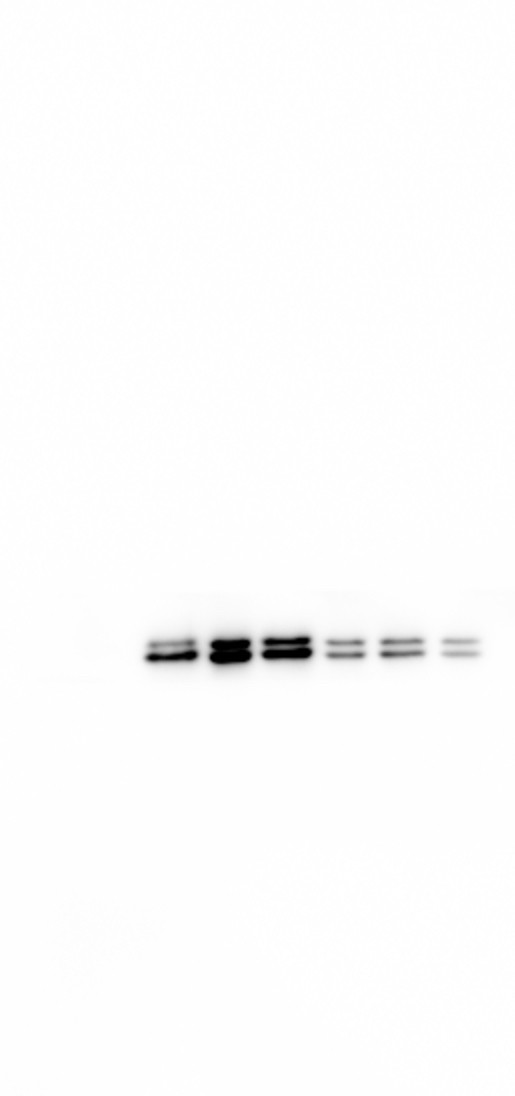

Supplement: Figure 2—source data 2. [file elife-104060-fig2-data2.zip › Figure 2-source data 2/2F/48h/p-ERK/p-erk=.jpg]

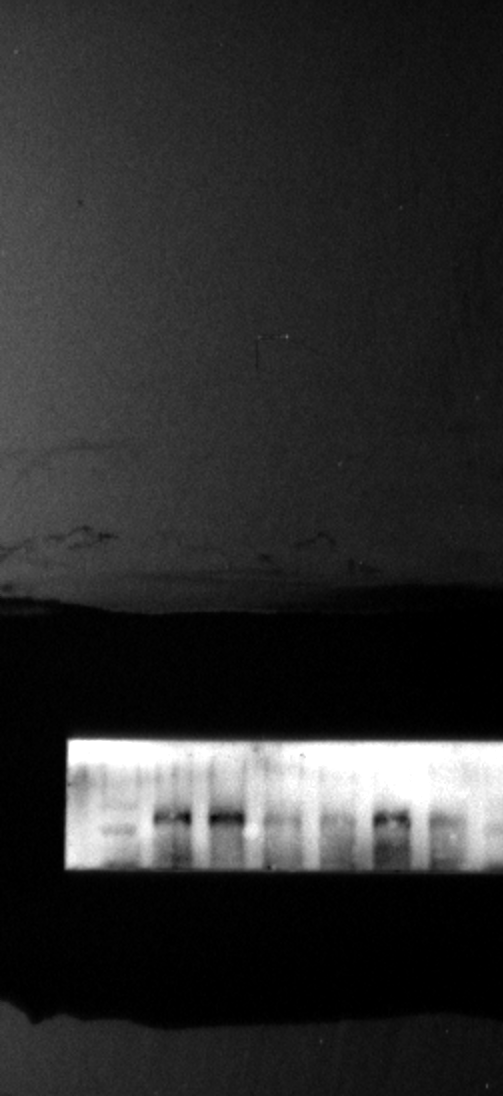

Supplement: Figure 2—source data 2. [file elife-104060-fig2-data2.zip › Figure 2-source data 2/2F/48h/p-FGFR/1 p-fgfr merge.Tif]

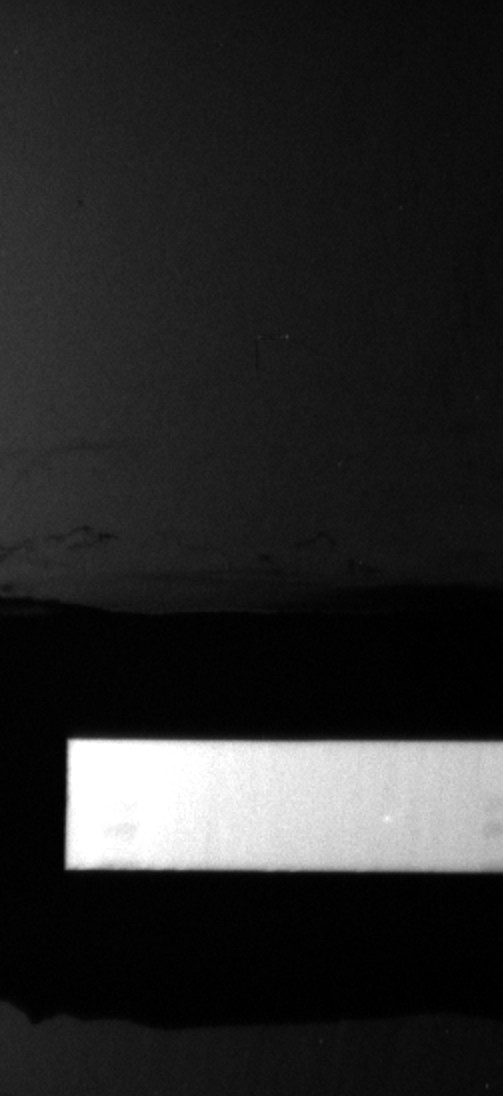

Supplement: Figure 2—source data 2. [file elife-104060-fig2-data2.zip › Figure 2-source data 2/2F/48h/p-FGFR/p-fgfr white=.jpg]

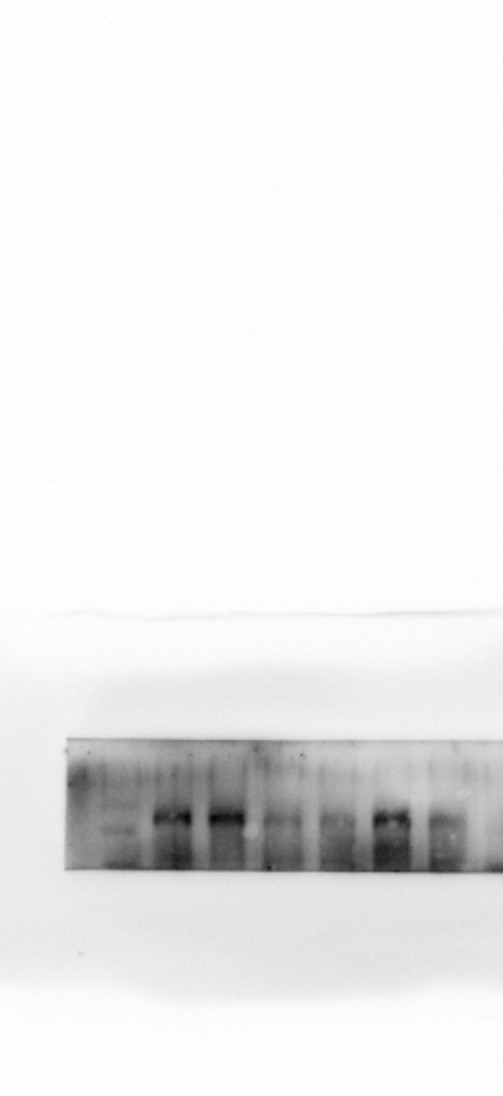

Supplement: Figure 2—source data 2. [file elife-104060-fig2-data2.zip › Figure 2-source data 2/2F/48h/p-FGFR/p-fgfr=.jpg]

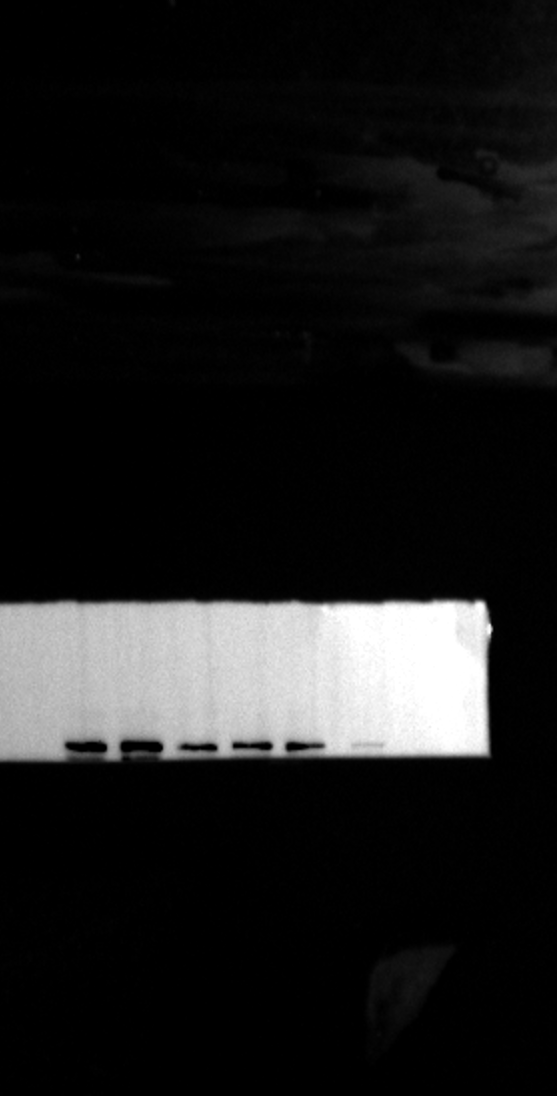

Supplement: Figure 2—source data 2. [file elife-104060-fig2-data2.zip › Figure 2-source data 2/2F/48h/p-mTOR/1 PMTOR MERGE.Tif]

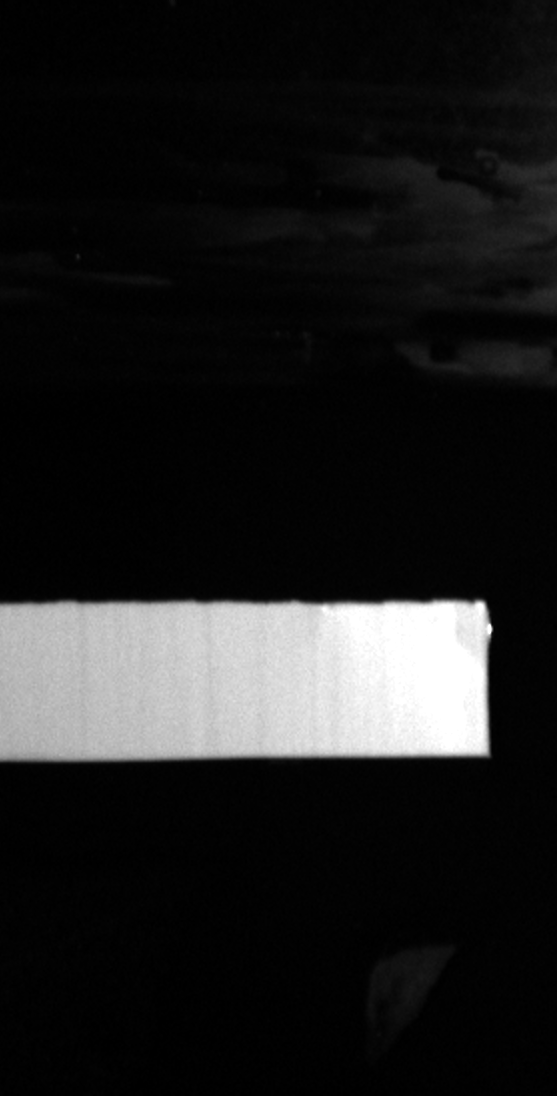

Supplement: Figure 2—source data 2. [file elife-104060-fig2-data2.zip › Figure 2-source data 2/2F/48h/p-mTOR/1 PMTOR WHITE.Tif]

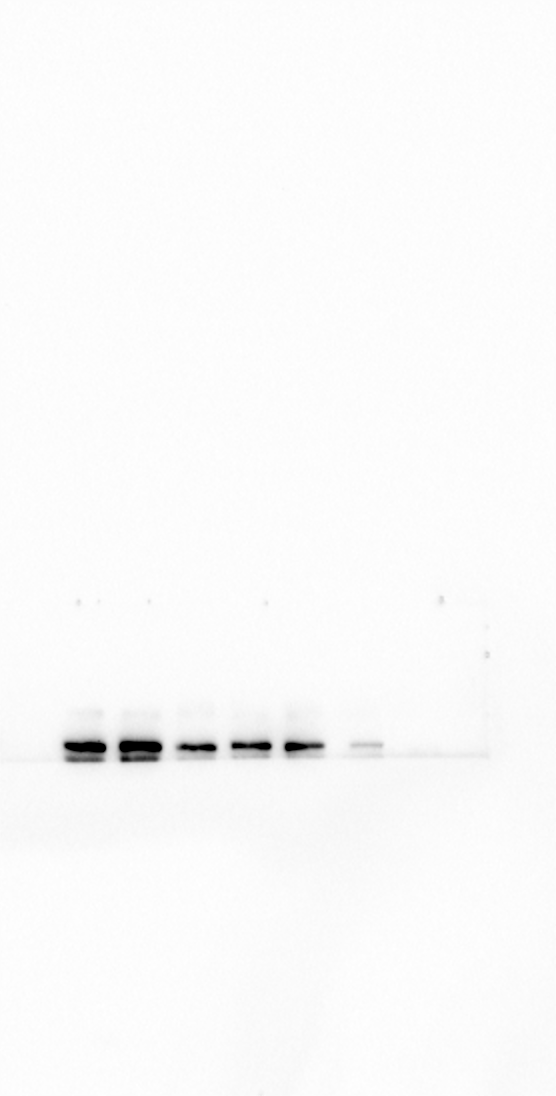

Supplement: Figure 2—source data 2. [file elife-104060-fig2-data2.zip › Figure 2-source data 2/2F/48h/p-mTOR/1 PMTOR.png]

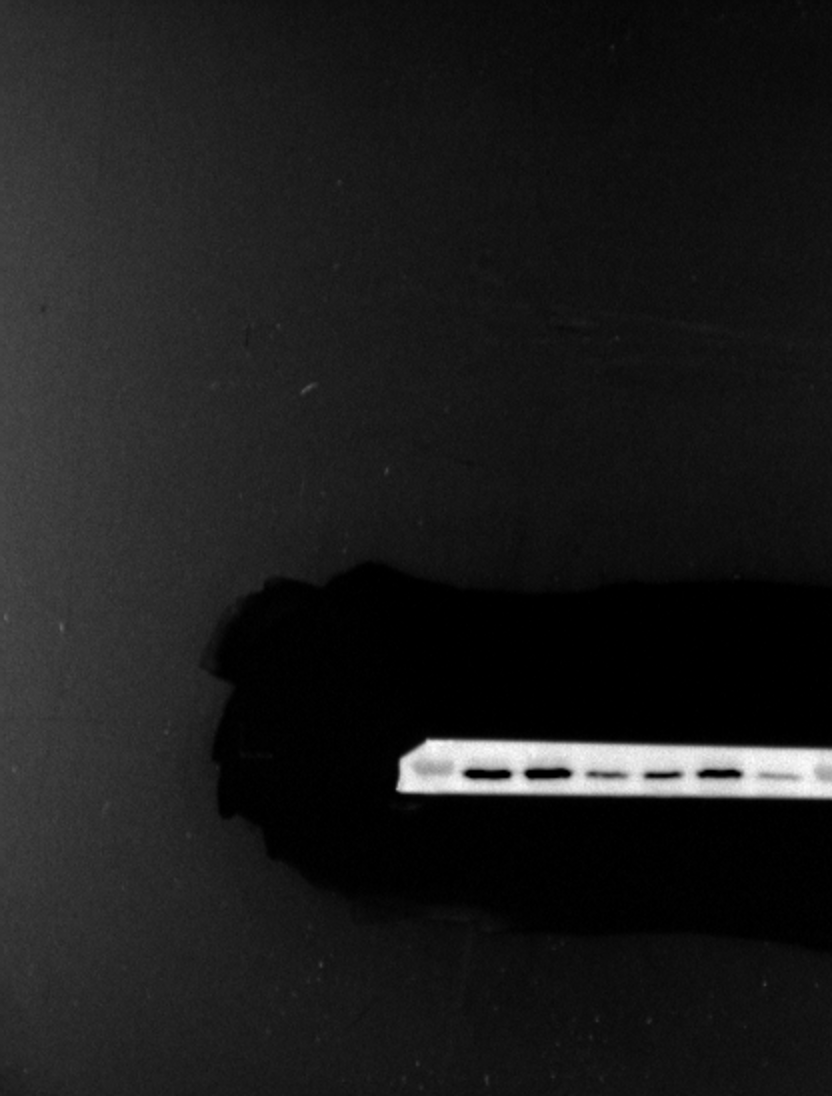

Supplement: Figure 2—source data 2. [file elife-104060-fig2-data2.zip › Figure 2-source data 2/2F/48h/p-p38/p-p38 merge==.Tif]

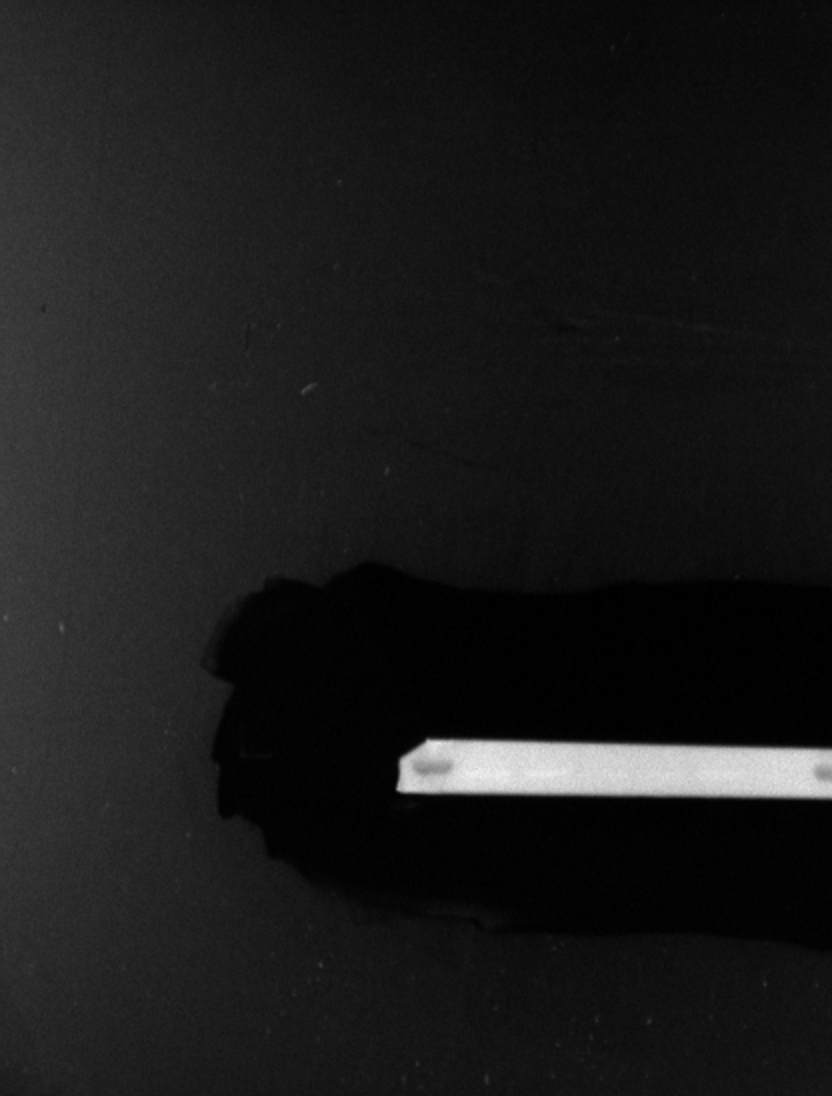

Supplement: Figure 2—source data 2. [file elife-104060-fig2-data2.zip › Figure 2-source data 2/2F/48h/p-p38/p-p38 white=.Tif]

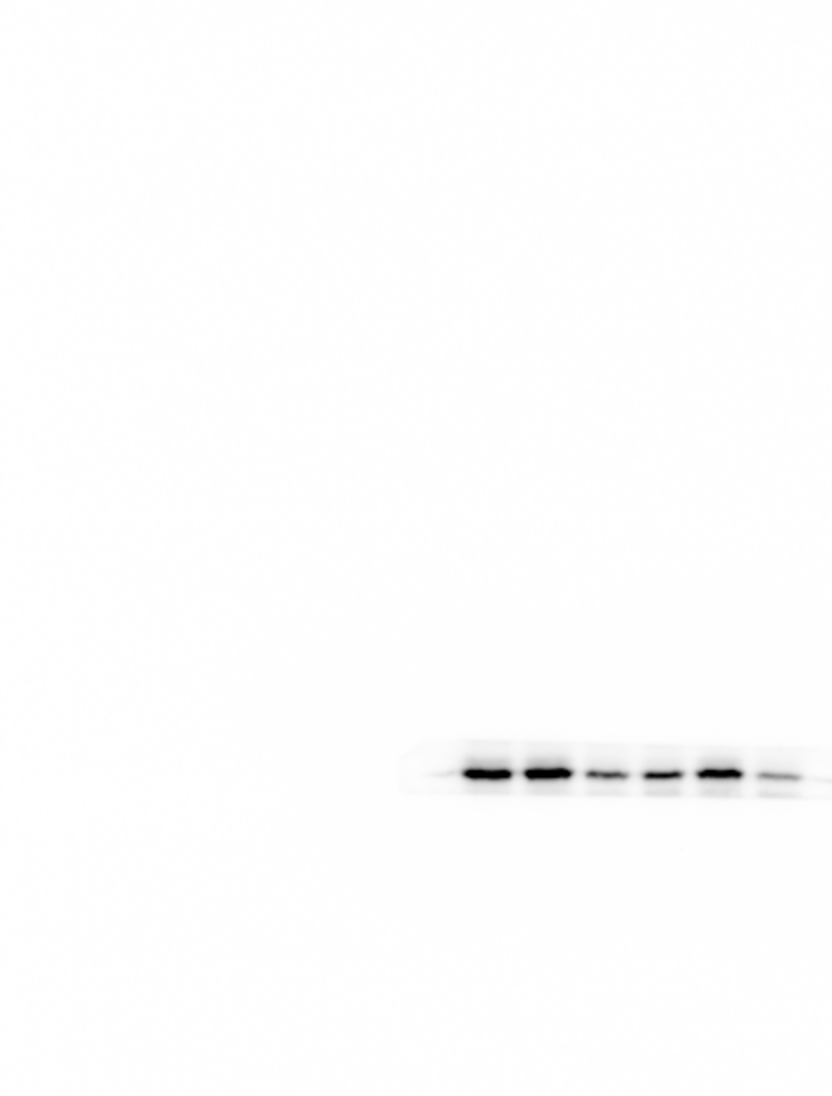

Supplement: Figure 2—source data 2. [file elife-104060-fig2-data2.zip › Figure 2-source data 2/2F/48h/p-p38/p-p38=.Tif]

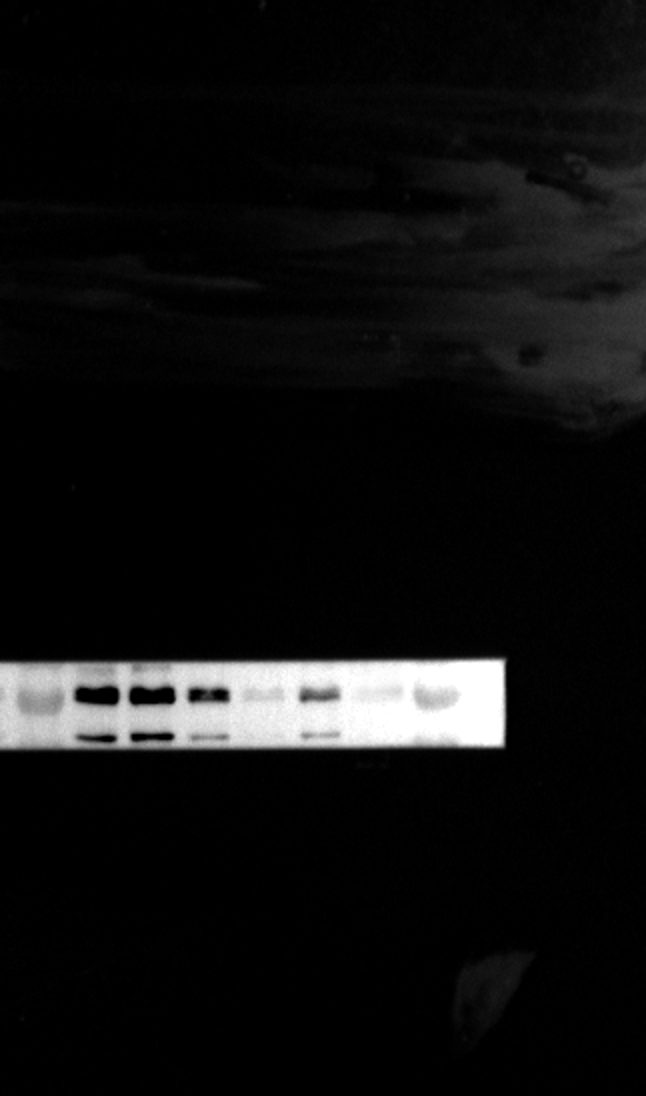

Supplement: Figure 2—source data 2. [file elife-104060-fig2-data2.zip › Figure 2-source data 2/2F/48h/p-SHP2/1 PSHP2 MERGE.Tif]

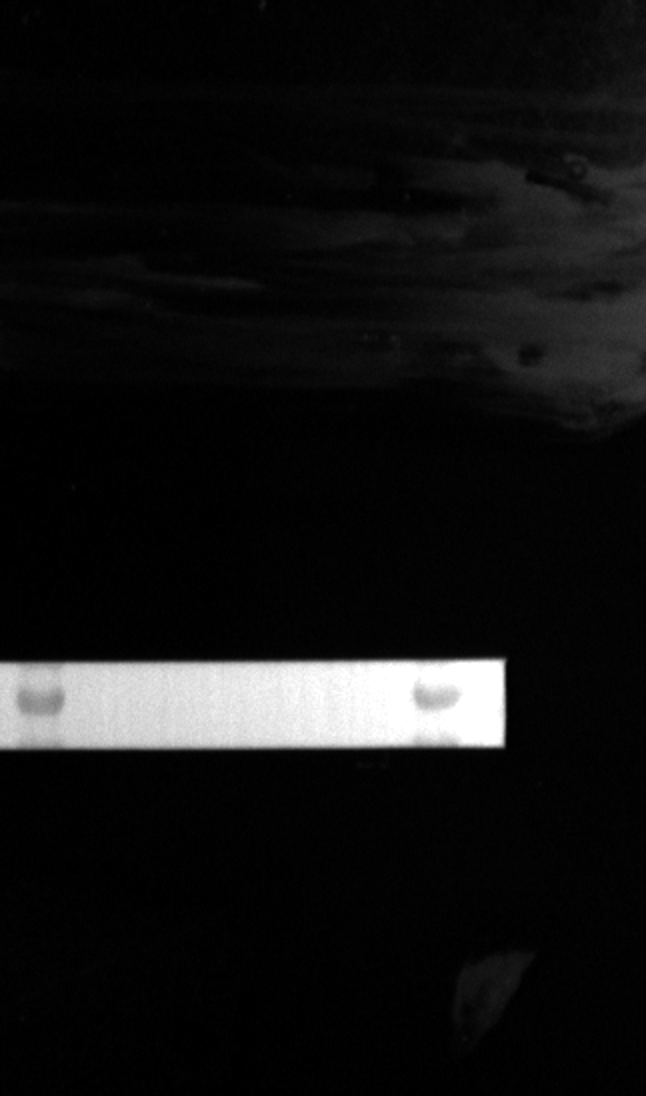

Supplement: Figure 2—source data 2. [file elife-104060-fig2-data2.zip › Figure 2-source data 2/2F/48h/p-SHP2/1 PSHP2 WHITE.Tif]

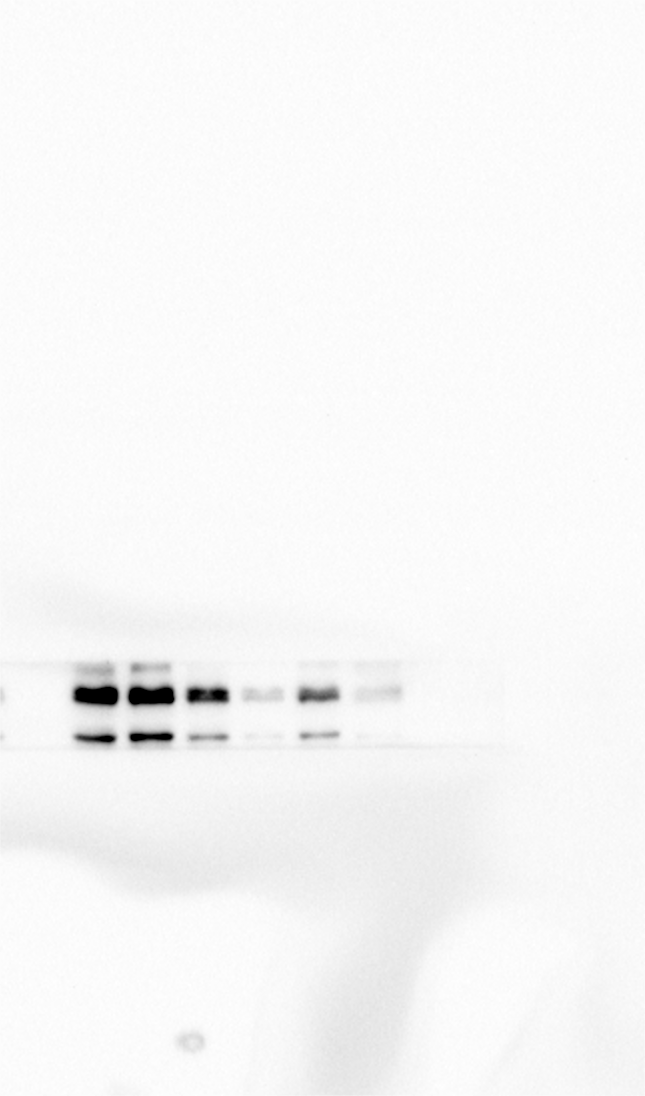

Supplement: Figure 2—source data 2. [file elife-104060-fig2-data2.zip › Figure 2-source data 2/2F/48h/p-SHP2/1 PSHP2.png]

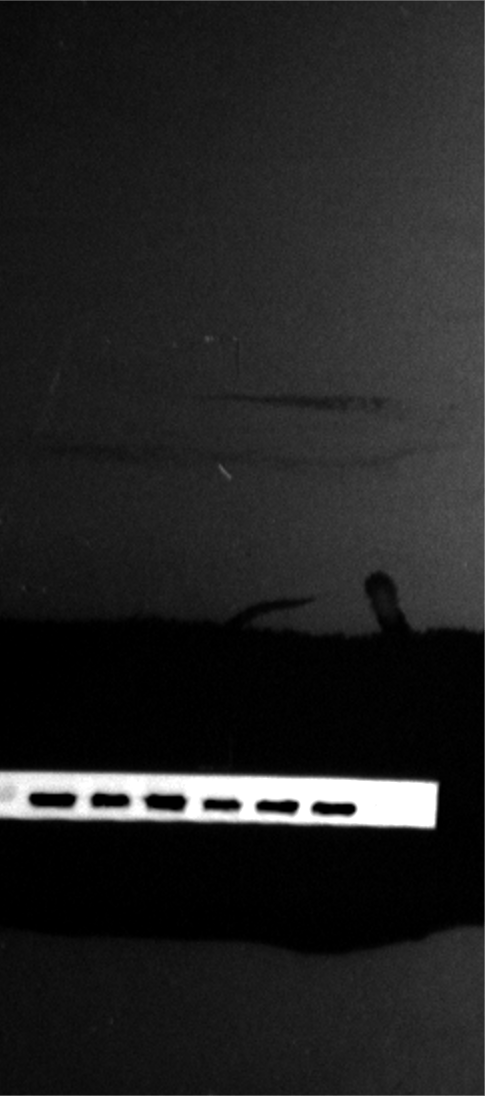

Supplement: Figure 2—source data 2. [file elife-104060-fig2-data2.zip › Figure 2-source data 2/2F/48h/p38/p38 merge=.png]

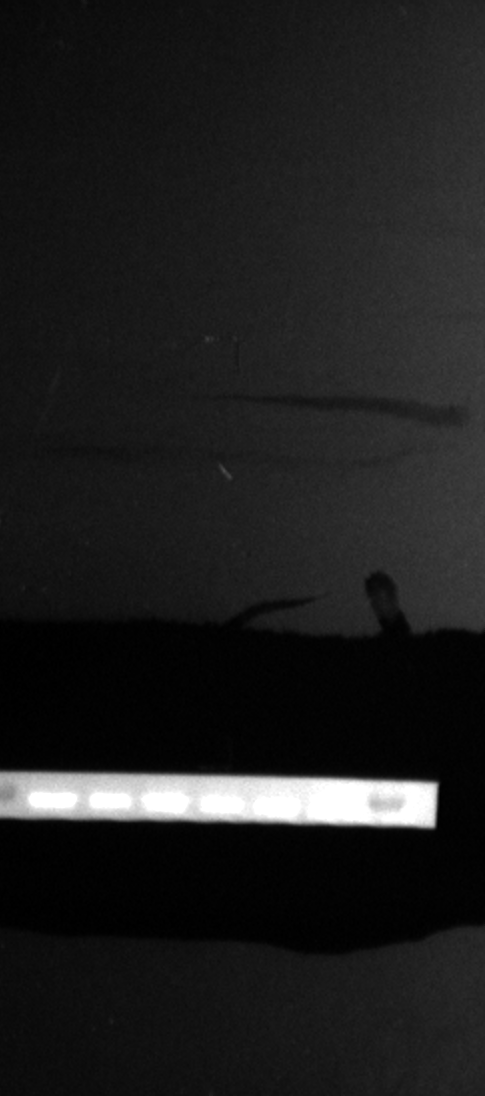

Supplement: Figure 2—source data 2. [file elife-104060-fig2-data2.zip › Figure 2-source data 2/2F/48h/p38/p38 white=.Tif]

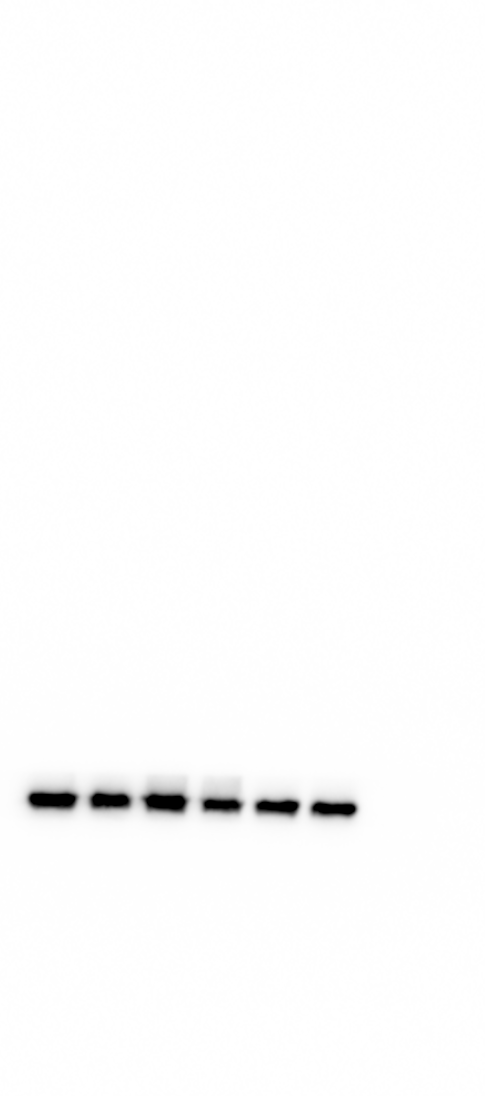

Supplement: Figure 2—source data 2. [file elife-104060-fig2-data2.zip › Figure 2-source data 2/2F/48h/p38/p38=.Tif]

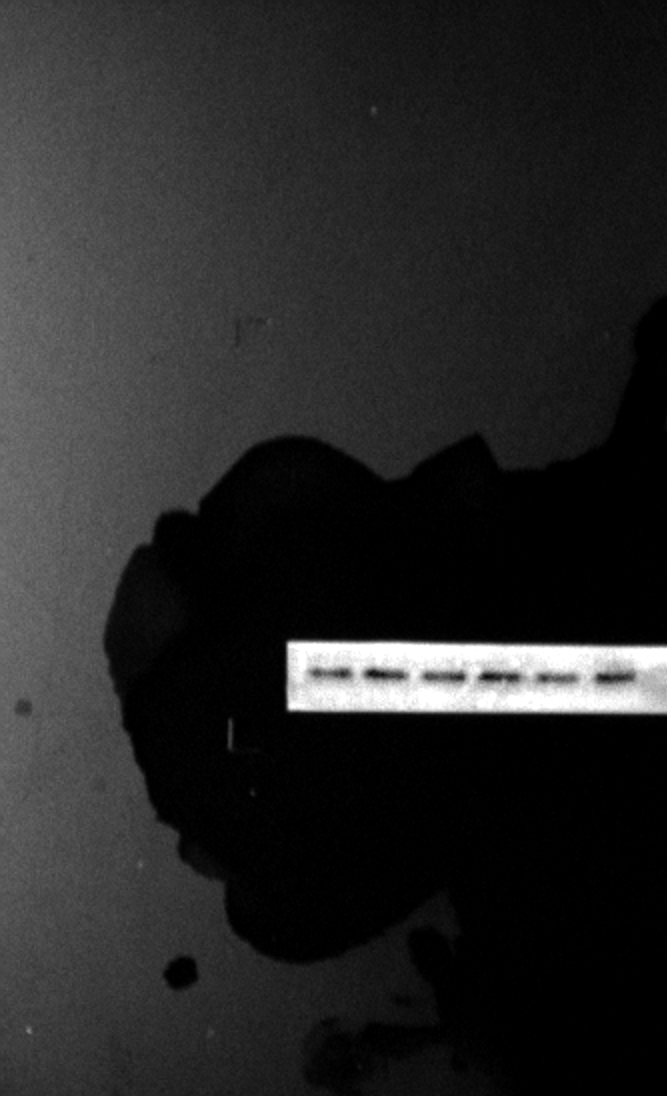

Supplement: Figure 2—source data 2. [file elife-104060-fig2-data2.zip › Figure 2-source data 2/2F/48h/SHP2/SHP2 merge=.Tif]

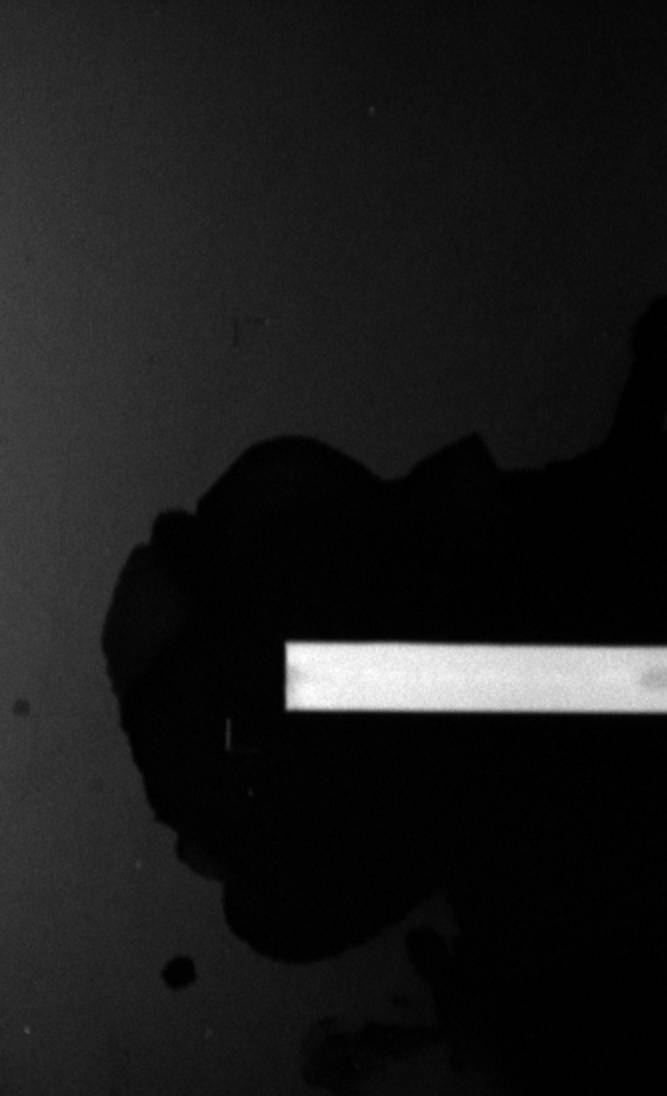

Supplement: Figure 2—source data 2. [file elife-104060-fig2-data2.zip › Figure 2-source data 2/2F/48h/SHP2/SHP2 white=.Tif]

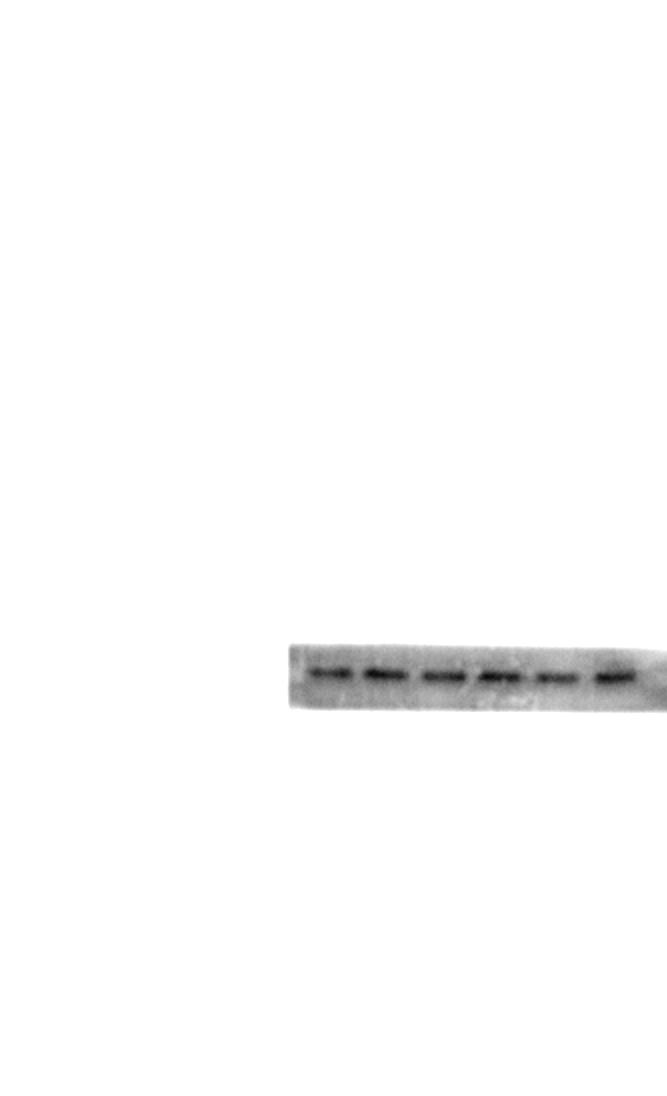

Supplement: Figure 2—source data 2. [file elife-104060-fig2-data2.zip › Figure 2-source data 2/2F/48h/SHP2/shp2=.Tif]

Figure 3

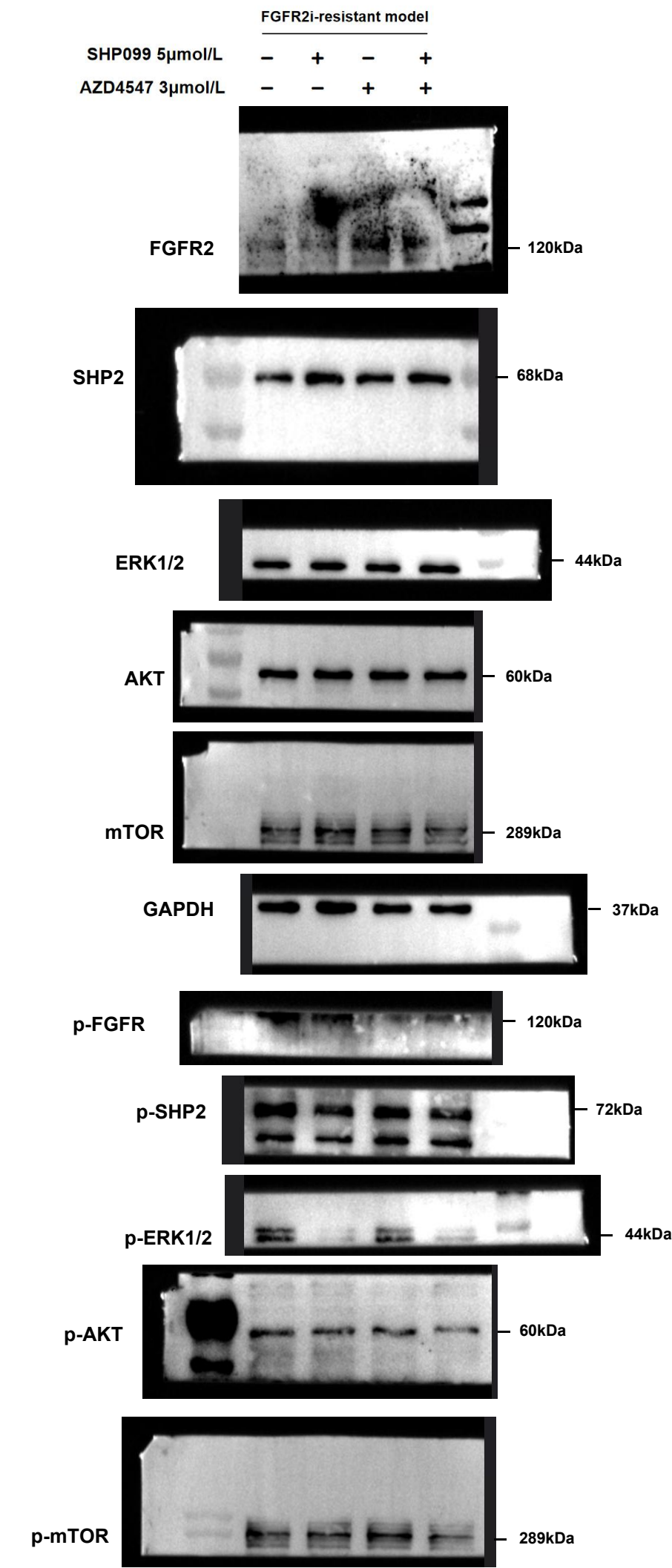

Supplement: Figure 3—source data 1. [file elife-104060-fig3-data1.pdf]

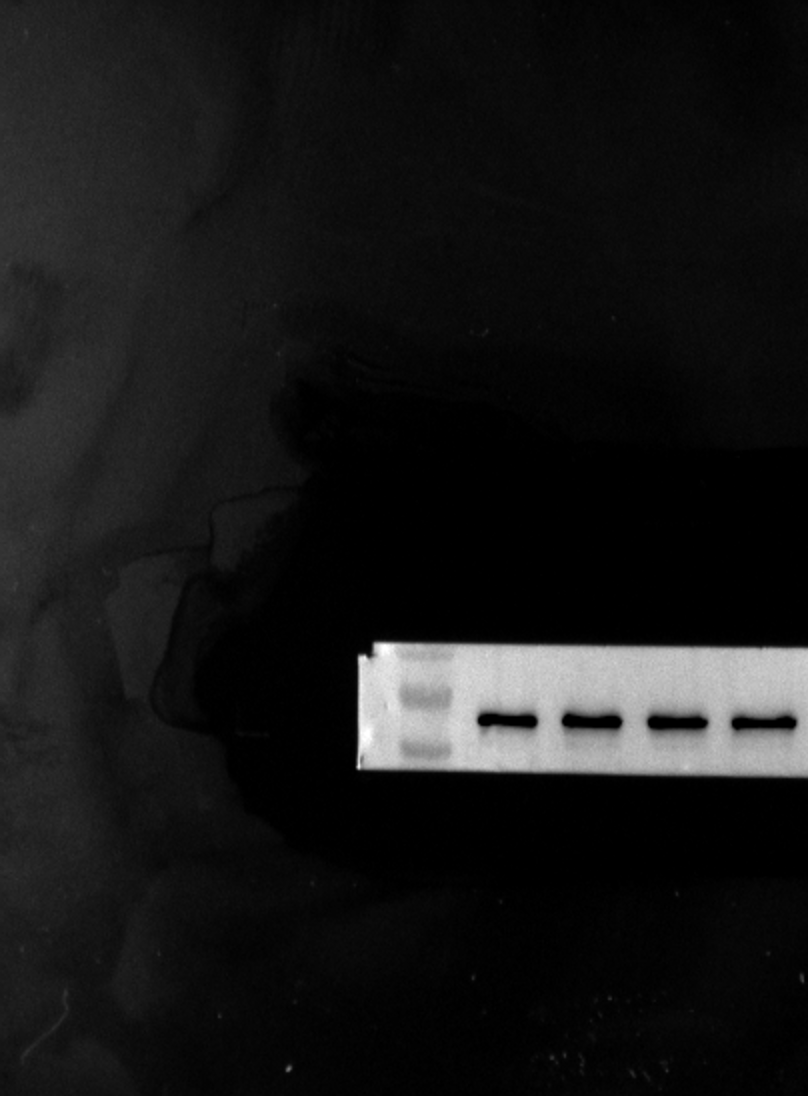

Supplement: Figure 3—source data 2. [file elife-104060-fig3-data2.zip › Figure 3-source data2/AKT/akt merge.Tif]

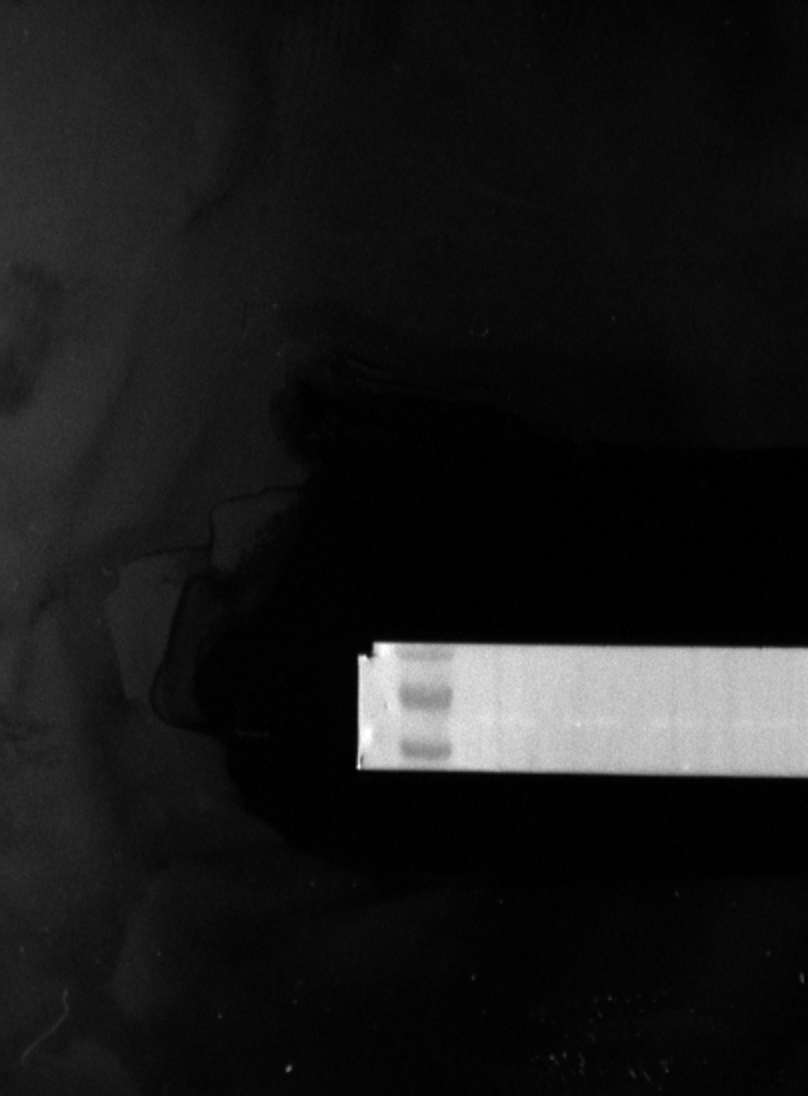

Supplement: Figure 3—source data 2. [file elife-104060-fig3-data2.zip › Figure 3-source data2/AKT/akt white.Tif]

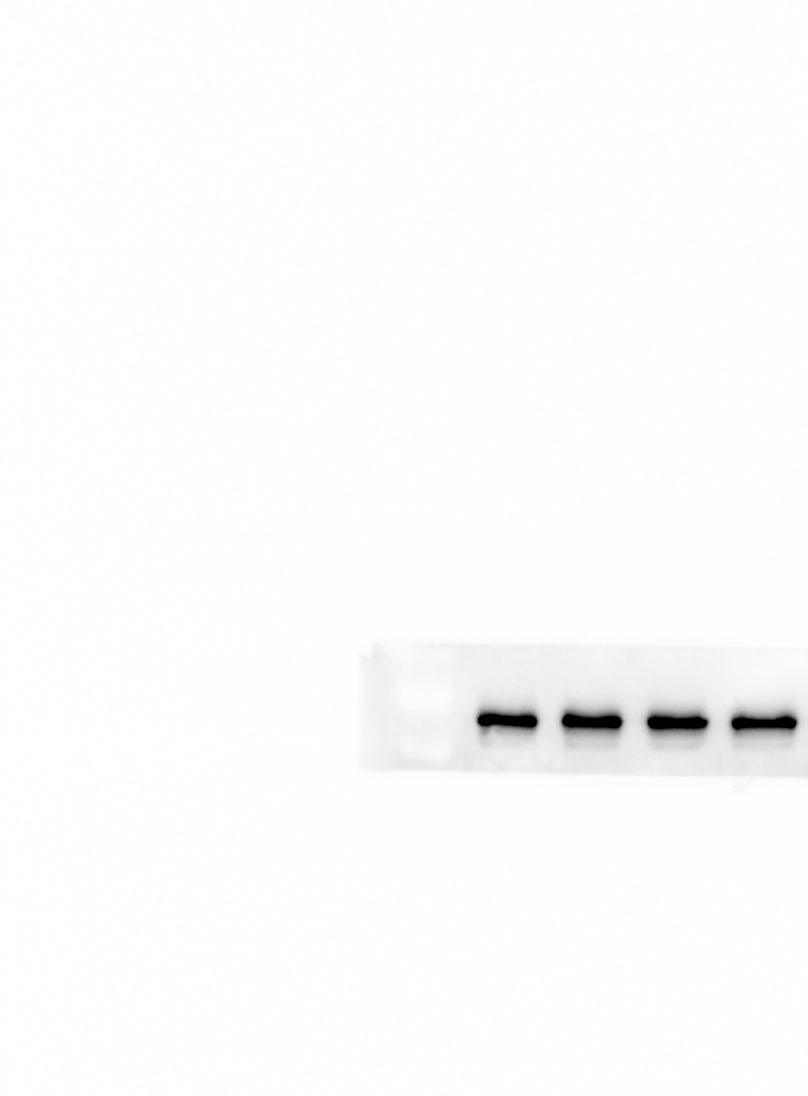

Supplement: Figure 3—source data 2. [file elife-104060-fig3-data2.zip › Figure 3-source data2/AKT/akt.Tif]

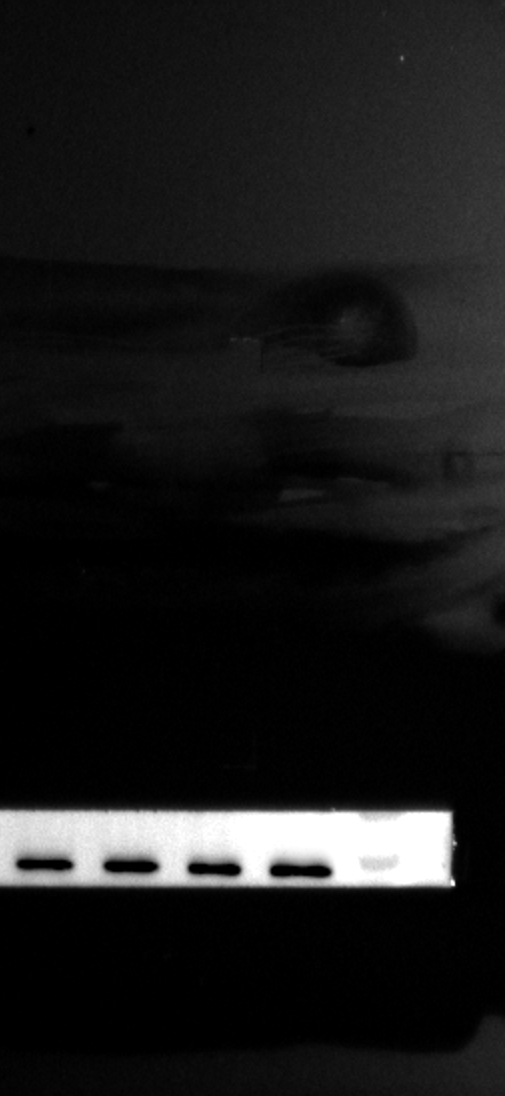

Supplement: Figure 3—source data 2. [file elife-104060-fig3-data2.zip › Figure 3-source data2/ERK/1 ERK MERGE.Tif]

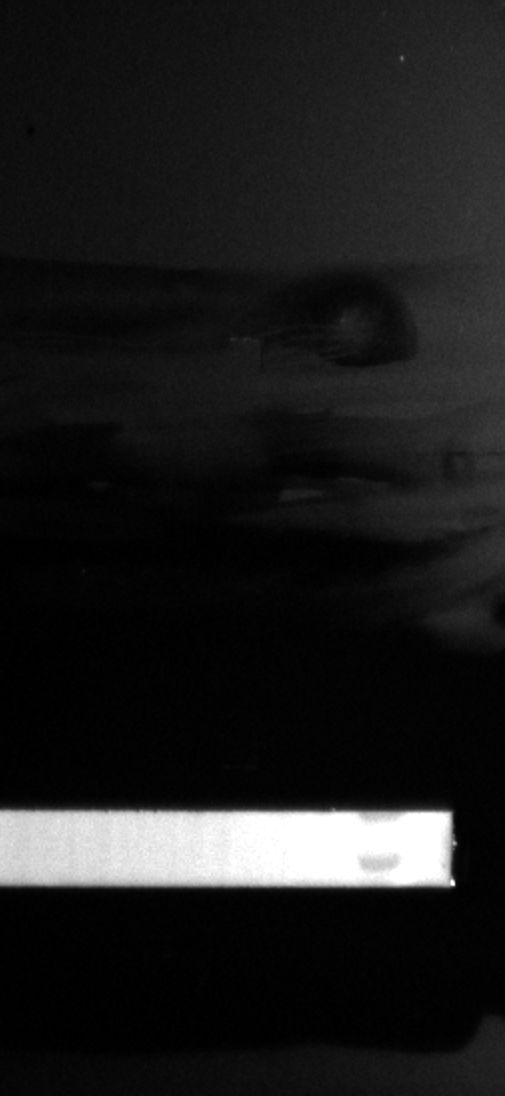

Supplement: Figure 3—source data 2. [file elife-104060-fig3-data2.zip › Figure 3-source data2/ERK/1 ERK WHITE.Tif]

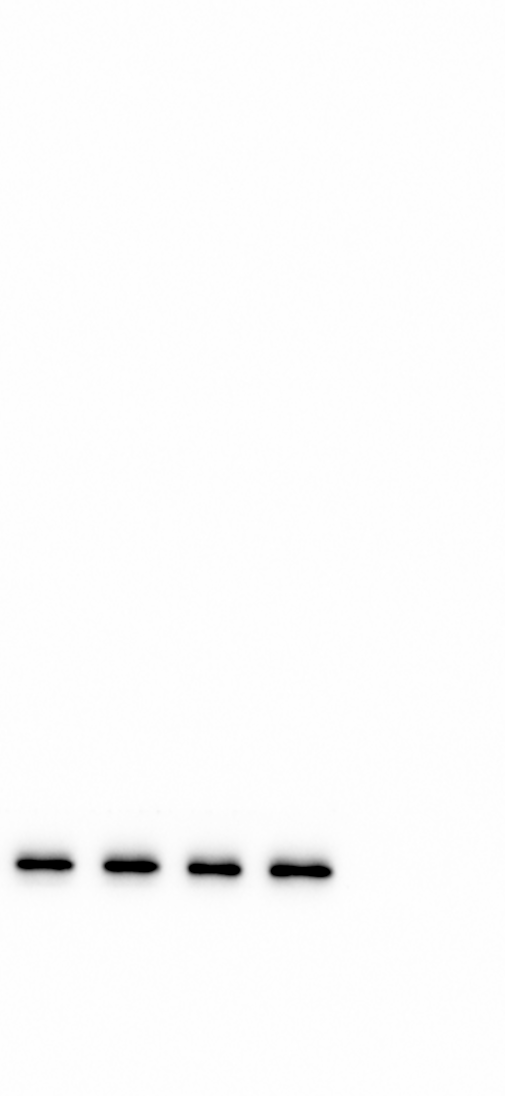

Supplement: Figure 3—source data 2. [file elife-104060-fig3-data2.zip › Figure 3-source data2/ERK/1 ERK.Tif]

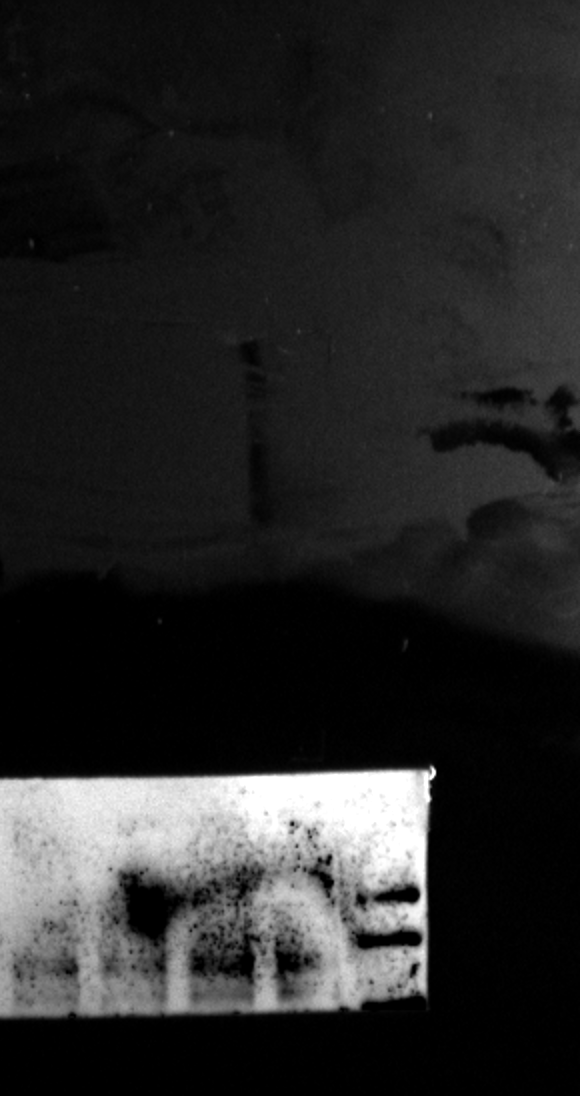

Supplement: Figure 3—source data 2. [file elife-104060-fig3-data2.zip › Figure 3-source data2/FGFR2/fgfr2 merge==.Tif]

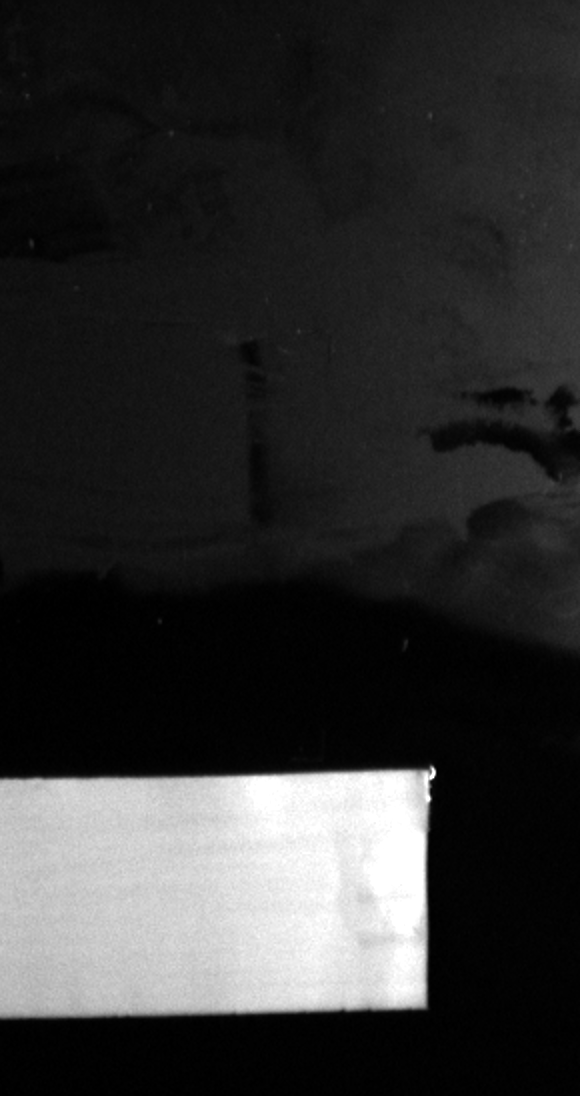

Supplement: Figure 3—source data 2. [file elife-104060-fig3-data2.zip › Figure 3-source data2/FGFR2/fgfr2 white=.Tif]

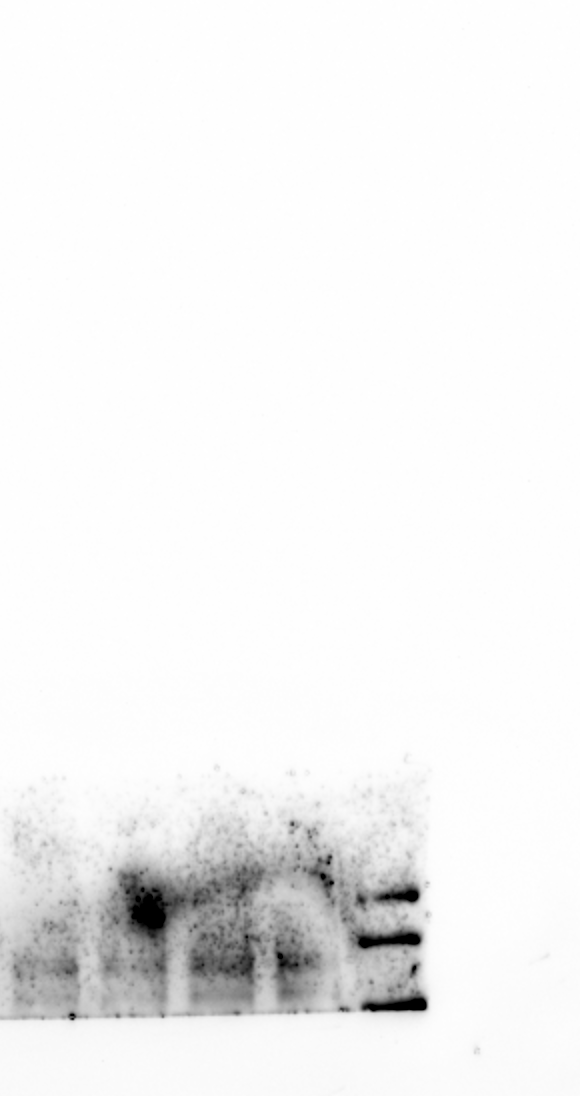

Supplement: Figure 3—source data 2. [file elife-104060-fig3-data2.zip › Figure 3-source data2/FGFR2/fgfr2==.Tif]

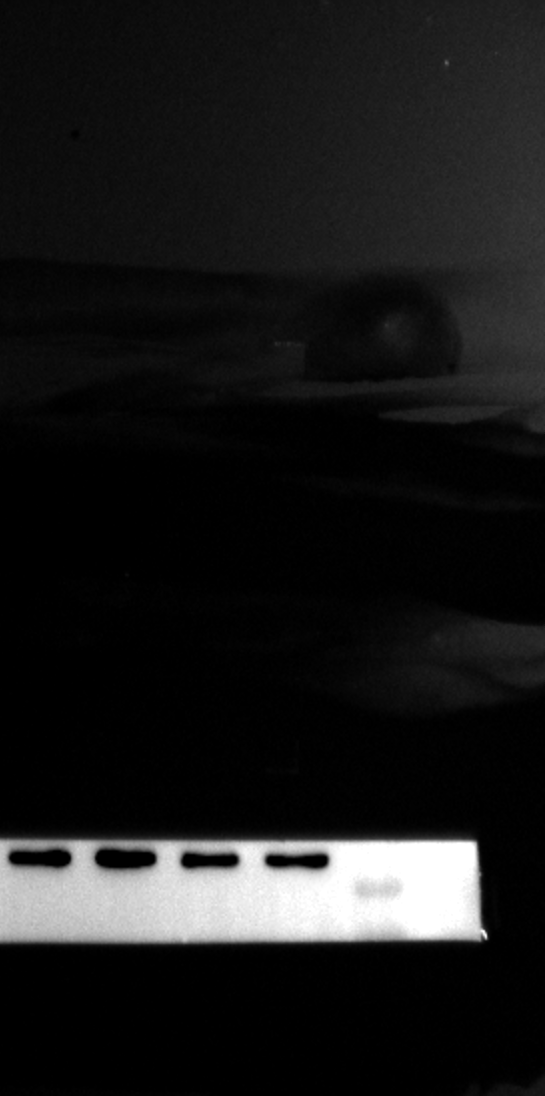

Supplement: Figure 3—source data 2. [file elife-104060-fig3-data2.zip › Figure 3-source data2/GAPDH/GAP MERGE.Tif]

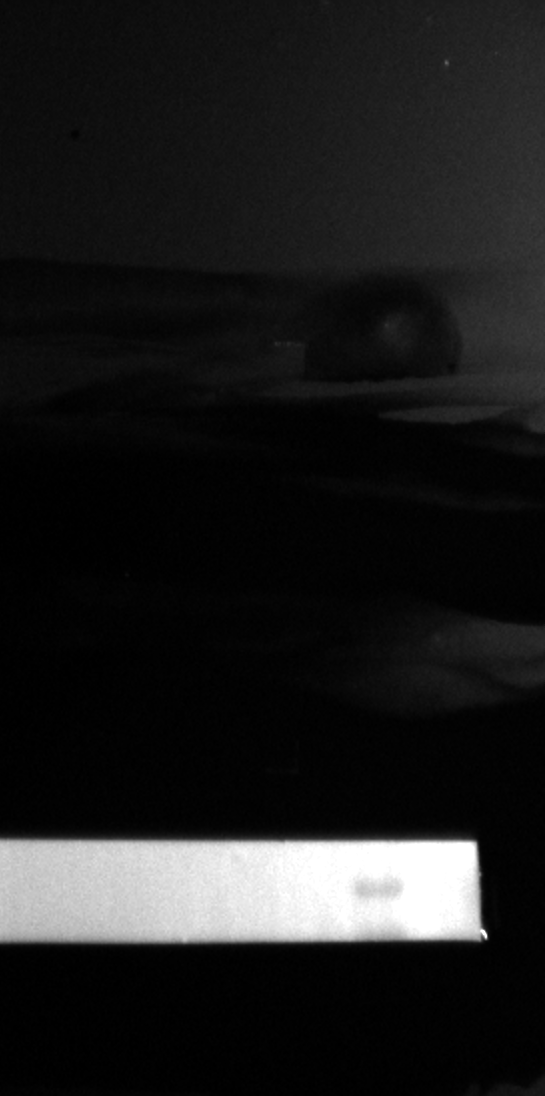

Supplement: Figure 3—source data 2. [file elife-104060-fig3-data2.zip › Figure 3-source data2/GAPDH/GAP WHITE.Tif]

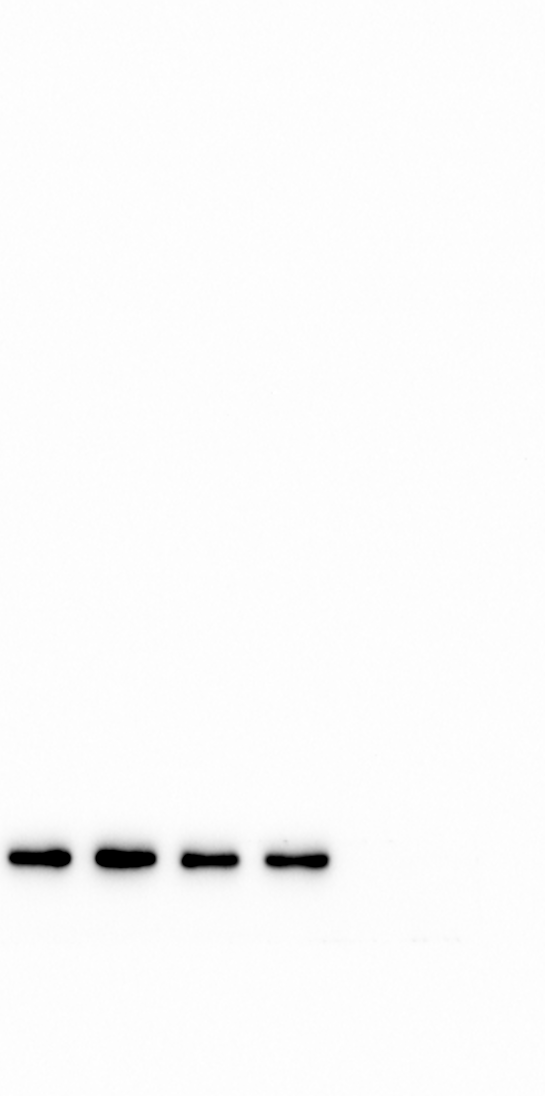

Supplement: Figure 3—source data 2. [file elife-104060-fig3-data2.zip › Figure 3-source data2/GAPDH/GAP.Tif]

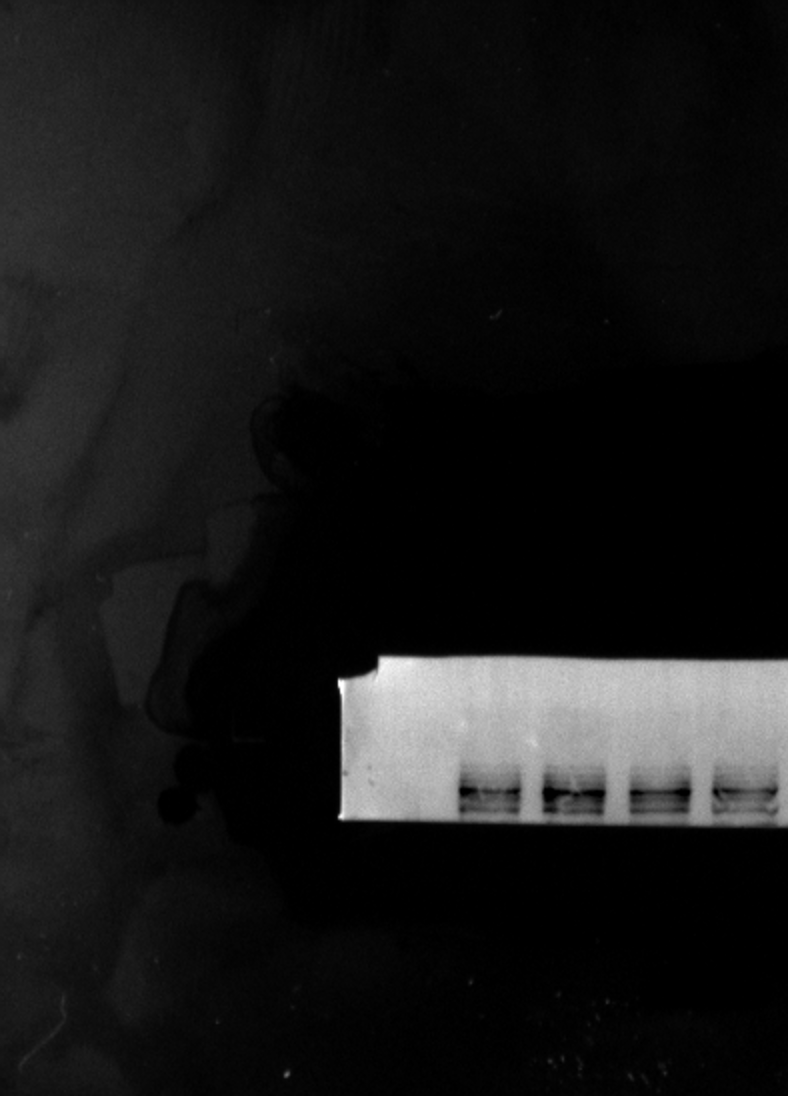

Supplement: Figure 3—source data 2. [file elife-104060-fig3-data2.zip › Figure 3-source data2/mTOR/mtor merge.Tif]

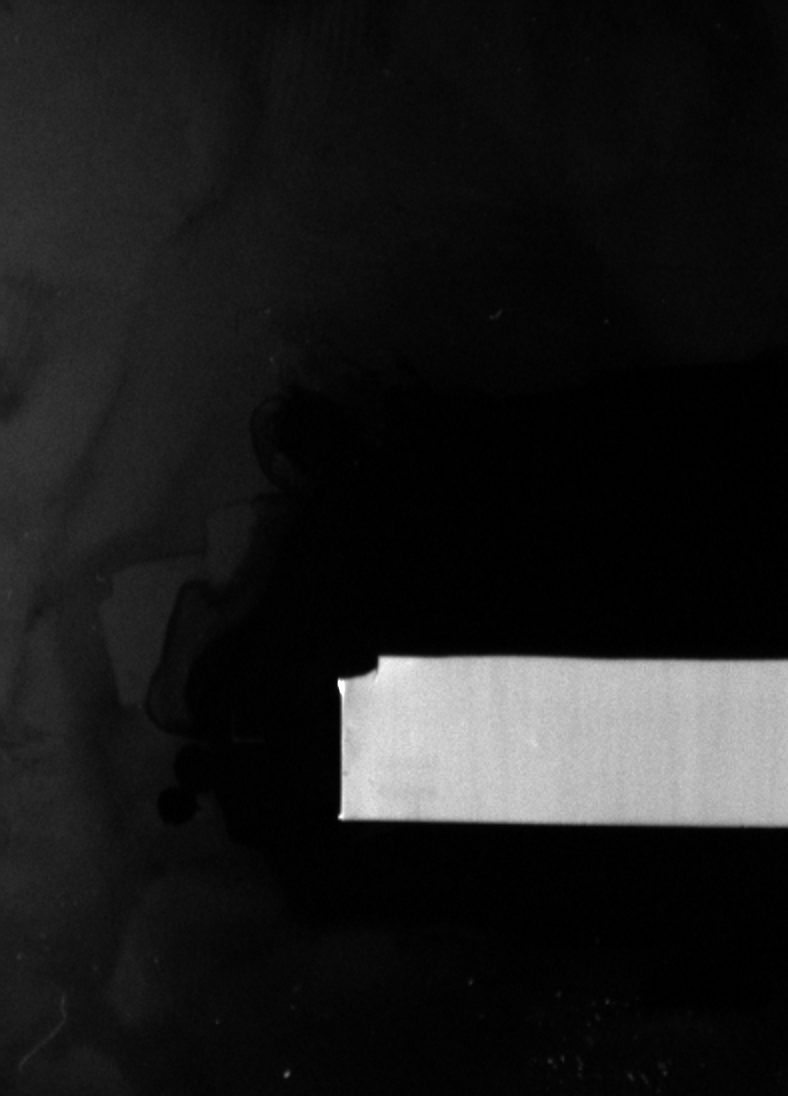

Supplement: Figure 3—source data 2. [file elife-104060-fig3-data2.zip › Figure 3-source data2/mTOR/mtor white.Tif]

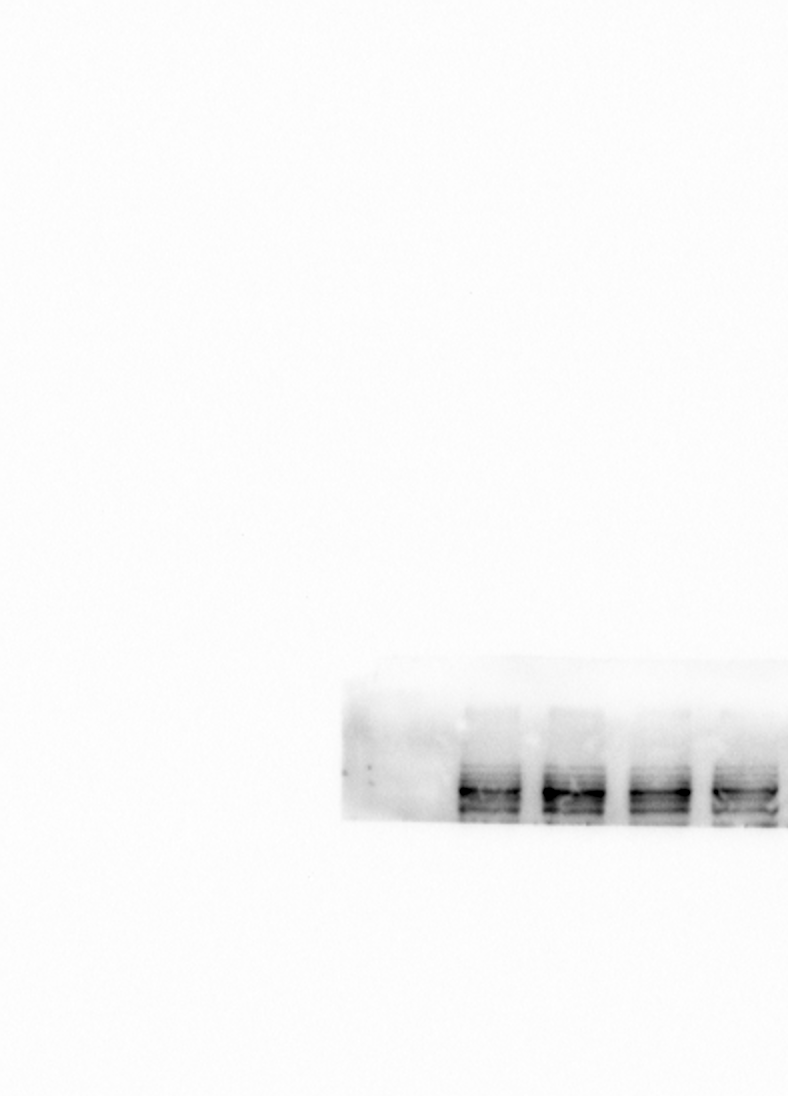

Supplement: Figure 3—source data 2. [file elife-104060-fig3-data2.zip › Figure 3-source data2/mTOR/mtor.Tif]

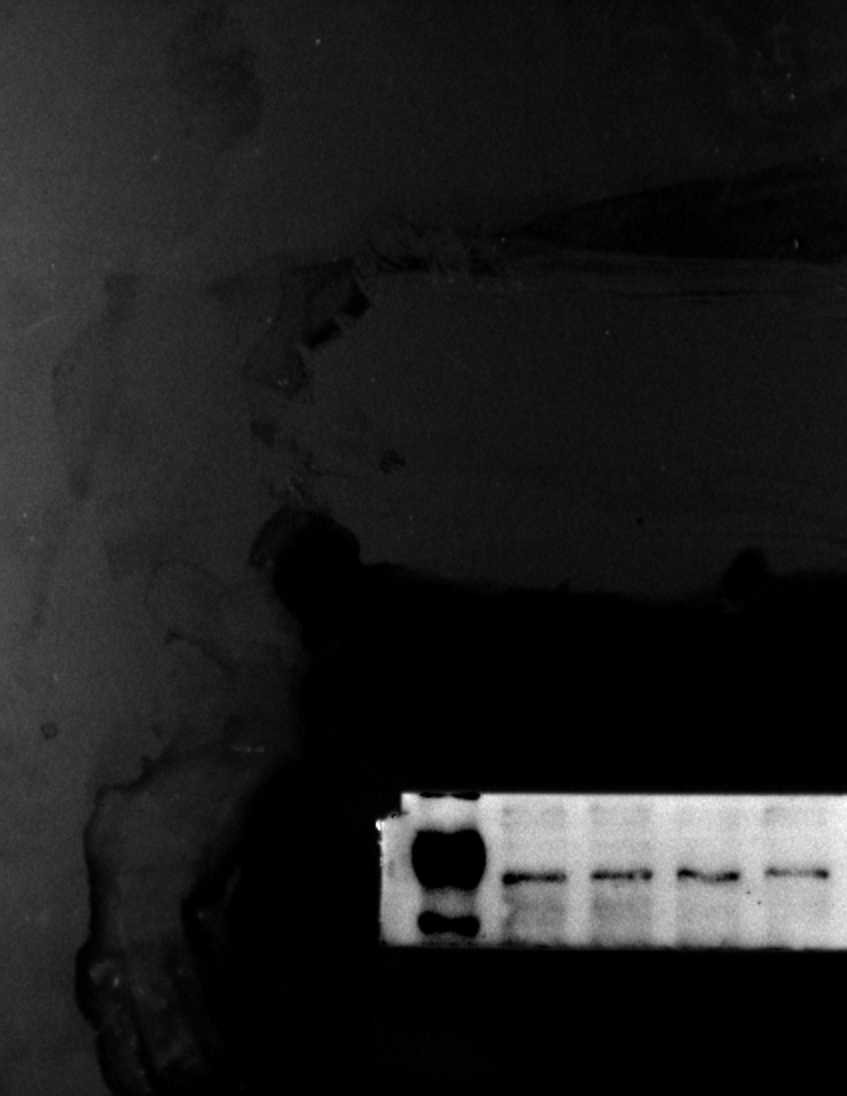

Supplement: Figure 3—source data 2. [file elife-104060-fig3-data2.zip › Figure 3-source data2/p-AKT/pakt merge.Tif]

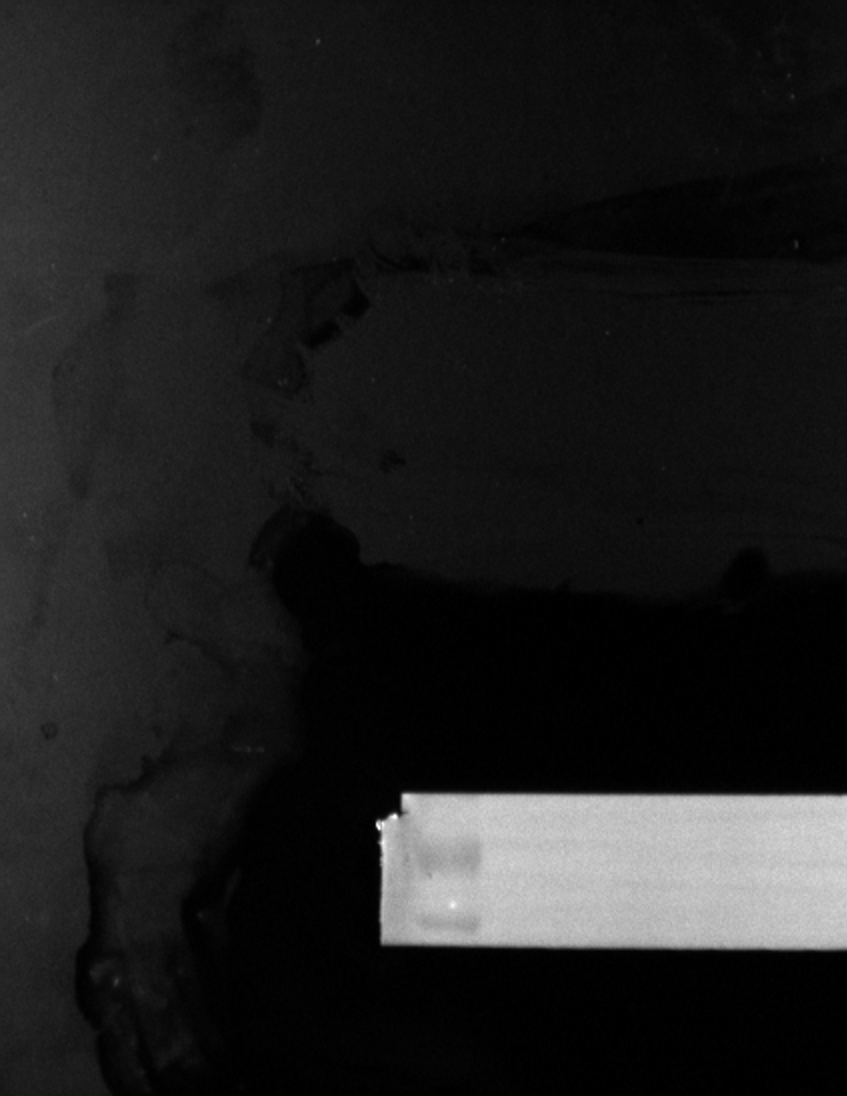

Supplement: Figure 3—source data 2. [file elife-104060-fig3-data2.zip › Figure 3-source data2/p-AKT/pakt white.Tif]

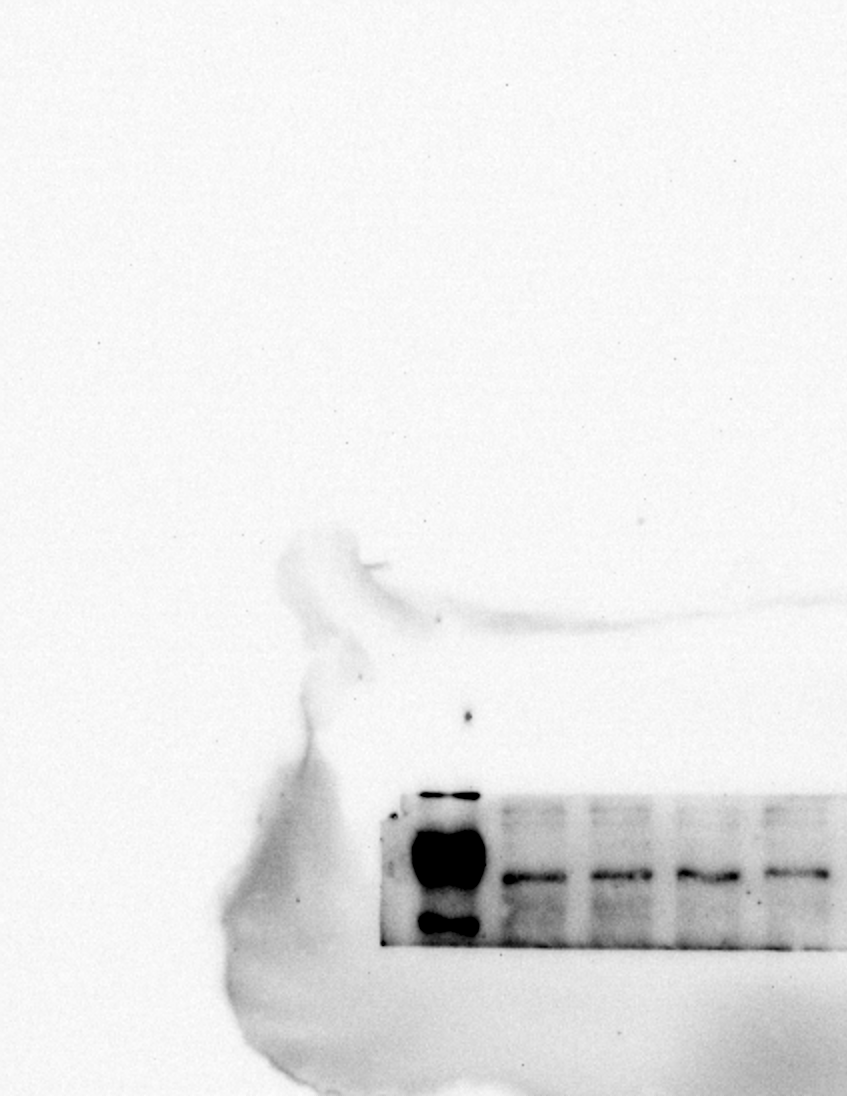

Supplement: Figure 3—source data 2. [file elife-104060-fig3-data2.zip › Figure 3-source data2/p-AKT/pakt.Tif]

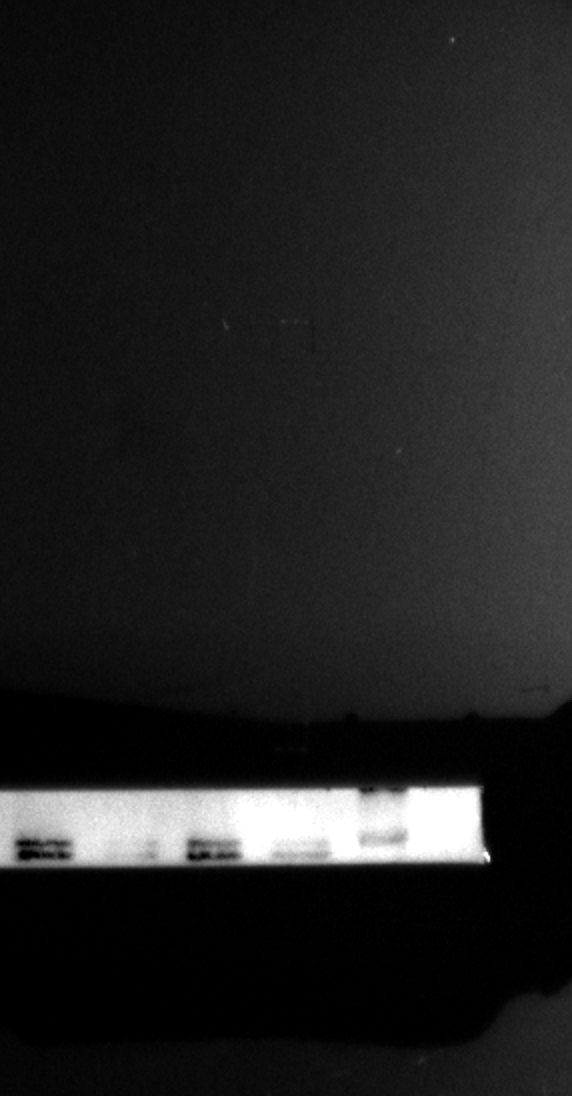

Supplement: Figure 3—source data 2. [file elife-104060-fig3-data2.zip › Figure 3-source data2/p-ERK/1 PERK MERGE.Tif]

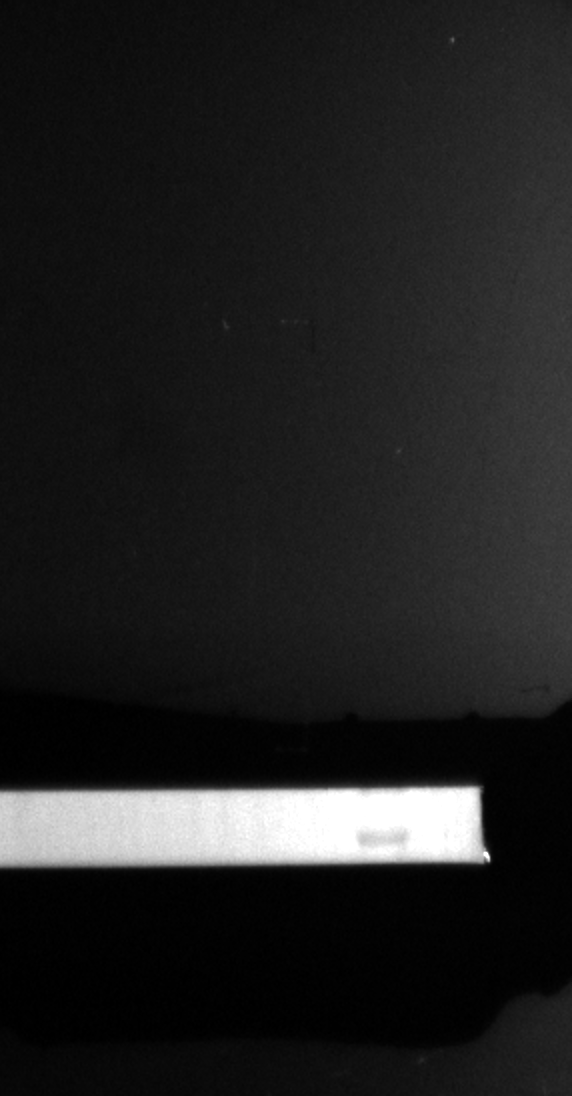

Supplement: Figure 3—source data 2. [file elife-104060-fig3-data2.zip › Figure 3-source data2/p-ERK/1 PERK WHITE.Tif]

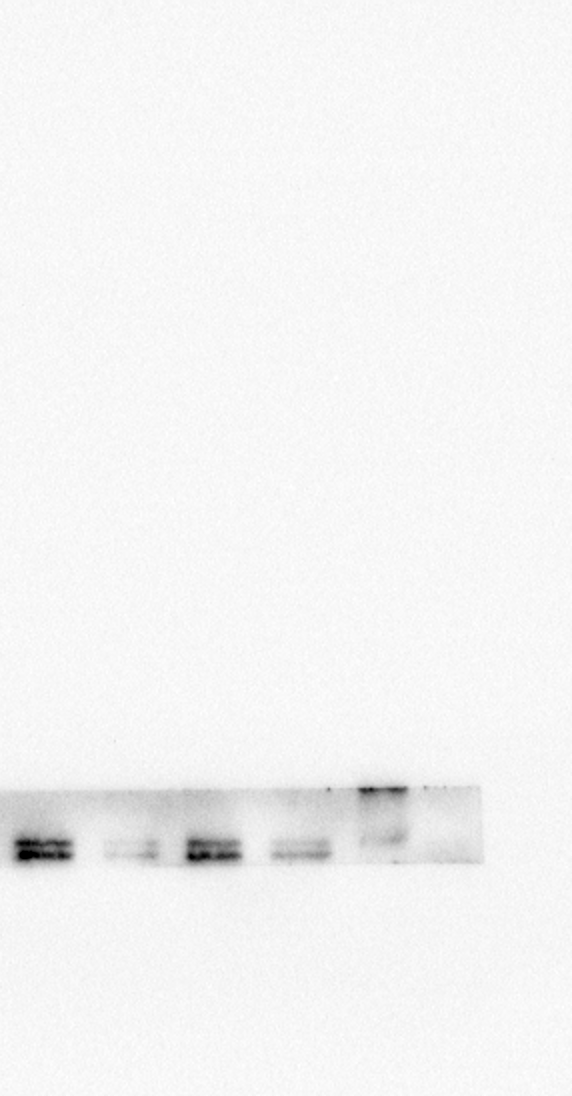

Supplement: Figure 3—source data 2. [file elife-104060-fig3-data2.zip › Figure 3-source data2/p-ERK/1 PERK.Tif]

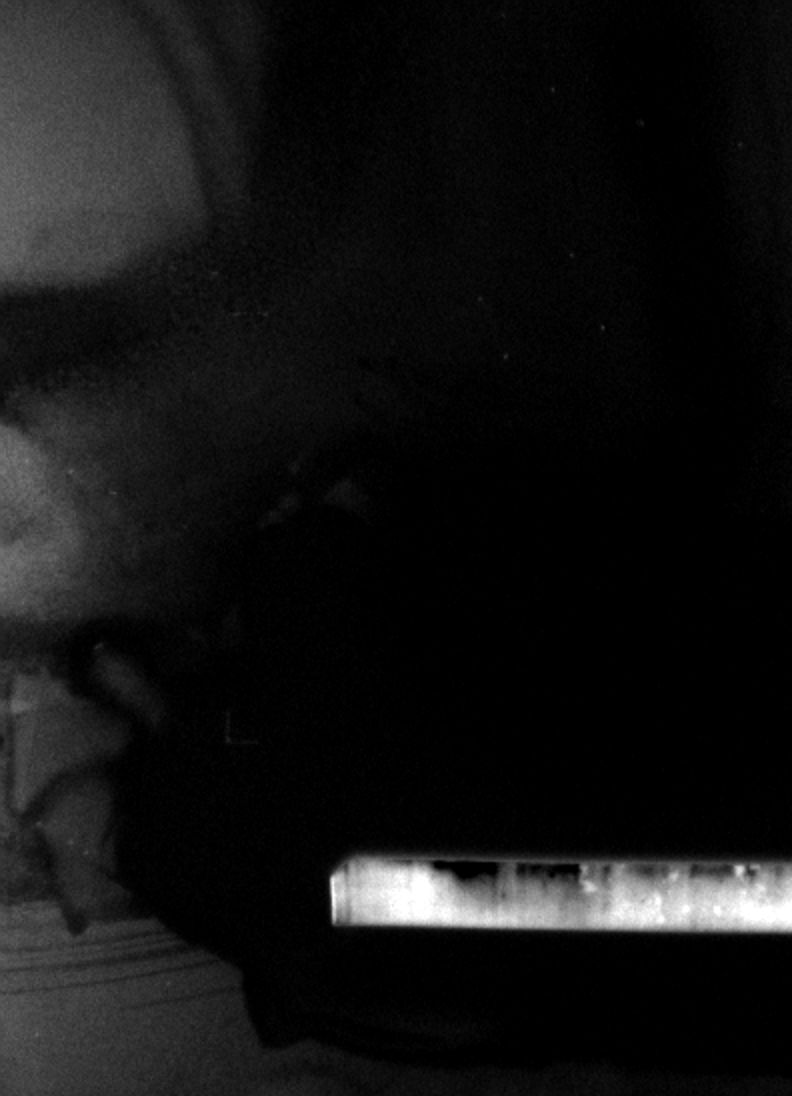

Supplement: Figure 3—source data 2. [file elife-104060-fig3-data2.zip › Figure 3-source data2/p-FGFR/PFGFR MERGE.Tif]

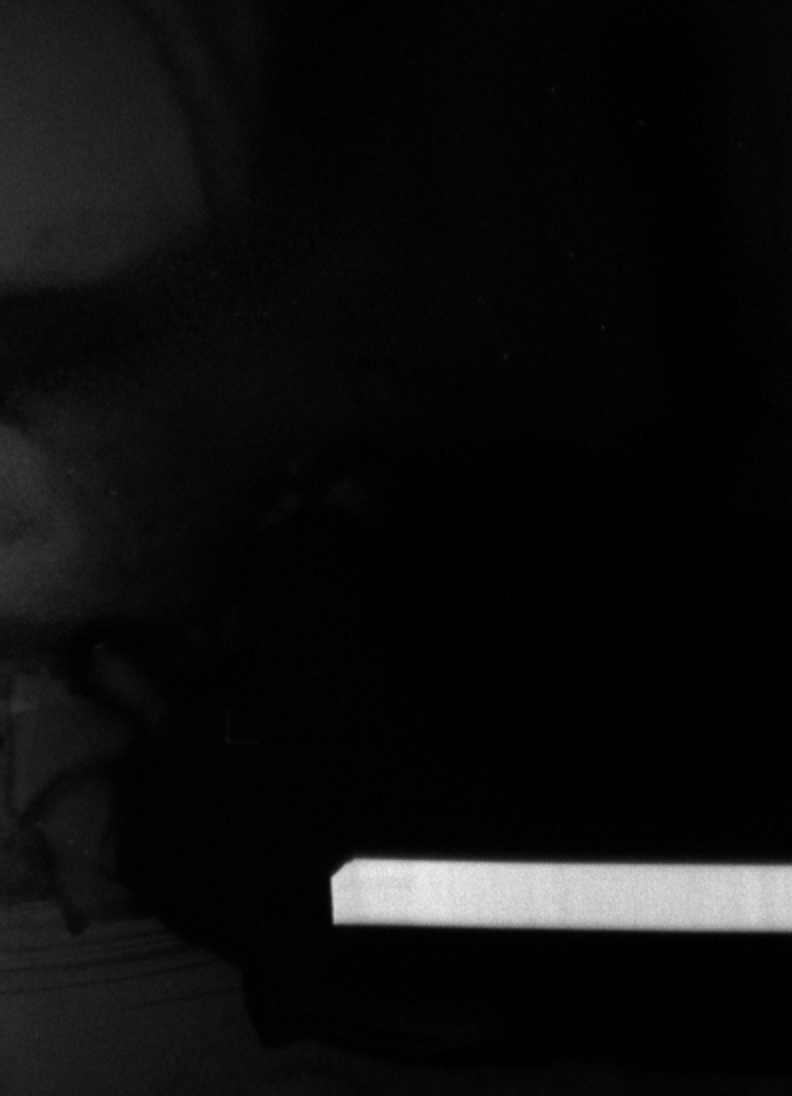

Supplement: Figure 3—source data 2. [file elife-104060-fig3-data2.zip › Figure 3-source data2/p-FGFR/PFGFR WHITE.Tif]

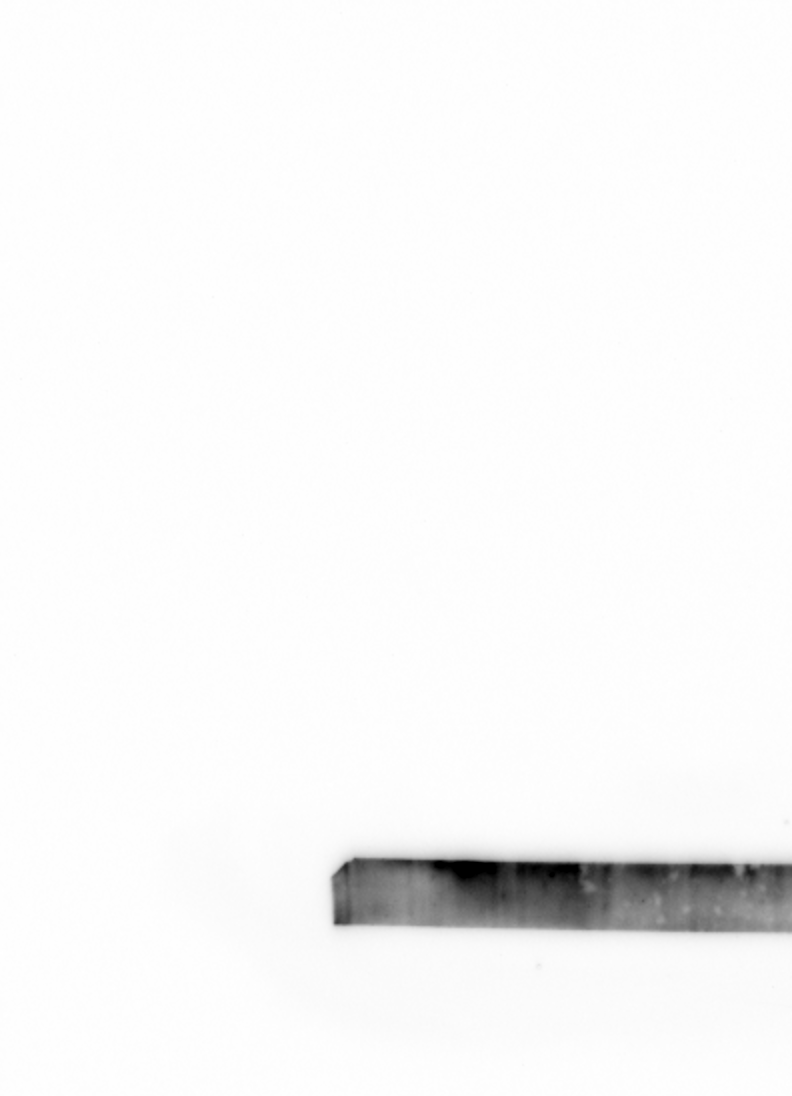

Supplement: Figure 3—source data 2. [file elife-104060-fig3-data2.zip › Figure 3-source data2/p-FGFR/PFGFR.Tif]

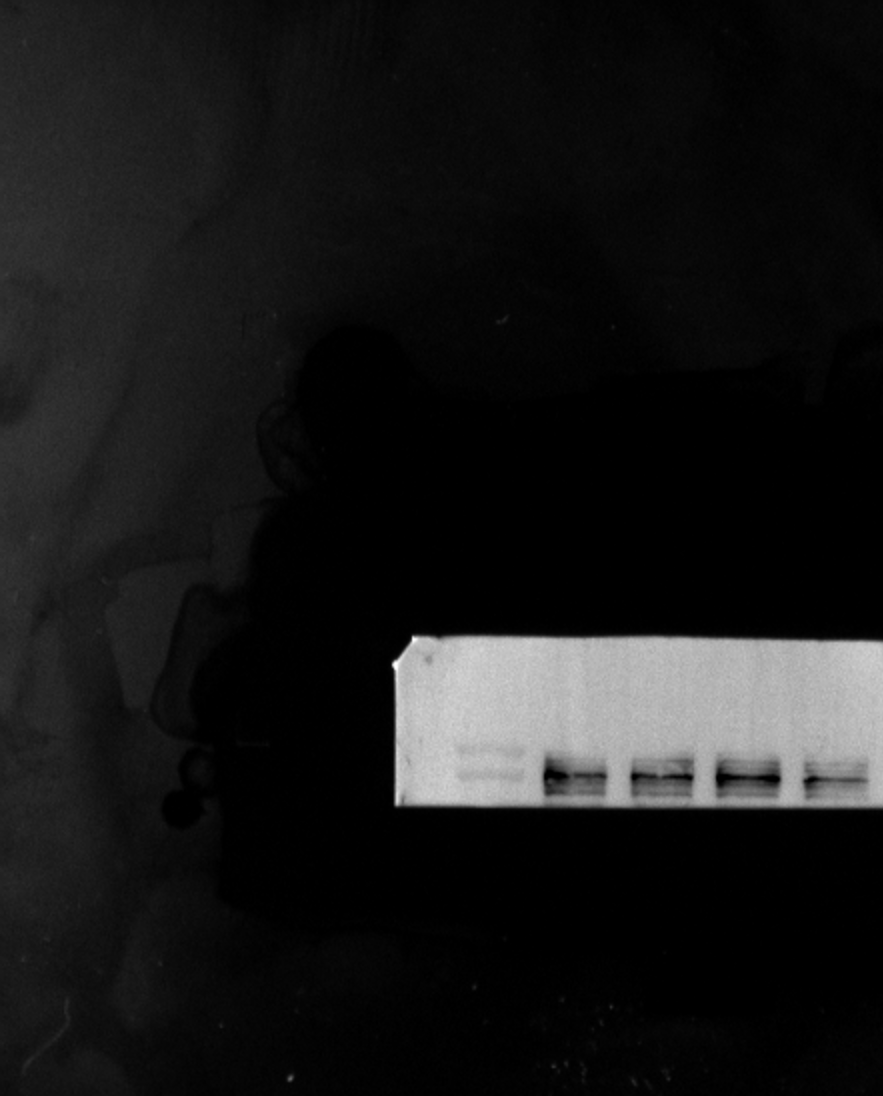

Supplement: Figure 3—source data 2. [file elife-104060-fig3-data2.zip › Figure 3-source data2/p-mTOR/pmtor merge.Tif]

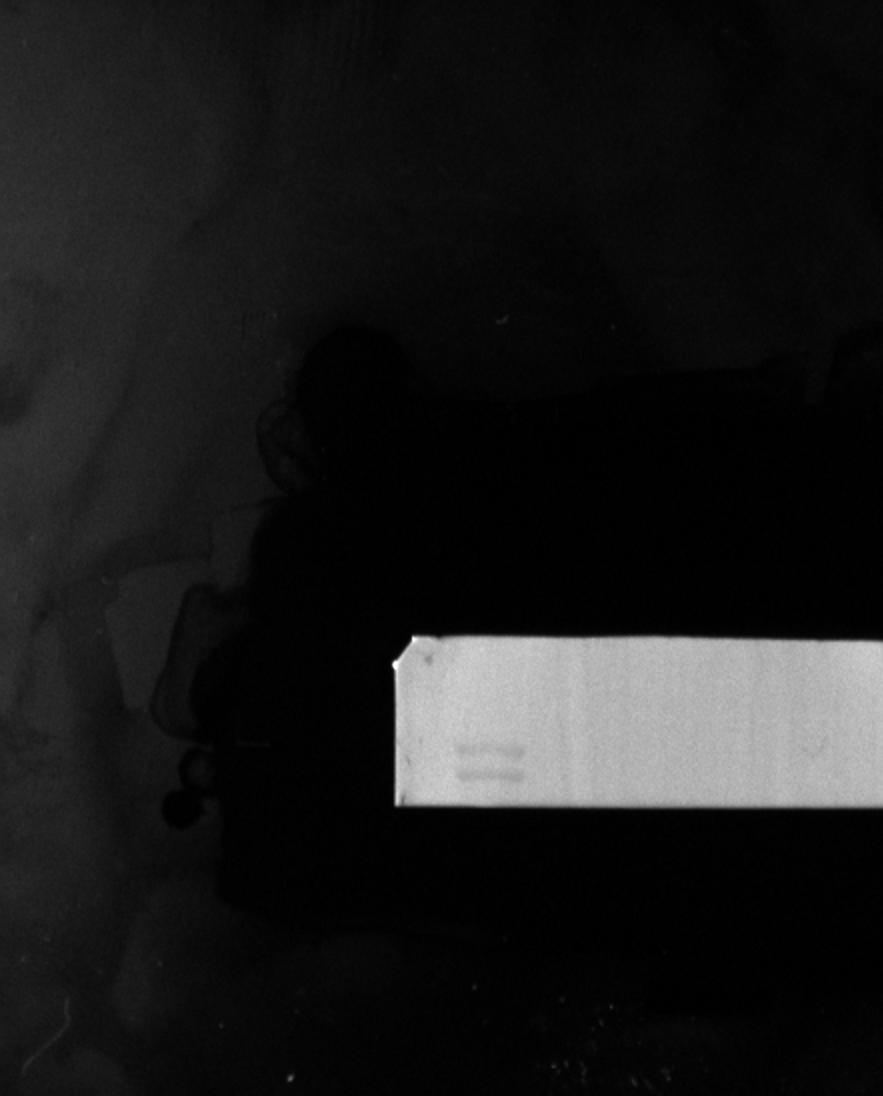

Supplement: Figure 3—source data 2. [file elife-104060-fig3-data2.zip › Figure 3-source data2/p-mTOR/pmtor white.Tif]

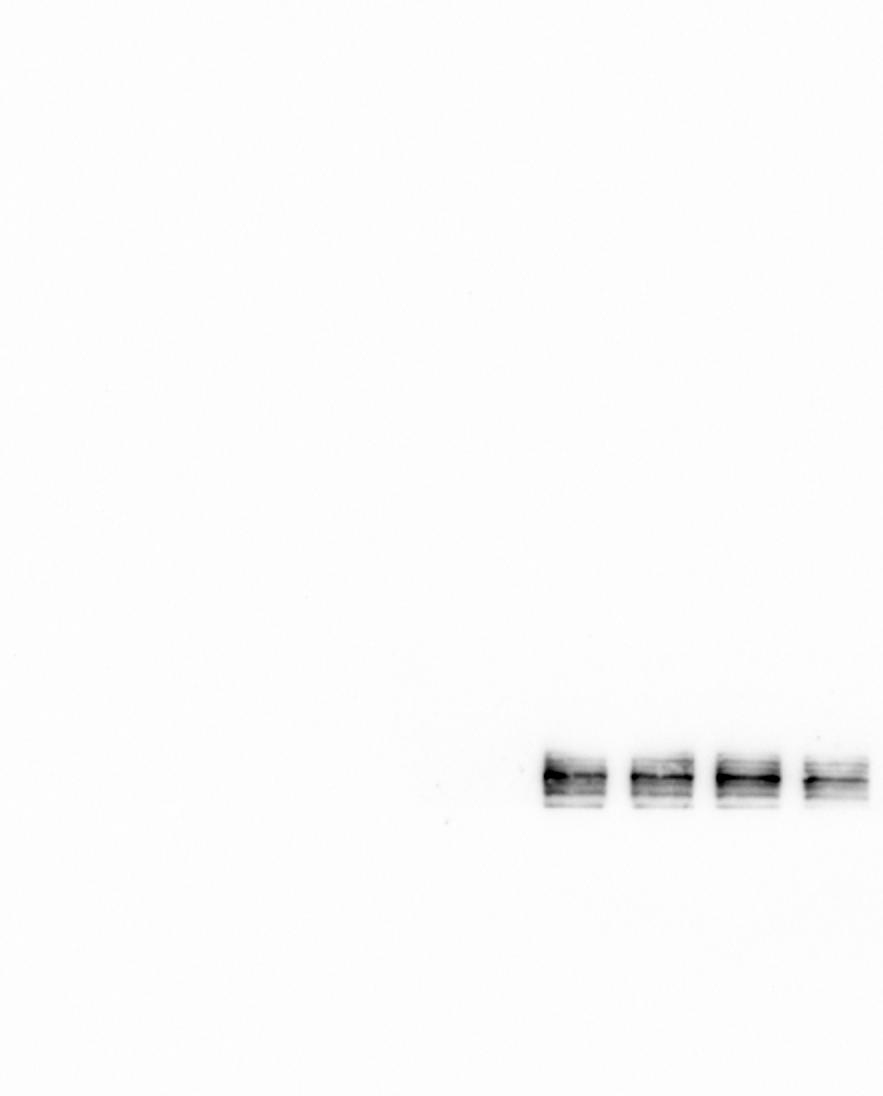

Supplement: Figure 3—source data 2. [file elife-104060-fig3-data2.zip › Figure 3-source data2/p-mTOR/pmtor.Tif]

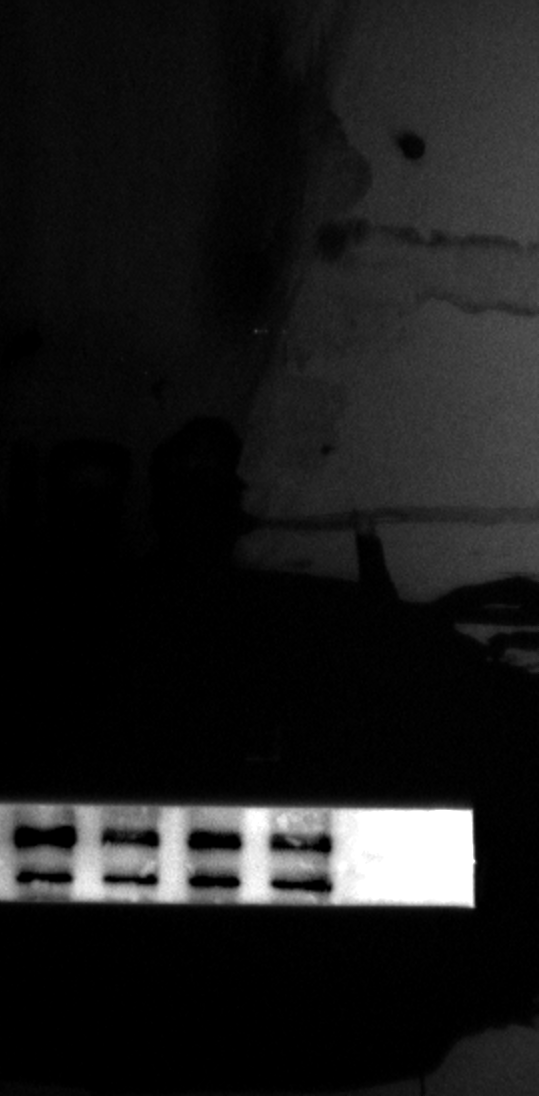

Supplement: Figure 3—source data 2. [file elife-104060-fig3-data2.zip › Figure 3-source data2/p-SHP2/PSHP2 MERGE.Tif]

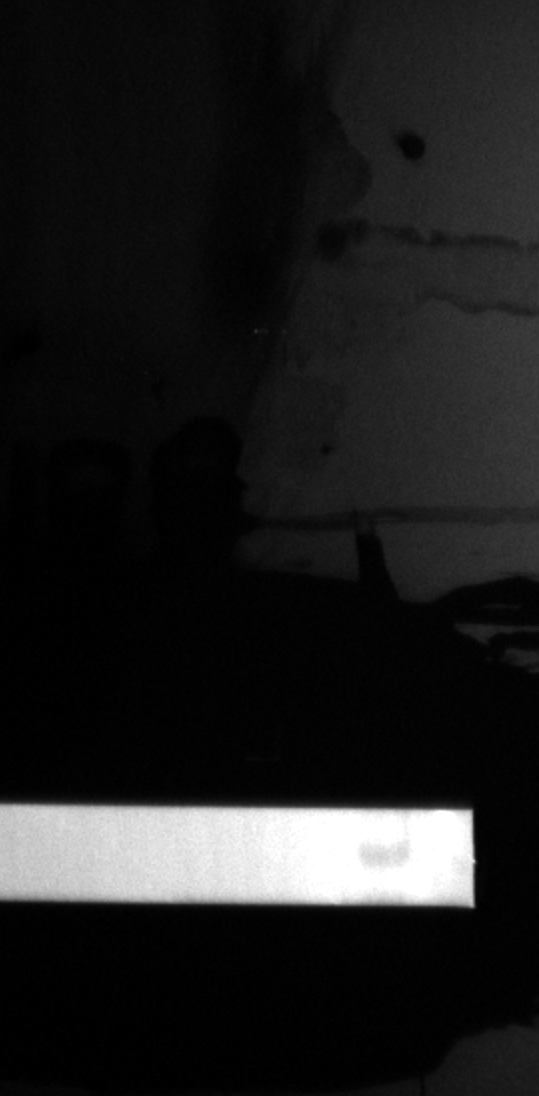

Supplement: Figure 3—source data 2. [file elife-104060-fig3-data2.zip › Figure 3-source data2/p-SHP2/PSHP2 WHITE.Tif]

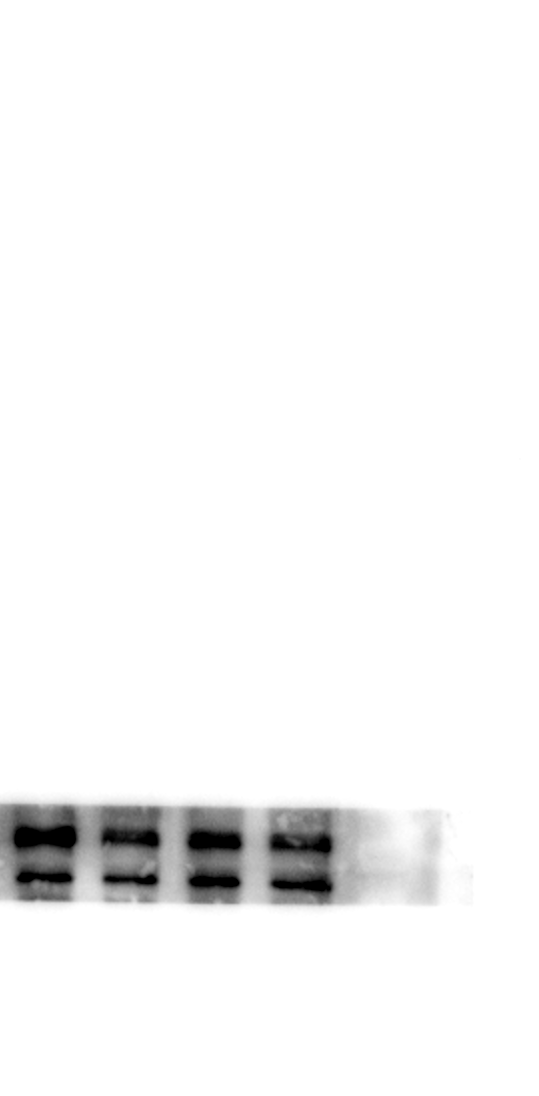

Supplement: Figure 3—source data 2. [file elife-104060-fig3-data2.zip › Figure 3-source data2/p-SHP2/PSHP2.Tif]

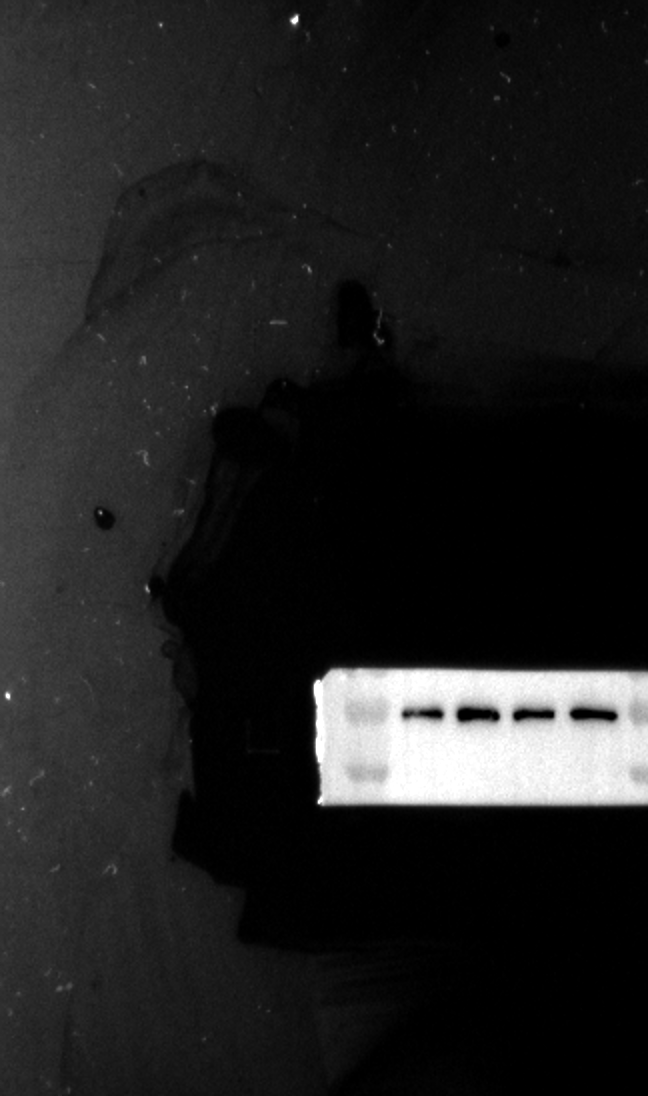

Supplement: Figure 3—source data 2. [file elife-104060-fig3-data2.zip › Figure 3-source data2/SHP2/1 shp2 merge.Tif]

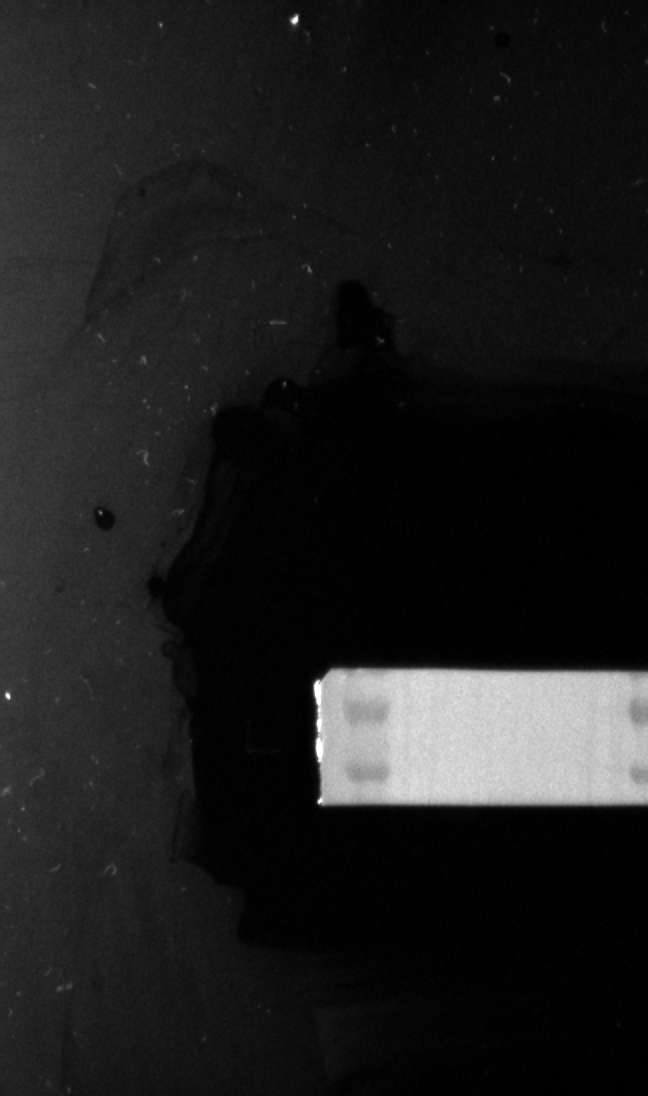

Supplement: Figure 3—source data 2. [file elife-104060-fig3-data2.zip › Figure 3-source data2/SHP2/1 shp2 white.Tif]

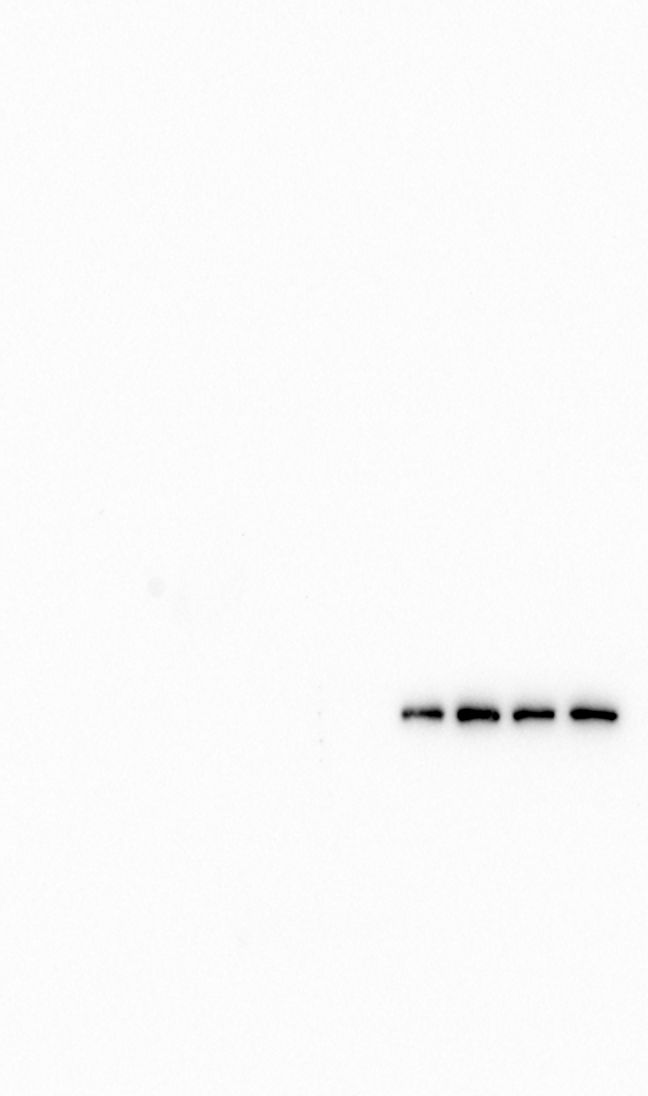

Supplement: Figure 3—source data 2. [file elife-104060-fig3-data2.zip › Figure 3-source data2/SHP2/1 shp2.Tif]

Figure 4

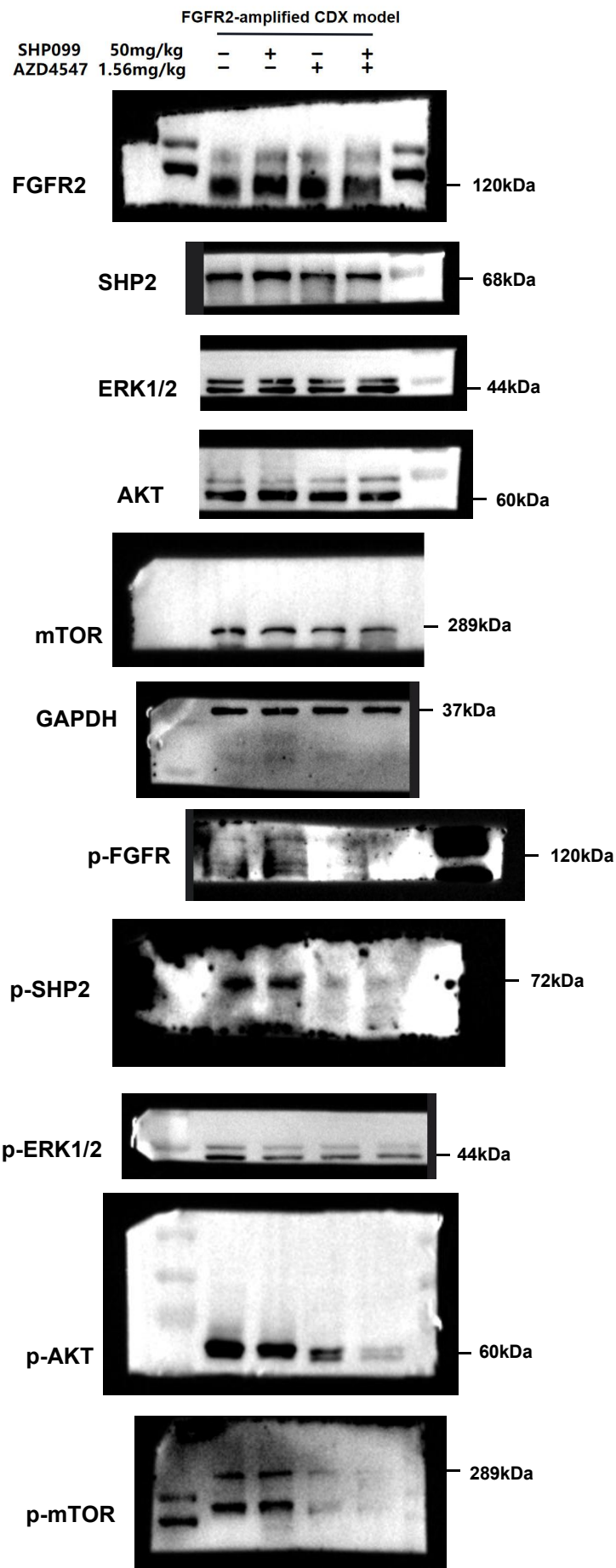

Supplement: Figure 4—source data 1. [file elife-104060-fig4-data1.pdf]

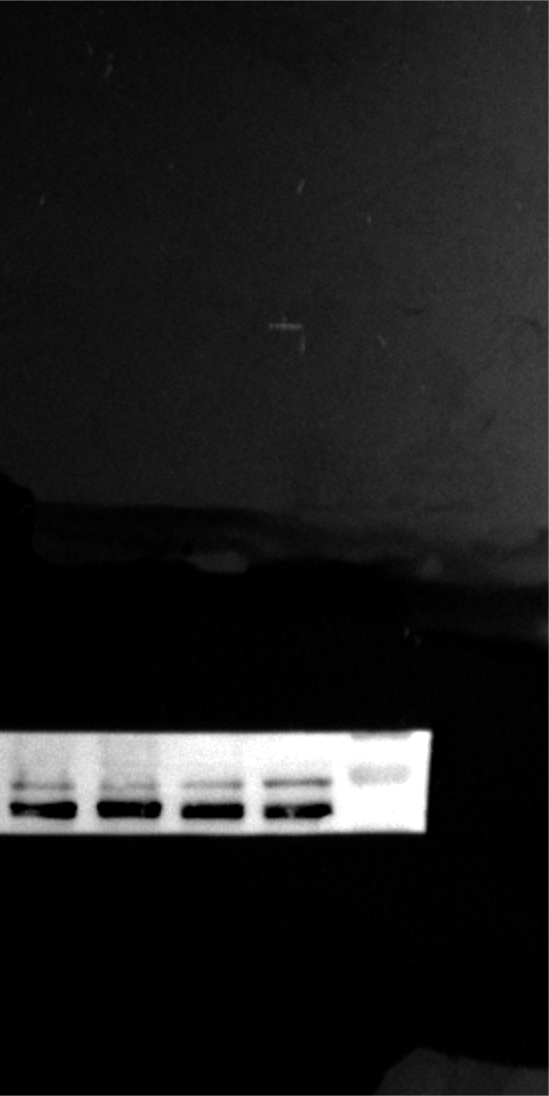

Supplement: Figure 4—source data 2. [file elife-104060-fig4-data2.zip › Figure 4-source data 2/AKT/akt2 merge=.png]

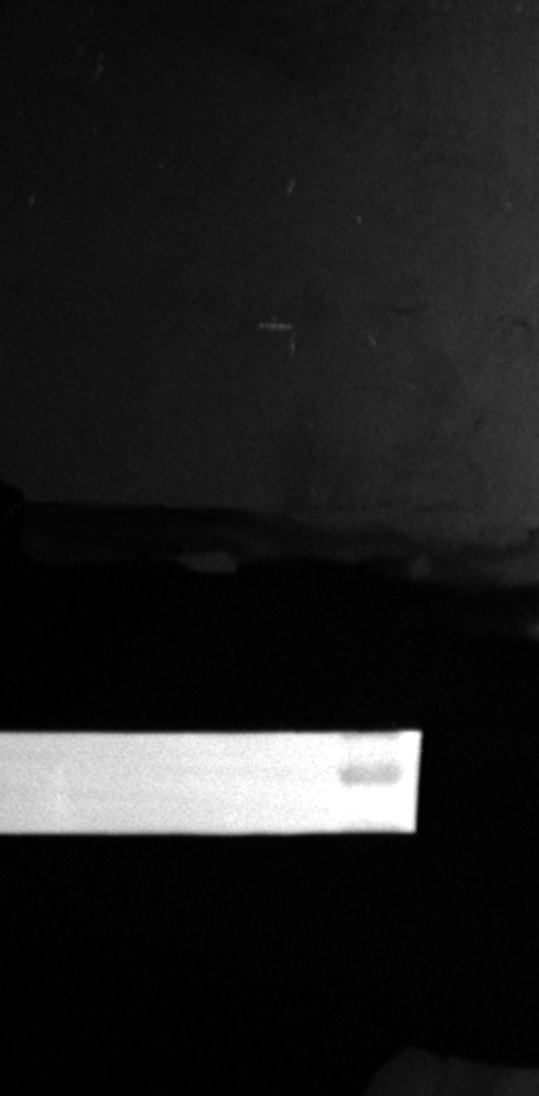

Supplement: Figure 4—source data 2. [file elife-104060-fig4-data2.zip › Figure 4-source data 2/AKT/akt2 white=.Tif]

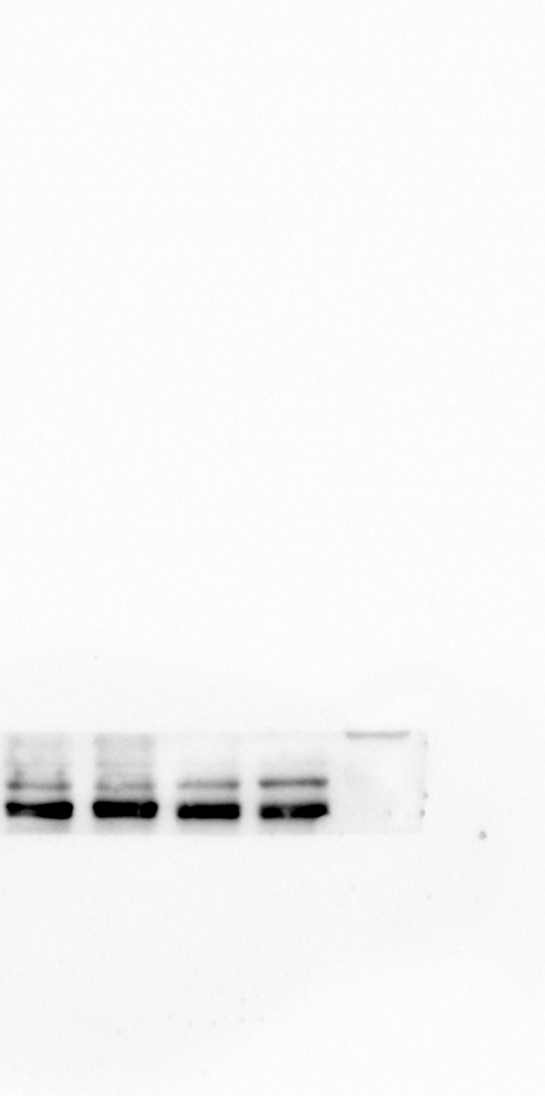

Supplement: Figure 4—source data 2. [file elife-104060-fig4-data2.zip › Figure 4-source data 2/AKT/akt2=.Tif]

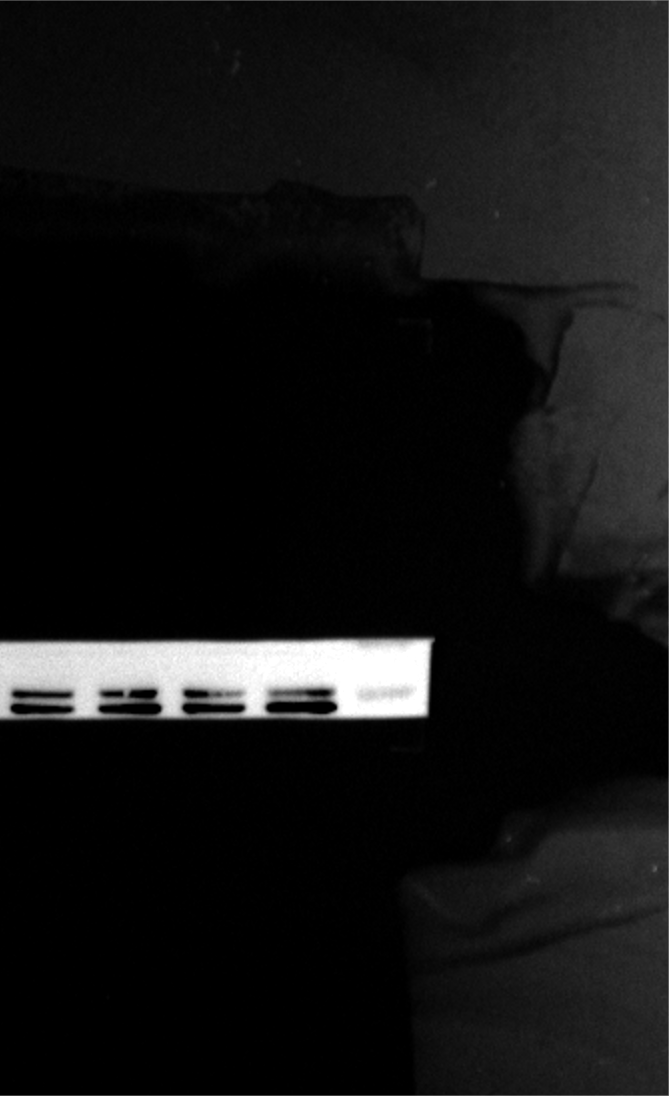

Supplement: Figure 4—source data 2. [file elife-104060-fig4-data2.zip › Figure 4-source data 2/ERK/erk merge=.png]

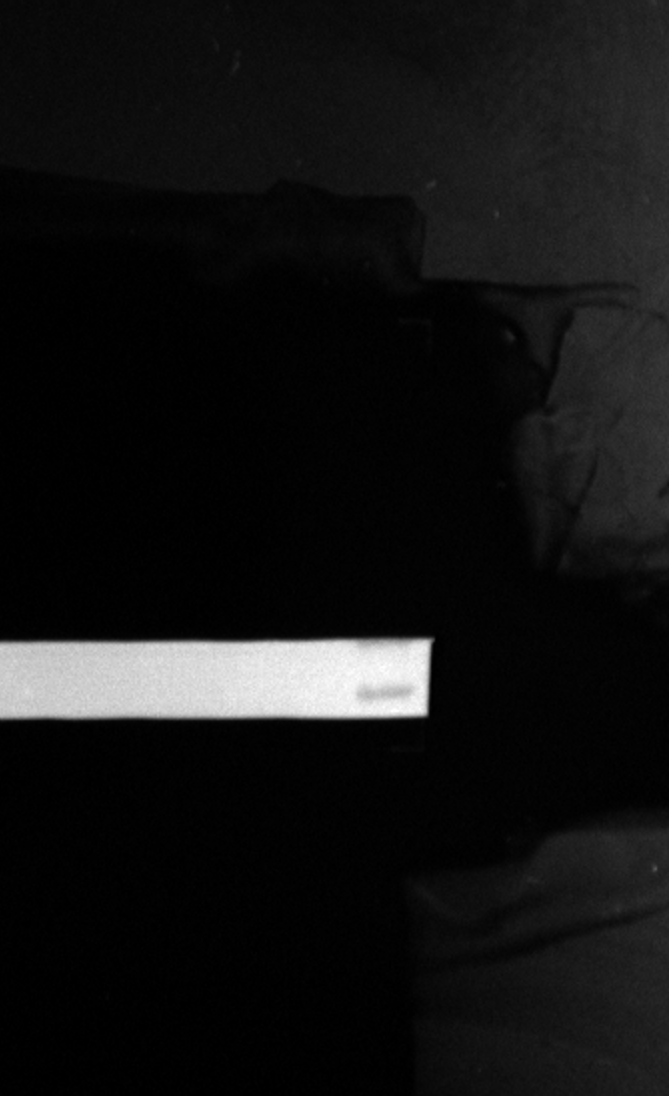

Supplement: Figure 4—source data 2. [file elife-104060-fig4-data2.zip › Figure 4-source data 2/ERK/erk white=.Tif]

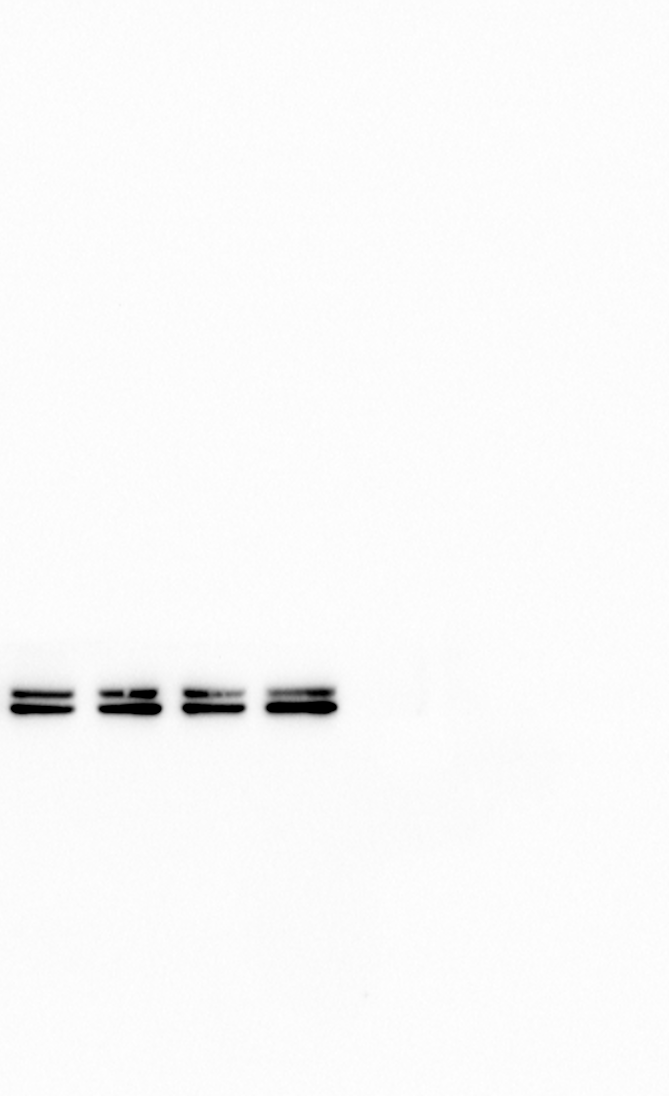

Supplement: Figure 4—source data 2. [file elife-104060-fig4-data2.zip › Figure 4-source data 2/ERK/erk=.Tif]

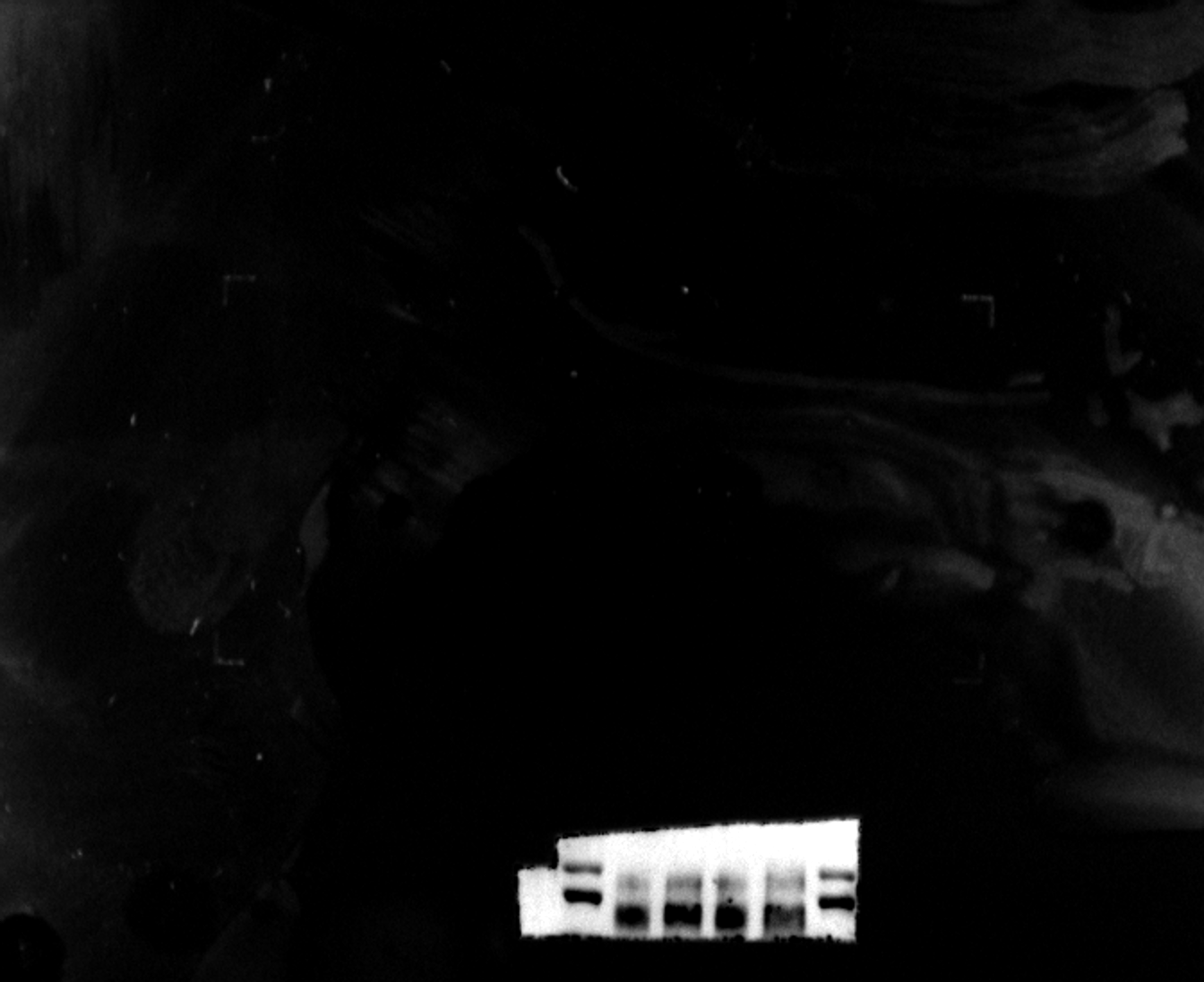

Supplement: Figure 4—source data 2. [file elife-104060-fig4-data2.zip › Figure 4-source data 2/FGFR2/fgfr2 merge.Tif]

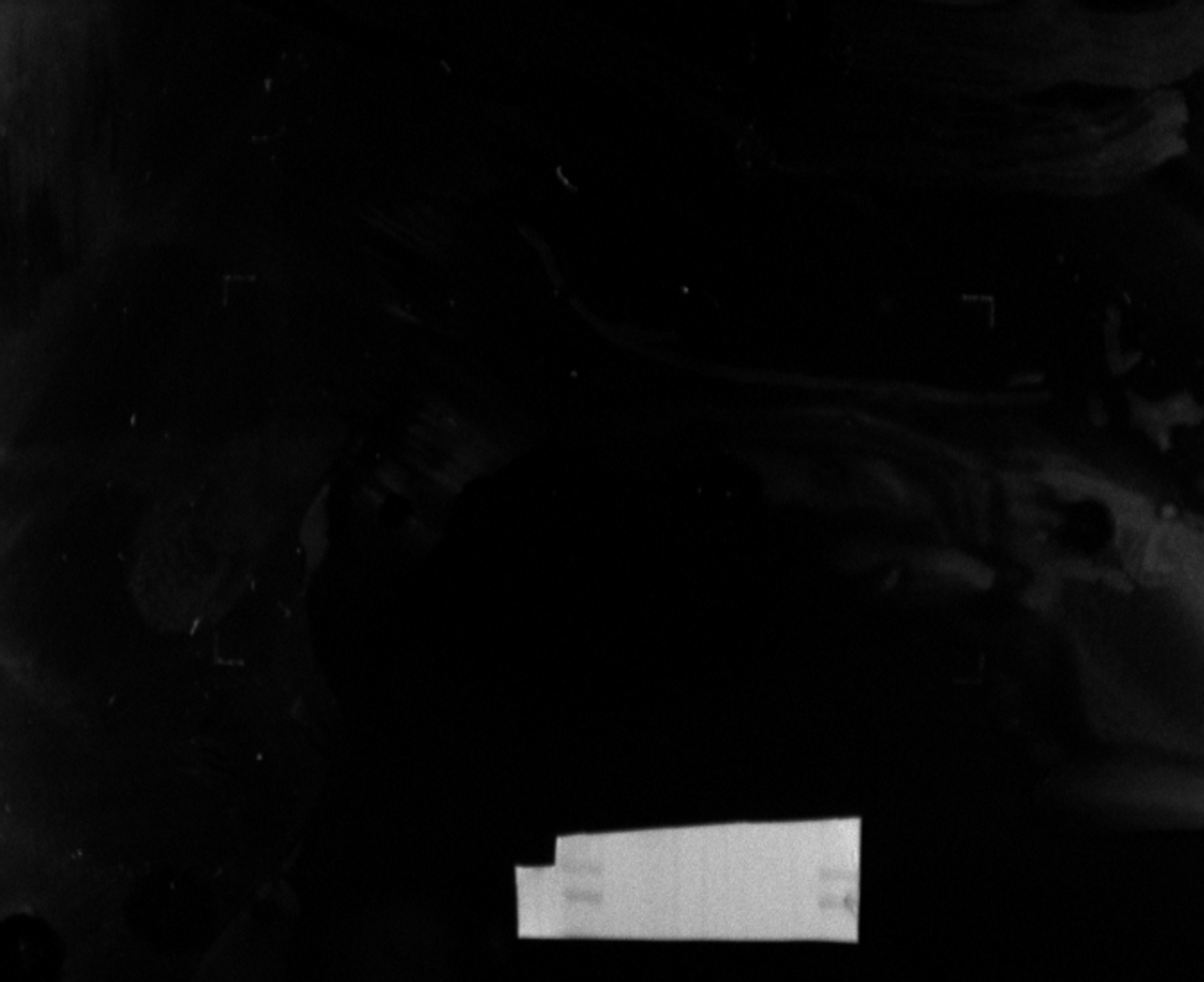

Supplement: Figure 4—source data 2. [file elife-104060-fig4-data2.zip › Figure 4-source data 2/FGFR2/fgfr2 white.Tif]

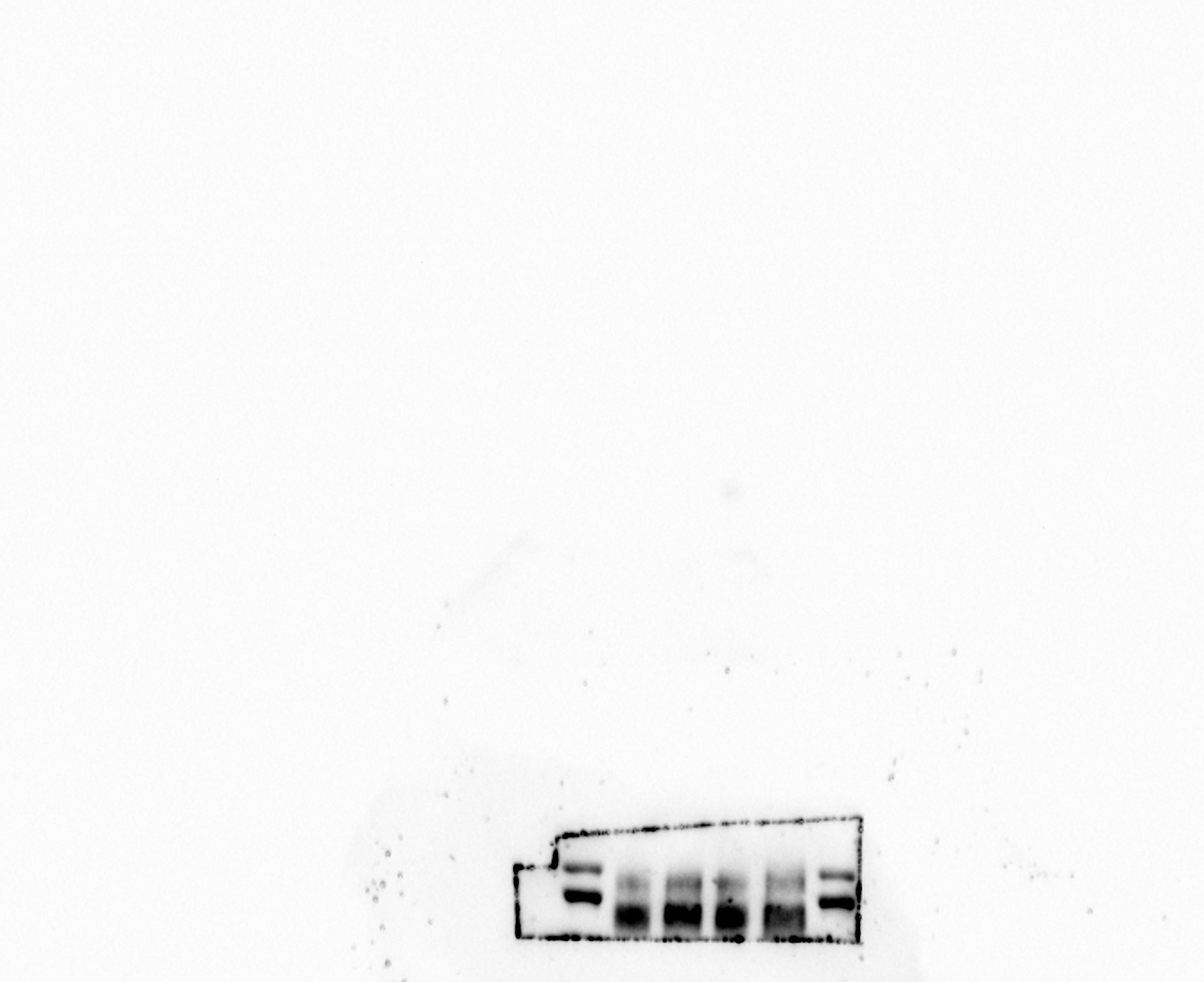

Supplement: Figure 4—source data 2. [file elife-104060-fig4-data2.zip › Figure 4-source data 2/FGFR2/fgfr2.Tif]
