# Supplementary figures and images for: Blocking SHP2 benefits FGFR2 inhibitor and overcomes its resistance in FGFR2-amplified gastric cancer (part 1 of 3)
Source: eLife. 2026 Mar 23;14:RP104060. doi: 10.7554/eLife.104060 (PMC13008354; doi:10.7554/eLife.104060)

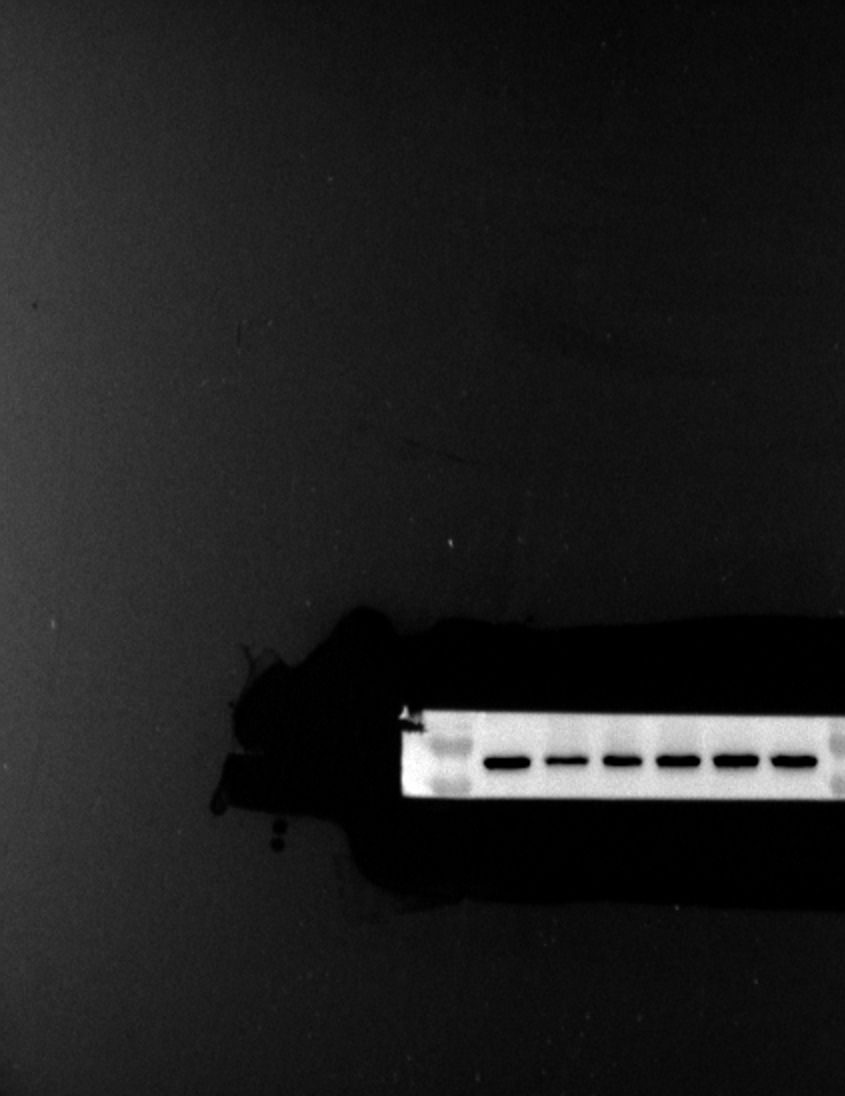

Supplement: Figure 2—source data 2. [file elife-104060-fig2-data2.zip › Figure 2-source data 2/2E/1h/AKT/akt merge.Tif]

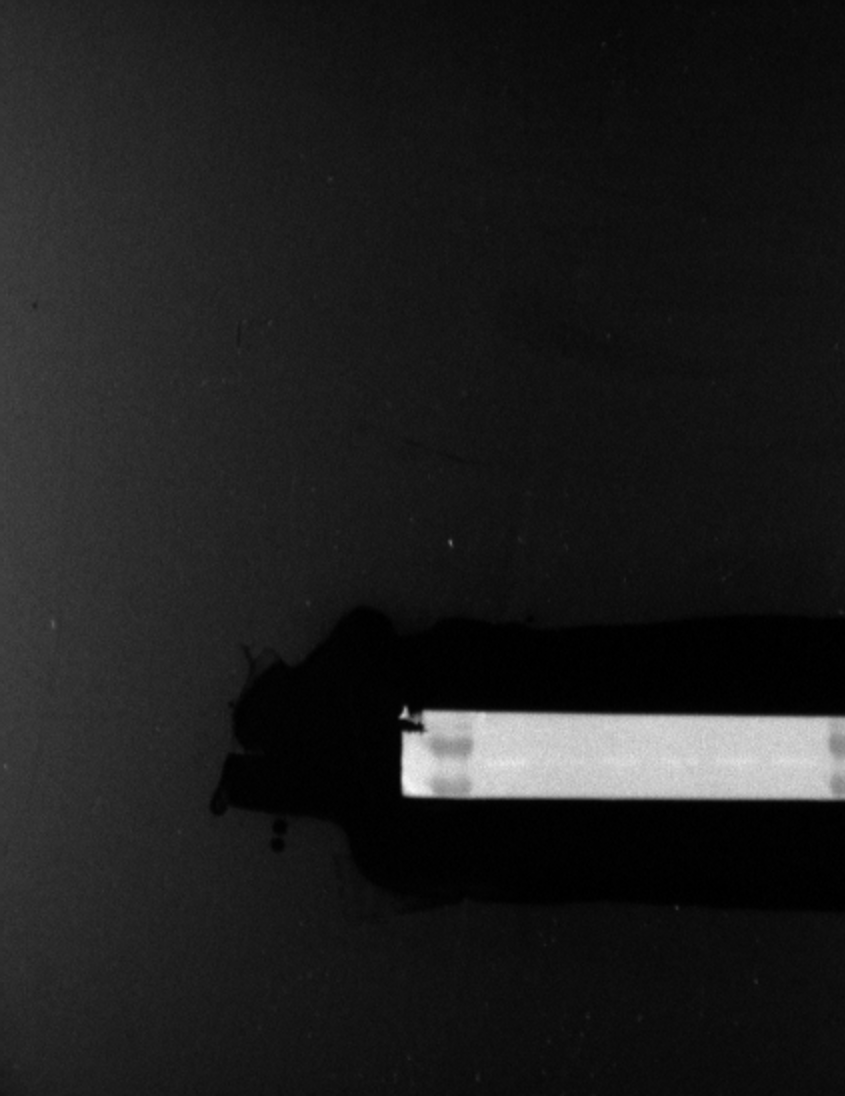

Supplement: Figure 2—source data 2. [file elife-104060-fig2-data2.zip › Figure 2-source data 2/2E/1h/AKT/akt white.Tif]

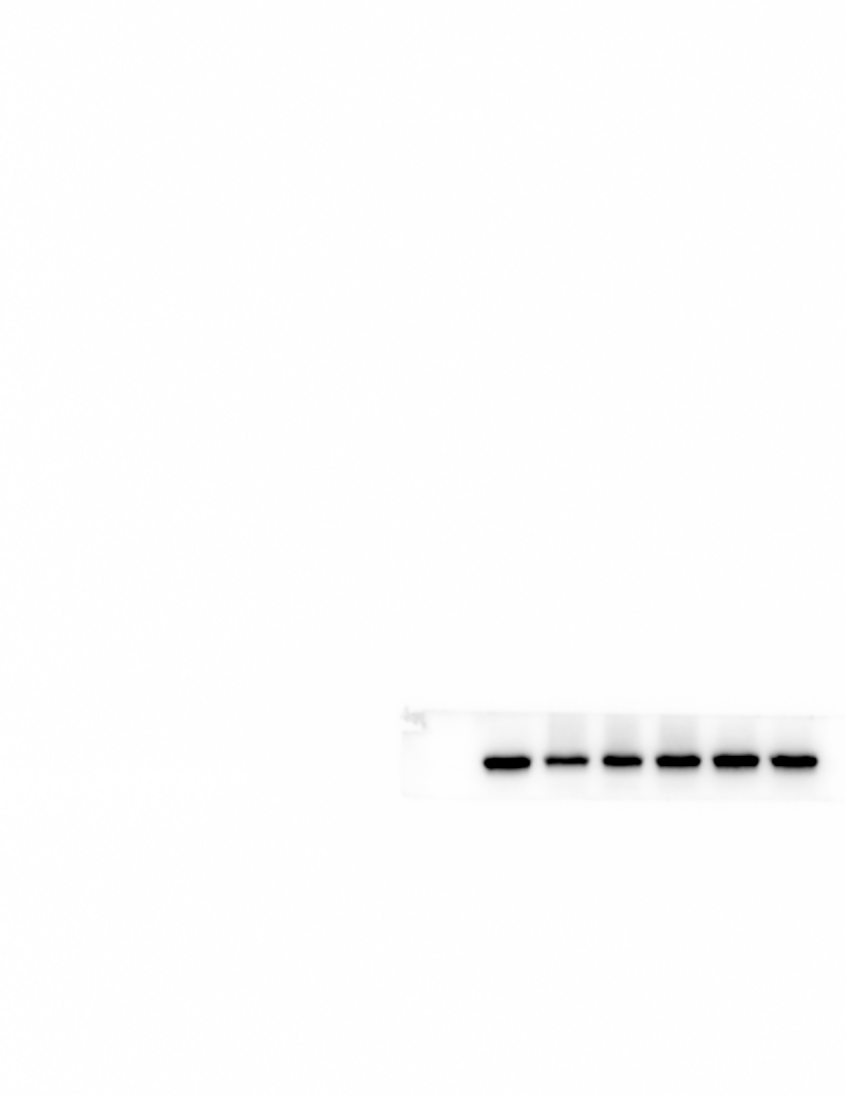

Supplement: Figure 2—source data 2. [file elife-104060-fig2-data2.zip › Figure 2-source data 2/2E/1h/AKT/akt.Tif]

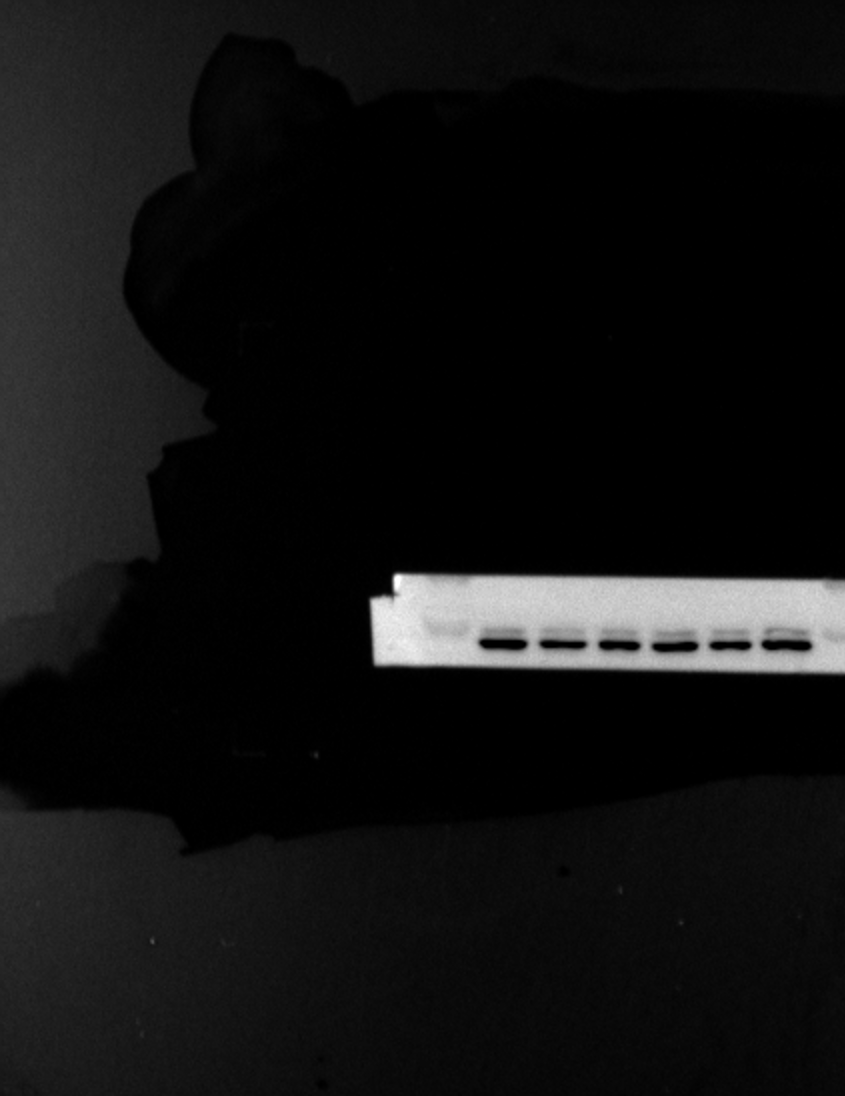

Supplement: Figure 2—source data 2. [file elife-104060-fig2-data2.zip › Figure 2-source data 2/2E/1h/ERK/erk merge.Tif]

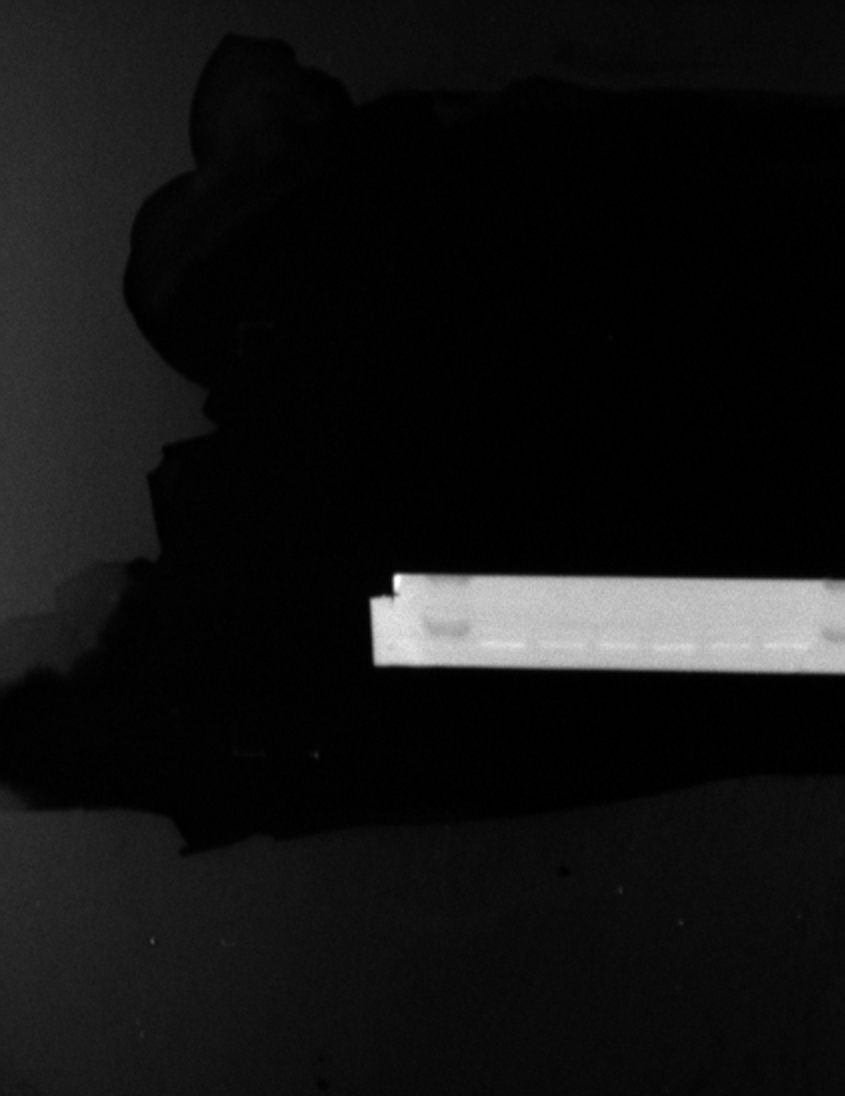

Supplement: Figure 2—source data 2. [file elife-104060-fig2-data2.zip › Figure 2-source data 2/2E/1h/ERK/erk white.Tif]

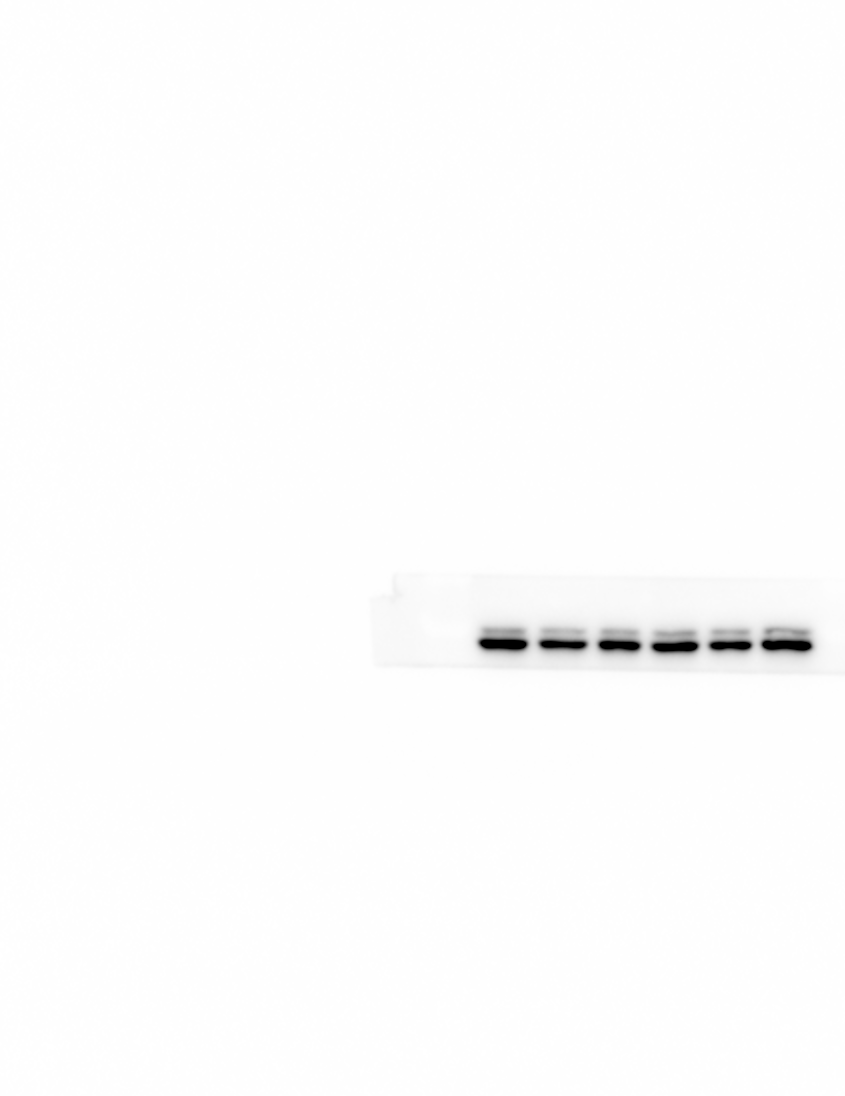

Supplement: Figure 2—source data 2. [file elife-104060-fig2-data2.zip › Figure 2-source data 2/2E/1h/ERK/erk.Tif]

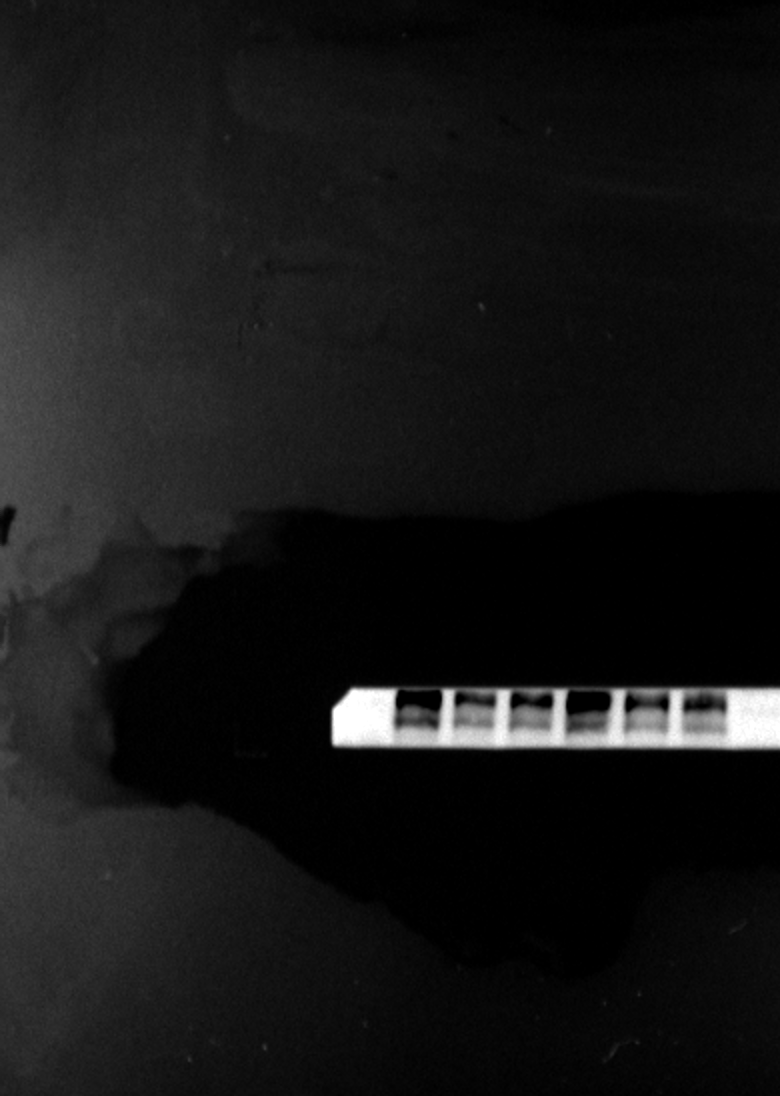

Supplement: Figure 2—source data 2. [file elife-104060-fig2-data2.zip › Figure 2-source data 2/2E/1h/FGFR2/1 fgfr2 merge.Tif]

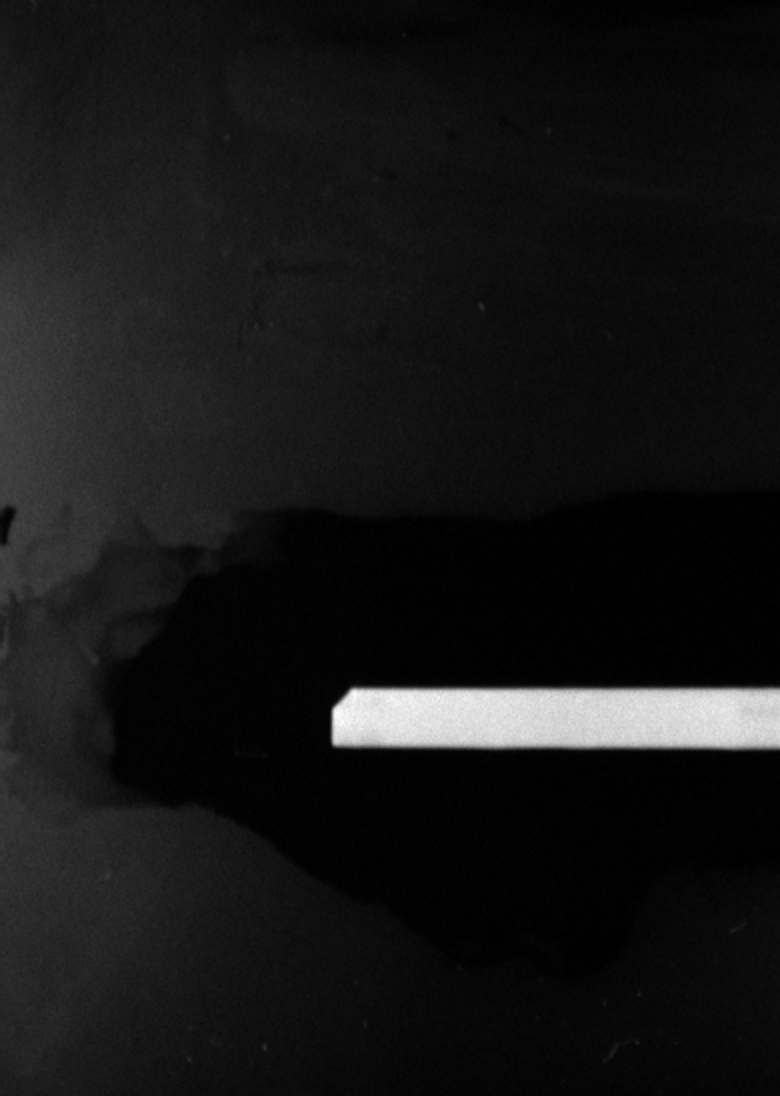

Supplement: Figure 2—source data 2. [file elife-104060-fig2-data2.zip › Figure 2-source data 2/2E/1h/FGFR2/1 fgfr2 white.Tif]

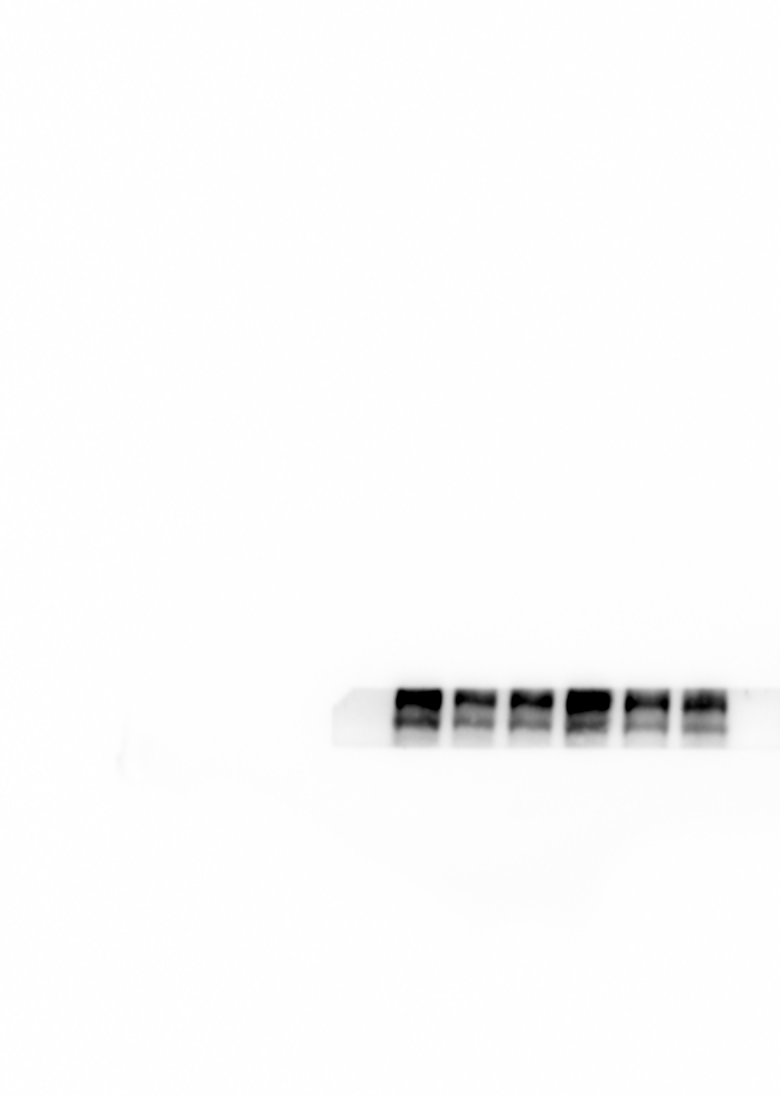

Supplement: Figure 2—source data 2. [file elife-104060-fig2-data2.zip › Figure 2-source data 2/2E/1h/FGFR2/1 fgfr2.Tif]

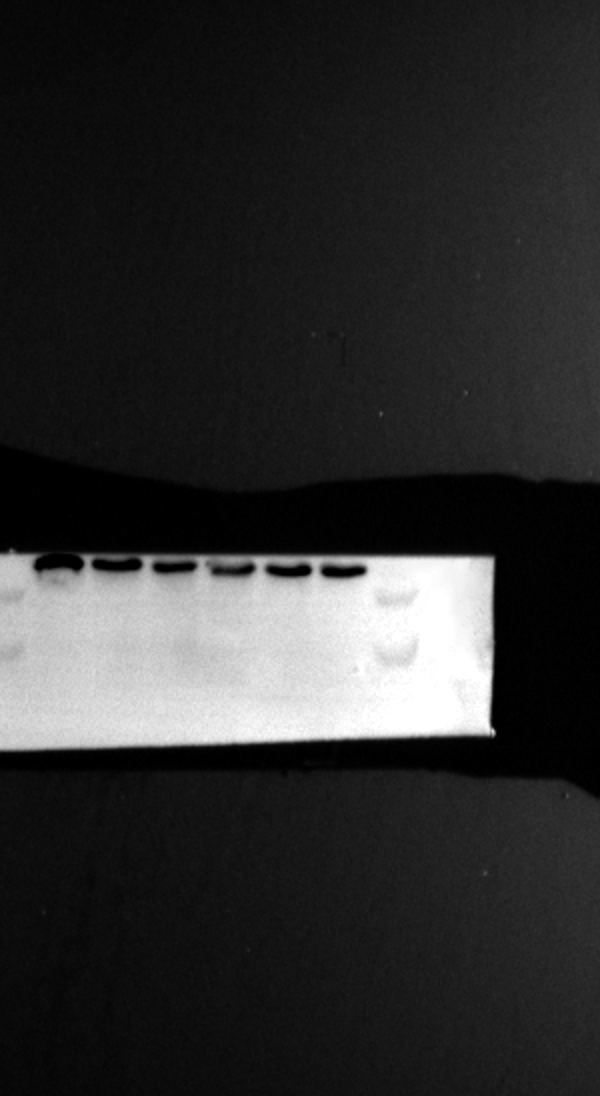

Supplement: Figure 2—source data 2. [file elife-104060-fig2-data2.zip › Figure 2-source data 2/2E/1h/GAPDH/1 gap merge.Tif]

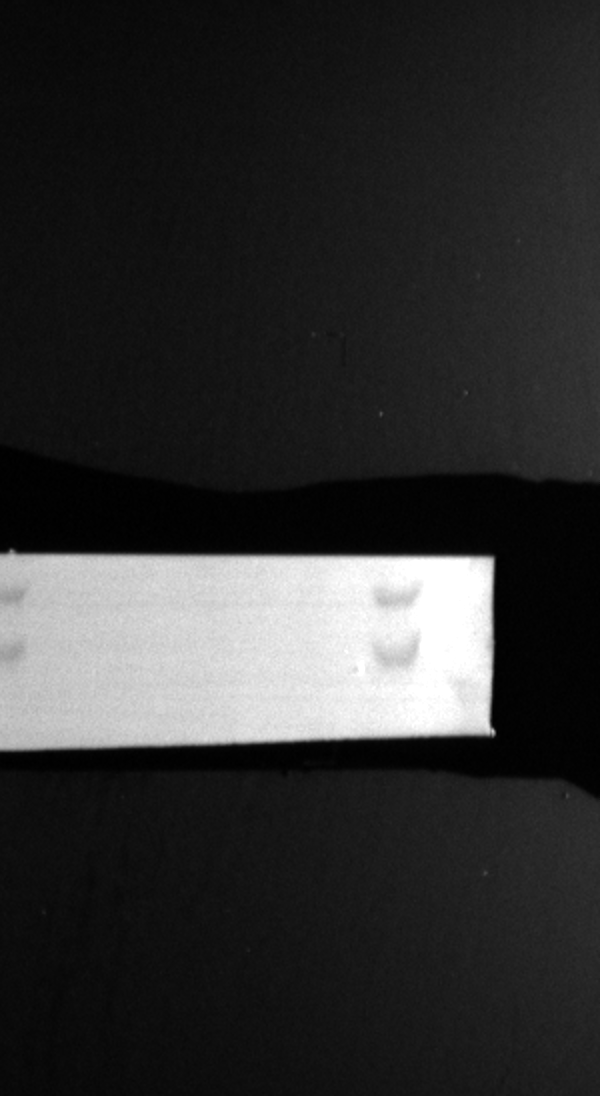

Supplement: Figure 2—source data 2. [file elife-104060-fig2-data2.zip › Figure 2-source data 2/2E/1h/GAPDH/1 gap white.Tif]

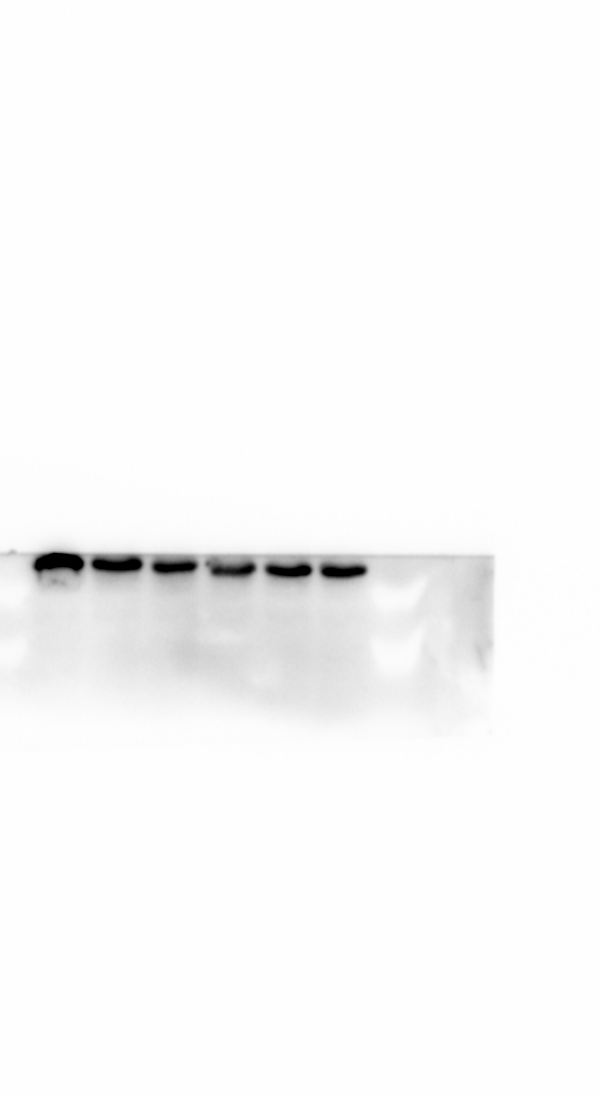

Supplement: Figure 2—source data 2. [file elife-104060-fig2-data2.zip › Figure 2-source data 2/2E/1h/GAPDH/1 gap.Tif]

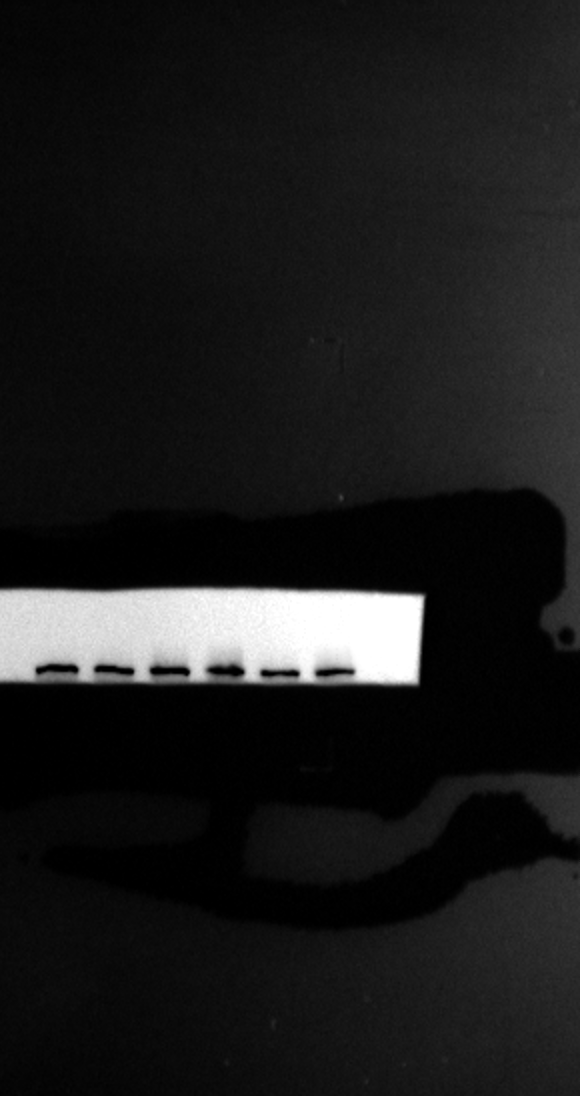

Supplement: Figure 2—source data 2. [file elife-104060-fig2-data2.zip › Figure 2-source data 2/2E/1h/mTOR/1 mtor merge.Tif]

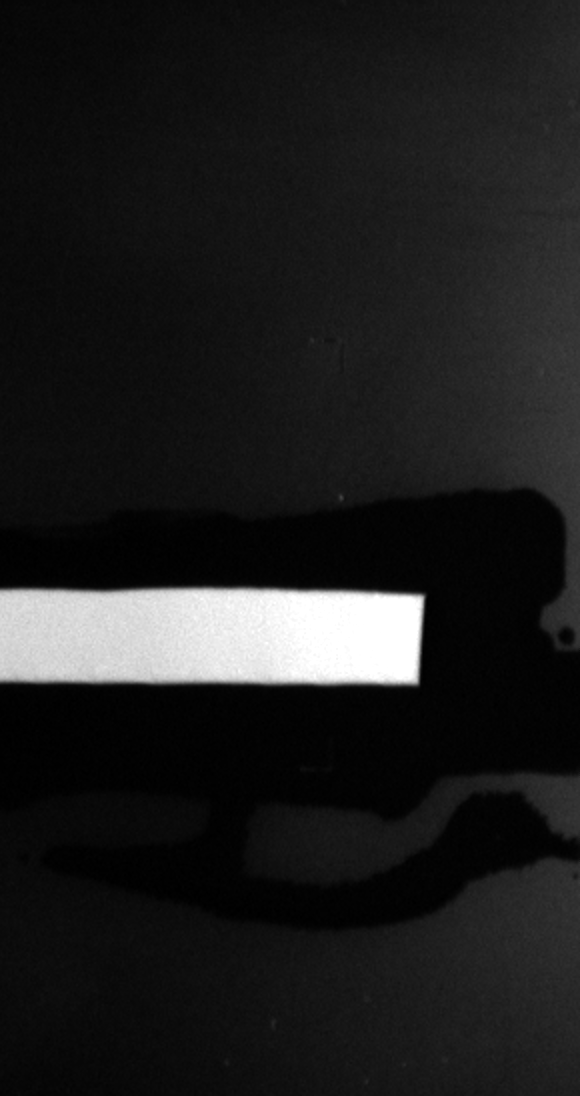

Supplement: Figure 2—source data 2. [file elife-104060-fig2-data2.zip › Figure 2-source data 2/2E/1h/mTOR/1 mtor white.Tif]

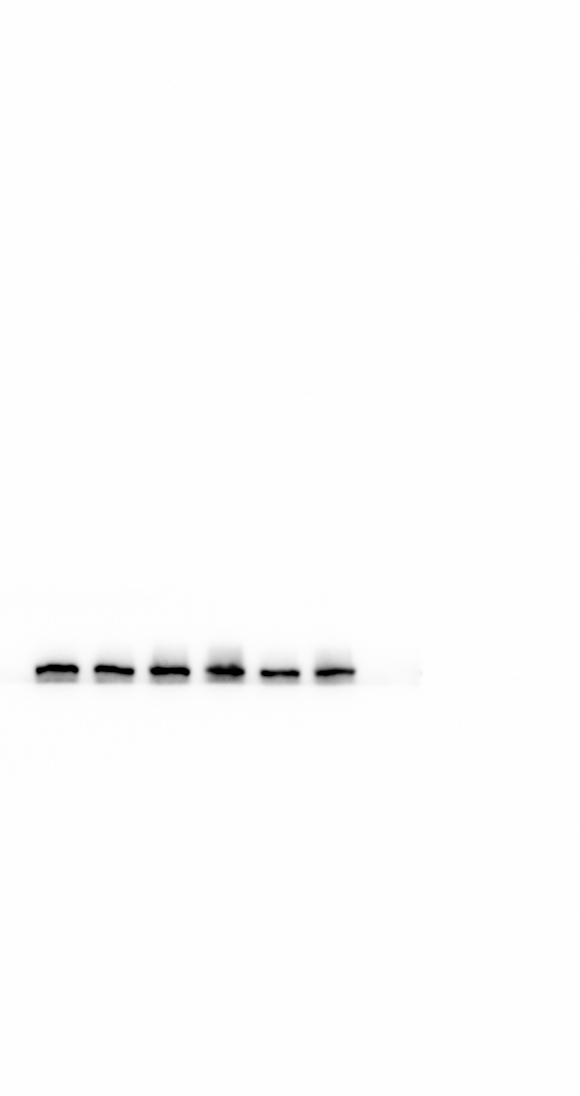

Supplement: Figure 2—source data 2. [file elife-104060-fig2-data2.zip › Figure 2-source data 2/2E/1h/mTOR/1 mtor.Tif]

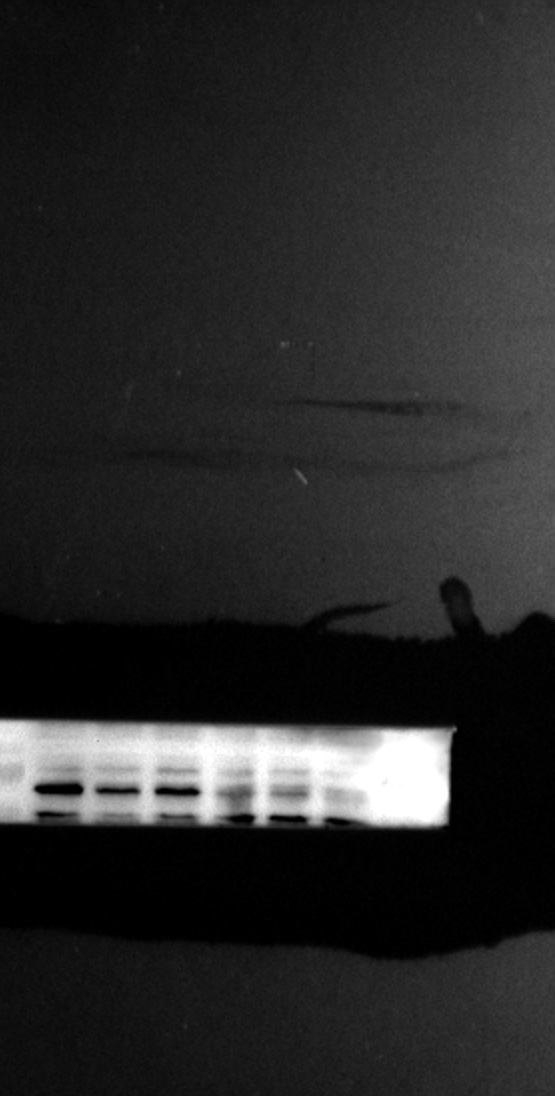

Supplement: Figure 2—source data 2. [file elife-104060-fig2-data2.zip › Figure 2-source data 2/2E/1h/p-AKT/p-akt merge.Tif]

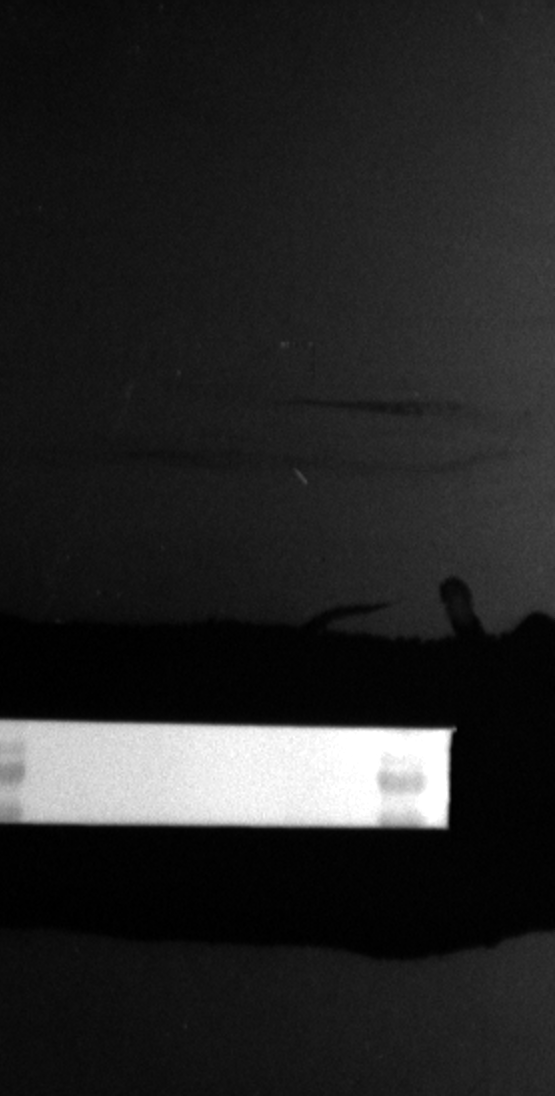

Supplement: Figure 2—source data 2. [file elife-104060-fig2-data2.zip › Figure 2-source data 2/2E/1h/p-AKT/p-akt white.Tif]

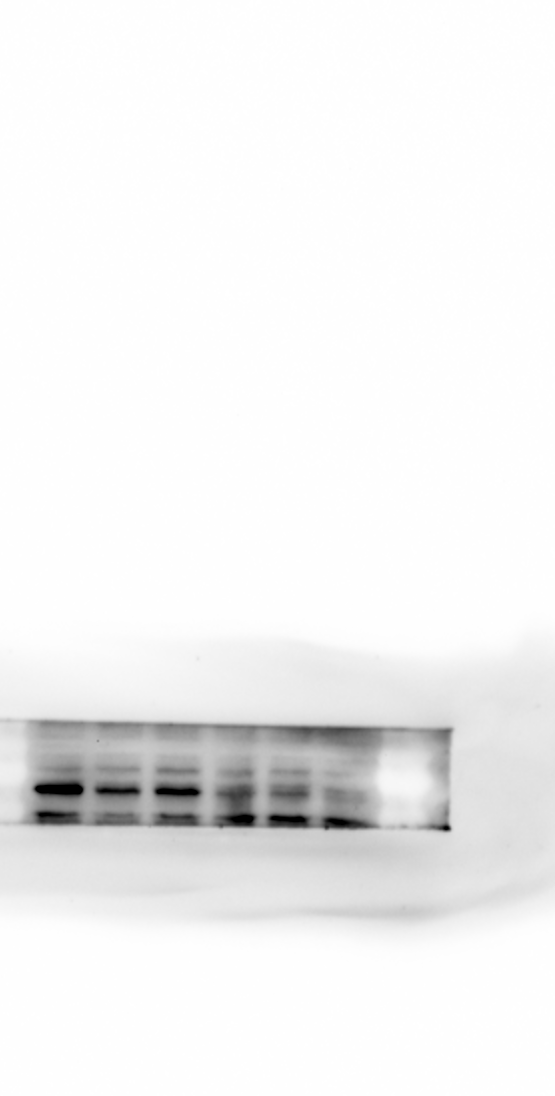

Supplement: Figure 2—source data 2. [file elife-104060-fig2-data2.zip › Figure 2-source data 2/2E/1h/p-AKT/p-akt.Tif]

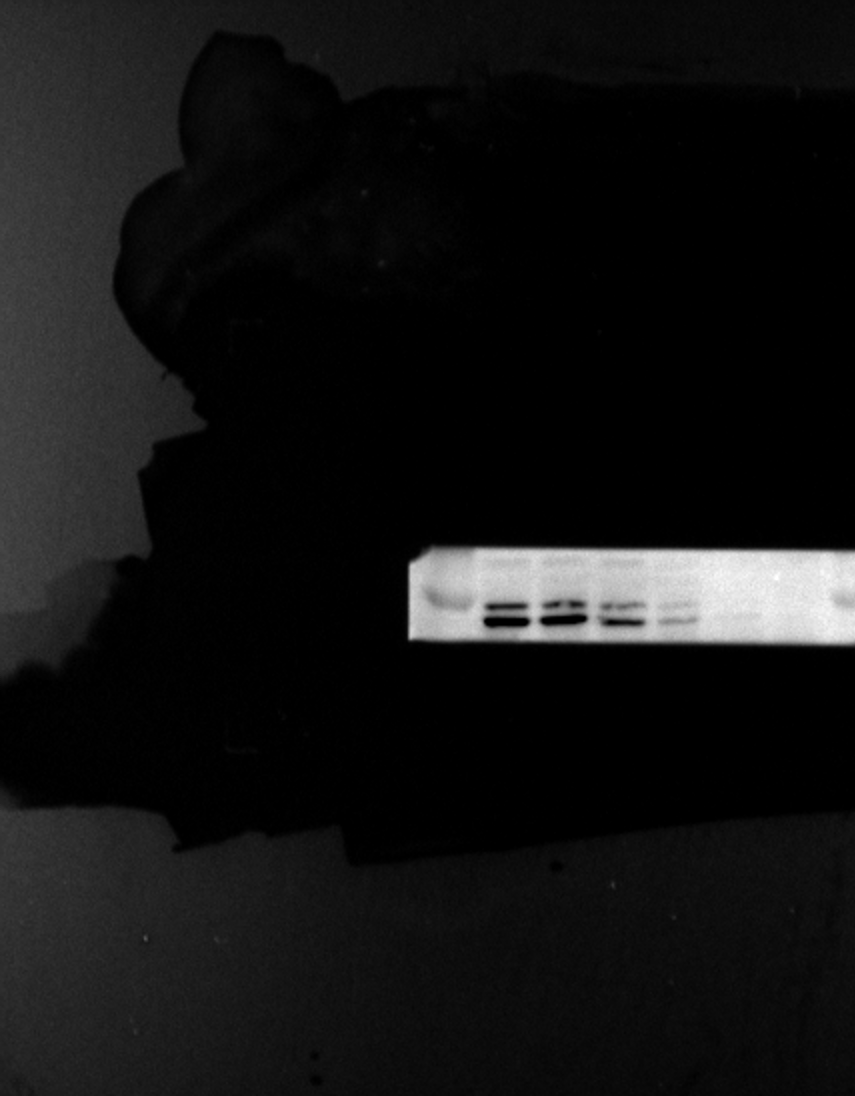

Supplement: Figure 2—source data 2. [file elife-104060-fig2-data2.zip › Figure 2-source data 2/2E/1h/p-ERK/perk merge.Tif]

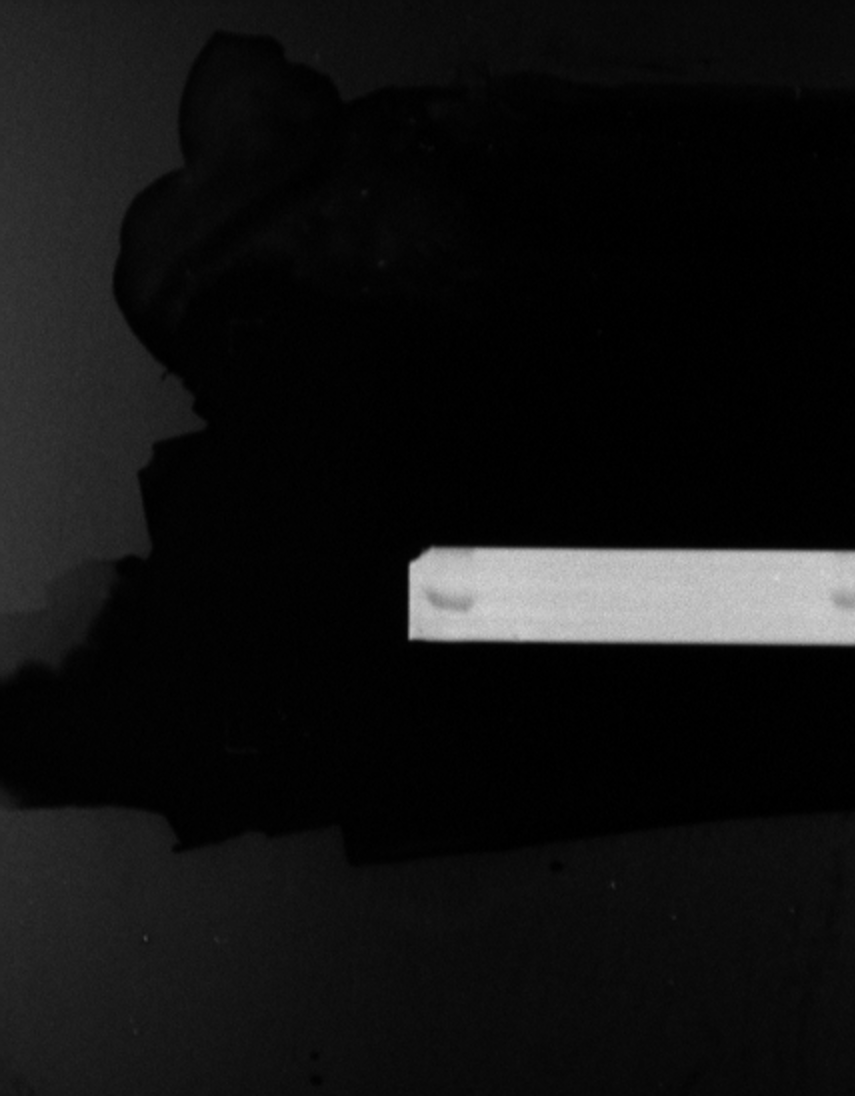

Supplement: Figure 2—source data 2. [file elife-104060-fig2-data2.zip › Figure 2-source data 2/2E/1h/p-ERK/perk white.Tif]

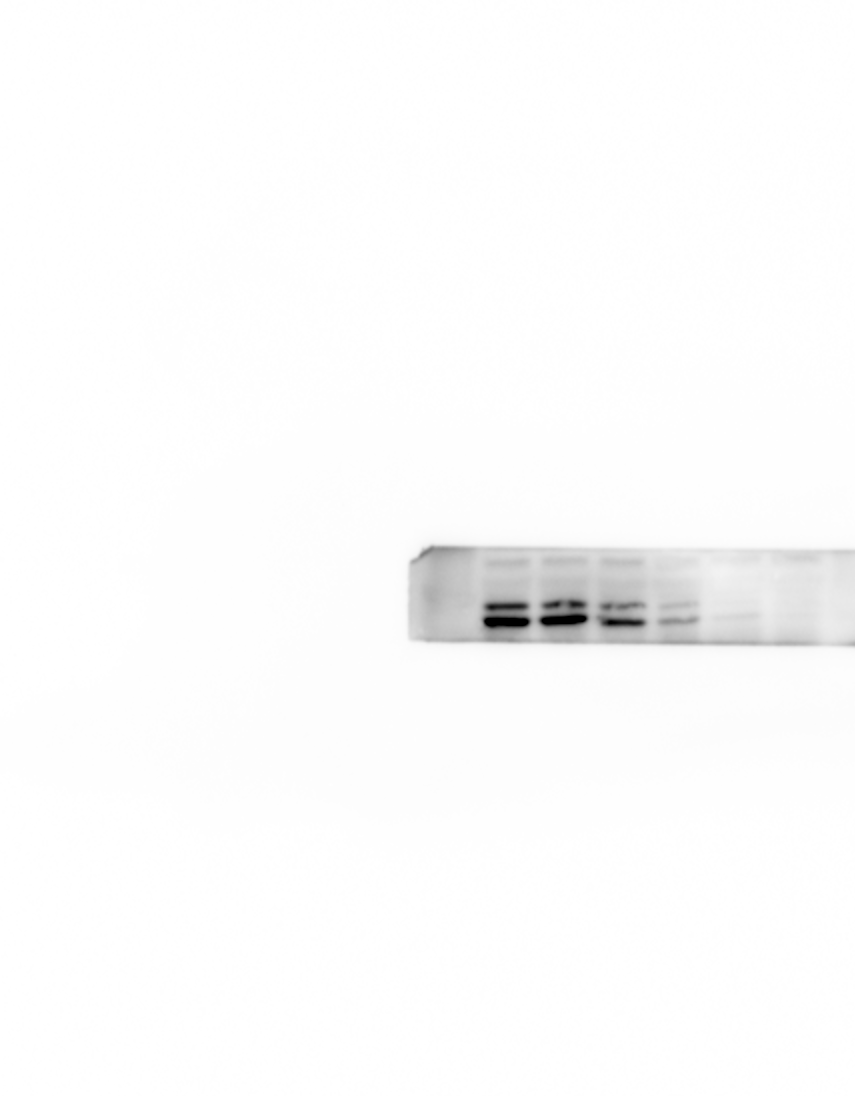

Supplement: Figure 2—source data 2. [file elife-104060-fig2-data2.zip › Figure 2-source data 2/2E/1h/p-ERK/perk.Tif]

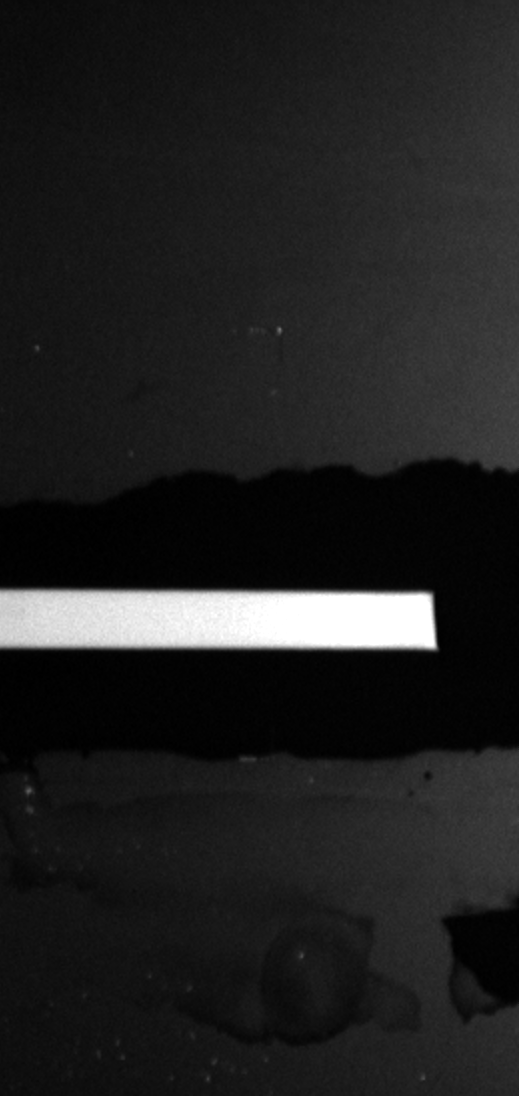

Supplement: Figure 2—source data 2. [file elife-104060-fig2-data2.zip › Figure 2-source data 2/2E/1h/p-FGFR/1-pfgfr white.Tif]

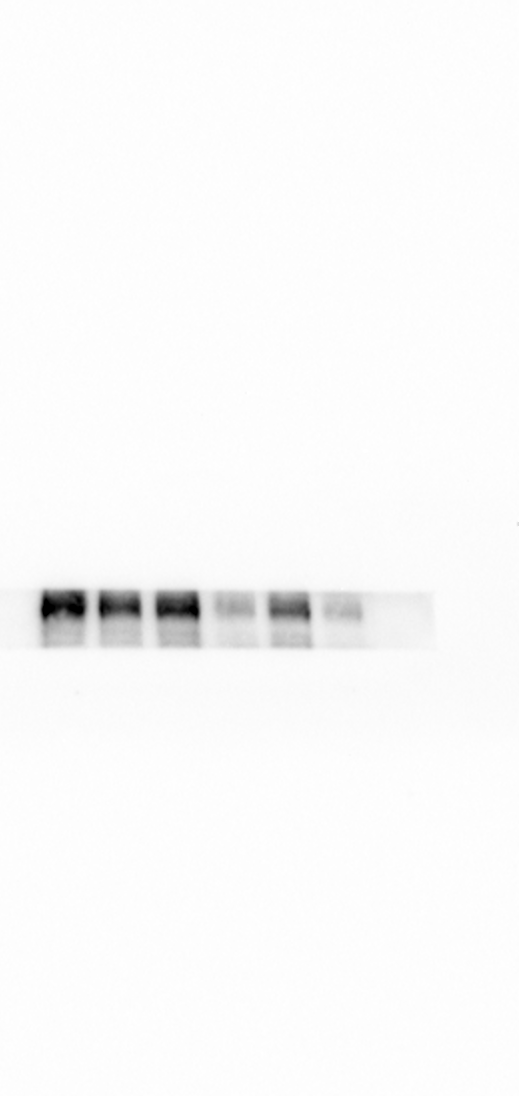

Supplement: Figure 2—source data 2. [file elife-104060-fig2-data2.zip › Figure 2-source data 2/2E/1h/p-FGFR/1-pfgfr.Tif]

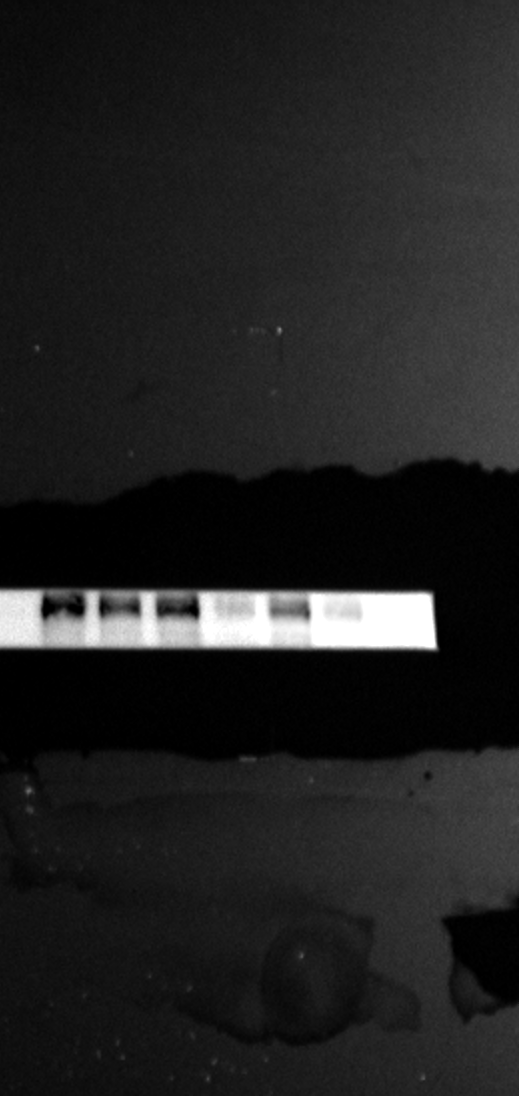

Supplement: Figure 2—source data 2. [file elife-104060-fig2-data2.zip › Figure 2-source data 2/2E/1h/p-FGFR/1-pfgfr2 merge.Tif]

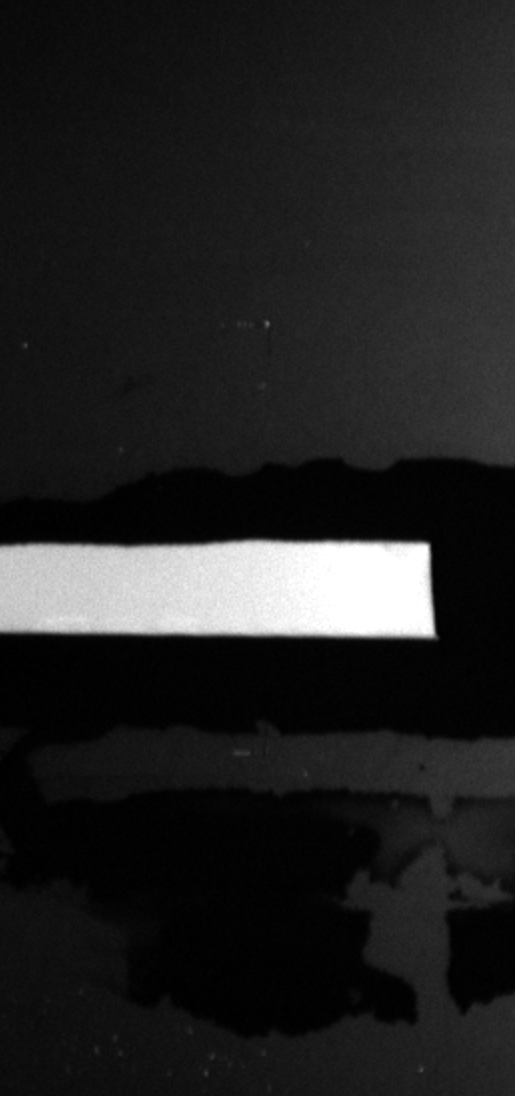

Supplement: Figure 2—source data 2. [file elife-104060-fig2-data2.zip › Figure 2-source data 2/2E/1h/p-mTOR/1-mtor white.Tif]

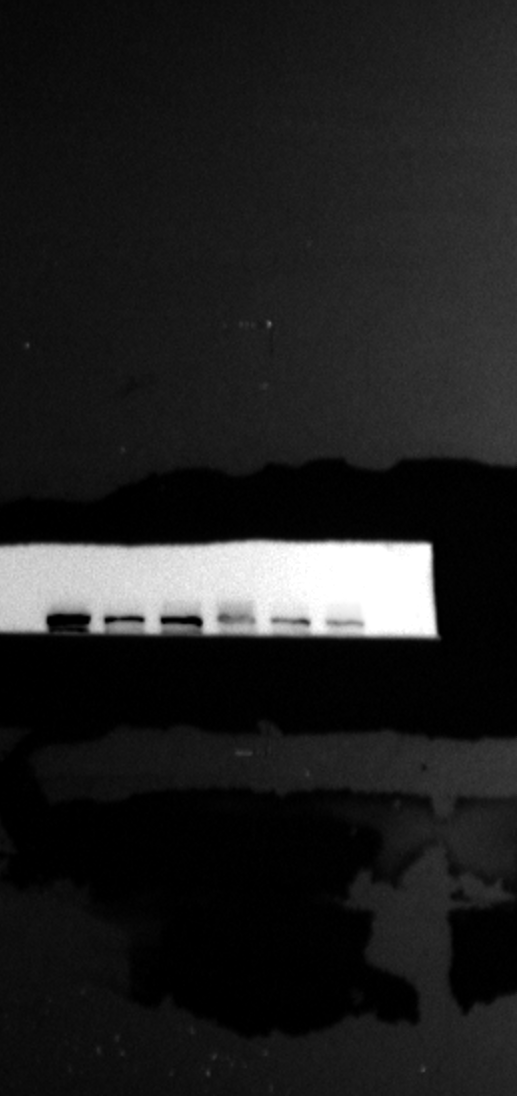

Supplement: Figure 2—source data 2. [file elife-104060-fig2-data2.zip › Figure 2-source data 2/2E/1h/p-mTOR/1-pmtor merge.Tif]

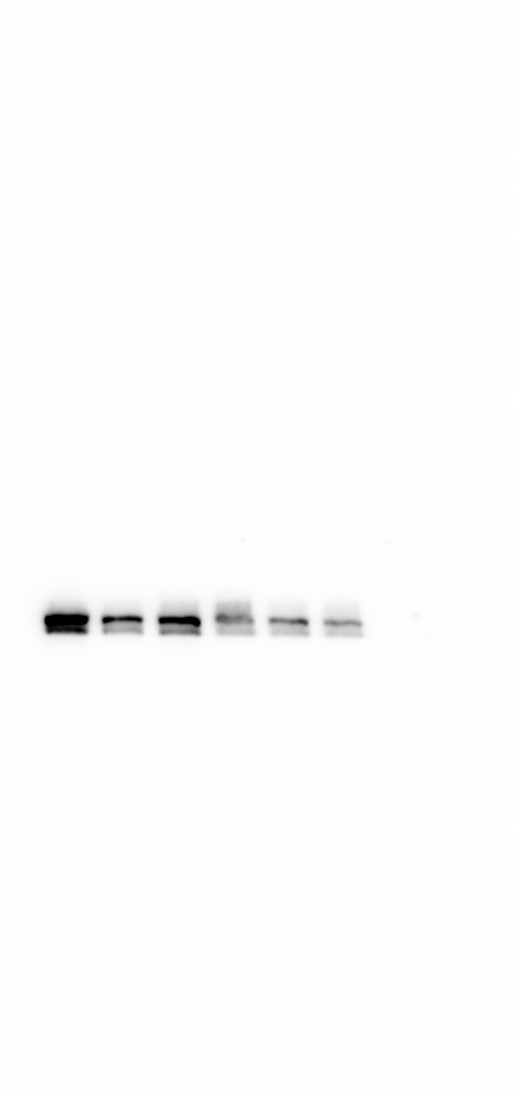

Supplement: Figure 2—source data 2. [file elife-104060-fig2-data2.zip › Figure 2-source data 2/2E/1h/p-mTOR/1-pmtor.Tif]

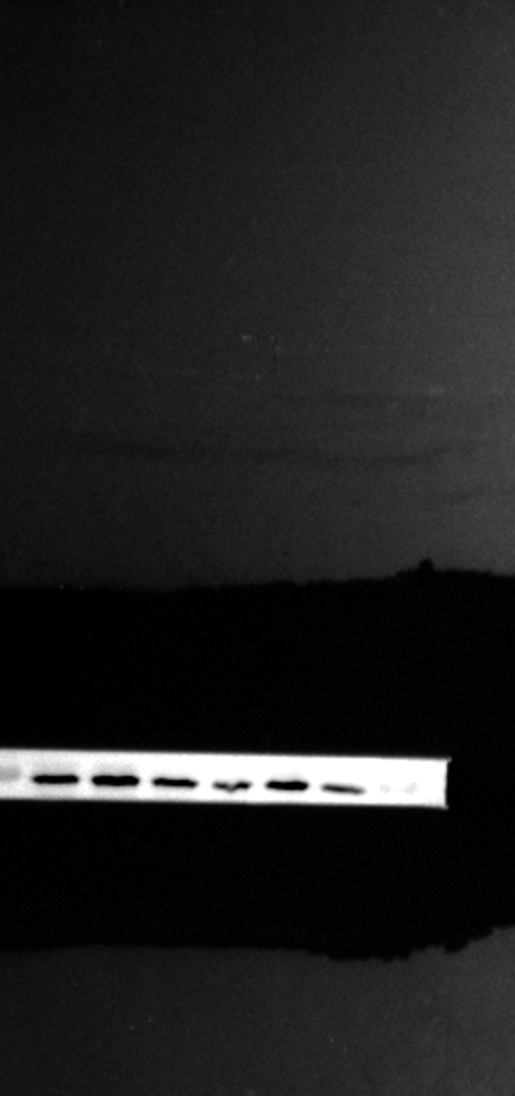

Supplement: Figure 2—source data 2. [file elife-104060-fig2-data2.zip › Figure 2-source data 2/2E/1h/p-p38/p-p38 merge==.Tif]

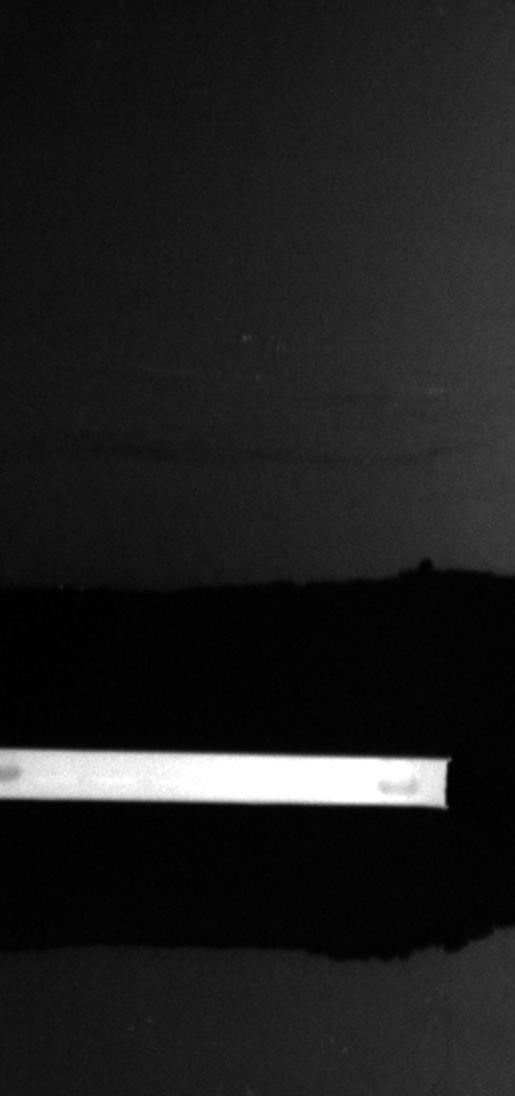

Supplement: Figure 2—source data 2. [file elife-104060-fig2-data2.zip › Figure 2-source data 2/2E/1h/p-p38/p-p38 white==.Tif]

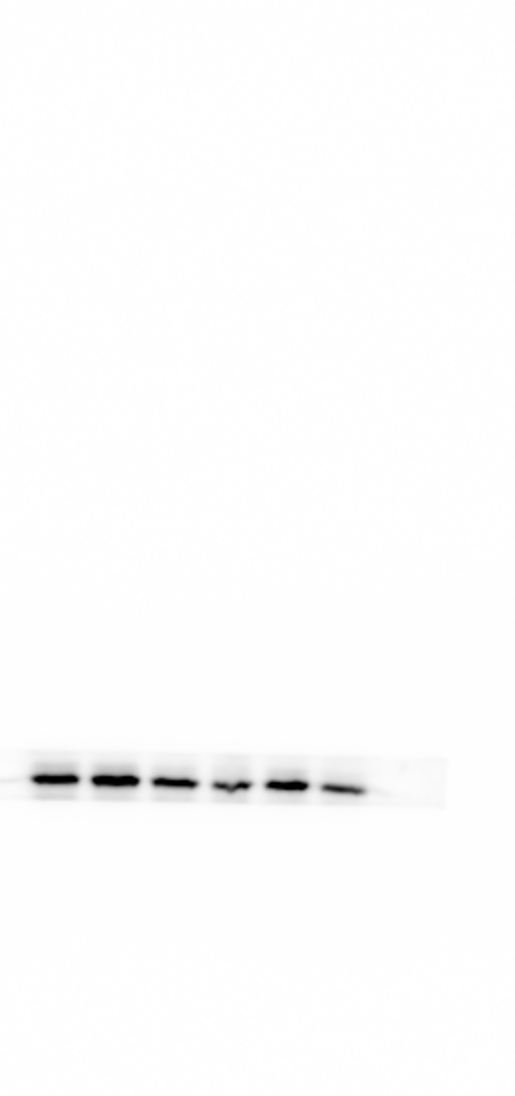

Supplement: Figure 2—source data 2. [file elife-104060-fig2-data2.zip › Figure 2-source data 2/2E/1h/p-p38/p-p38==.Tif]

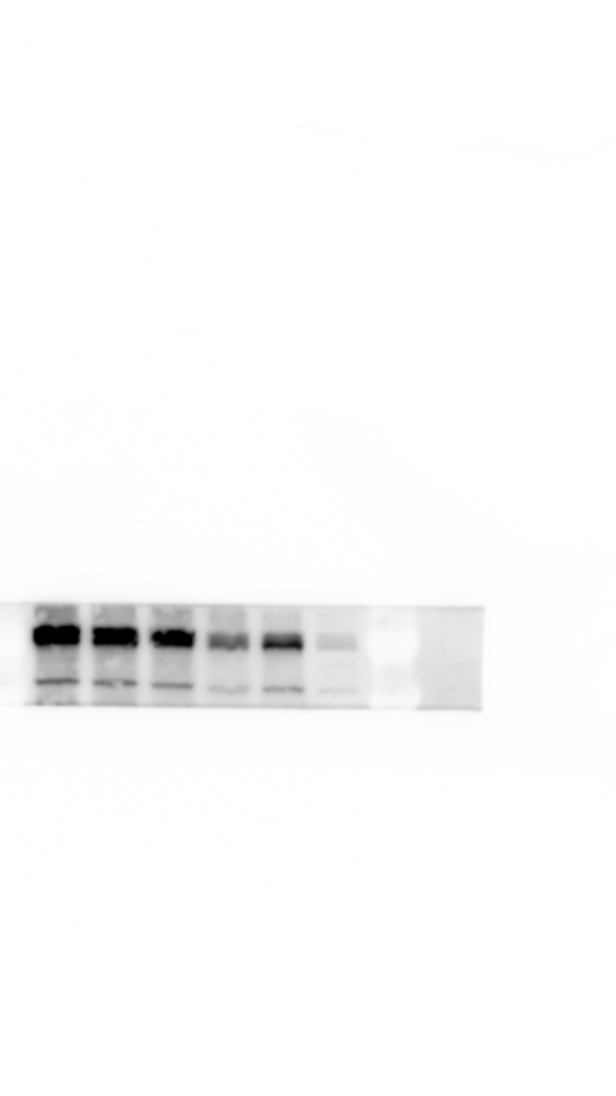

Supplement: Figure 2—source data 2. [file elife-104060-fig2-data2.zip › Figure 2-source data 2/2E/1h/p-SHP2/1 pshp2 merge.Tif]

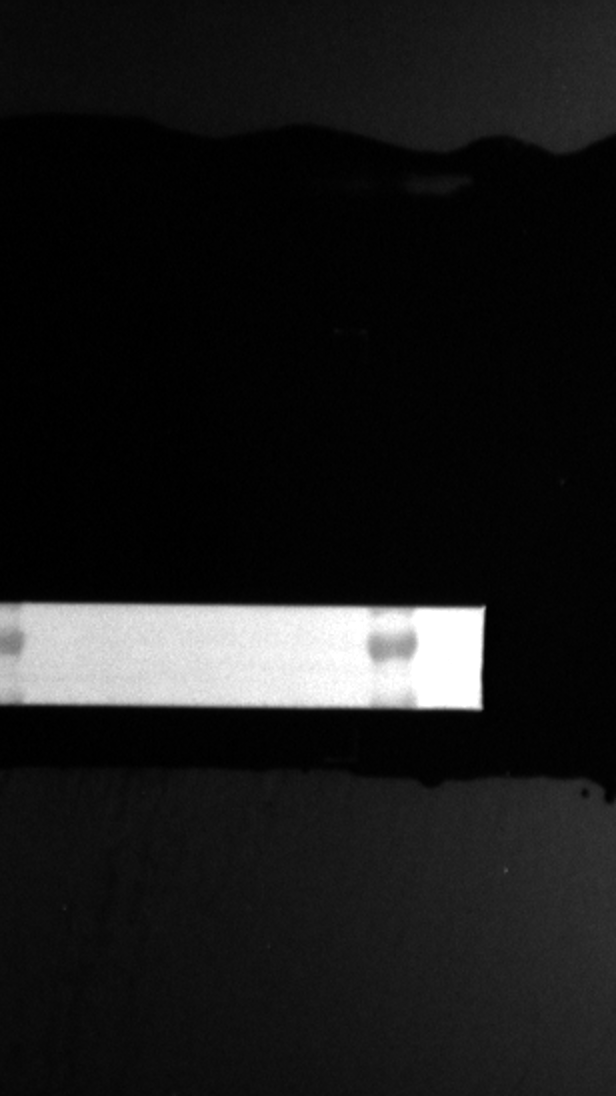

Supplement: Figure 2—source data 2. [file elife-104060-fig2-data2.zip › Figure 2-source data 2/2E/1h/p-SHP2/1 pshp2 white.Tif]

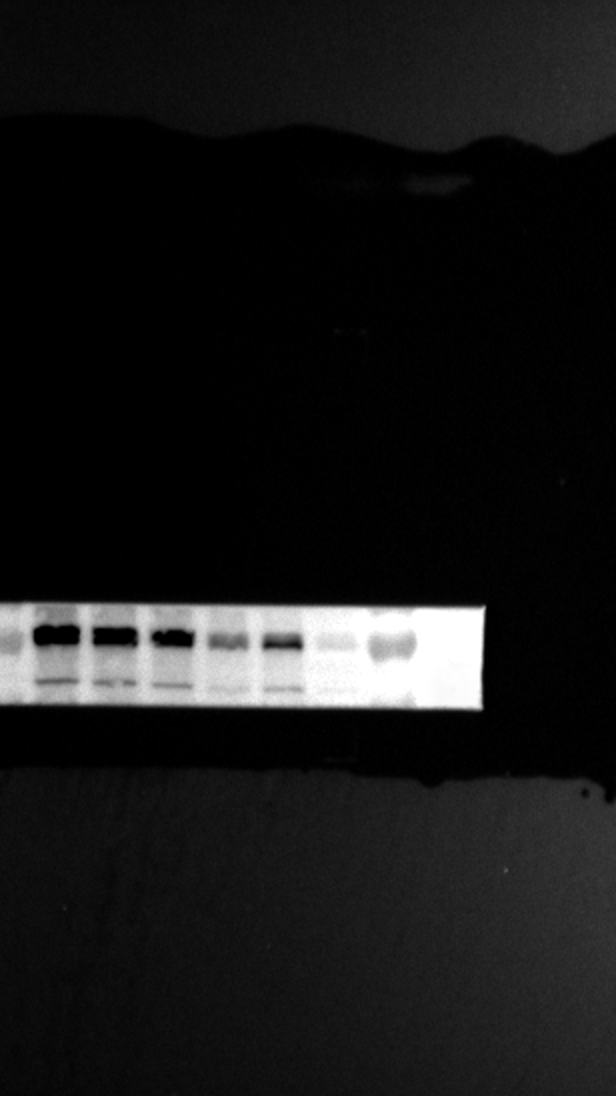

Supplement: Figure 2—source data 2. [file elife-104060-fig2-data2.zip › Figure 2-source data 2/2E/1h/p-SHP2/1 pshp2.Tif]

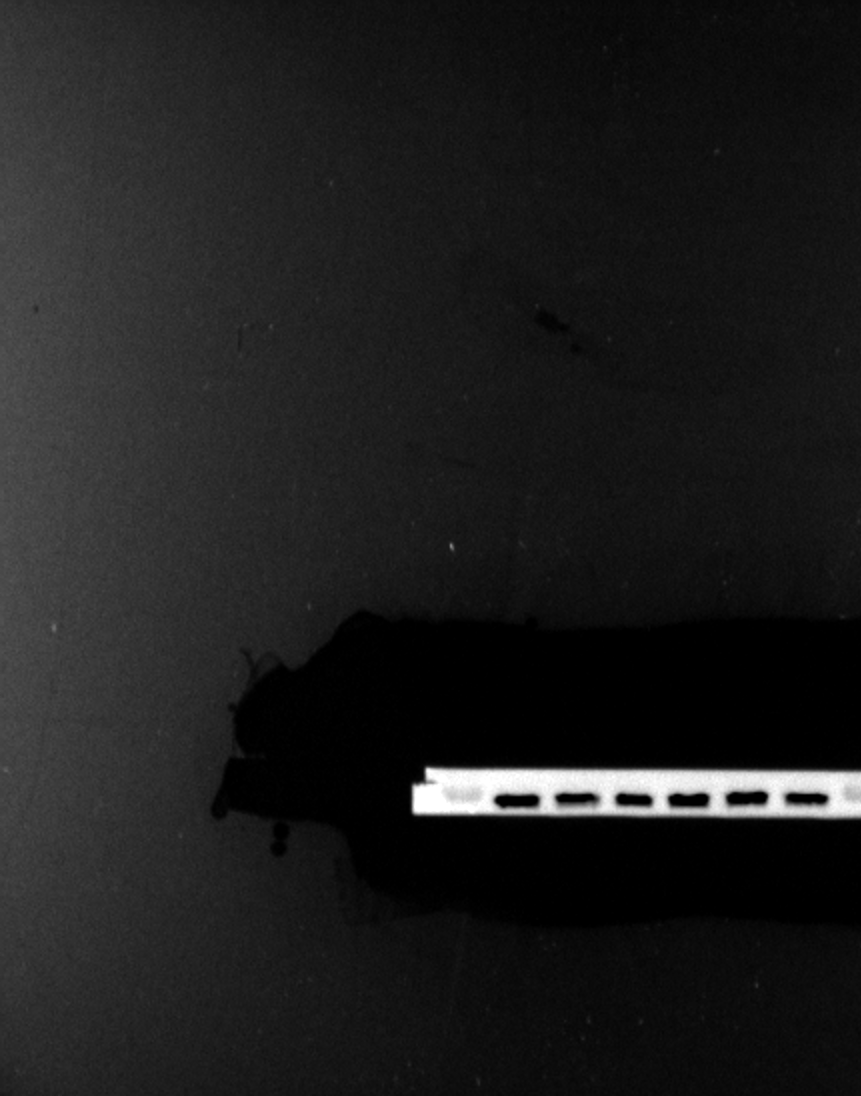

Supplement: Figure 2—source data 2. [file elife-104060-fig2-data2.zip › Figure 2-source data 2/2E/1h/p38/p38 merge.Tif]

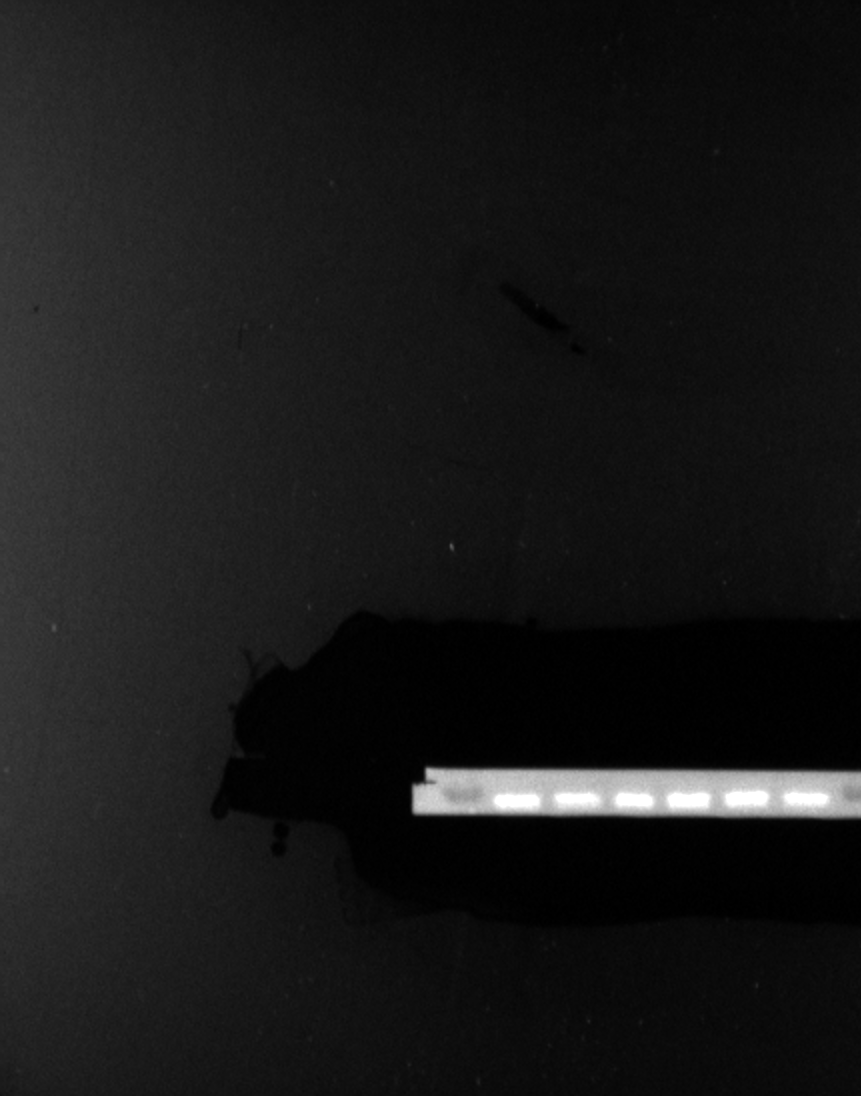

Supplement: Figure 2—source data 2. [file elife-104060-fig2-data2.zip › Figure 2-source data 2/2E/1h/p38/p38 white.Tif]

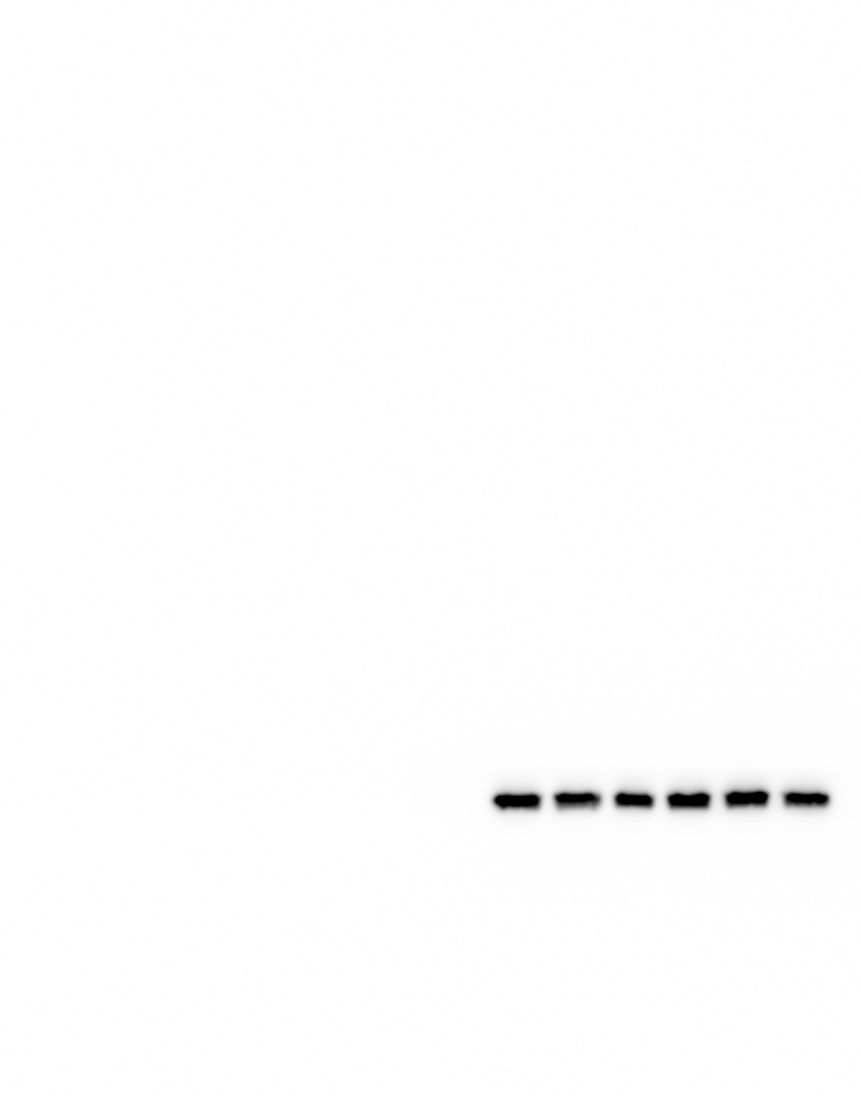

Supplement: Figure 2—source data 2. [file elife-104060-fig2-data2.zip › Figure 2-source data 2/2E/1h/p38/p38.Tif]

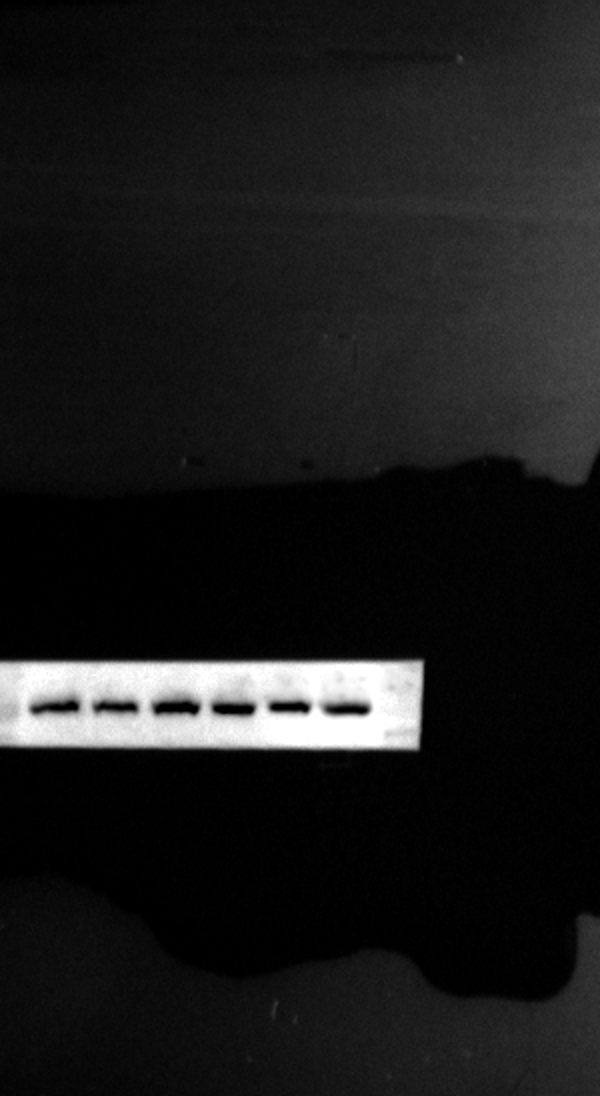

Supplement: Figure 2—source data 2. [file elife-104060-fig2-data2.zip › Figure 2-source data 2/2E/1h/SHP2/1 shp2 merge.Tif]

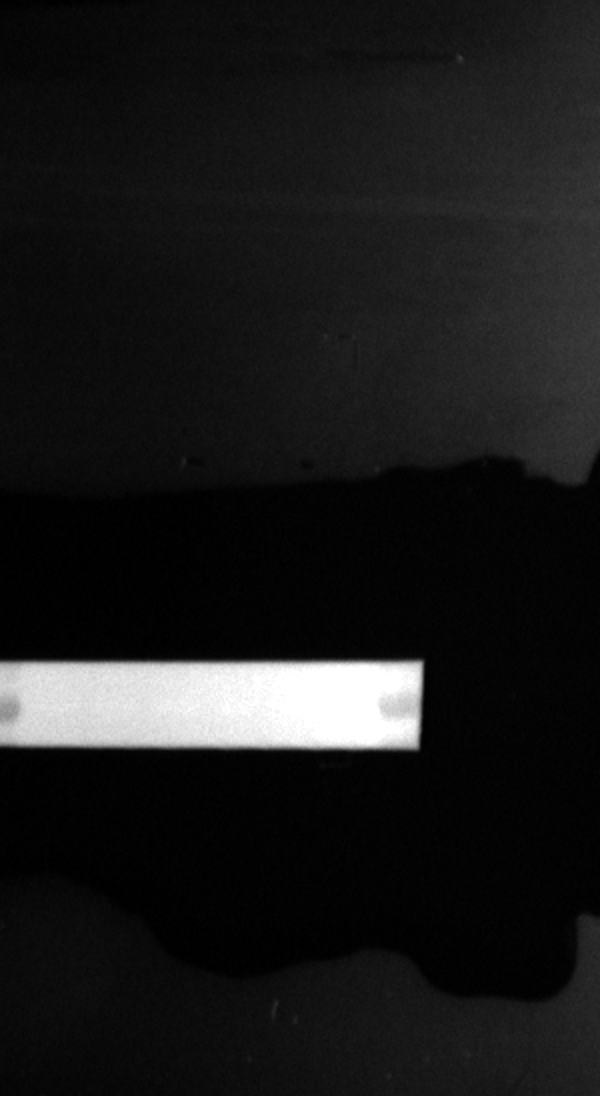

Supplement: Figure 2—source data 2. [file elife-104060-fig2-data2.zip › Figure 2-source data 2/2E/1h/SHP2/1 shp2 white.Tif]

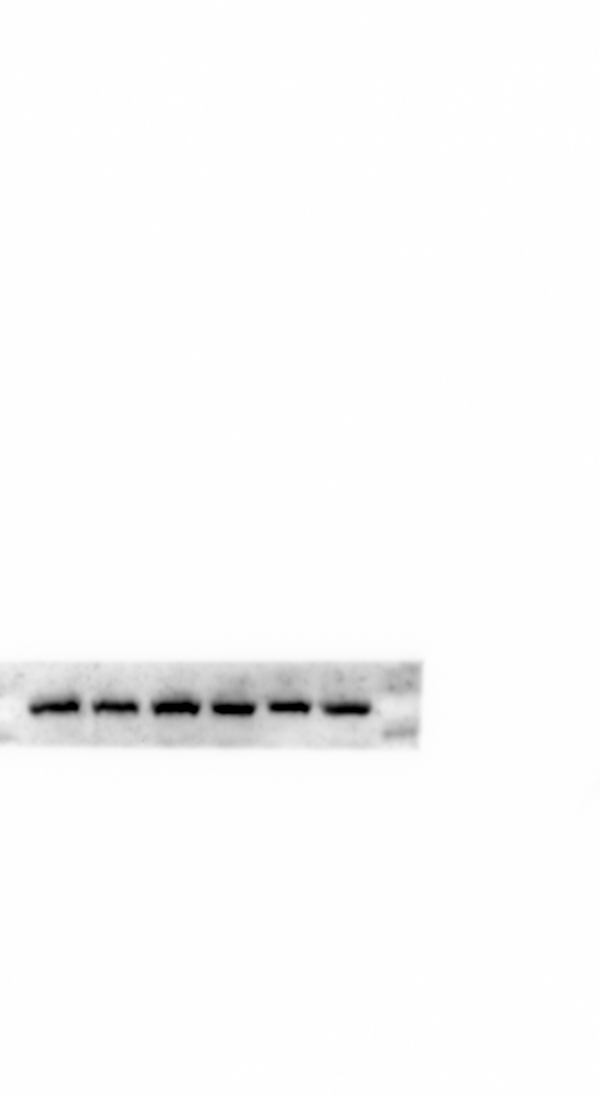

Supplement: Figure 2—source data 2. [file elife-104060-fig2-data2.zip › Figure 2-source data 2/2E/1h/SHP2/1 shp2.Tif]

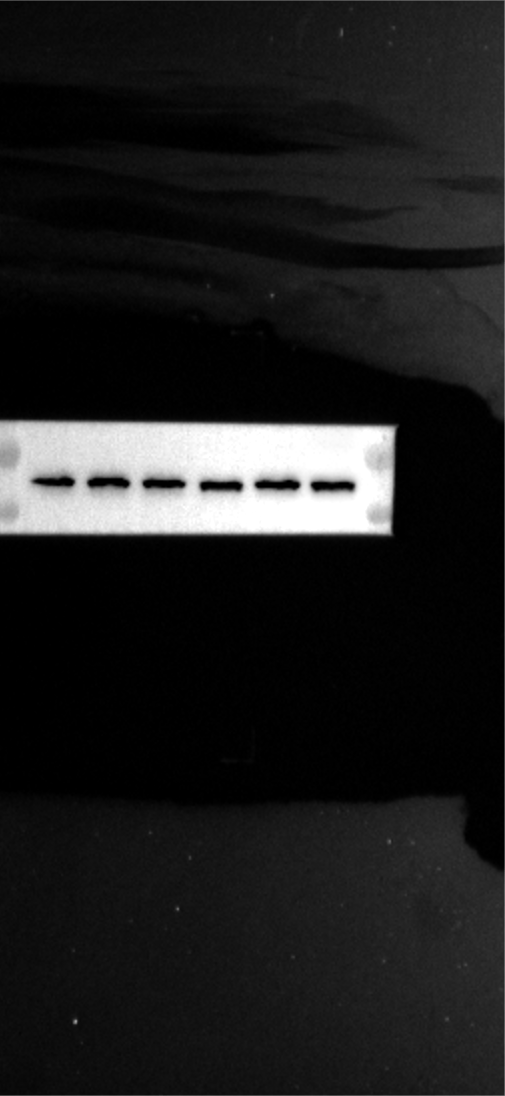

Supplement: Figure 2—source data 2. [file elife-104060-fig2-data2.zip › Figure 2-source data 2/2E/48h/AKT/1 akt merge=.png]

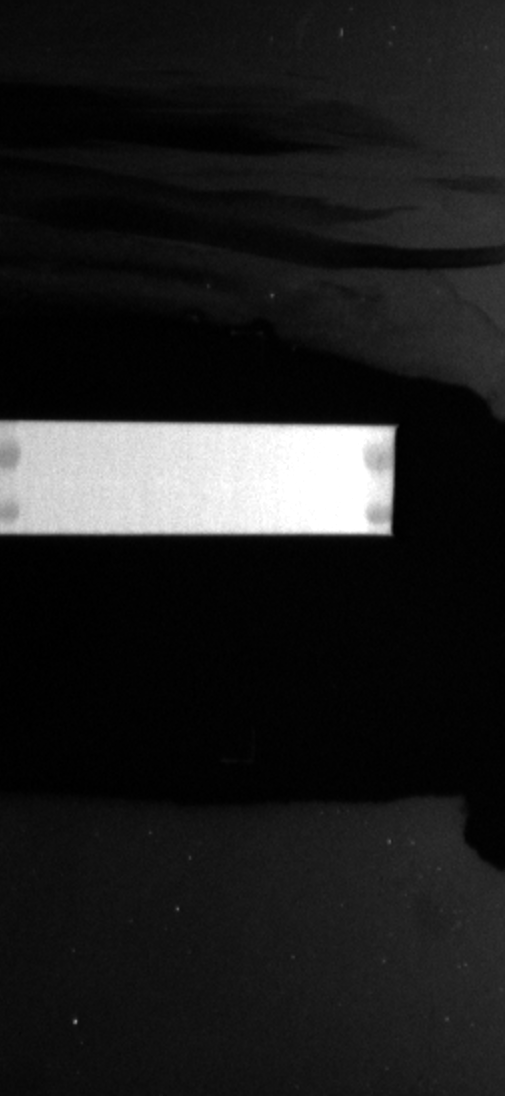

Supplement: Figure 2—source data 2. [file elife-104060-fig2-data2.zip › Figure 2-source data 2/2E/48h/AKT/1 akt white=.Tif]

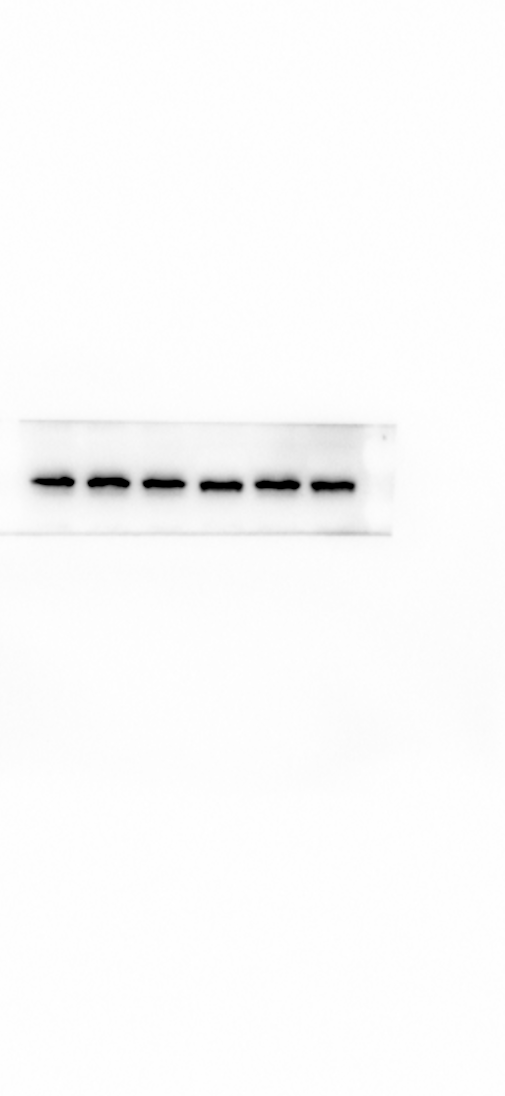

Supplement: Figure 2—source data 2. [file elife-104060-fig2-data2.zip › Figure 2-source data 2/2E/48h/AKT/1 akt=.Tif]

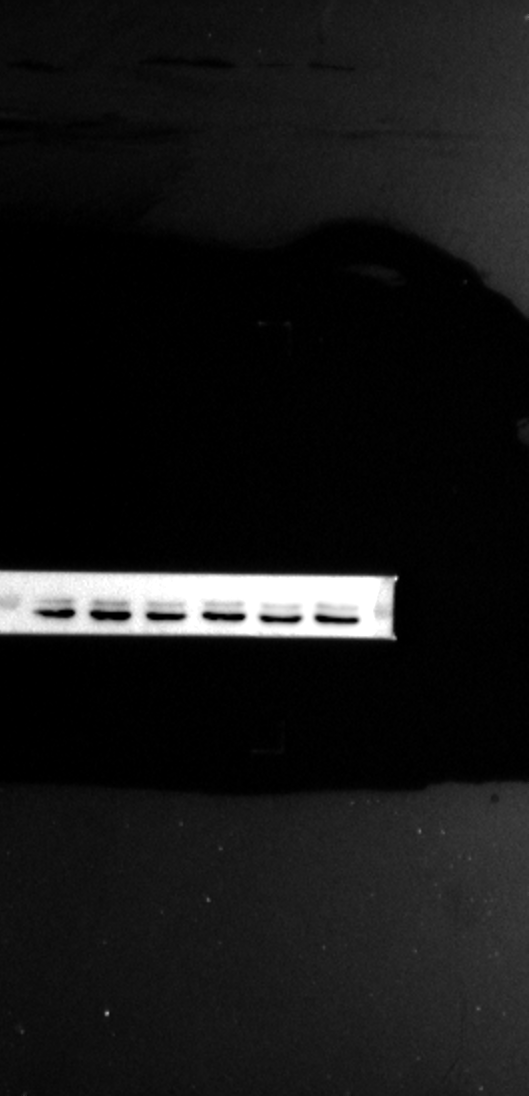

Supplement: Figure 2—source data 2. [file elife-104060-fig2-data2.zip › Figure 2-source data 2/2E/48h/ERK/1 erk merge=.Tif]

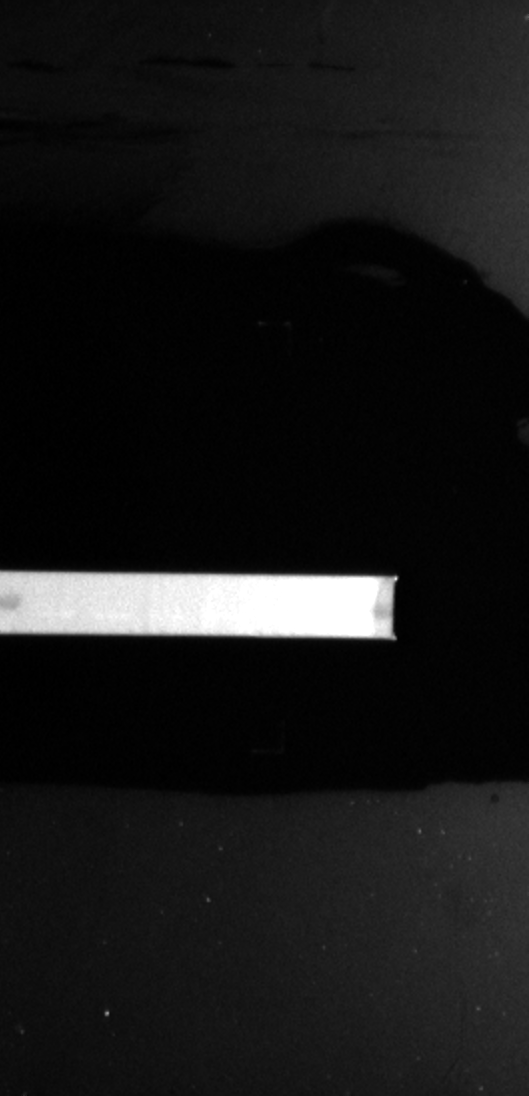

Supplement: Figure 2—source data 2. [file elife-104060-fig2-data2.zip › Figure 2-source data 2/2E/48h/ERK/1 erk white=.Tif]

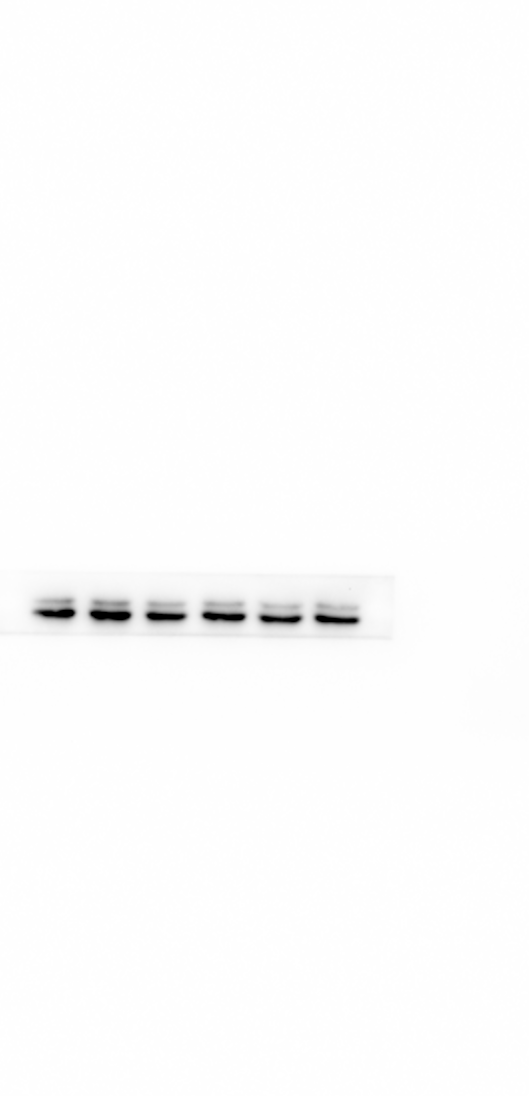

Supplement: Figure 2—source data 2. [file elife-104060-fig2-data2.zip › Figure 2-source data 2/2E/48h/ERK/1 erk=.Tif]

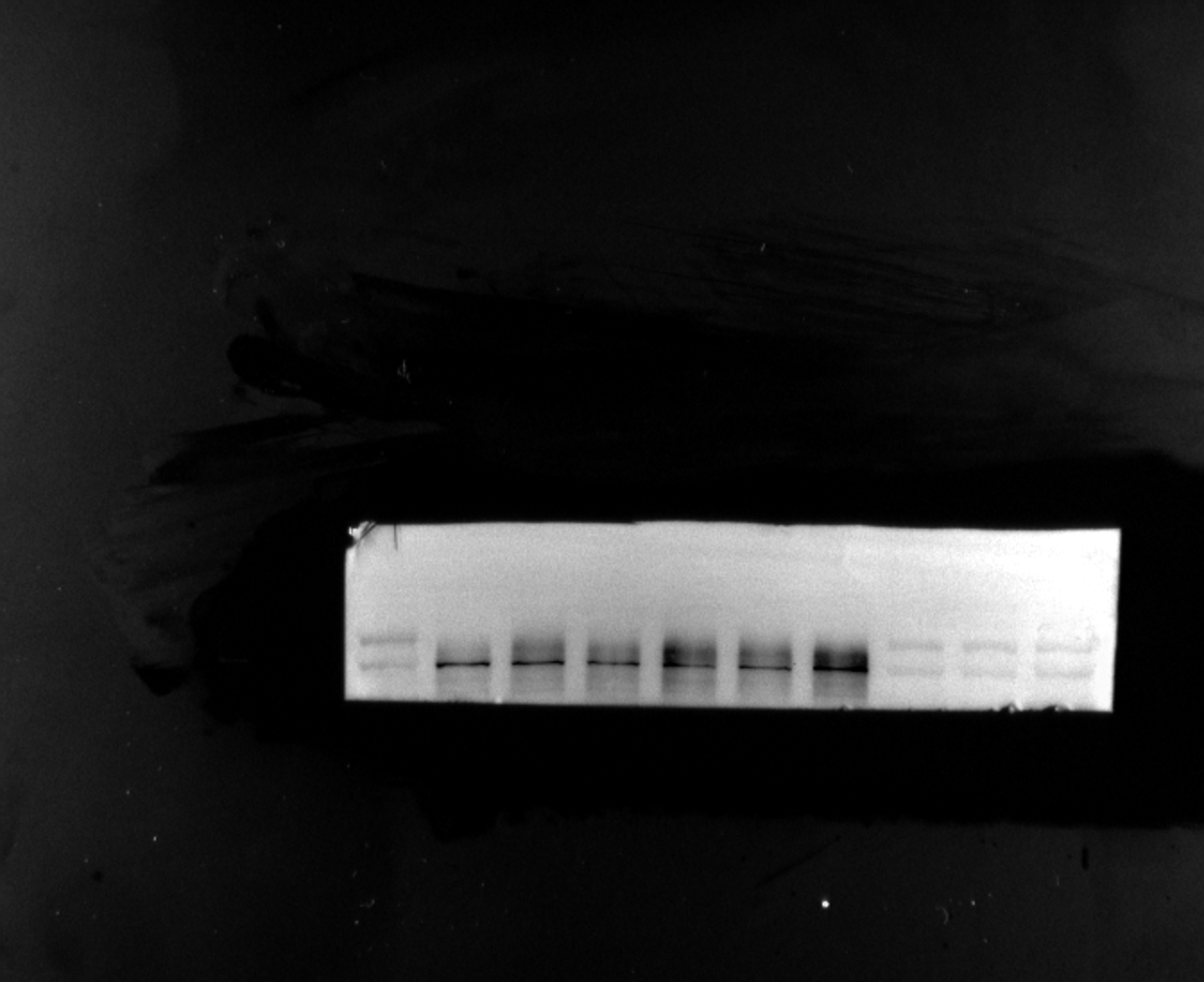

Supplement: Figure 2—source data 2. [file elife-104060-fig2-data2.zip › Figure 2-source data 2/2E/48h/FGFR2/1 fgfr2 merge.Tif]

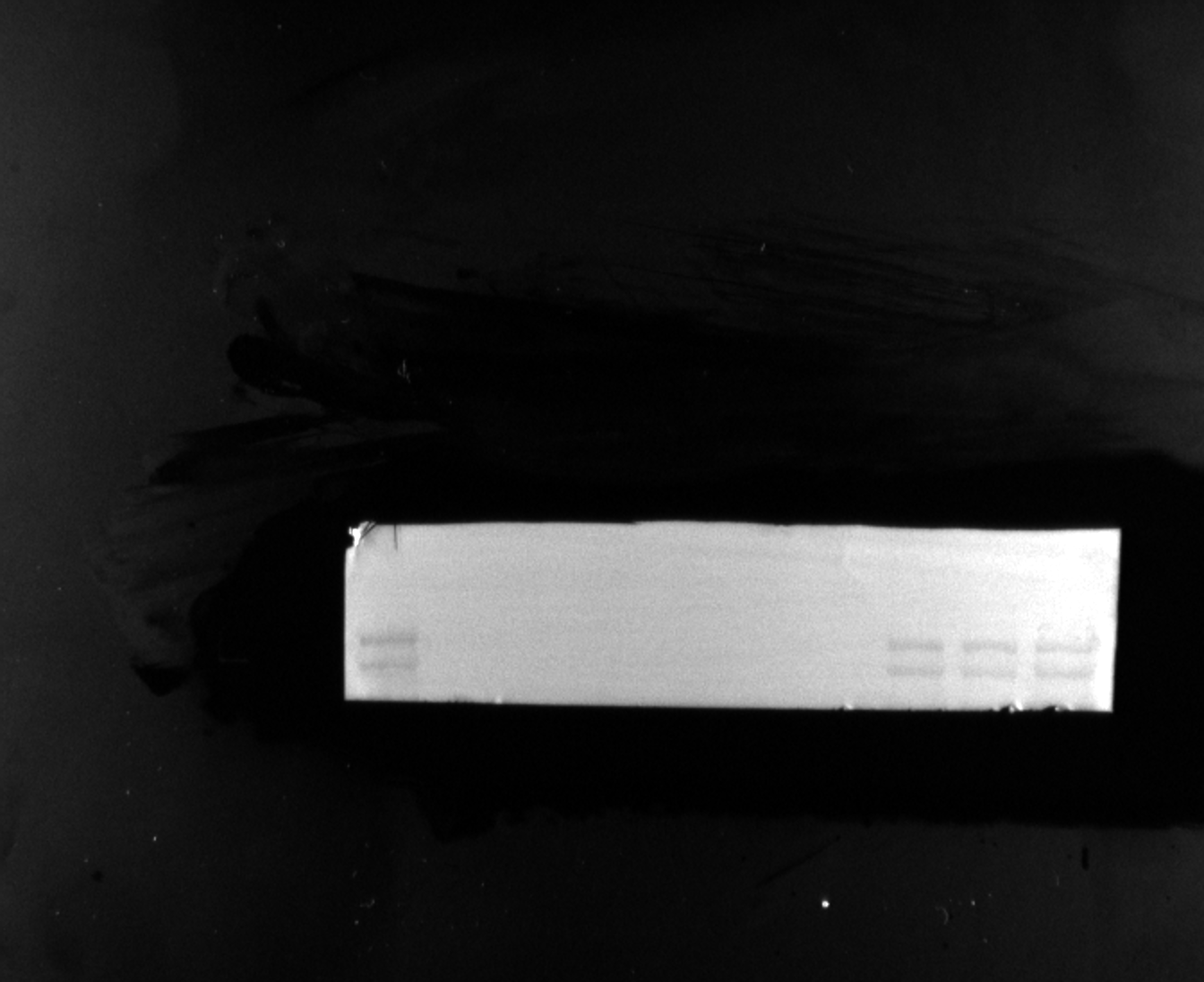

Supplement: Figure 2—source data 2. [file elife-104060-fig2-data2.zip › Figure 2-source data 2/2E/48h/FGFR2/1 fgfr2 white.Tif]

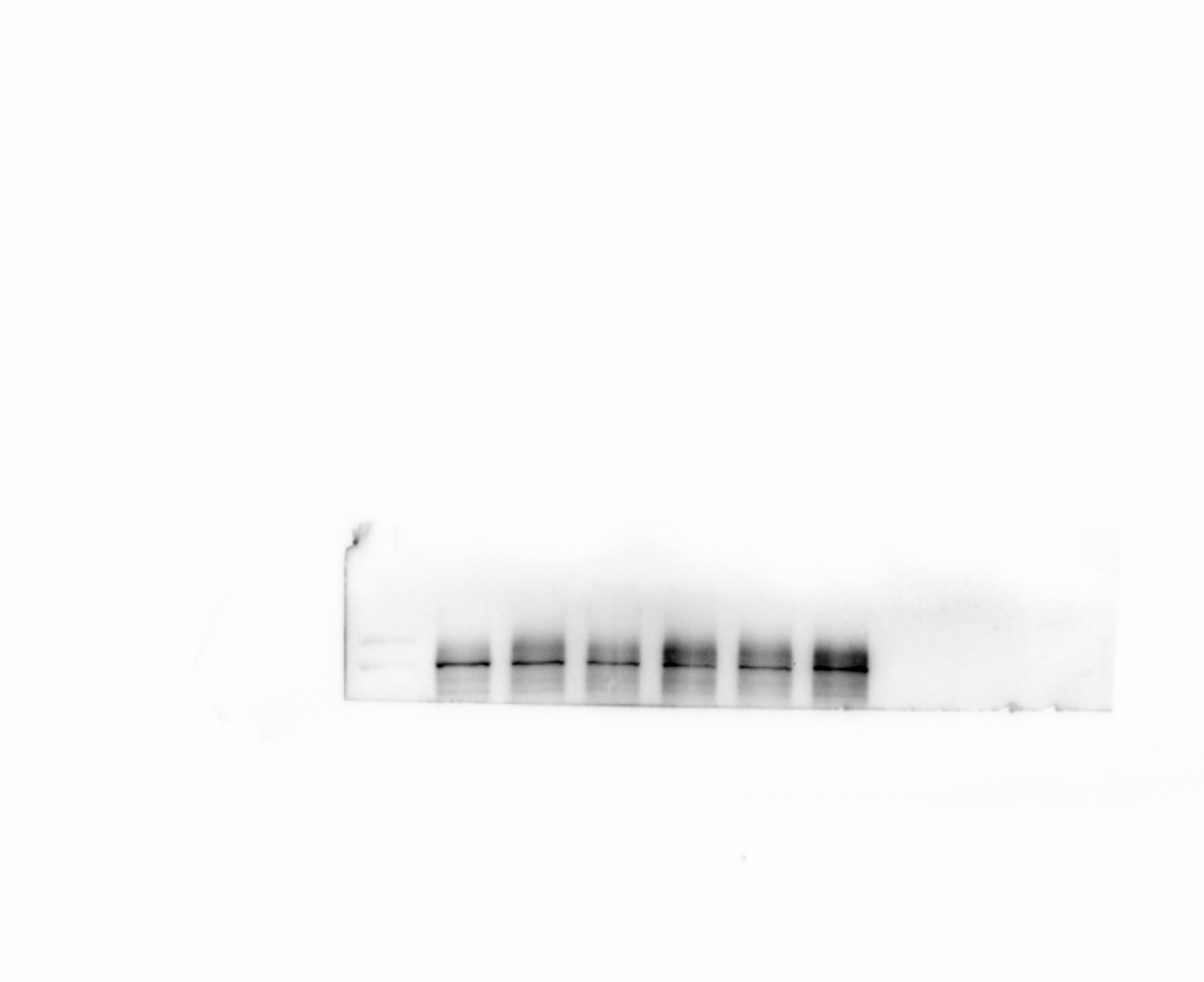

Supplement: Figure 2—source data 2. [file elife-104060-fig2-data2.zip › Figure 2-source data 2/2E/48h/FGFR2/1 fgfr2.Tif]

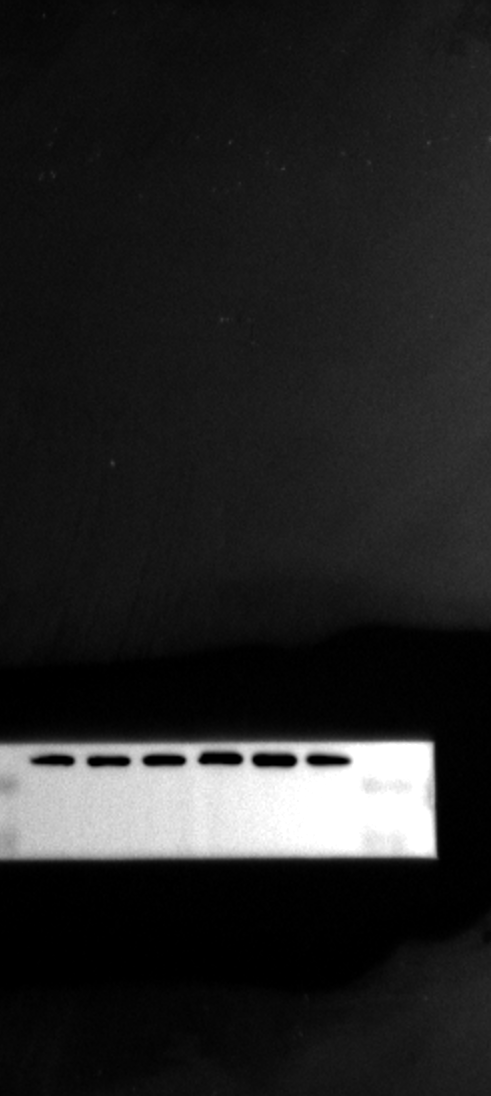

Supplement: Figure 2—source data 2. [file elife-104060-fig2-data2.zip › Figure 2-source data 2/2E/48h/GAPDH/1 gap merge=.Tif]

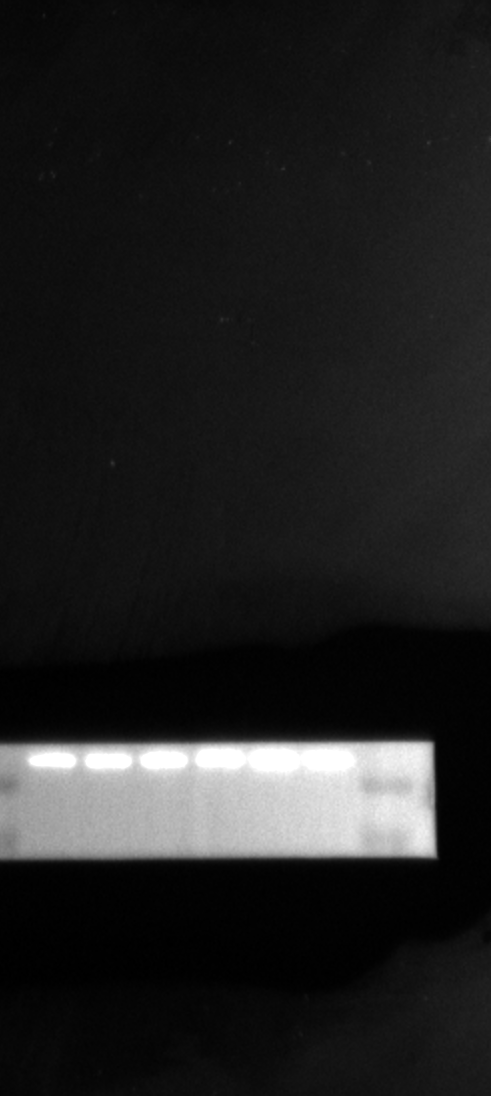

Supplement: Figure 2—source data 2. [file elife-104060-fig2-data2.zip › Figure 2-source data 2/2E/48h/GAPDH/1 gap white=.Tif]

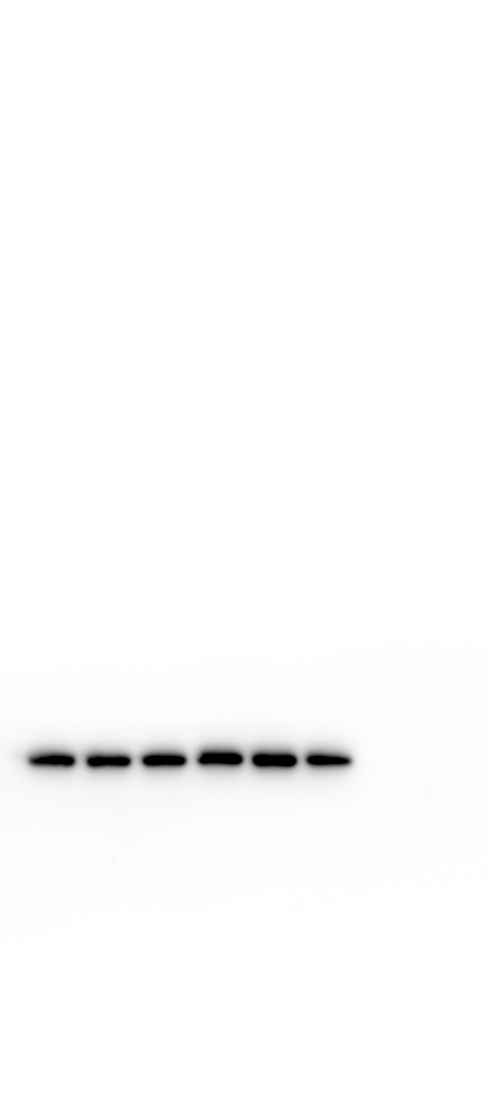

Supplement: Figure 2—source data 2. [file elife-104060-fig2-data2.zip › Figure 2-source data 2/2E/48h/GAPDH/1 gap=.Tif]

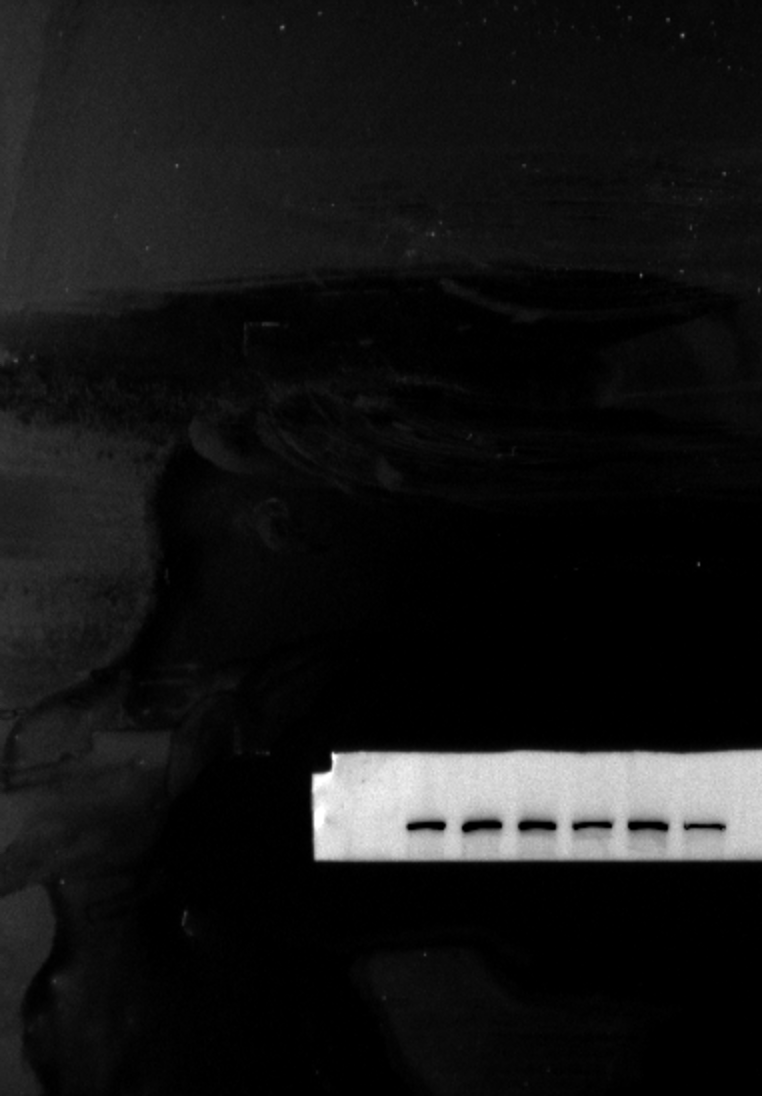

Supplement: Figure 2—source data 2. [file elife-104060-fig2-data2.zip › Figure 2-source data 2/2E/48h/mTOR/2-mtor merge=.Tif]

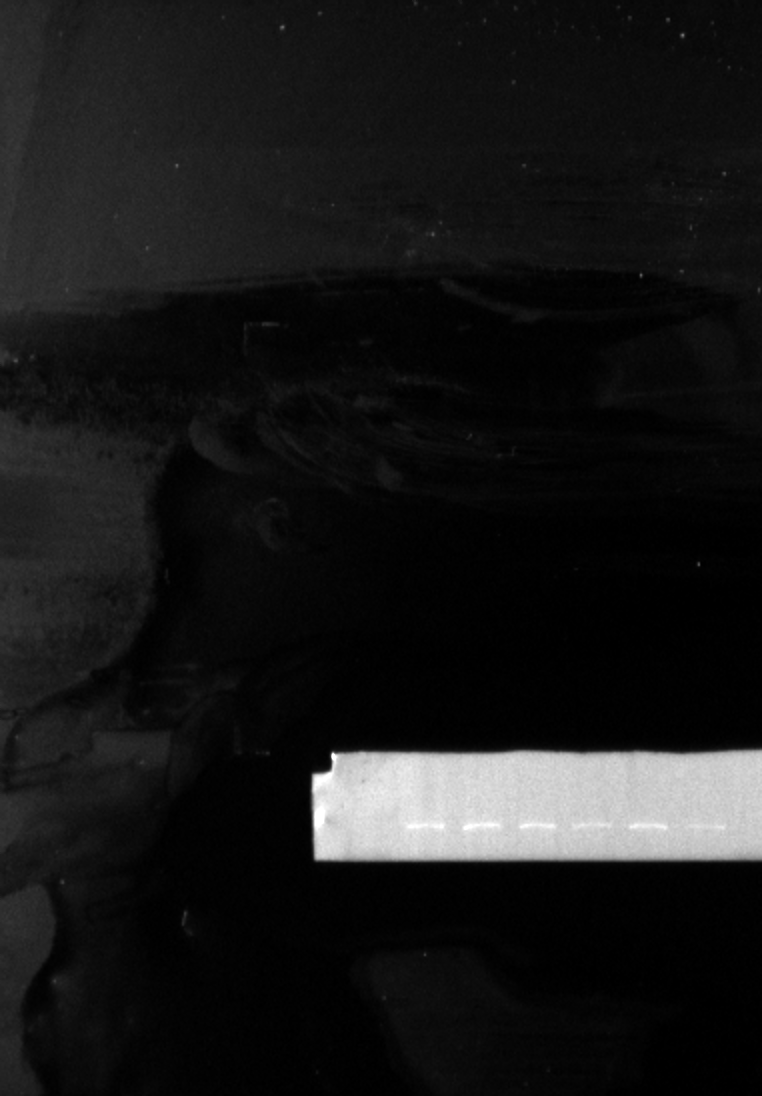

Supplement: Figure 2—source data 2. [file elife-104060-fig2-data2.zip › Figure 2-source data 2/2E/48h/mTOR/2-mtor white=.Tif]

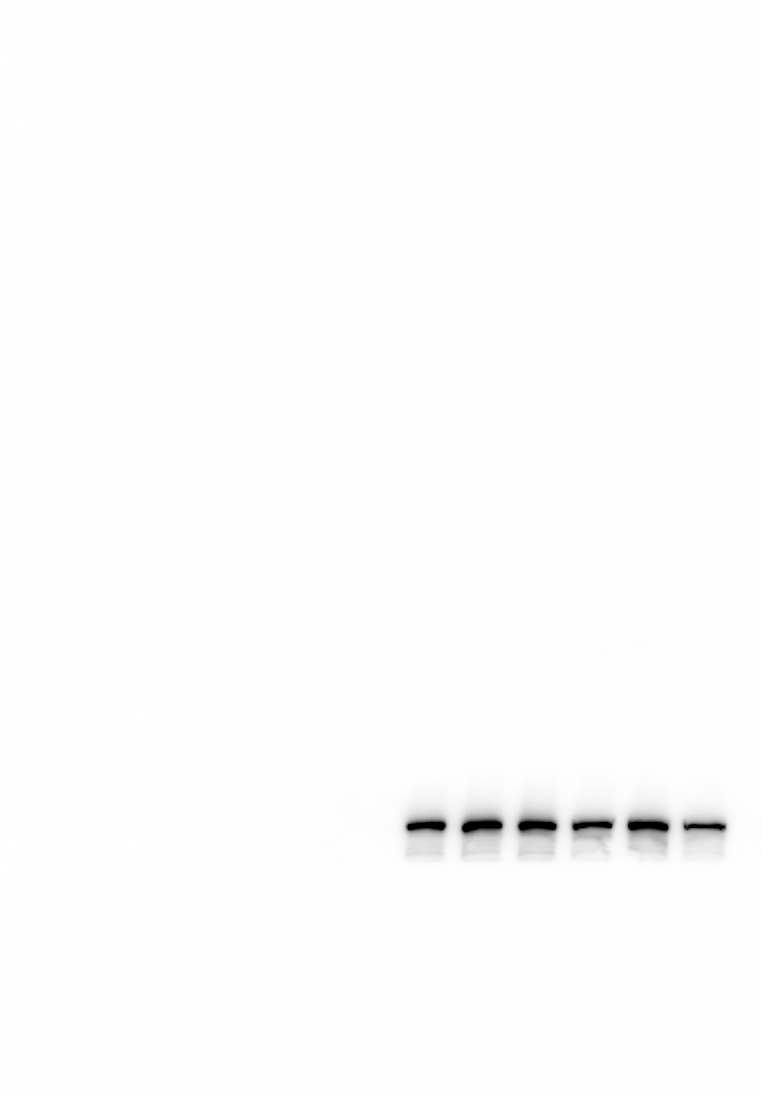

Supplement: Figure 2—source data 2. [file elife-104060-fig2-data2.zip › Figure 2-source data 2/2E/48h/mTOR/2-mtor=.Tif]

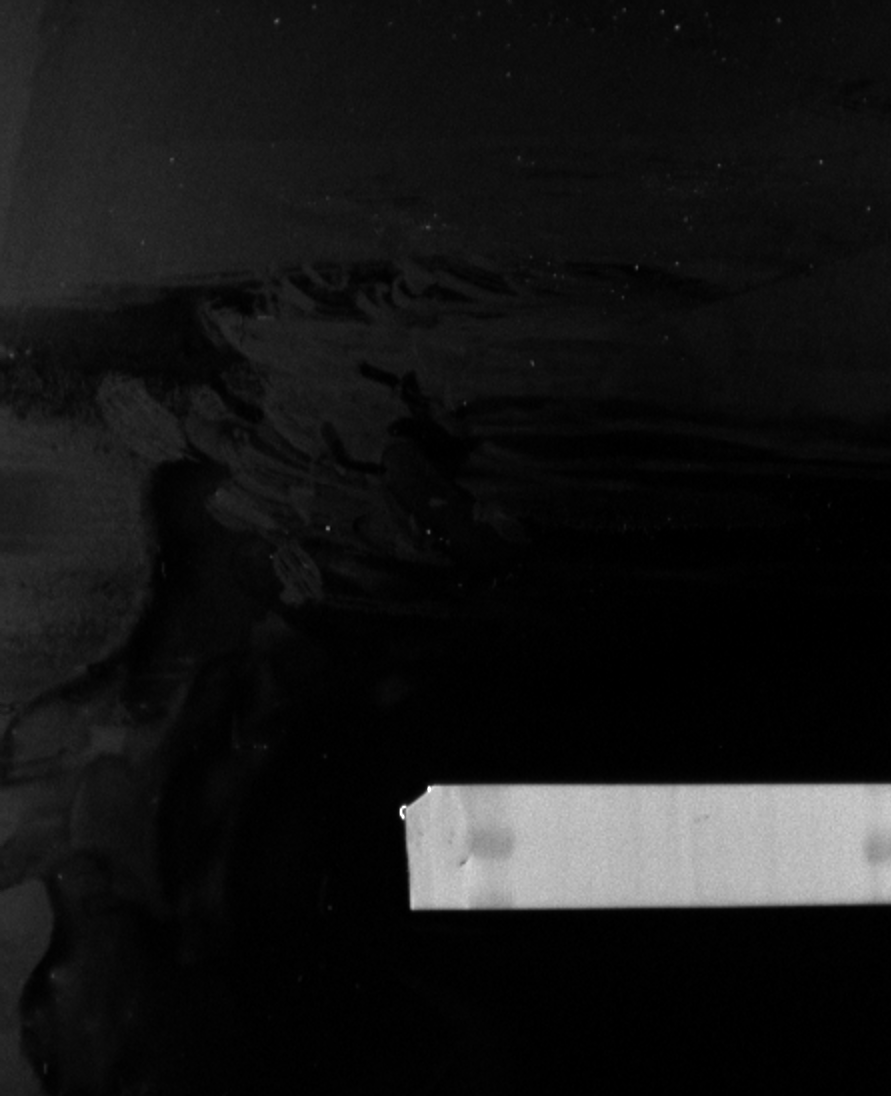

Supplement: Figure 2—source data 2. [file elife-104060-fig2-data2.zip › Figure 2-source data 2/2E/48h/p-AKT/p akt=.Tif]

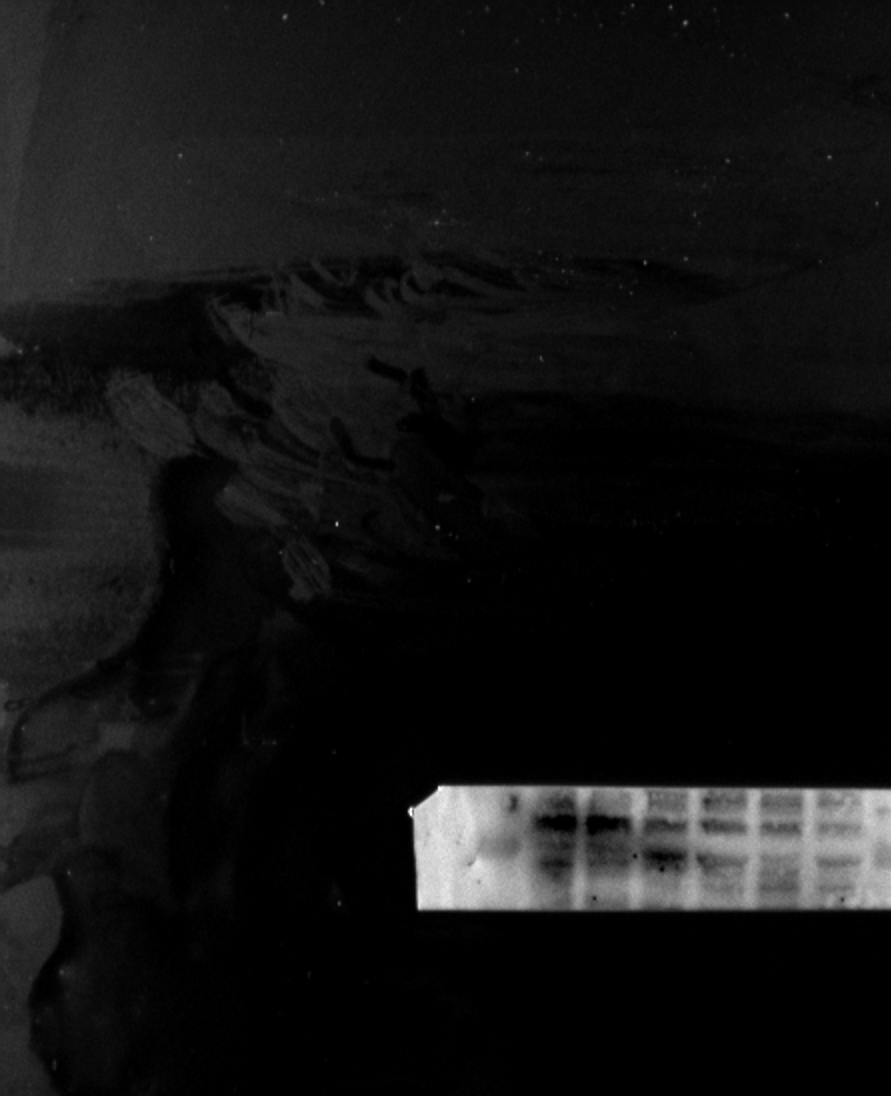

Supplement: Figure 2—source data 2. [file elife-104060-fig2-data2.zip › Figure 2-source data 2/2E/48h/p-AKT/p akt==-.Tif]

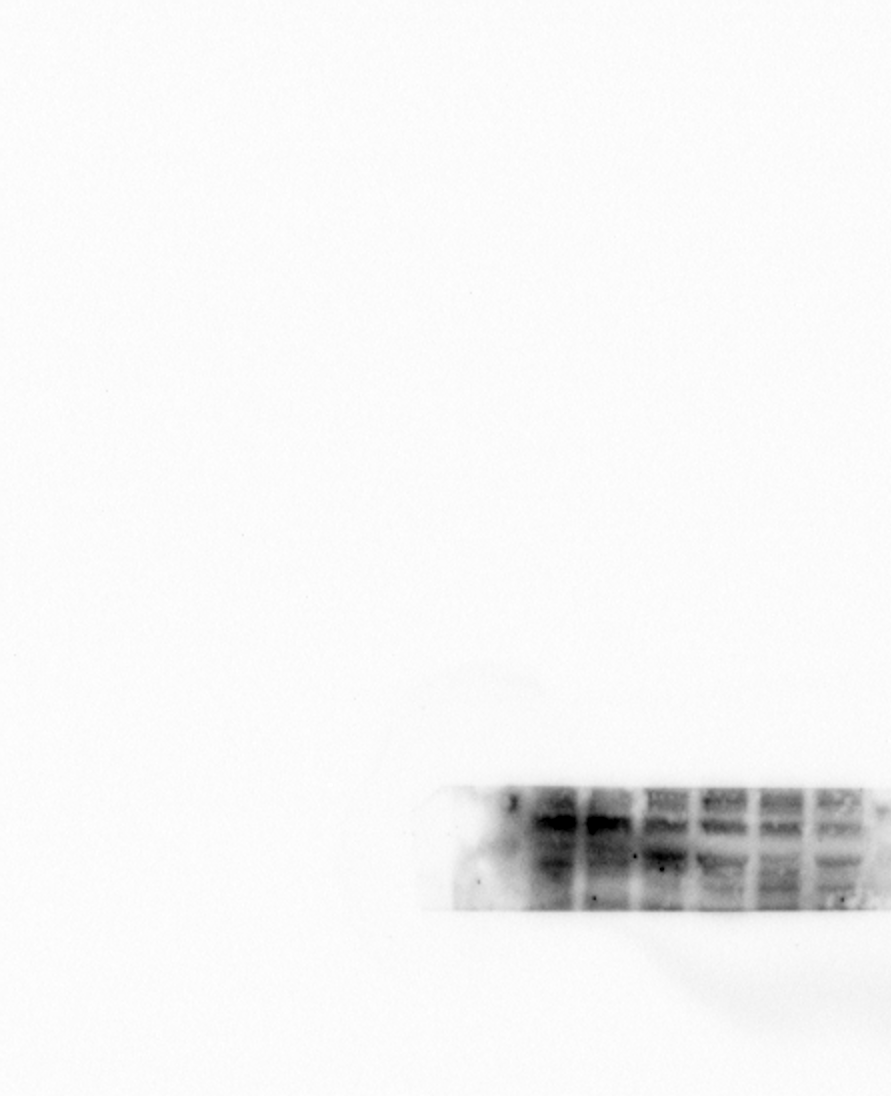

Supplement: Figure 2—source data 2. [file elife-104060-fig2-data2.zip › Figure 2-source data 2/2E/48h/p-AKT/p akt===.Tif]

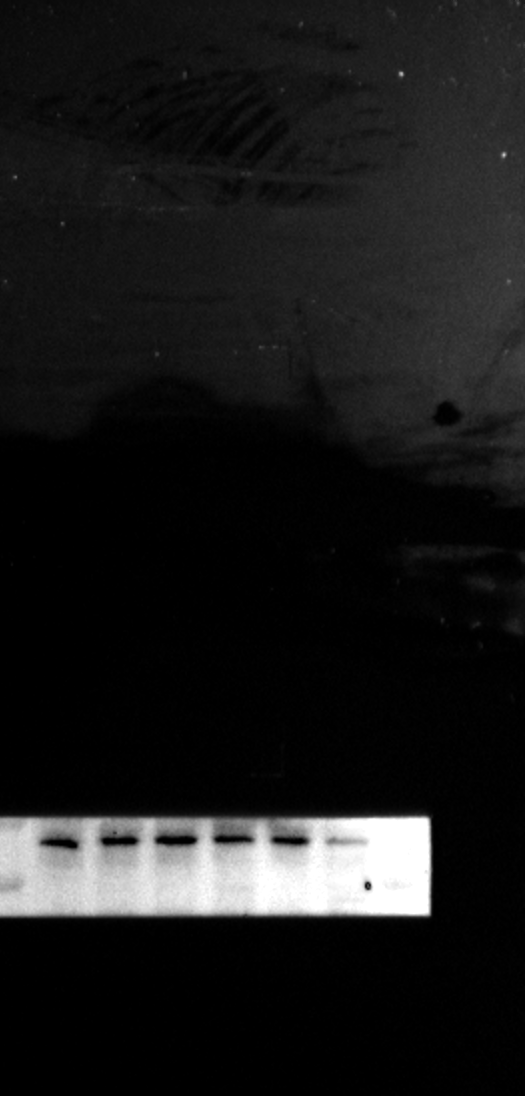

Supplement: Figure 2—source data 2. [file elife-104060-fig2-data2.zip › Figure 2-source data 2/2E/48h/p-ERK/1 perk merge.Tif]

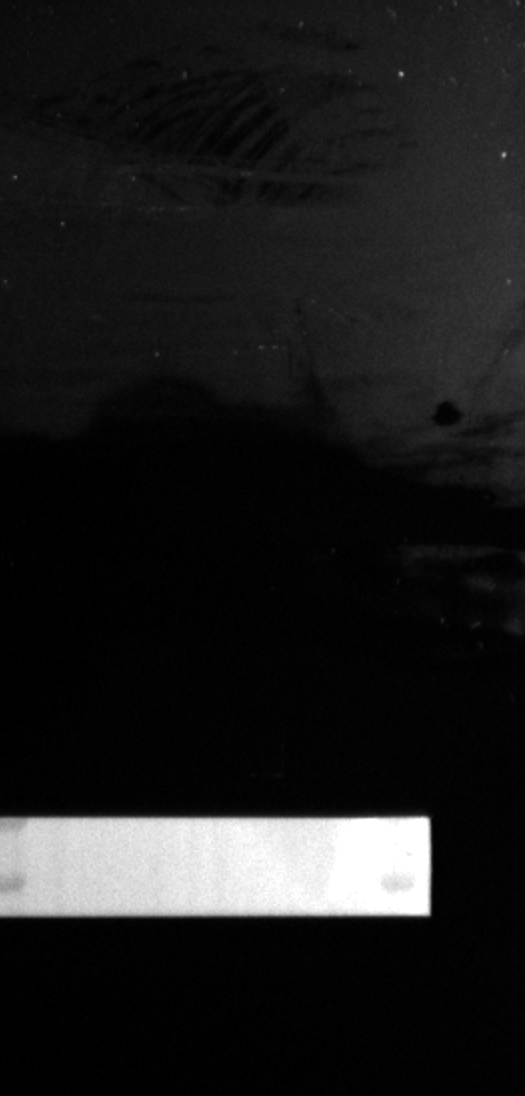

Supplement: Figure 2—source data 2. [file elife-104060-fig2-data2.zip › Figure 2-source data 2/2E/48h/p-ERK/1 perk white=.Tif]

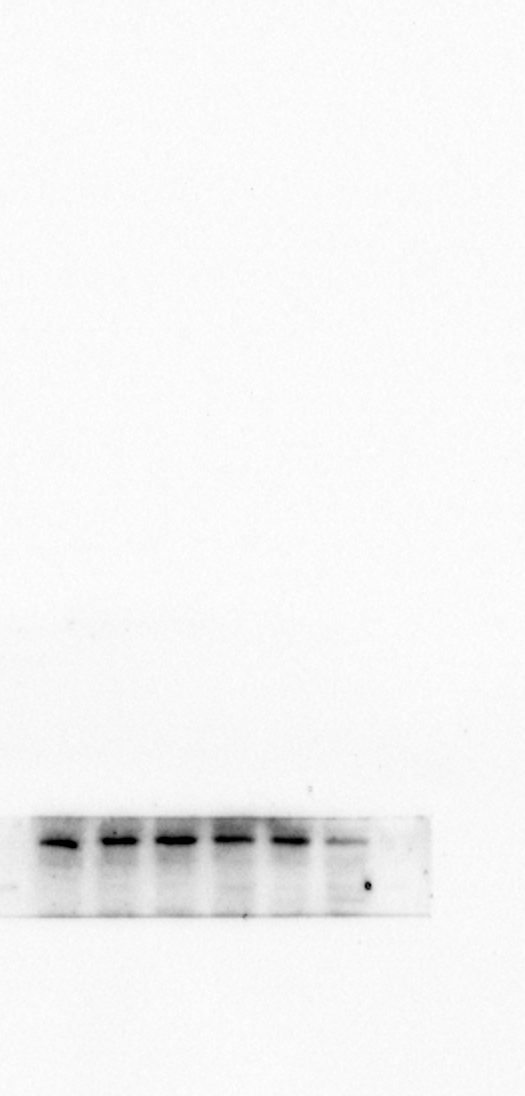

Supplement: Figure 2—source data 2. [file elife-104060-fig2-data2.zip › Figure 2-source data 2/2E/48h/p-ERK/1 perk=.Tif]

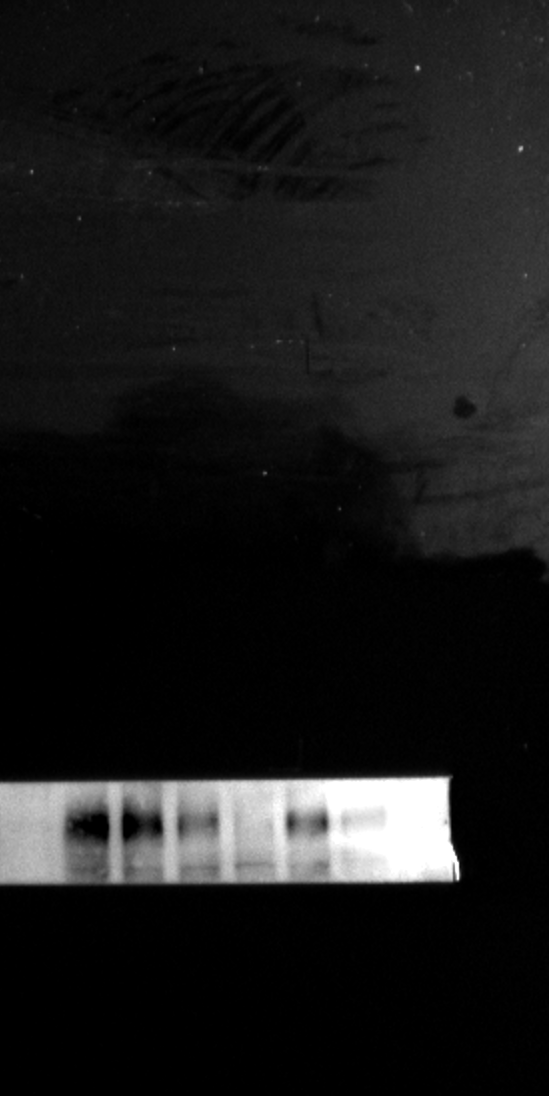

Supplement: Figure 2—source data 2. [file elife-104060-fig2-data2.zip › Figure 2-source data 2/2E/48h/p-FGFR/1 pfgfr merge.Tif]

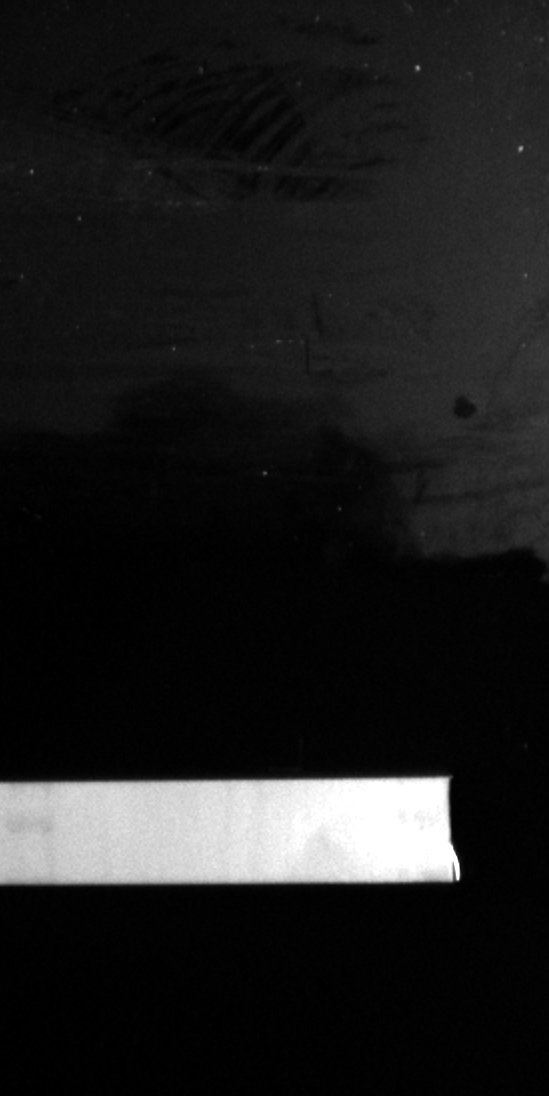

Supplement: Figure 2—source data 2. [file elife-104060-fig2-data2.zip › Figure 2-source data 2/2E/48h/p-FGFR/1 pfgfr white.Tif]

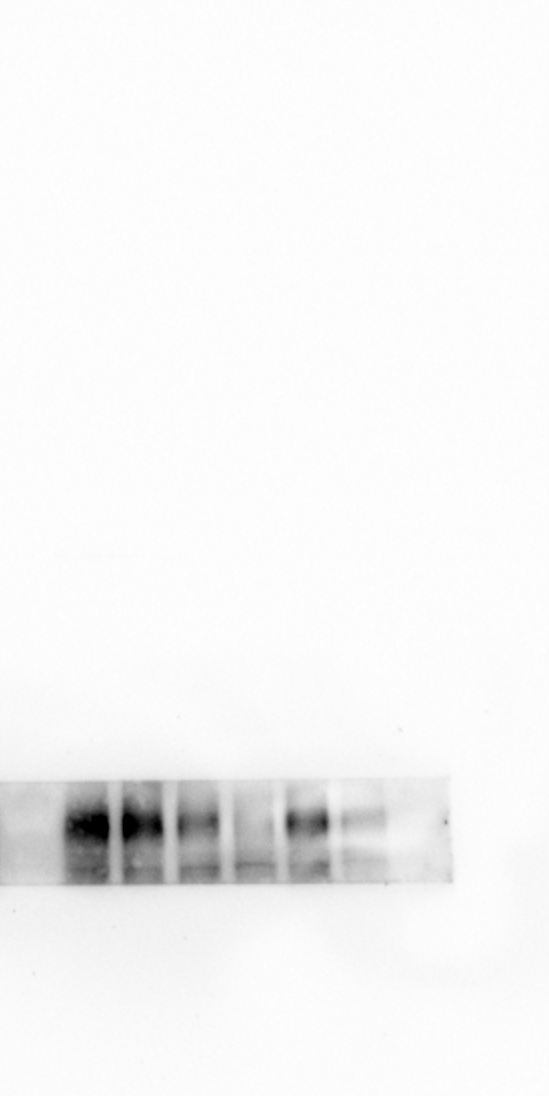

Supplement: Figure 2—source data 2. [file elife-104060-fig2-data2.zip › Figure 2-source data 2/2E/48h/p-FGFR/1 pfgfr.png]

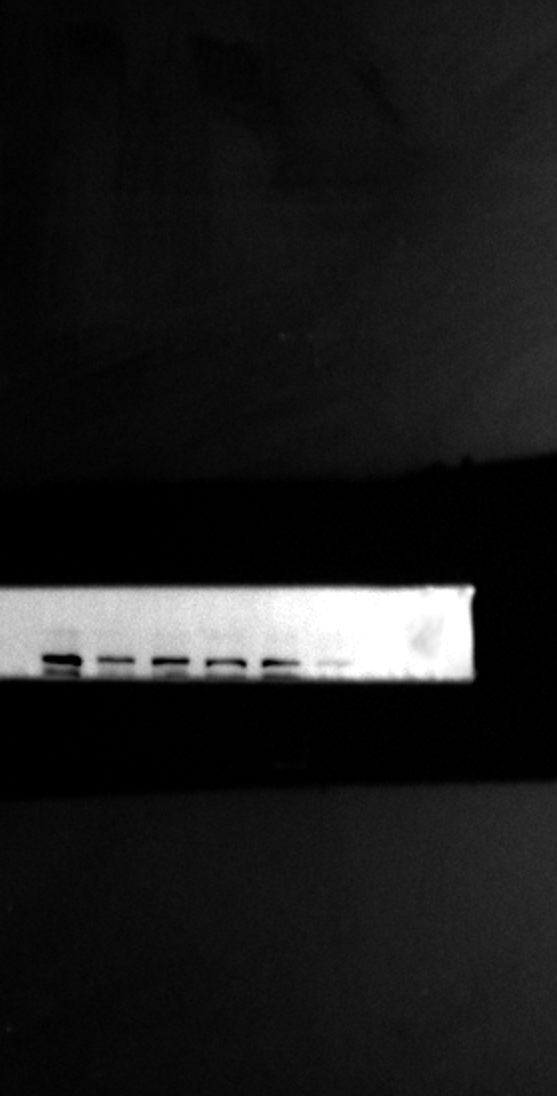

Supplement: Figure 2—source data 2. [file elife-104060-fig2-data2.zip › Figure 2-source data 2/2E/48h/p-mTOR/1-PMTOR MERGE.Tif]

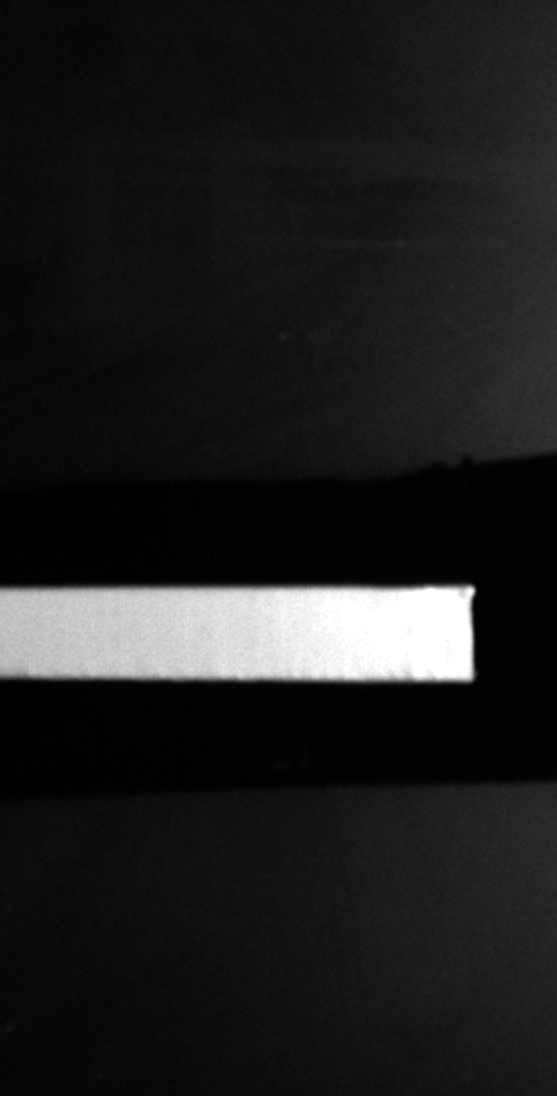

Supplement: Figure 2—source data 2. [file elife-104060-fig2-data2.zip › Figure 2-source data 2/2E/48h/p-mTOR/1-PMTOR WHITE.Tif]

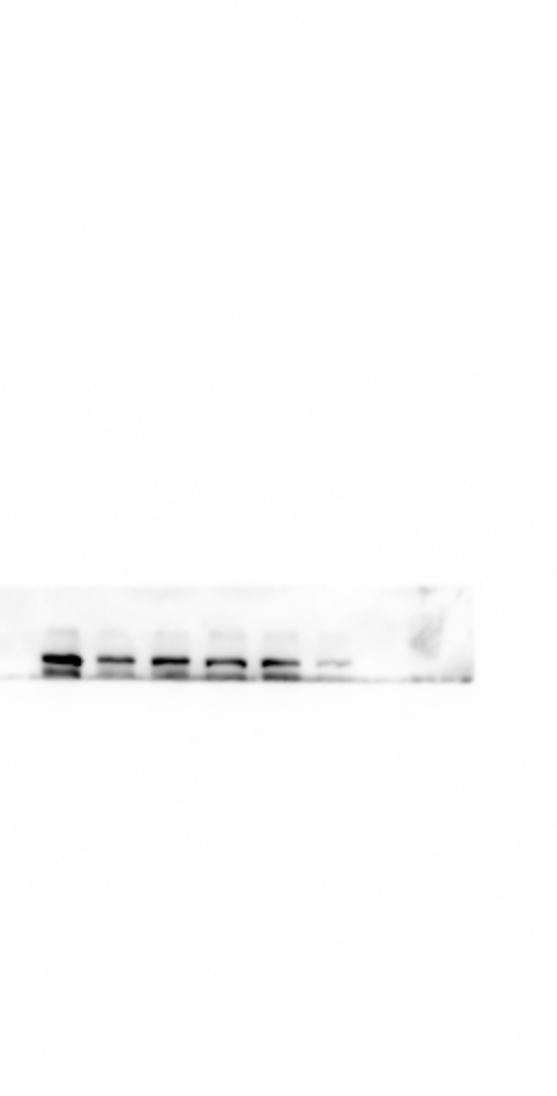

Supplement: Figure 2—source data 2. [file elife-104060-fig2-data2.zip › Figure 2-source data 2/2E/48h/p-mTOR/1-PMTOR.Tif]

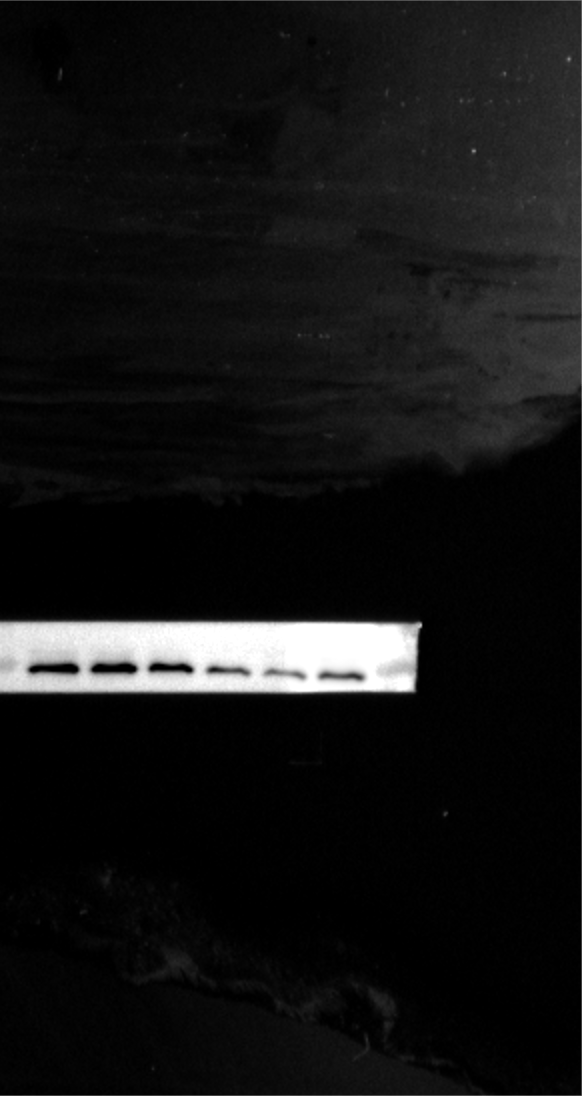

Supplement: Figure 2—source data 2. [file elife-104060-fig2-data2.zip › Figure 2-source data 2/2E/48h/p-p38/1 p-p38 merge=.png]

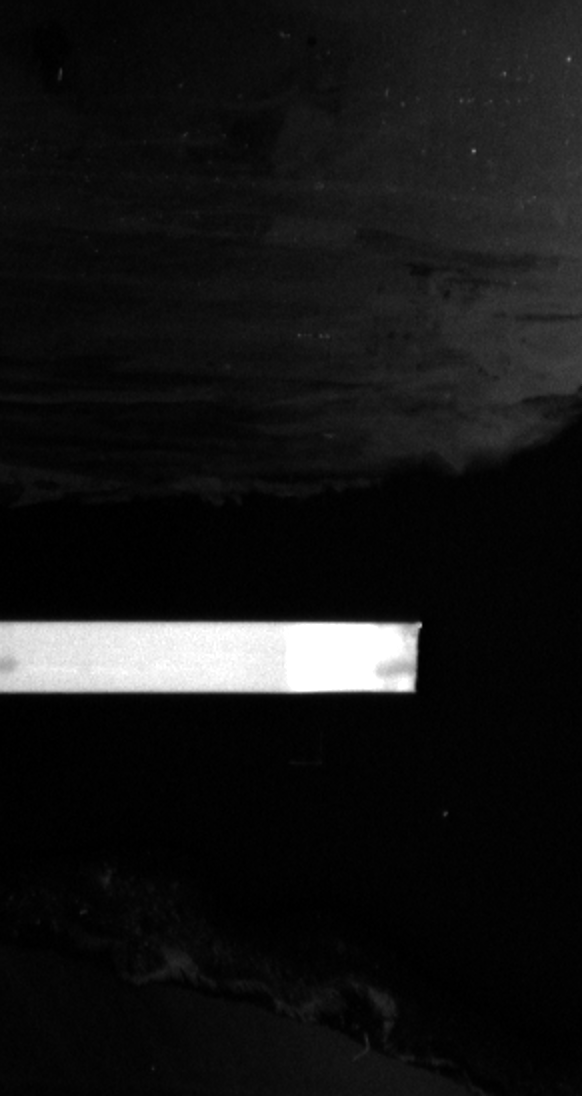

Supplement: Figure 2—source data 2. [file elife-104060-fig2-data2.zip › Figure 2-source data 2/2E/48h/p-p38/1 p-p38 white=.Tif]

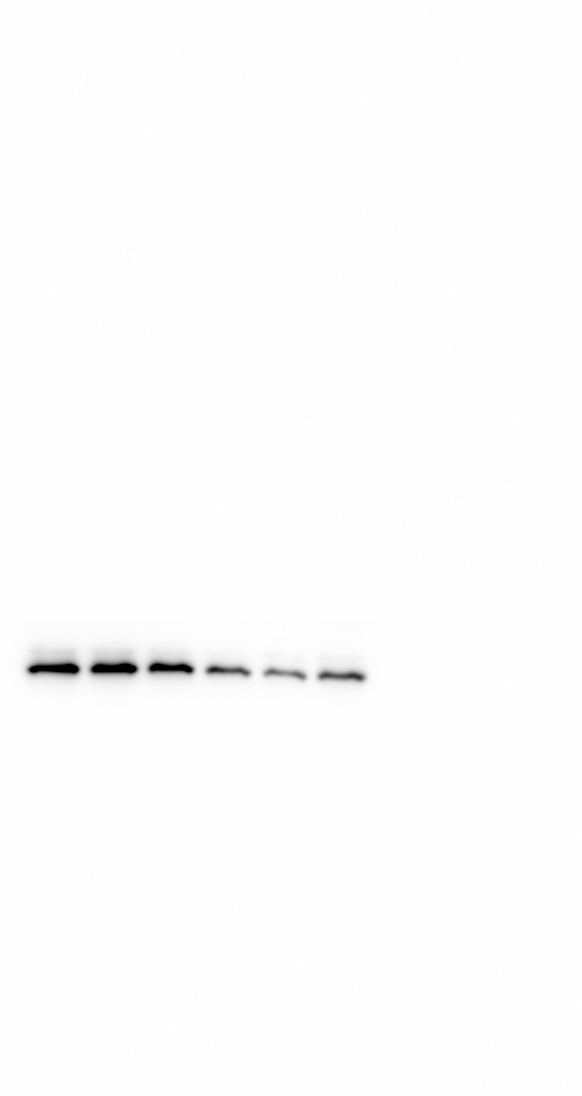

Supplement: Figure 2—source data 2. [file elife-104060-fig2-data2.zip › Figure 2-source data 2/2E/48h/p-p38/1 p-p38=.Tif]

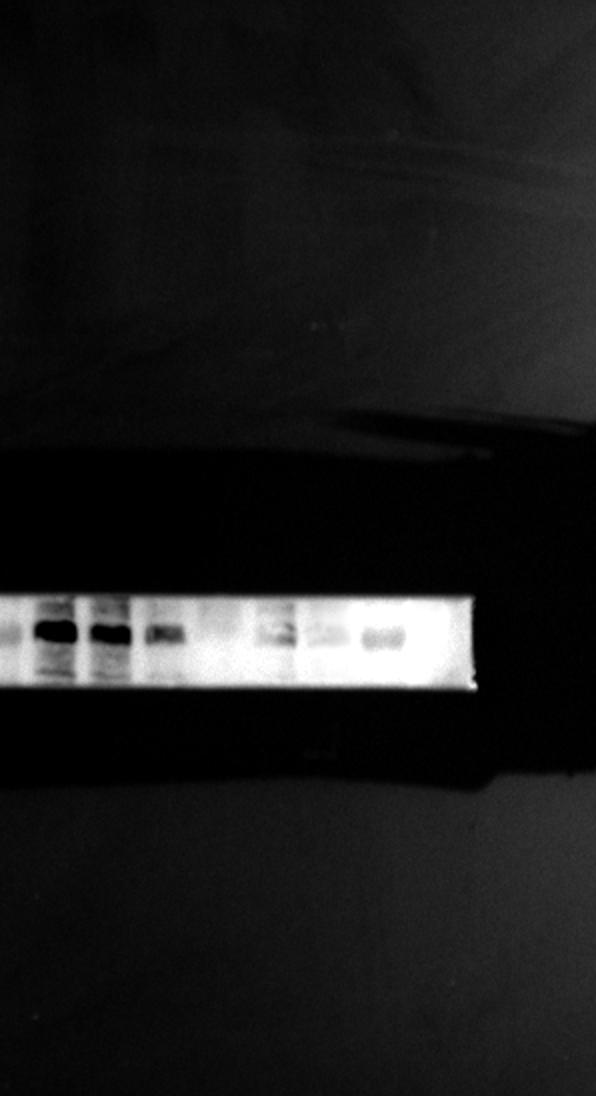

Supplement: Figure 2—source data 2. [file elife-104060-fig2-data2.zip › Figure 2-source data 2/2E/48h/p-SHP2/1-PSHP2 MERGE.Tif]

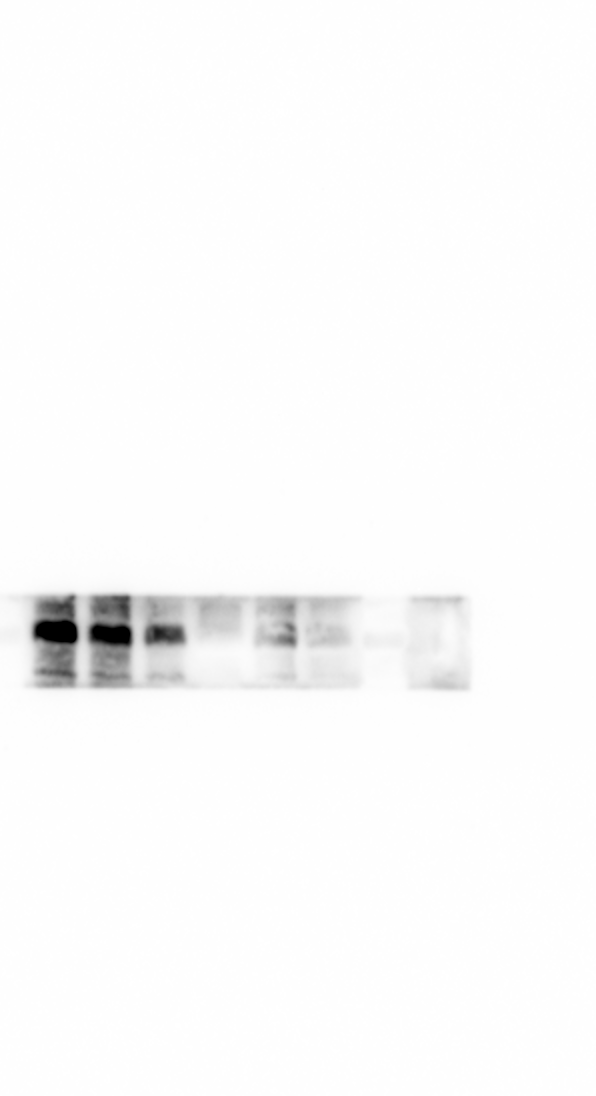

Supplement: Figure 2—source data 2. [file elife-104060-fig2-data2.zip › Figure 2-source data 2/2E/48h/p-SHP2/1-PSHP2.Tif]

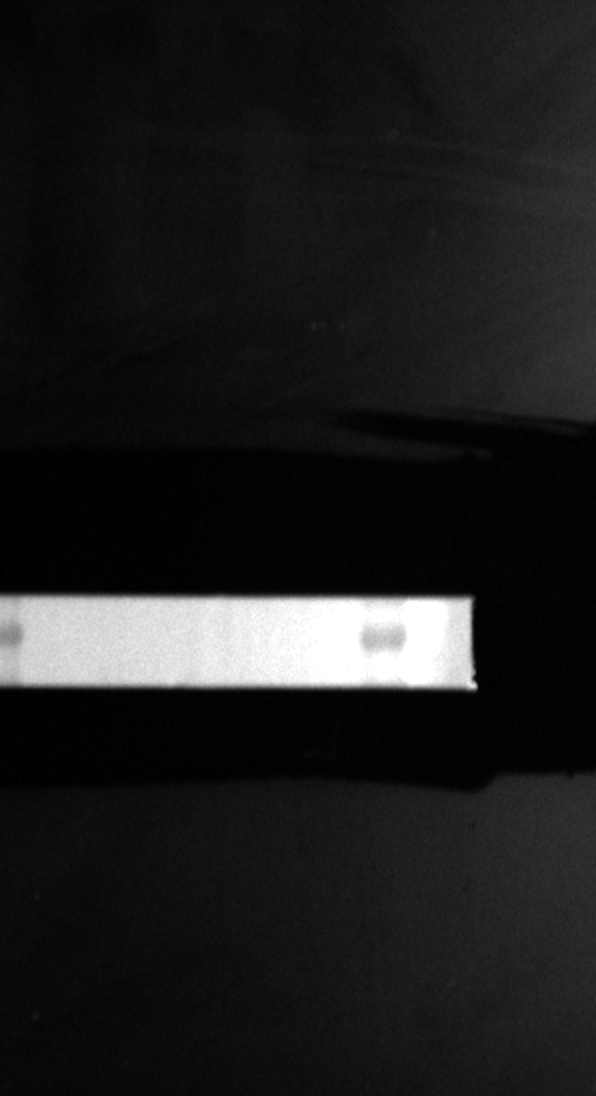

Supplement: Figure 2—source data 2. [file elife-104060-fig2-data2.zip › Figure 2-source data 2/2E/48h/p-SHP2/2-PSHP2 WHITE.Tif]

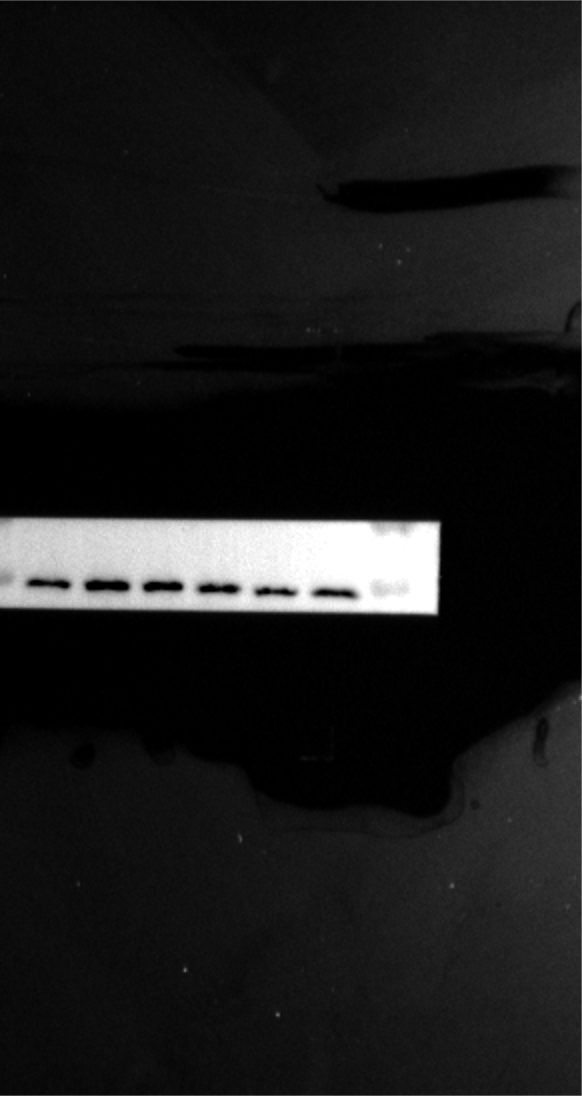

Supplement: Figure 2—source data 2. [file elife-104060-fig2-data2.zip › Figure 2-source data 2/2E/48h/p38/p38 merge=.png]

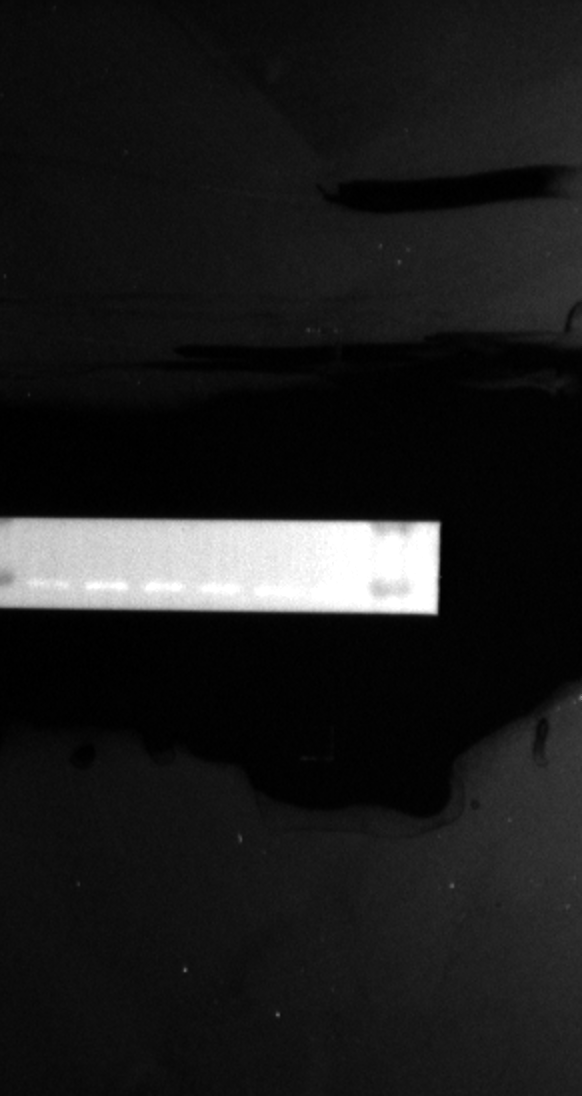

Supplement: Figure 2—source data 2. [file elife-104060-fig2-data2.zip › Figure 2-source data 2/2E/48h/p38/p38 white=.Tif]

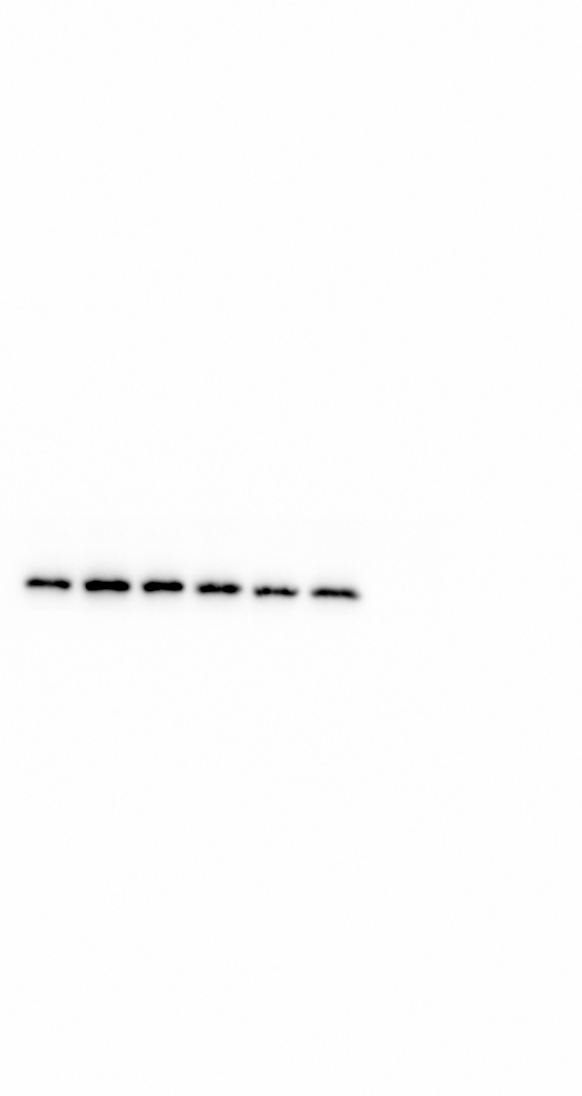

Supplement: Figure 2—source data 2. [file elife-104060-fig2-data2.zip › Figure 2-source data 2/2E/48h/p38/p38=.Tif]

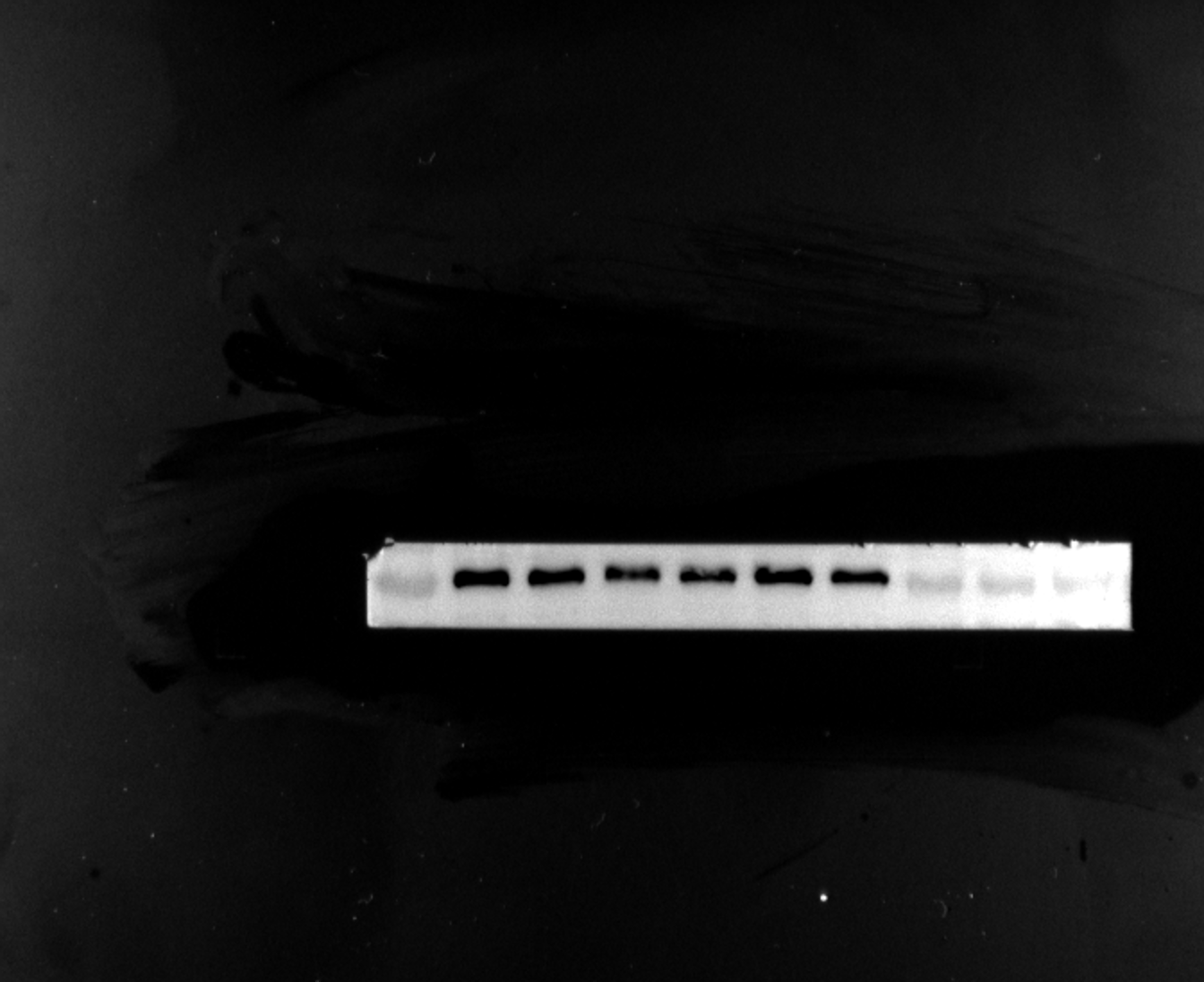

Supplement: Figure 2—source data 2. [file elife-104060-fig2-data2.zip › Figure 2-source data 2/2E/48h/SHP2/shp2 merge.Tif]

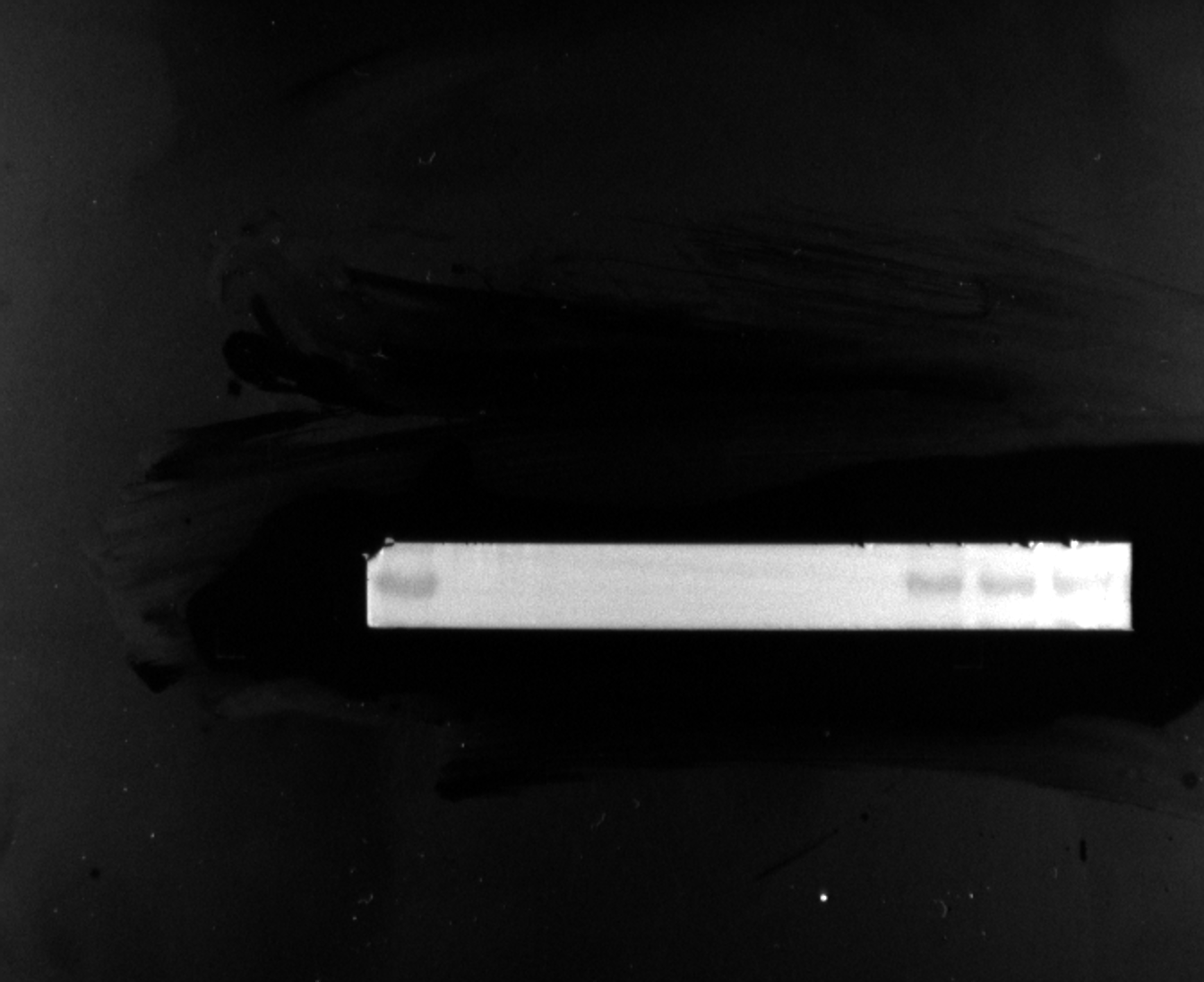

Supplement: Figure 2—source data 2. [file elife-104060-fig2-data2.zip › Figure 2-source data 2/2E/48h/SHP2/shp2 white.Tif]

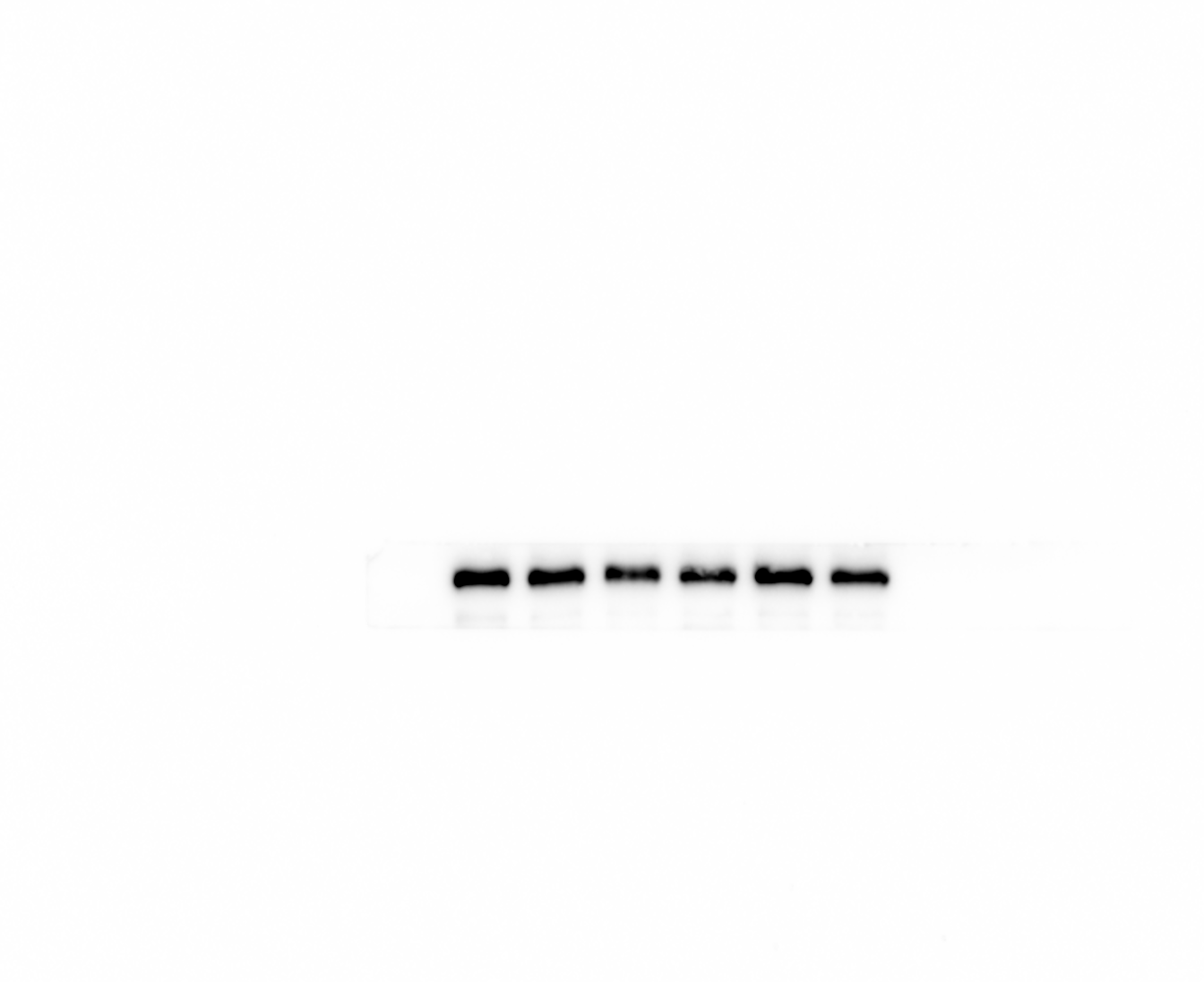

Supplement: Figure 2—source data 2. [file elife-104060-fig2-data2.zip › Figure 2-source data 2/2E/48h/SHP2/shp2.Tif]

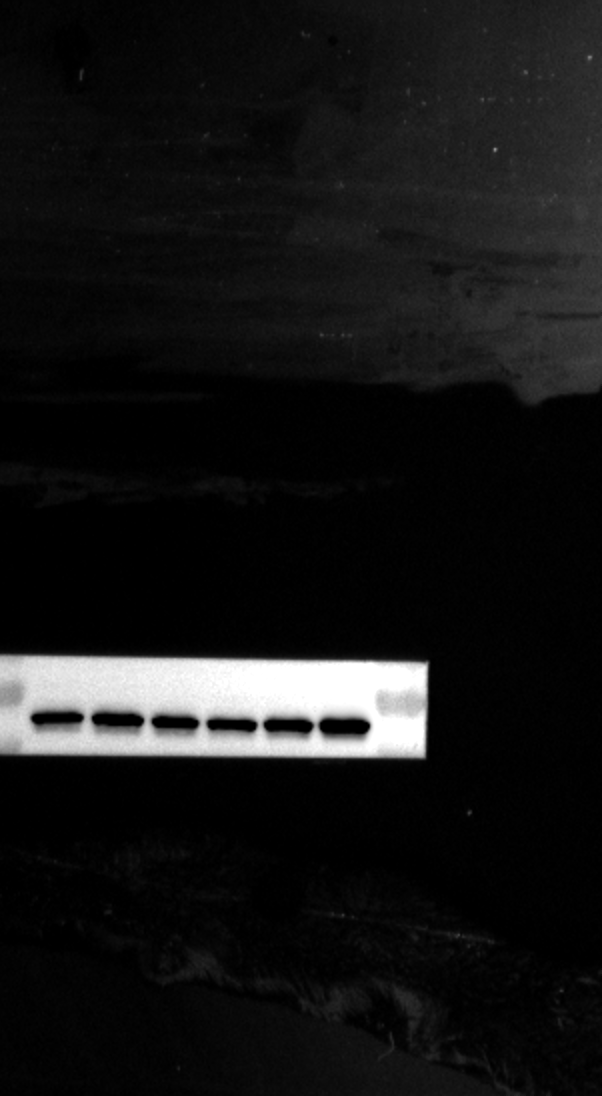

Supplement: Figure 2—source data 2. [file elife-104060-fig2-data2.zip › Figure 2-source data 2/2F/1h/AKT/2 akt merge.Tif]

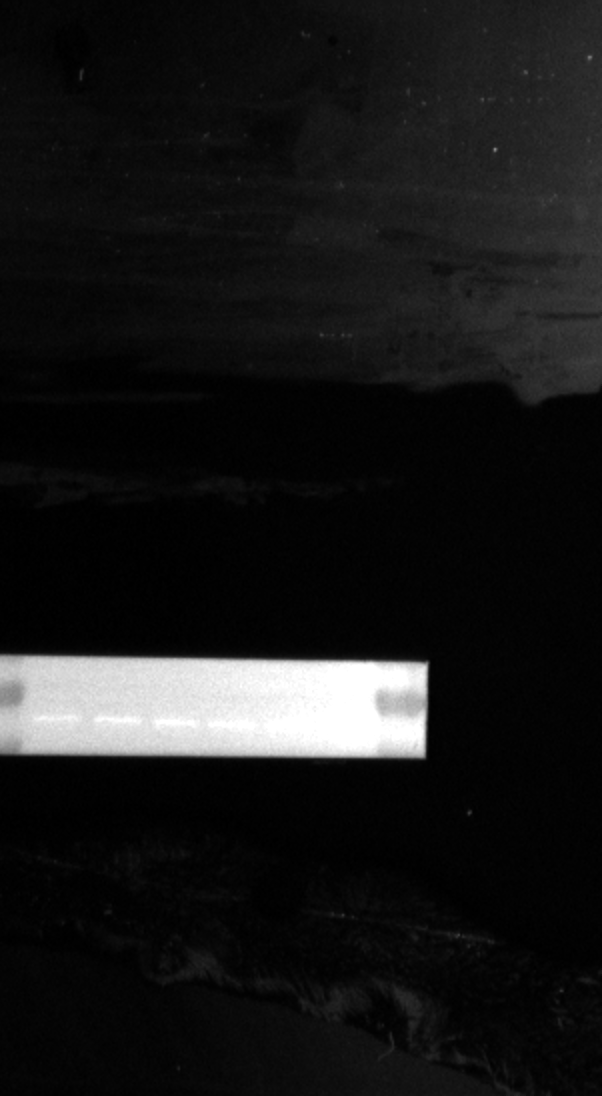

Supplement: Figure 2—source data 2. [file elife-104060-fig2-data2.zip › Figure 2-source data 2/2F/1h/AKT/2 akt white=.Tif]

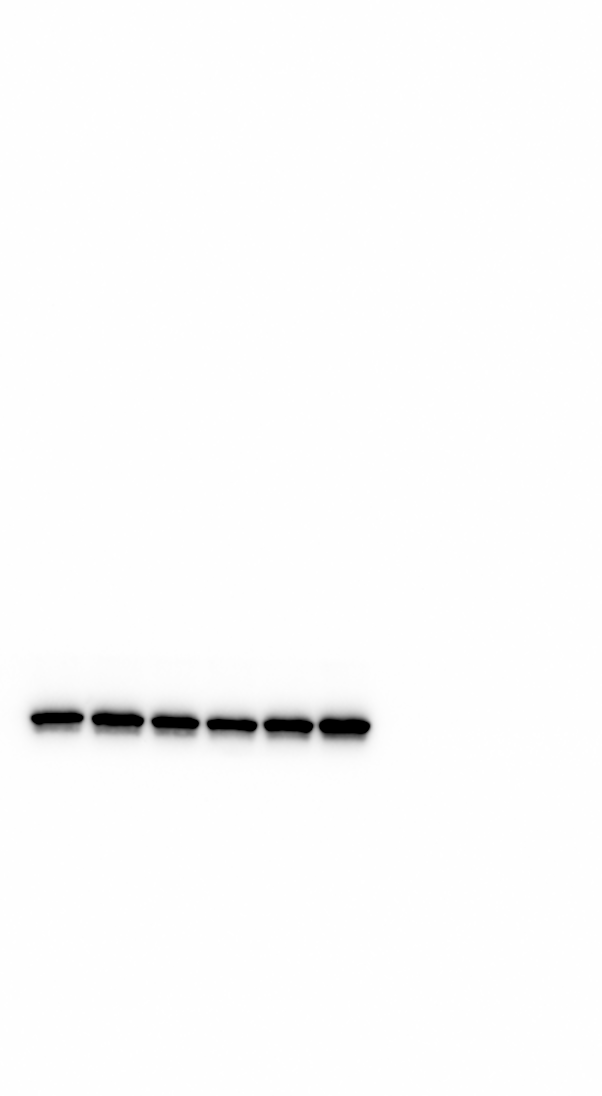

Supplement: Figure 2—source data 2. [file elife-104060-fig2-data2.zip › Figure 2-source data 2/2F/1h/AKT/2 akt=.Tif]

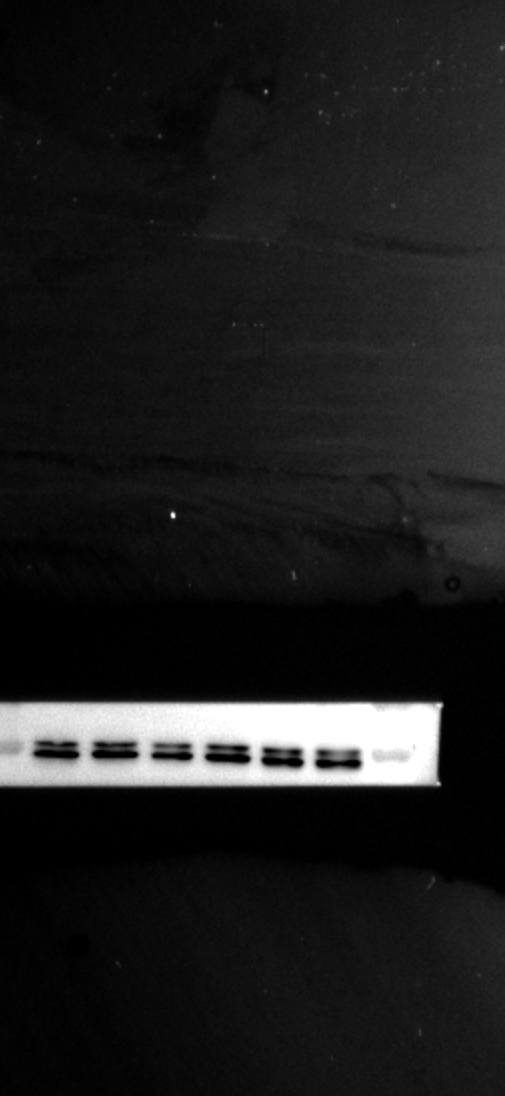

Supplement: Figure 2—source data 2. [file elife-104060-fig2-data2.zip › Figure 2-source data 2/2F/1h/ERK/1 ERK MERGE.Tif]

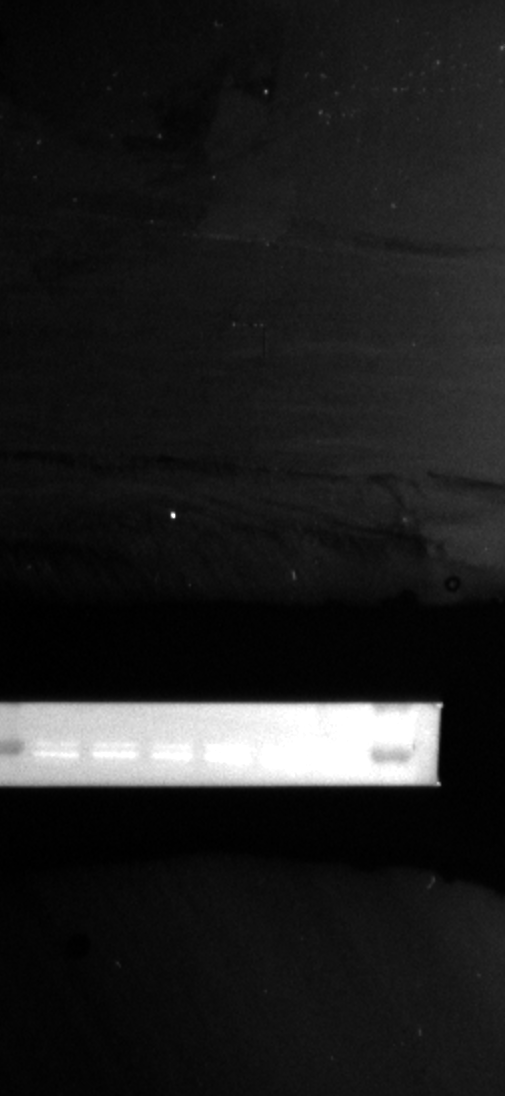

Supplement: Figure 2—source data 2. [file elife-104060-fig2-data2.zip › Figure 2-source data 2/2F/1h/ERK/1 ERK WHITE=.Tif]

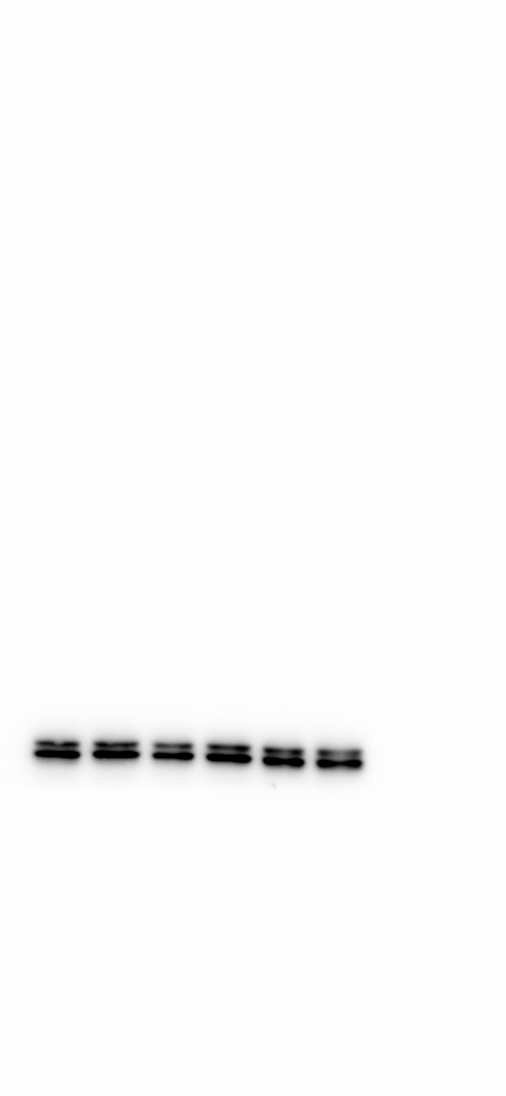

Supplement: Figure 2—source data 2. [file elife-104060-fig2-data2.zip › Figure 2-source data 2/2F/1h/ERK/1 ERK=.Tif]

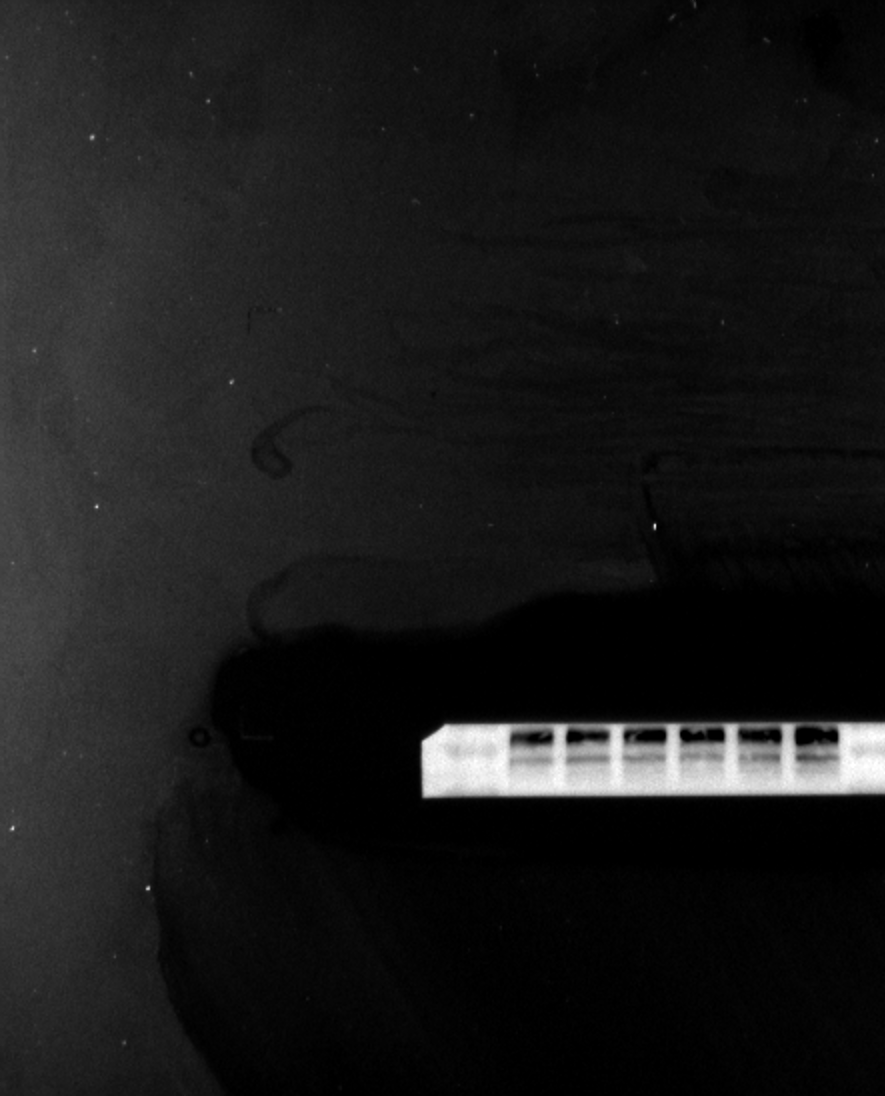

Supplement: Figure 2—source data 2. [file elife-104060-fig2-data2.zip › Figure 2-source data 2/2F/1h/FGFR2/1 FGFR2 MERGE.Tif]

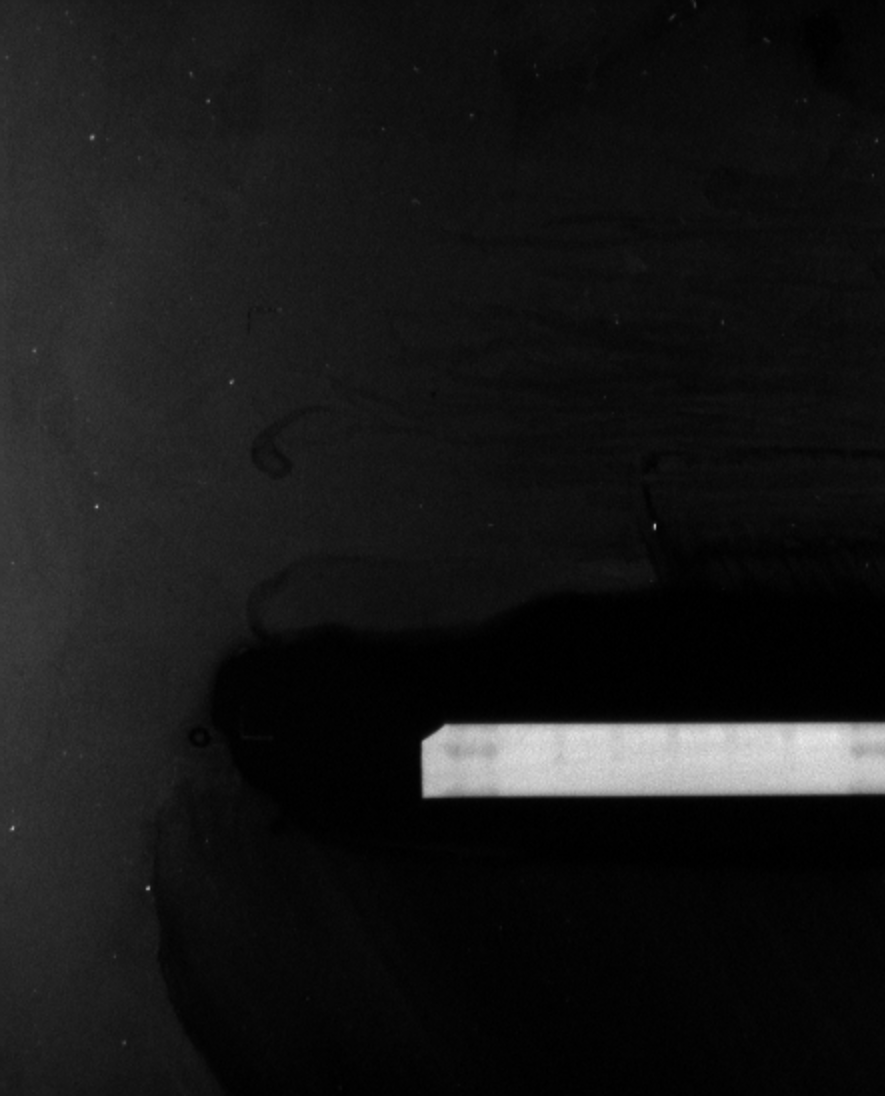

Supplement: Figure 2—source data 2. [file elife-104060-fig2-data2.zip › Figure 2-source data 2/2F/1h/FGFR2/1 FGFR2 WHITE.Tif]

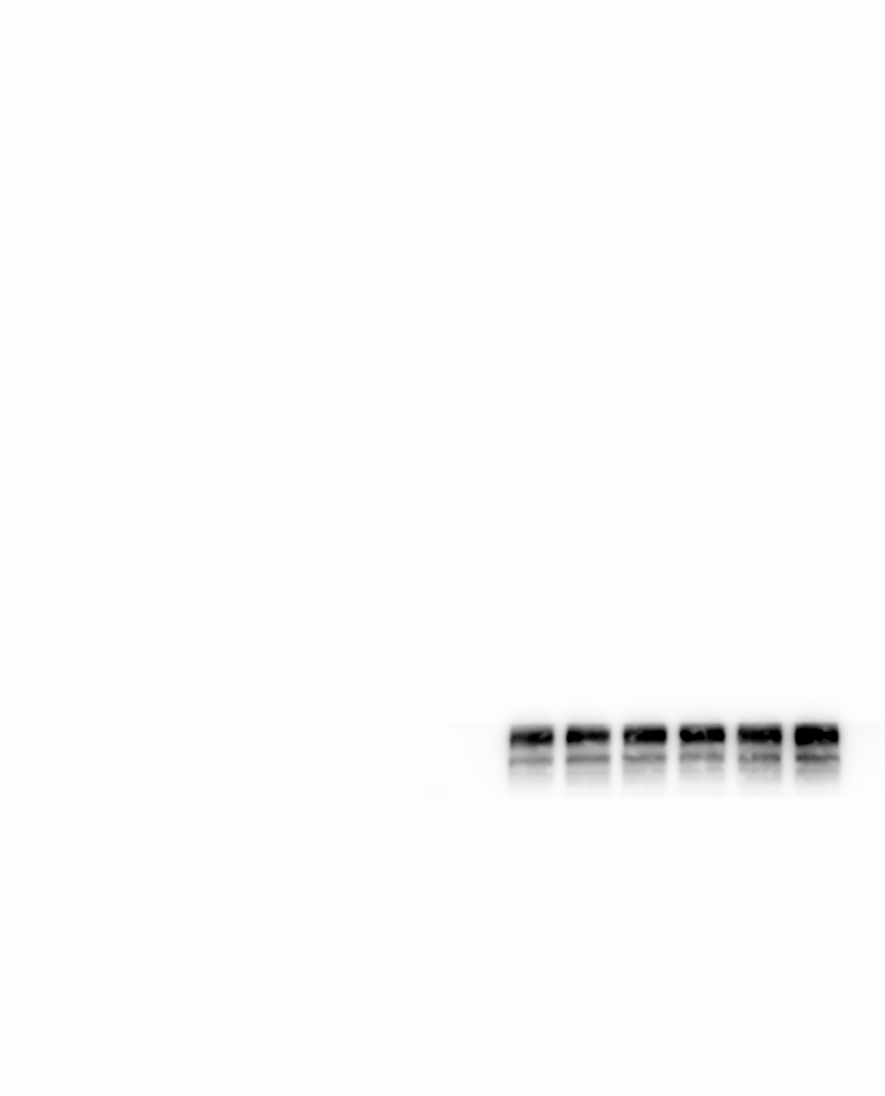

Supplement: Figure 2—source data 2. [file elife-104060-fig2-data2.zip › Figure 2-source data 2/2F/1h/FGFR2/1 FGFR2.Tif]

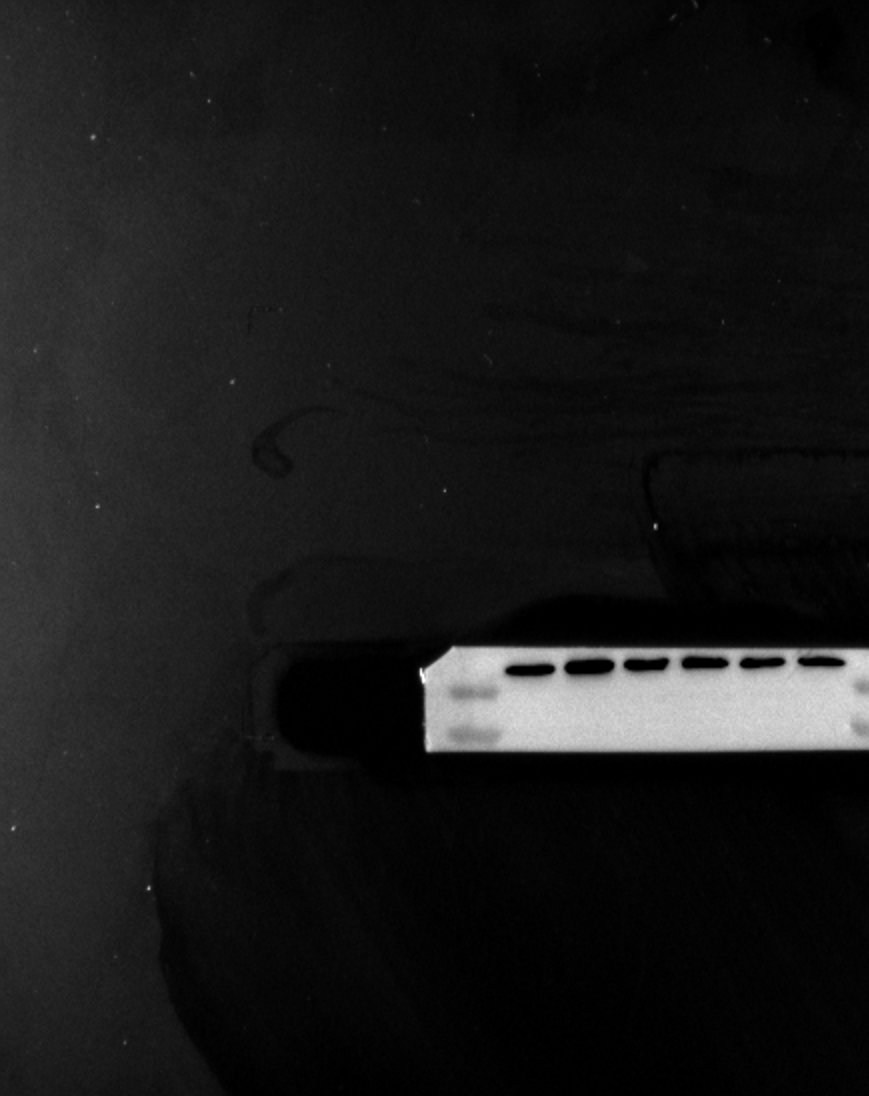

Supplement: Figure 2—source data 2. [file elife-104060-fig2-data2.zip › Figure 2-source data 2/2F/1h/GAPDH/1 GAP MERGE.Tif]

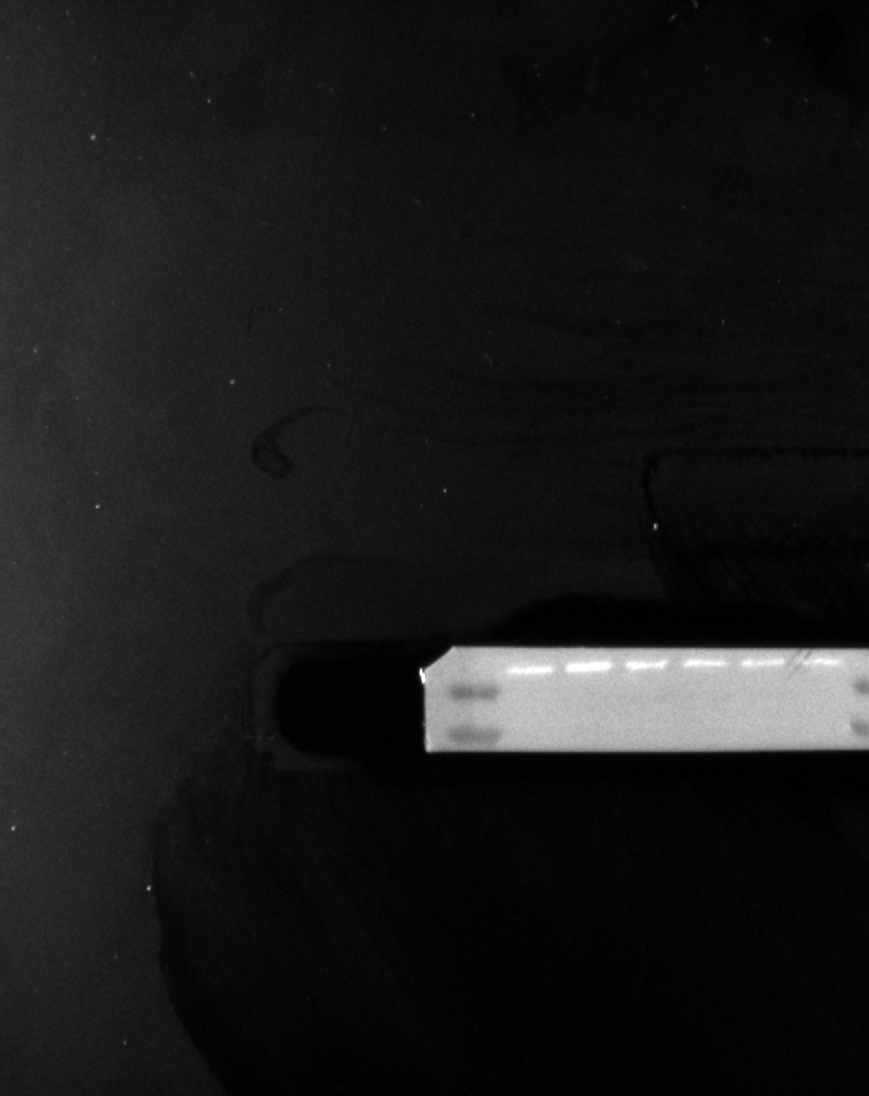

Supplement: Figure 2—source data 2. [file elife-104060-fig2-data2.zip › Figure 2-source data 2/2F/1h/GAPDH/1 GAP WHITE=.Tif]

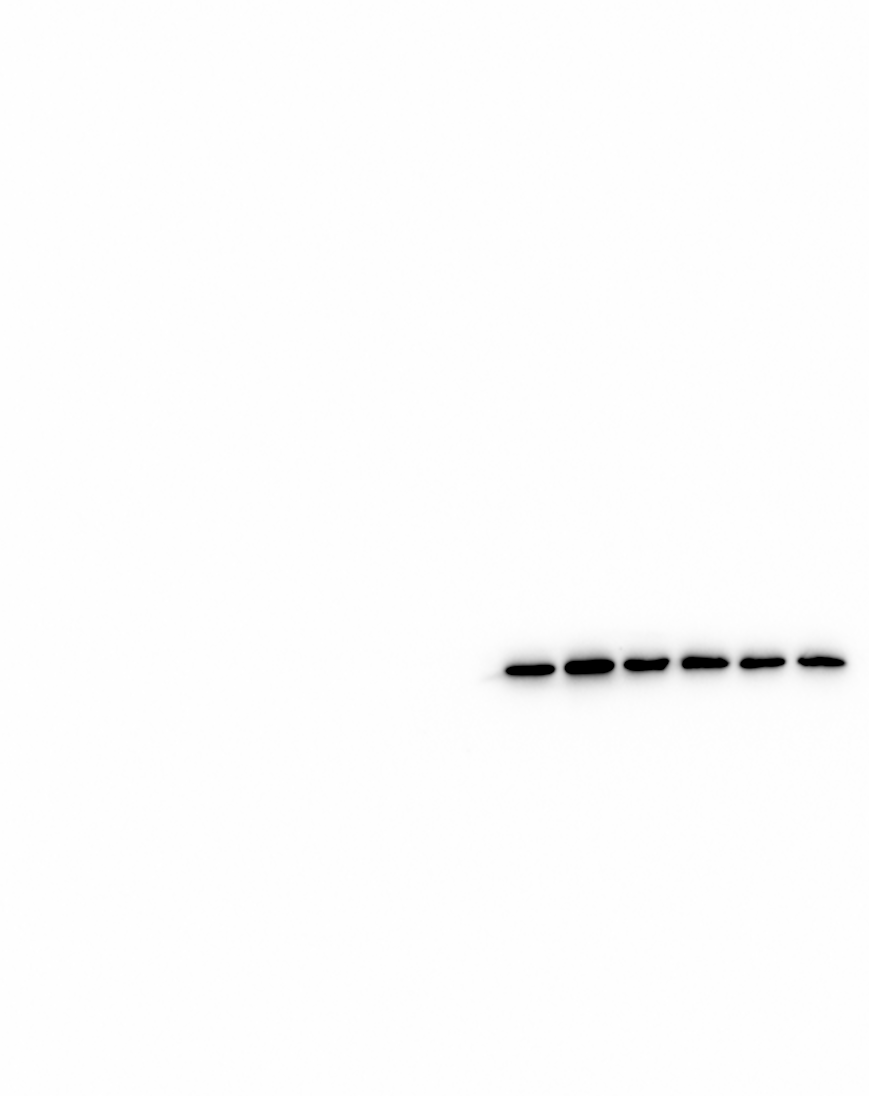

Supplement: Figure 2—source data 2. [file elife-104060-fig2-data2.zip › Figure 2-source data 2/2F/1h/GAPDH/1 GAP=.Tif]

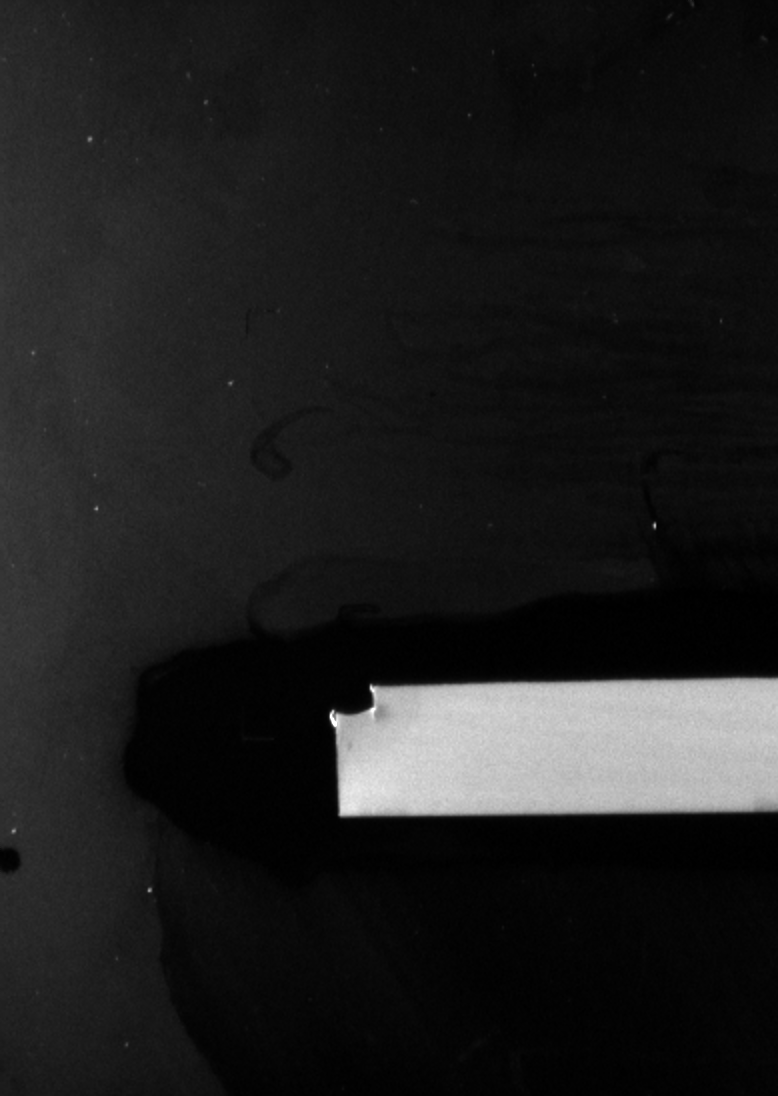

Supplement: Figure 2—source data 2. [file elife-104060-fig2-data2.zip › Figure 2-source data 2/2F/1h/mTOR/2 MROR WHITE=.Tif]

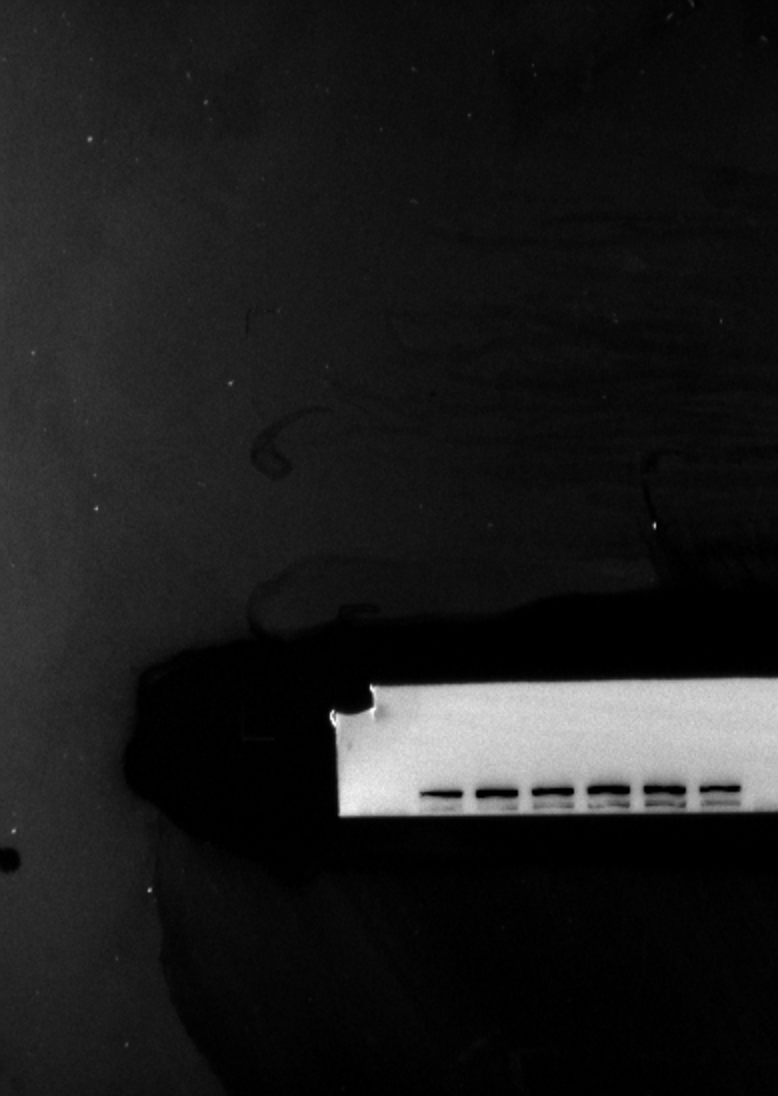

Supplement: Figure 2—source data 2. [file elife-104060-fig2-data2.zip › Figure 2-source data 2/2F/1h/mTOR/2 MTOR MERGE.Tif]

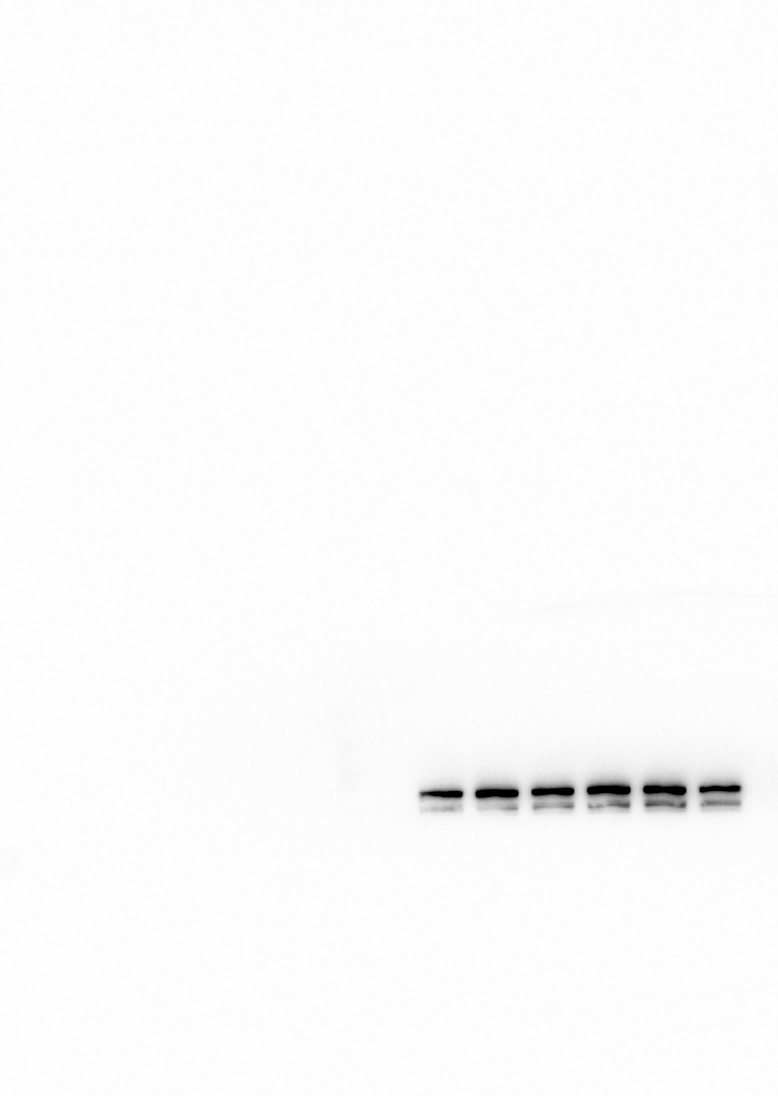

Supplement: Figure 2—source data 2. [file elife-104060-fig2-data2.zip › Figure 2-source data 2/2F/1h/mTOR/2 MTOR=.Tif]

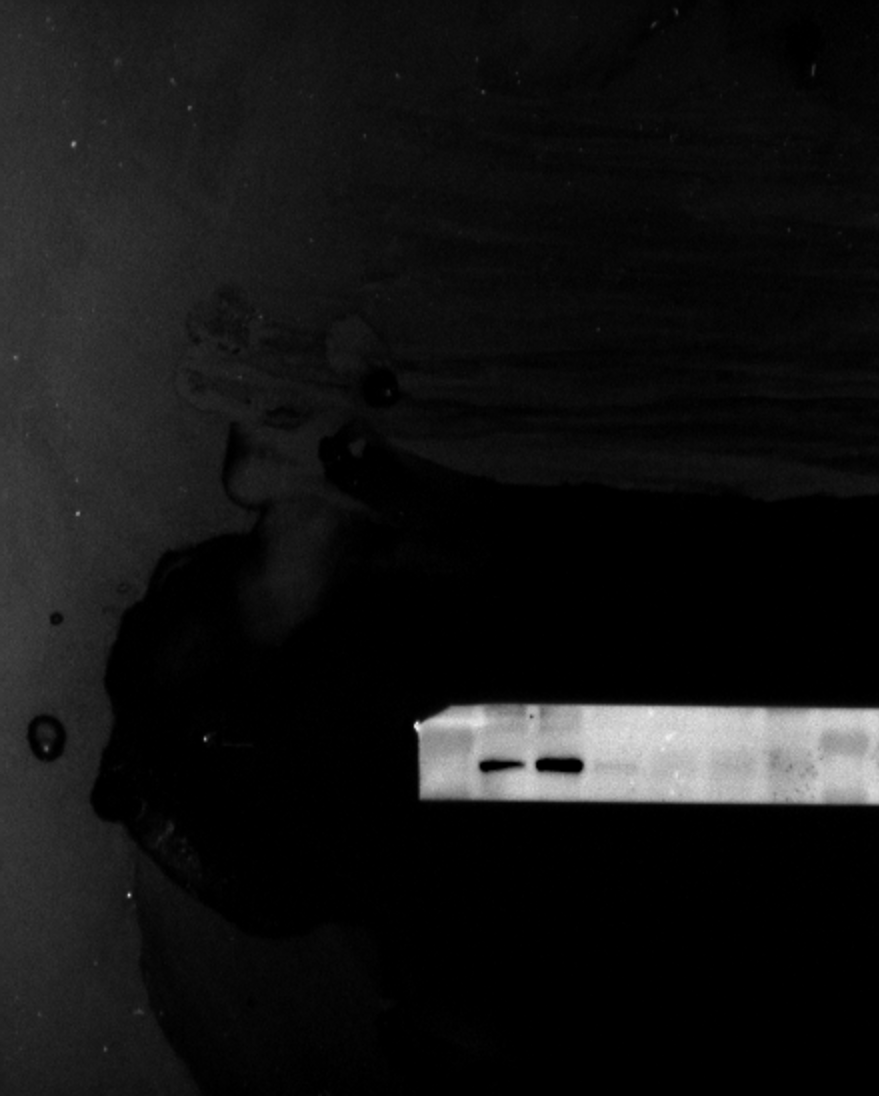

Supplement: Figure 2—source data 2. [file elife-104060-fig2-data2.zip › Figure 2-source data 2/2F/1h/p-AKT/p-akt merge.Tif]

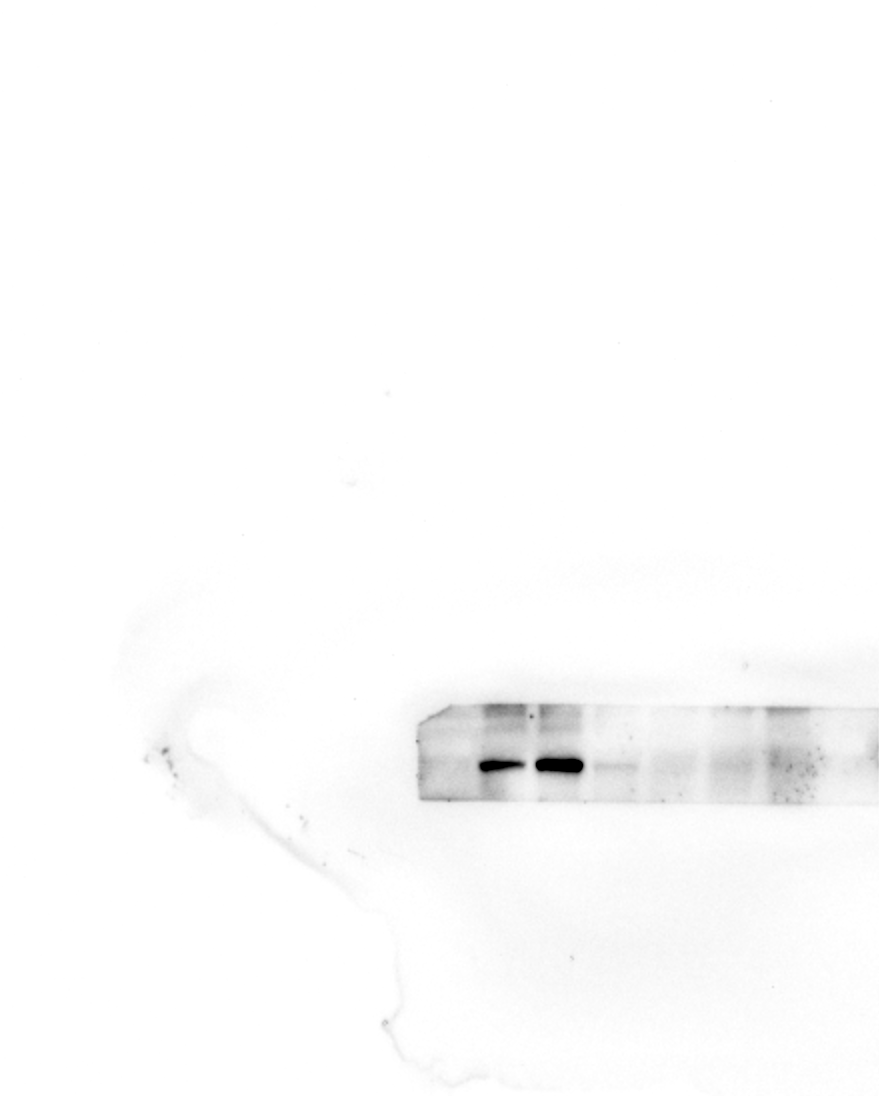

Supplement: Figure 2—source data 2. [file elife-104060-fig2-data2.zip › Figure 2-source data 2/2F/1h/p-AKT/p-akt.png]

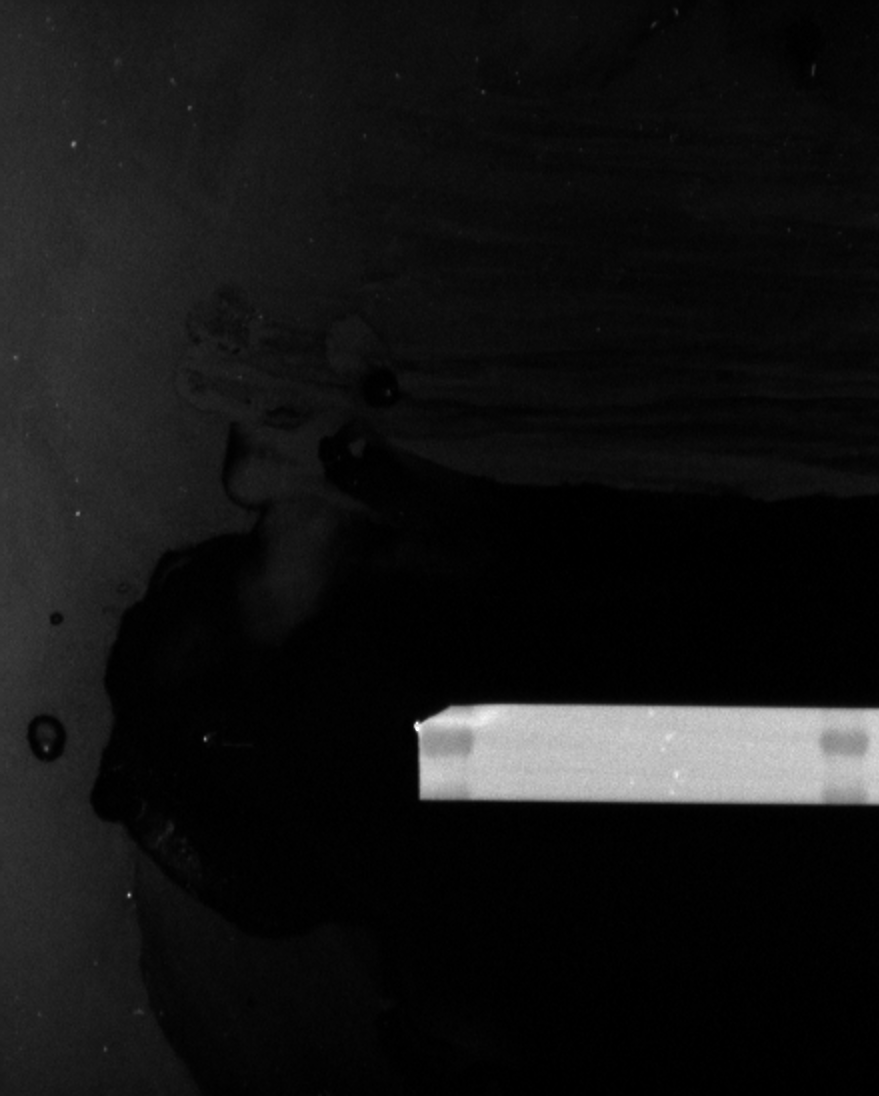

Supplement: Figure 2—source data 2. [file elife-104060-fig2-data2.zip › Figure 2-source data 2/2F/1h/p-AKT/p-pakt white.Tif]

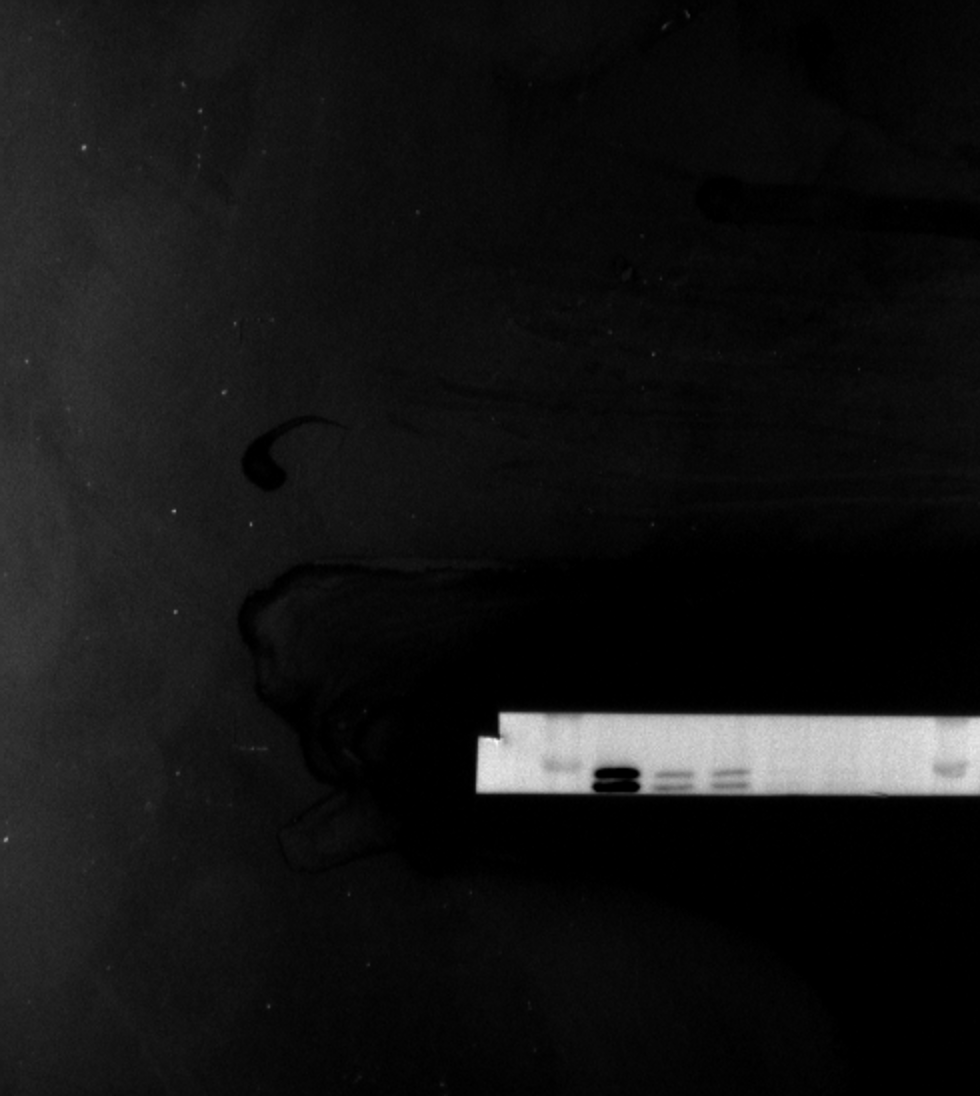

Supplement: Figure 2—source data 2. [file elife-104060-fig2-data2.zip › Figure 2-source data 2/2F/1h/p-ERK/1 PERK MERGE.Tif]

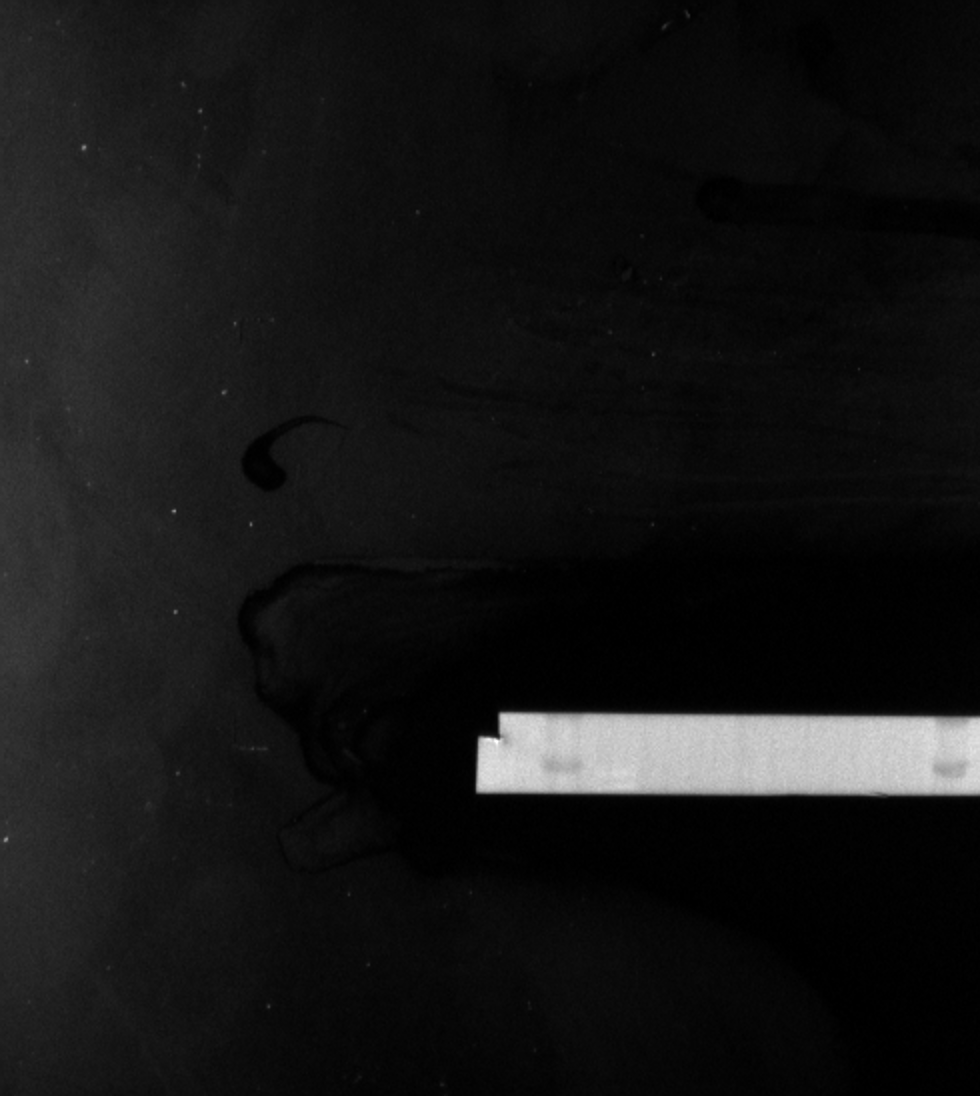

Supplement: Figure 2—source data 2. [file elife-104060-fig2-data2.zip › Figure 2-source data 2/2F/1h/p-ERK/2 PERK WHITE.Tif]

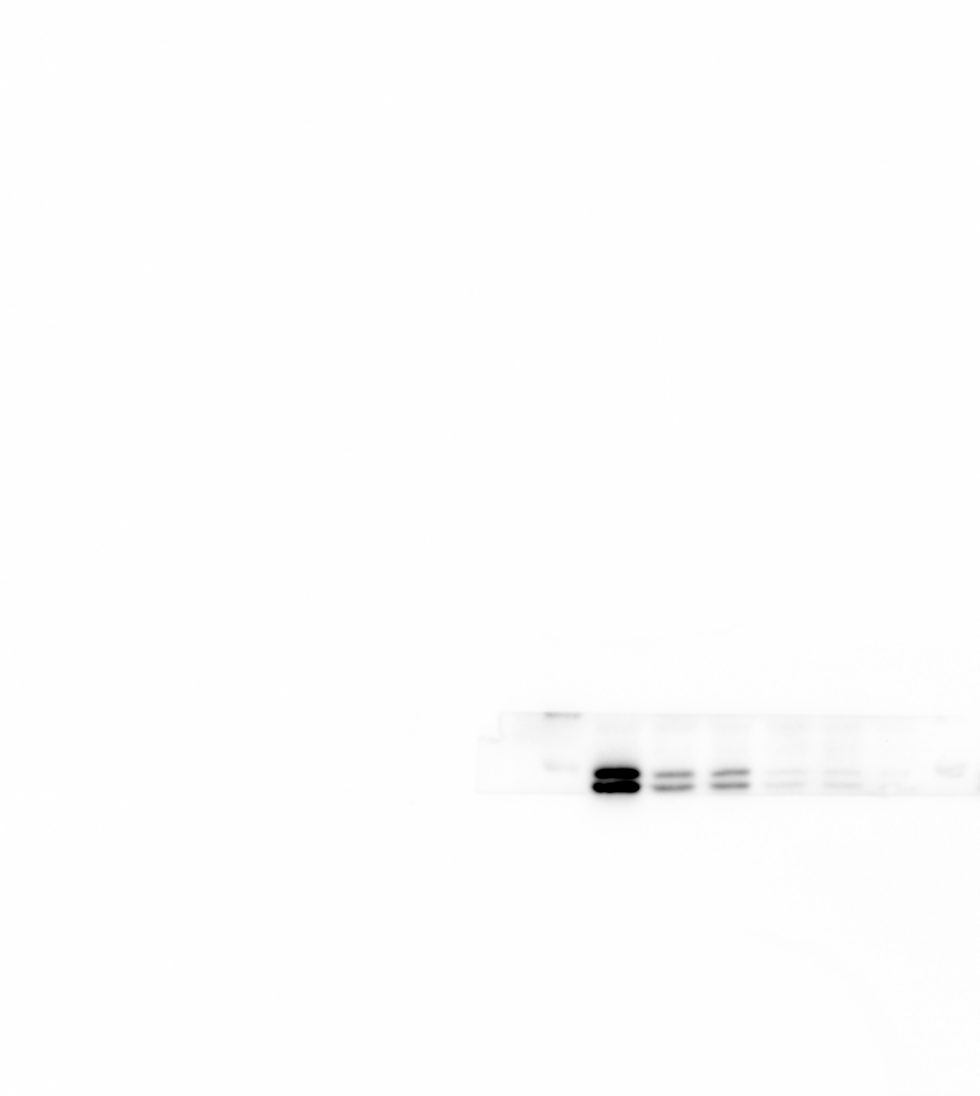

Supplement: Figure 2—source data 2. [file elife-104060-fig2-data2.zip › Figure 2-source data 2/2F/1h/p-ERK/2 PERK.png]

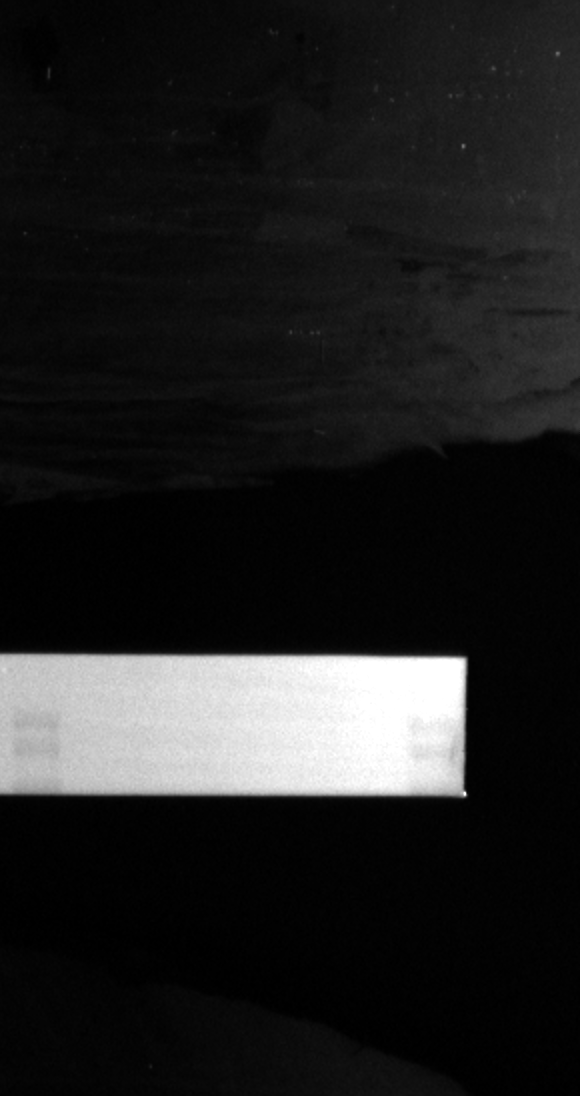

Supplement: Figure 2—source data 2. [file elife-104060-fig2-data2.zip › Figure 2-source data 2/2F/1h/p-FGFR/1 p-fgfr white.Tif]
