# Supplementary figures and images for: Blocking SHP2 benefits FGFR2 inhibitor and overcomes its resistance in FGFR2-amplified gastric cancer (part 3 of 3)
Source: eLife. 2026 Mar 23;14:RP104060. doi: 10.7554/eLife.104060 (PMC13008354; doi:10.7554/eLife.104060)

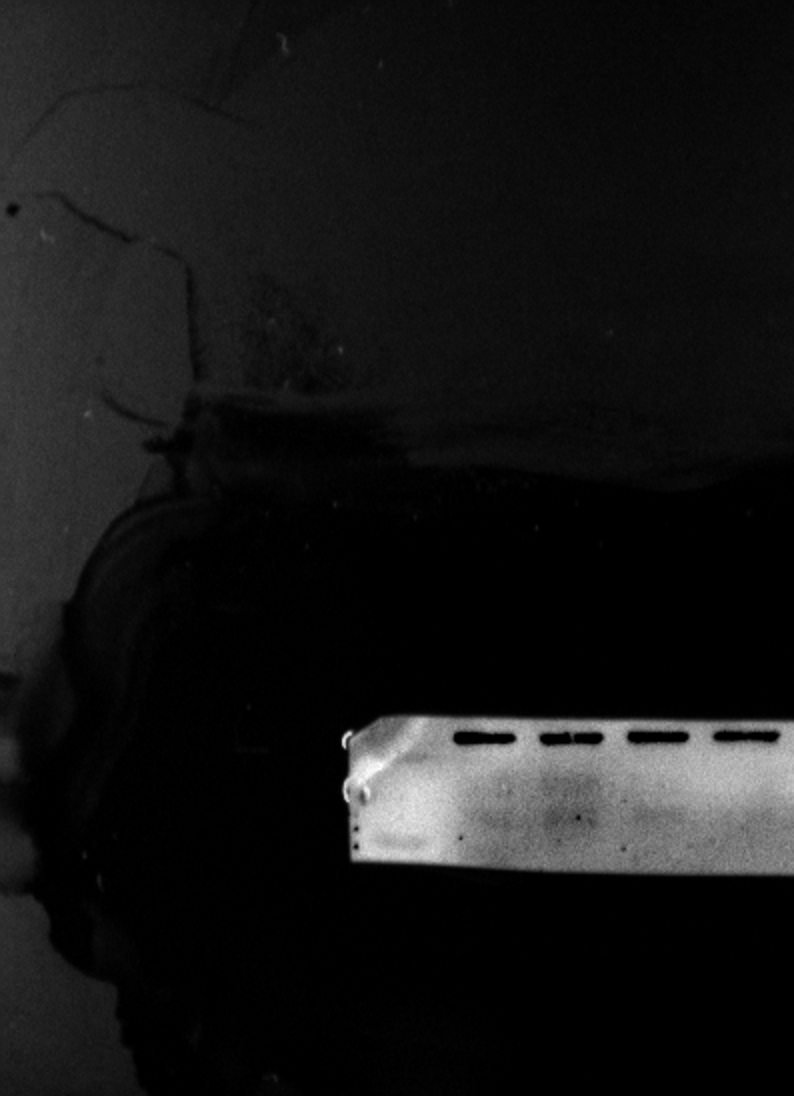

Supplement: Figure 4—source data 2. [file elife-104060-fig4-data2.zip › Figure 4-source data 2/GAPDH/1 gap 2 merge.Tif]

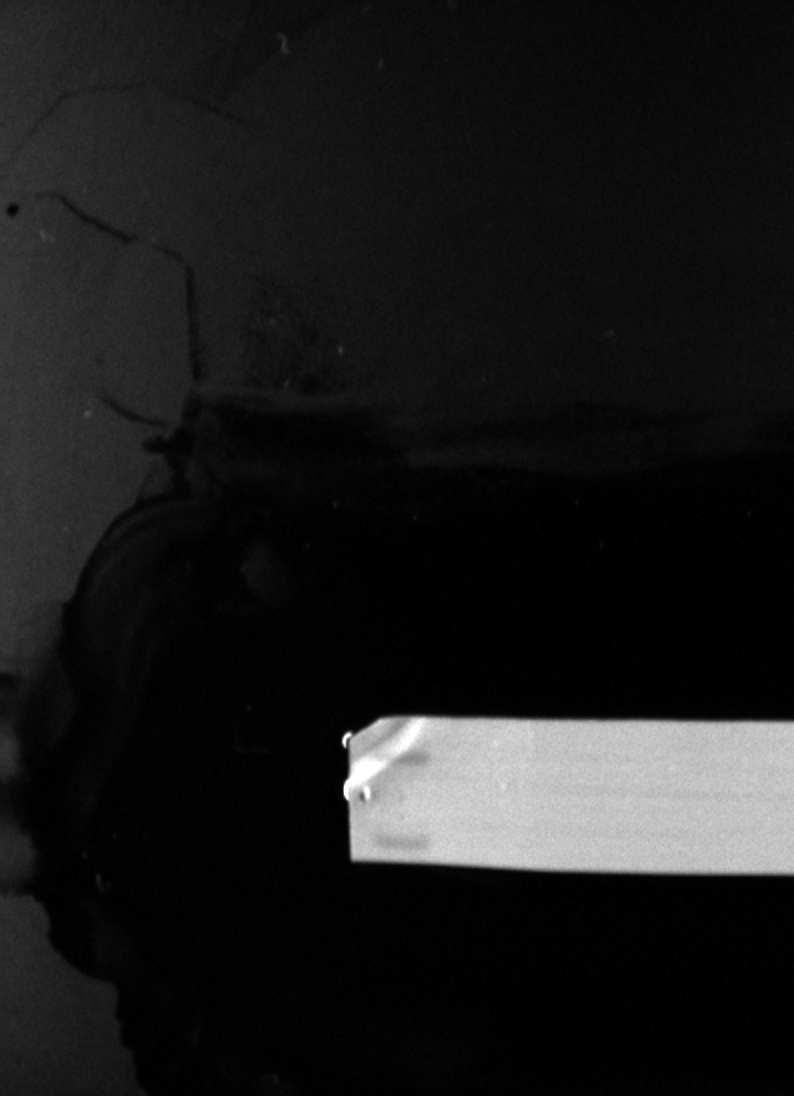

Supplement: Figure 4—source data 2. [file elife-104060-fig4-data2.zip › Figure 4-source data 2/GAPDH/1 gap 2 white=.Tif]

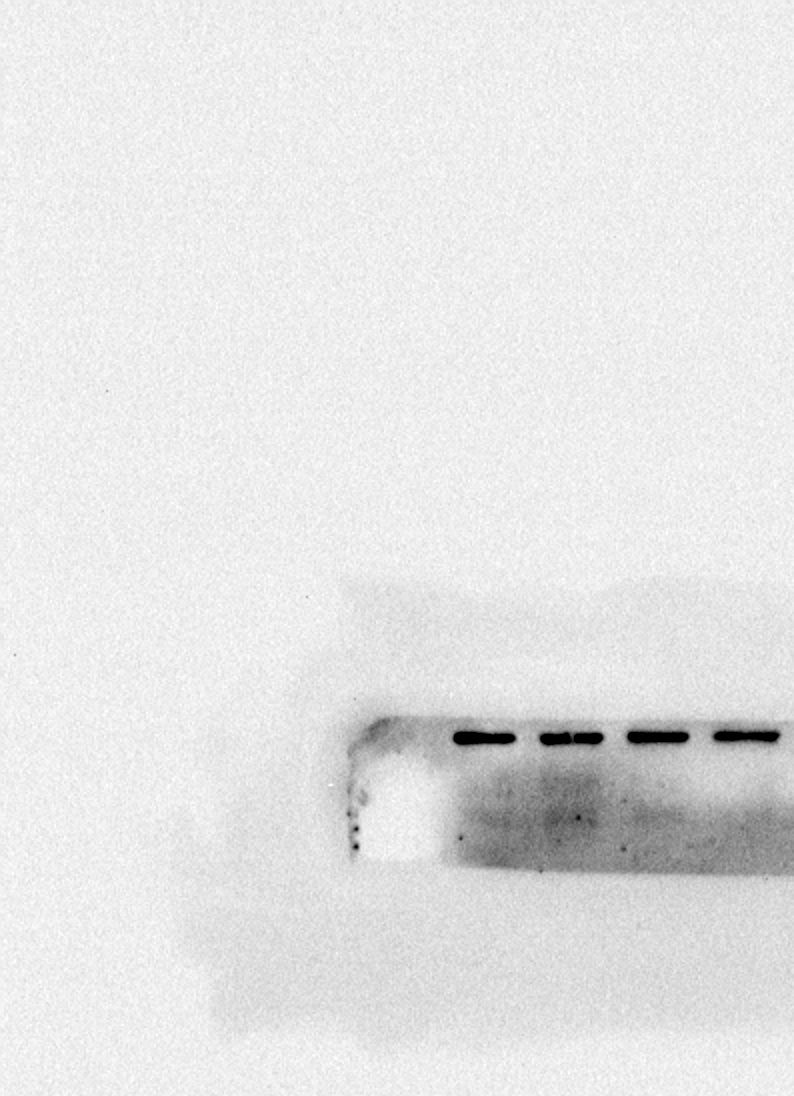

Supplement: Figure 4—source data 2. [file elife-104060-fig4-data2.zip › Figure 4-source data 2/GAPDH/1 gap 2=.Tif]

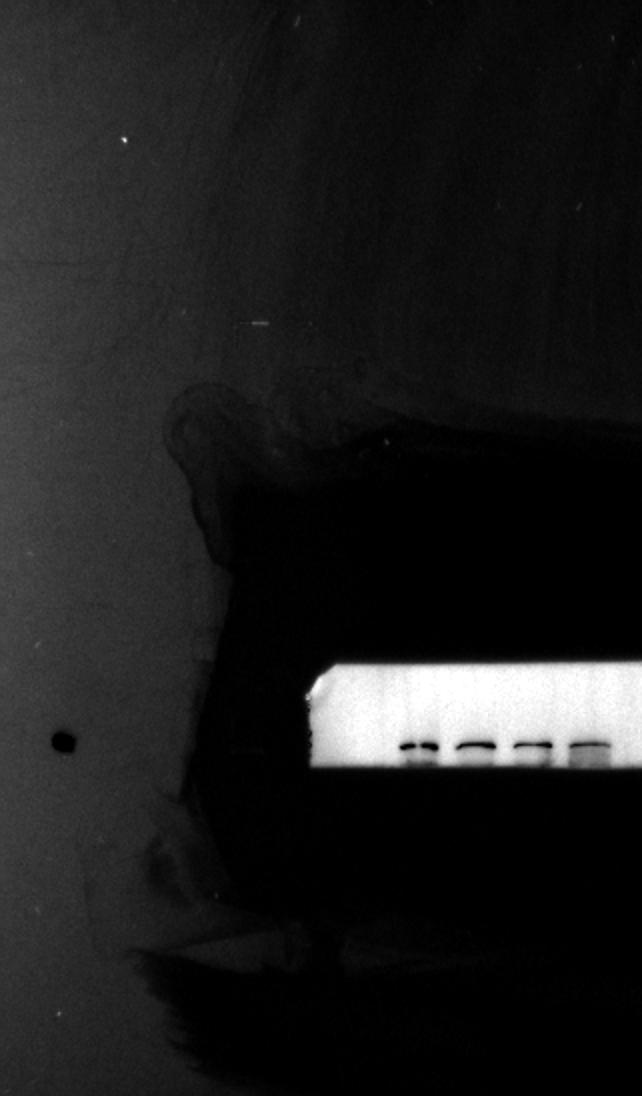

Supplement: Figure 4—source data 2. [file elife-104060-fig4-data2.zip › Figure 4-source data 2/mTOR/mtor merge.Tif]

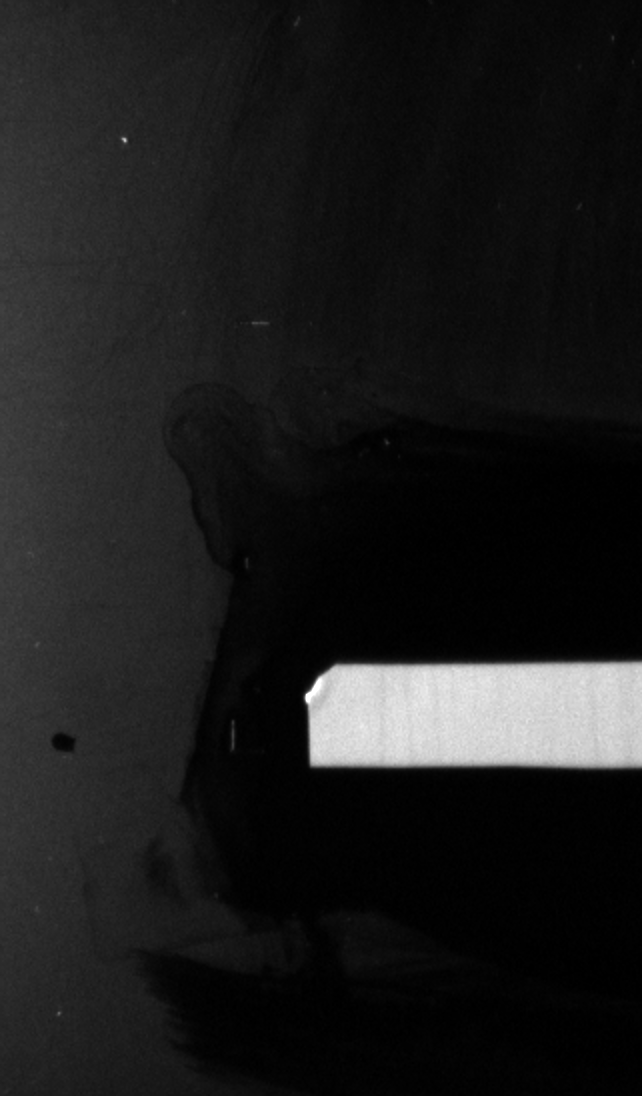

Supplement: Figure 4—source data 2. [file elife-104060-fig4-data2.zip › Figure 4-source data 2/mTOR/mtor white=.Tif]

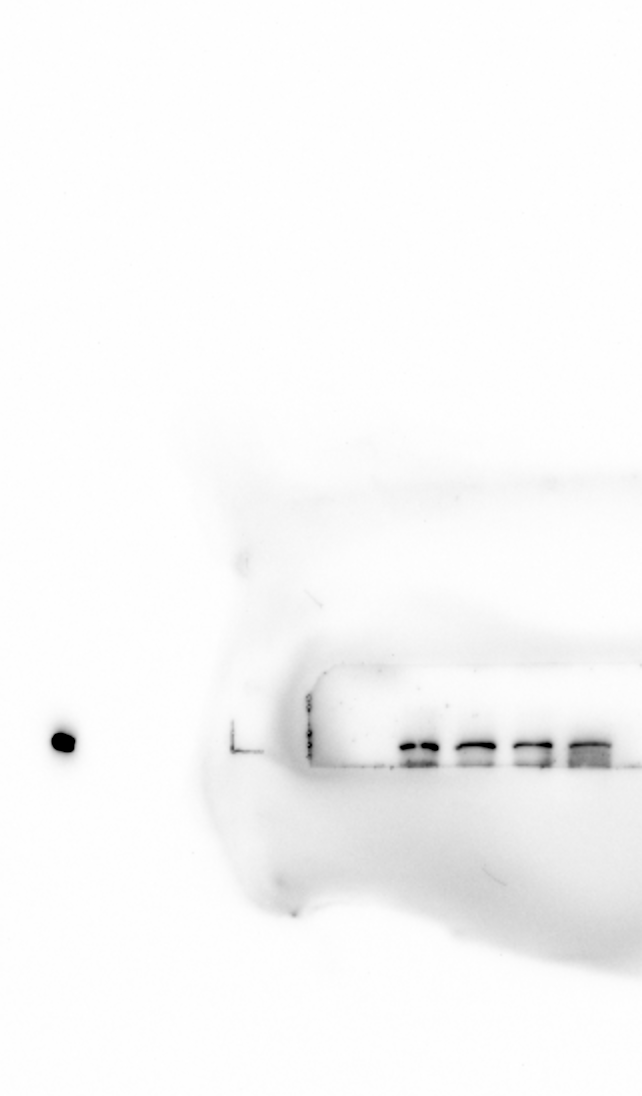

Supplement: Figure 4—source data 2. [file elife-104060-fig4-data2.zip › Figure 4-source data 2/mTOR/mtor=.Tif]

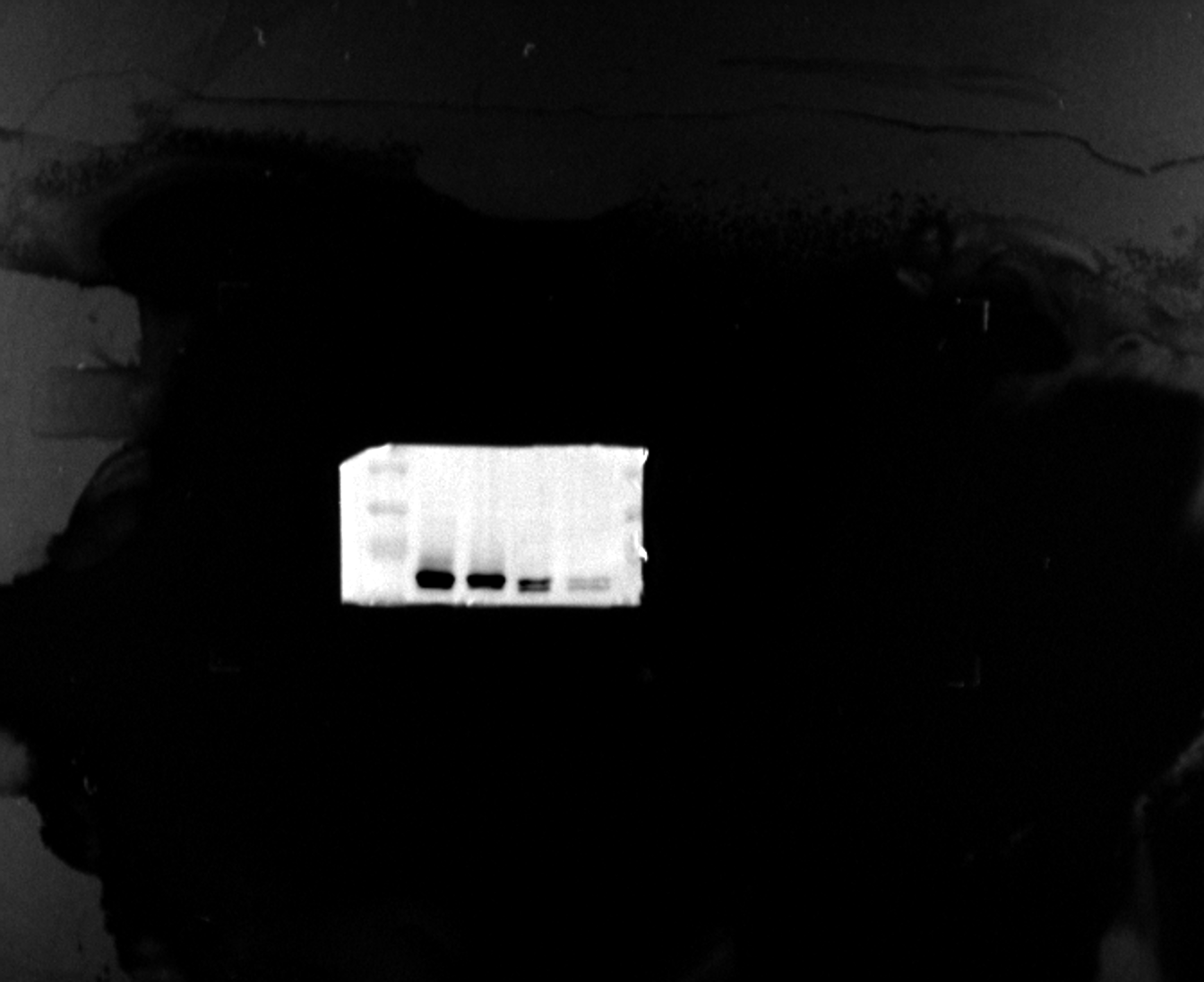

Supplement: Figure 4—source data 2. [file elife-104060-fig4-data2.zip › Figure 4-source data 2/p-AKT/p-akt 2 merge.Tif]

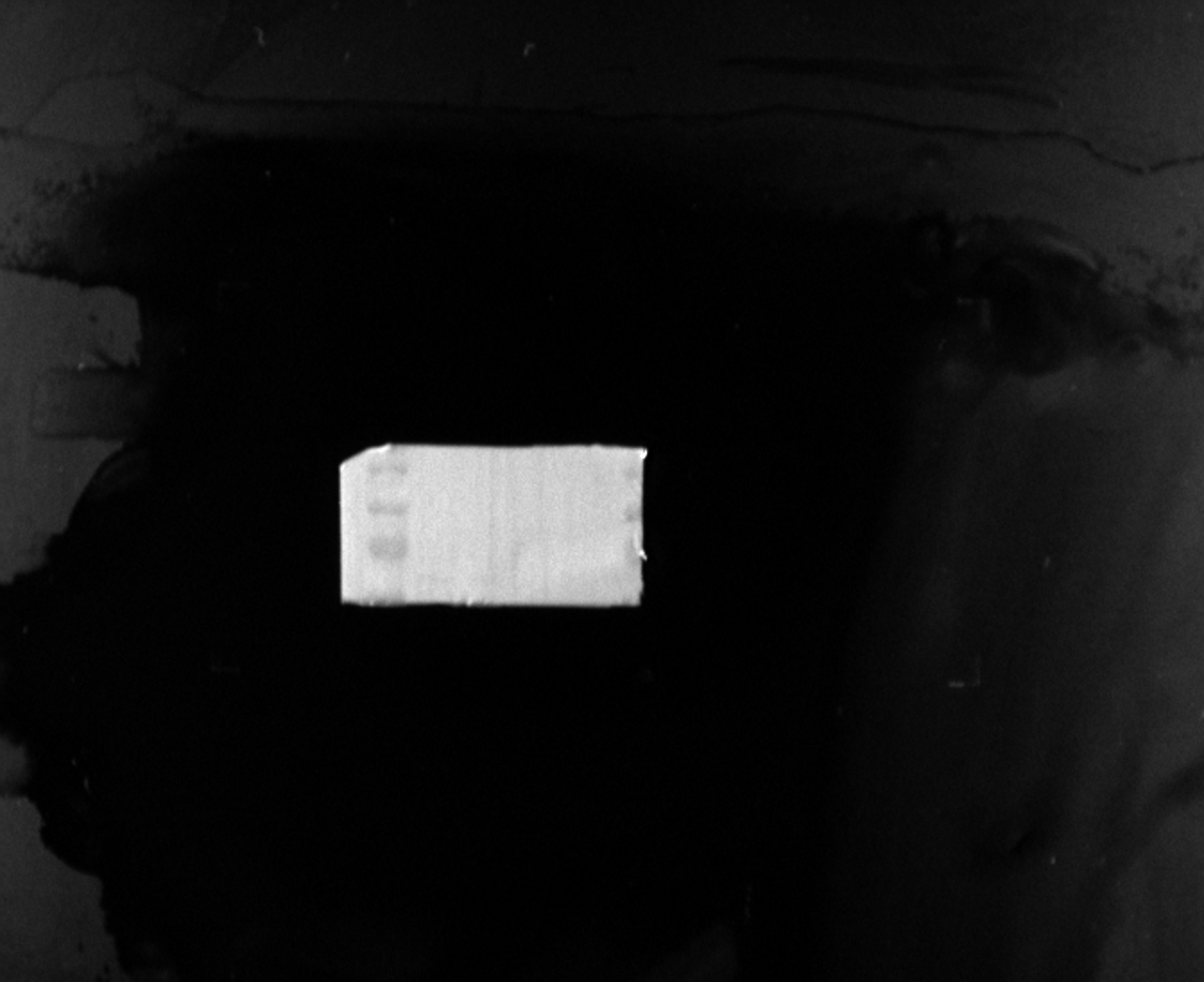

Supplement: Figure 4—source data 2. [file elife-104060-fig4-data2.zip › Figure 4-source data 2/p-AKT/p-akt 2 white.Tif]

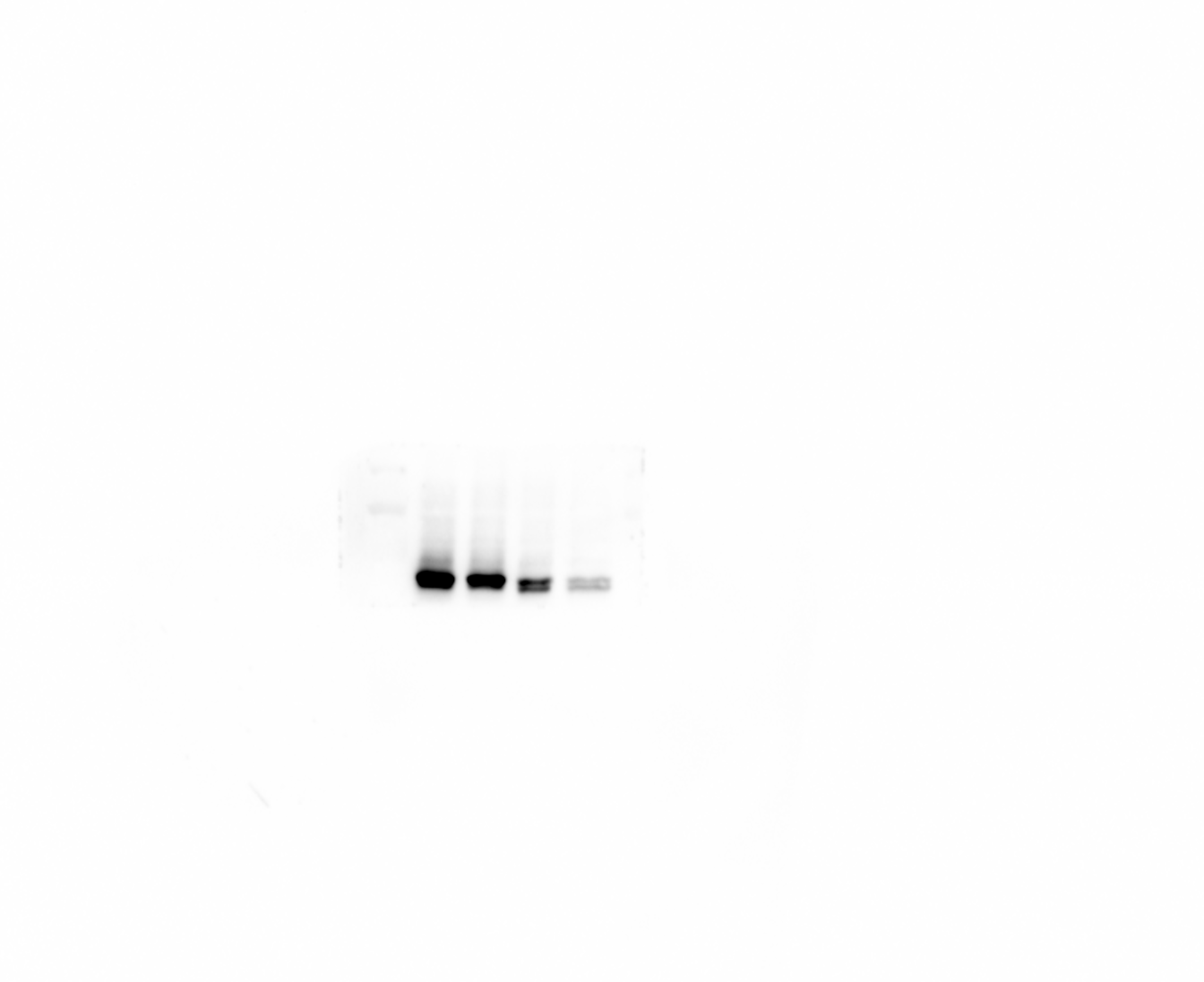

Supplement: Figure 4—source data 2. [file elife-104060-fig4-data2.zip › Figure 4-source data 2/p-AKT/p-akt 2.Tif]

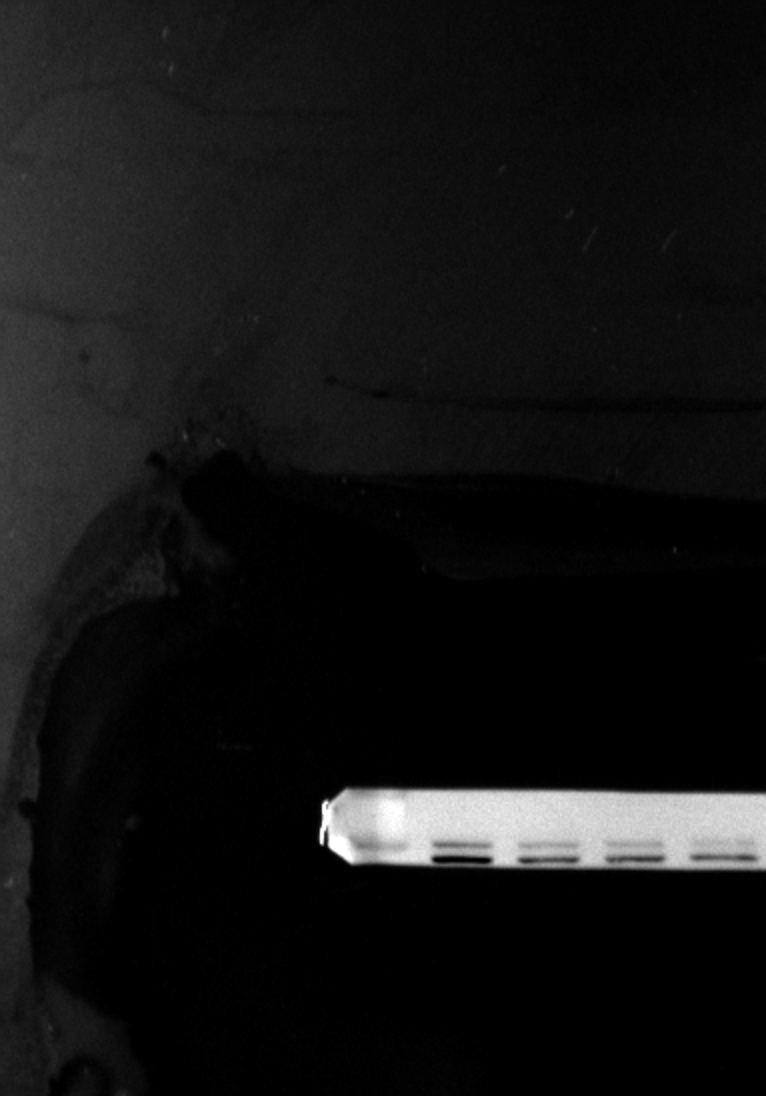

Supplement: Figure 4—source data 2. [file elife-104060-fig4-data2.zip › Figure 4-source data 2/p-ERK/p-erk merge.Tif]

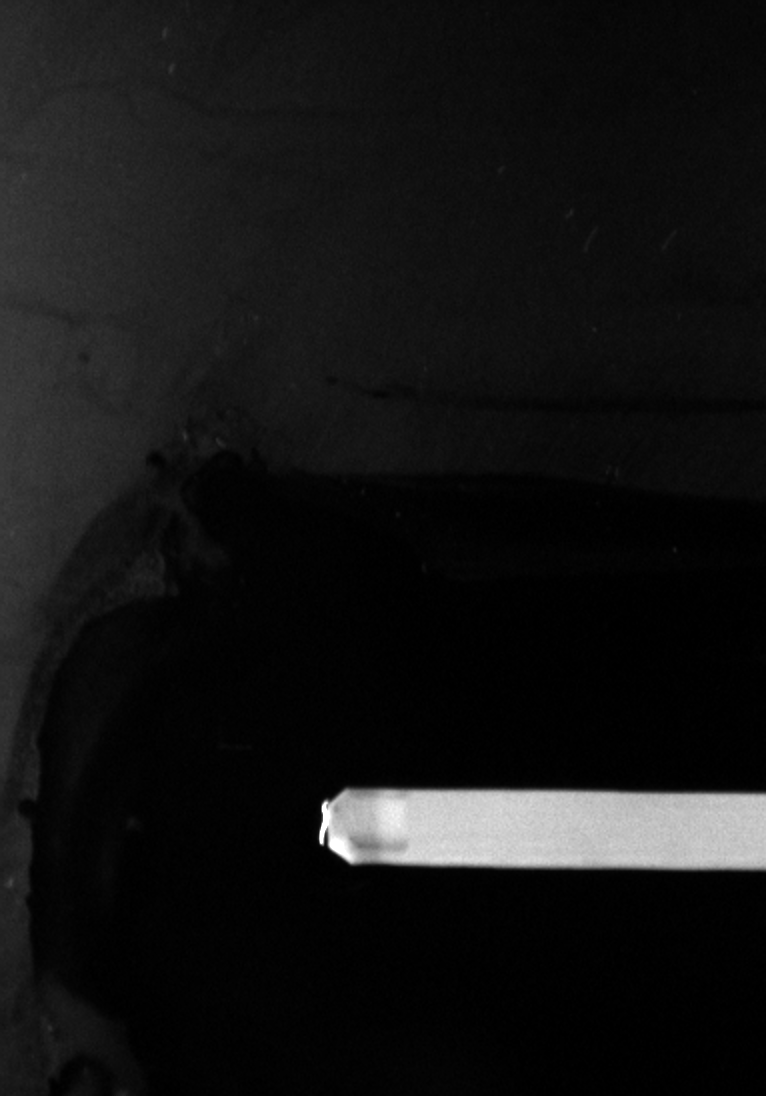

Supplement: Figure 4—source data 2. [file elife-104060-fig4-data2.zip › Figure 4-source data 2/p-ERK/p-erk white.Tif]

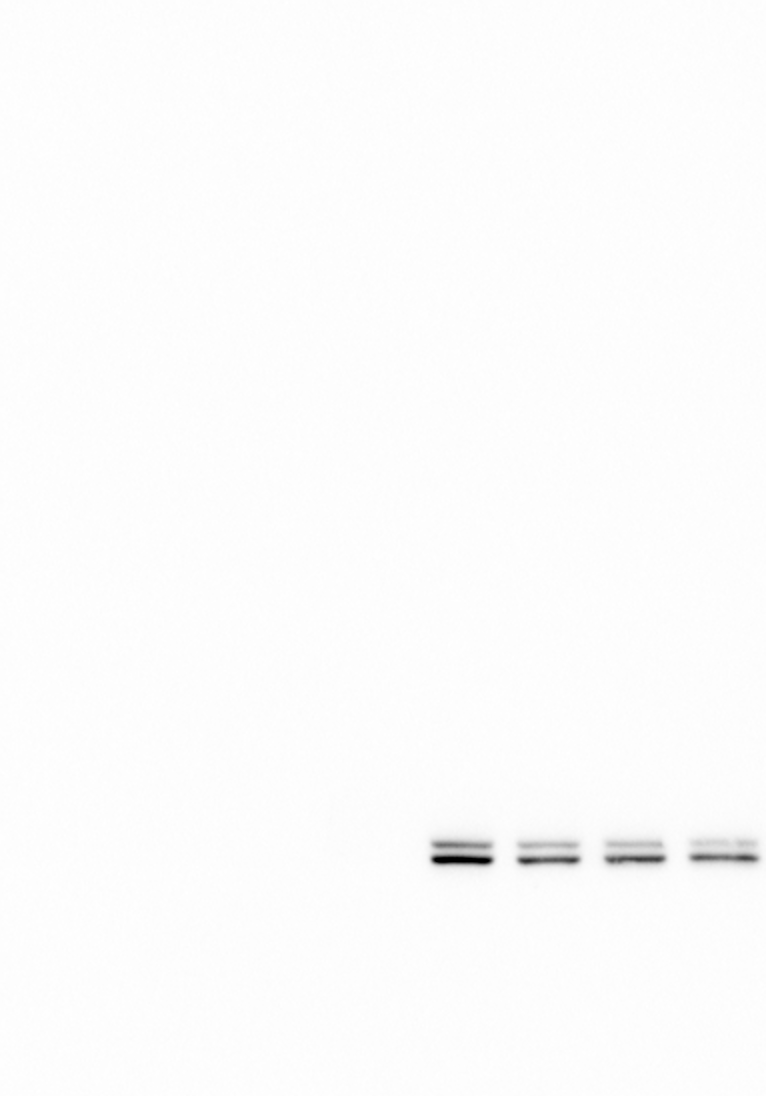

Supplement: Figure 4—source data 2. [file elife-104060-fig4-data2.zip › Figure 4-source data 2/p-ERK/p-erk.Tif]

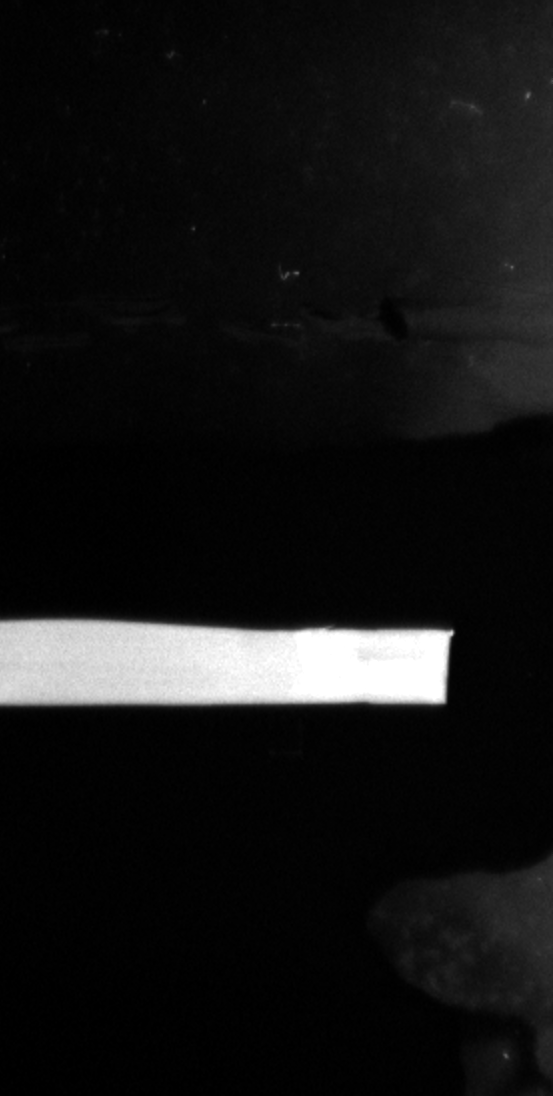

Supplement: Figure 4—source data 2. [file elife-104060-fig4-data2.zip › Figure 4-source data 2/p-FGFR/p-fgfr white.Tif]

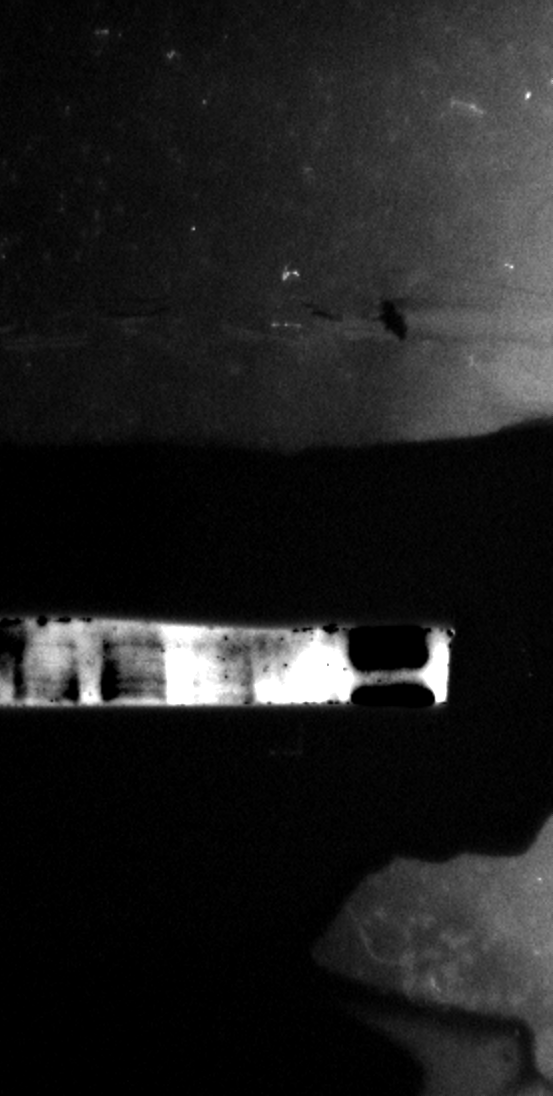

Supplement: Figure 4—source data 2. [file elife-104060-fig4-data2.zip › Figure 4-source data 2/p-FGFR/p-fgfr2 merge.Tif]

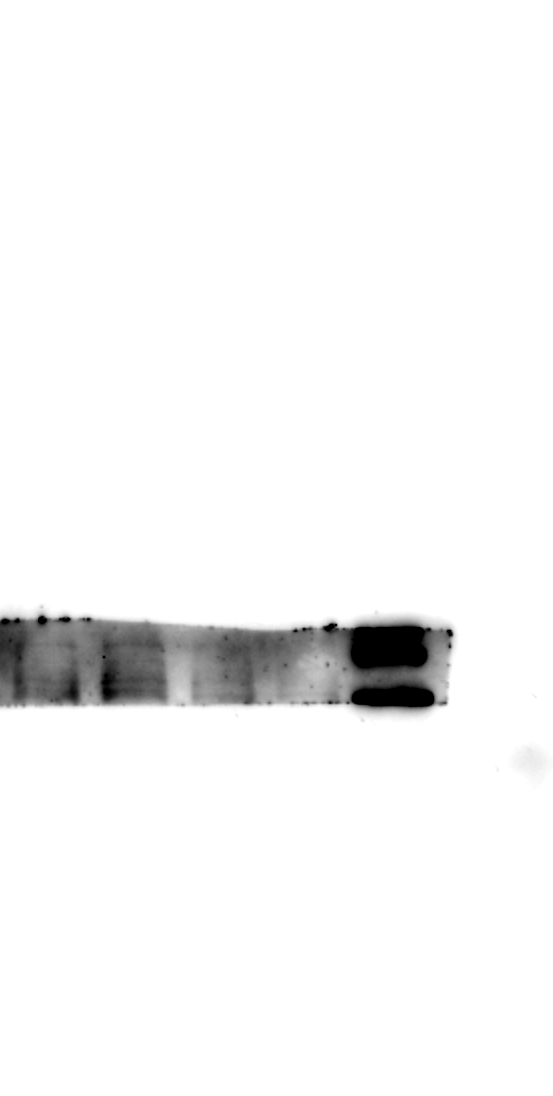

Supplement: Figure 4—source data 2. [file elife-104060-fig4-data2.zip › Figure 4-source data 2/p-FGFR/p-fgfr2(2).Tif]

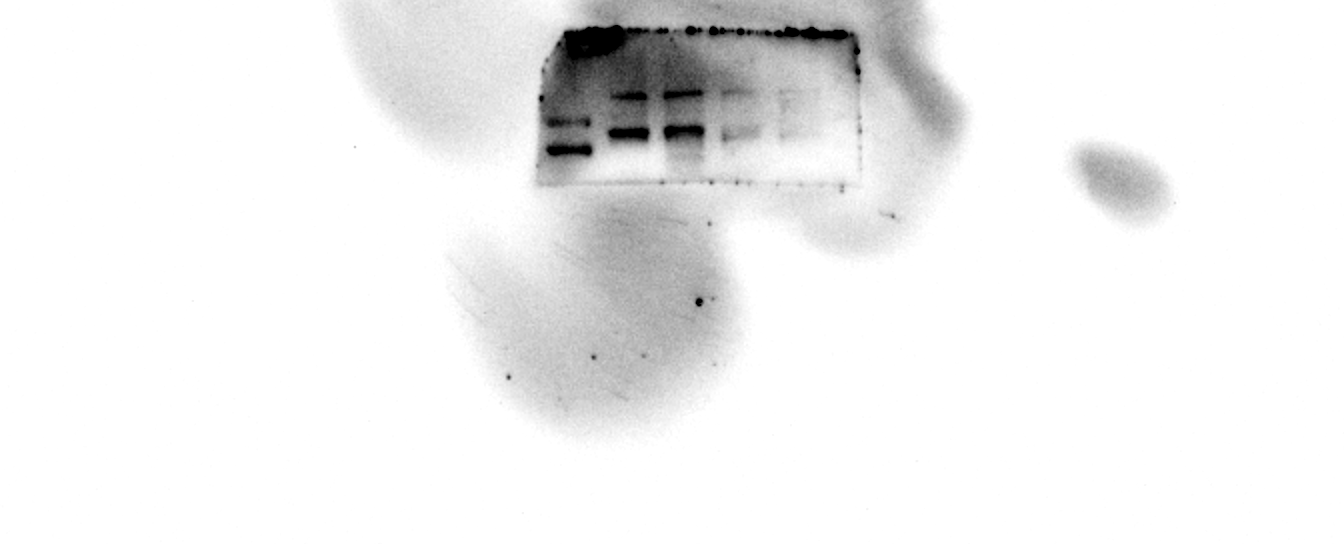

Supplement: Figure 4—source data 2. [file elife-104060-fig4-data2.zip › Figure 4-source data 2/p-mTOR/p-mtor 2.Tif]

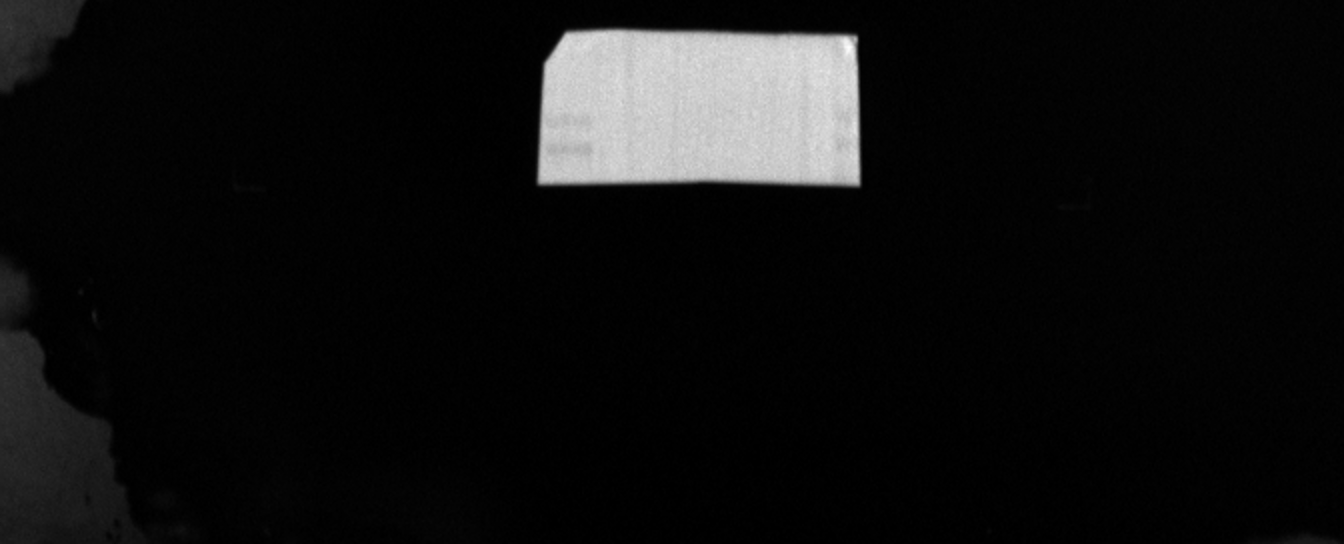

Supplement: Figure 4—source data 2. [file elife-104060-fig4-data2.zip › Figure 4-source data 2/p-mTOR/p-mtor white 2.Tif]

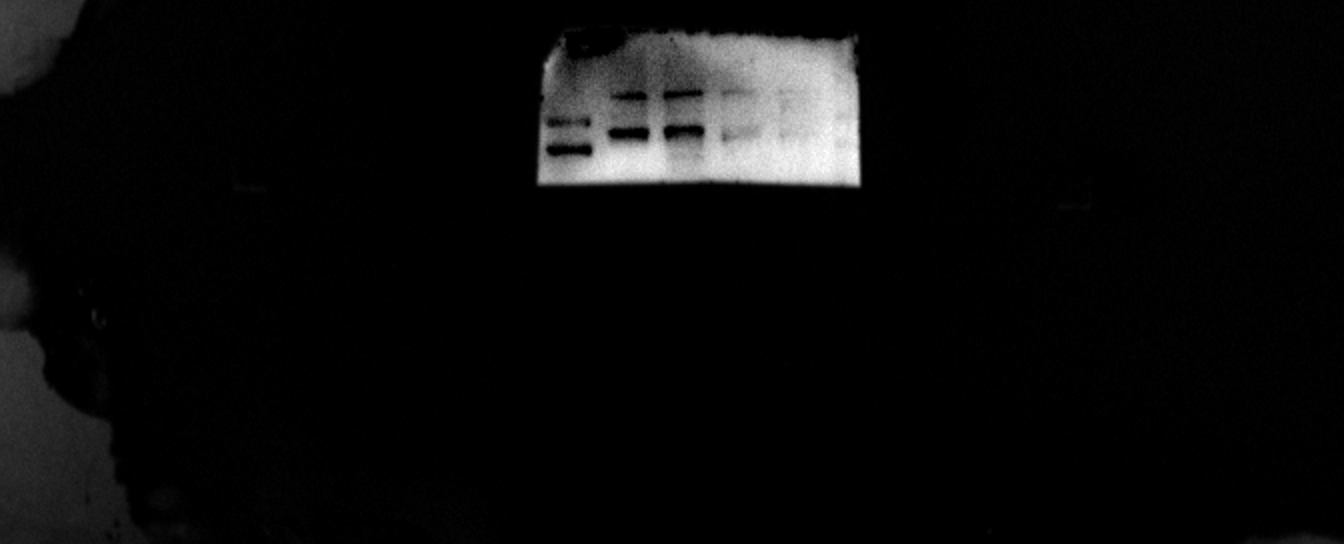

Supplement: Figure 4—source data 2. [file elife-104060-fig4-data2.zip › Figure 4-source data 2/p-mTOR/pmtor merge 2.Tif]

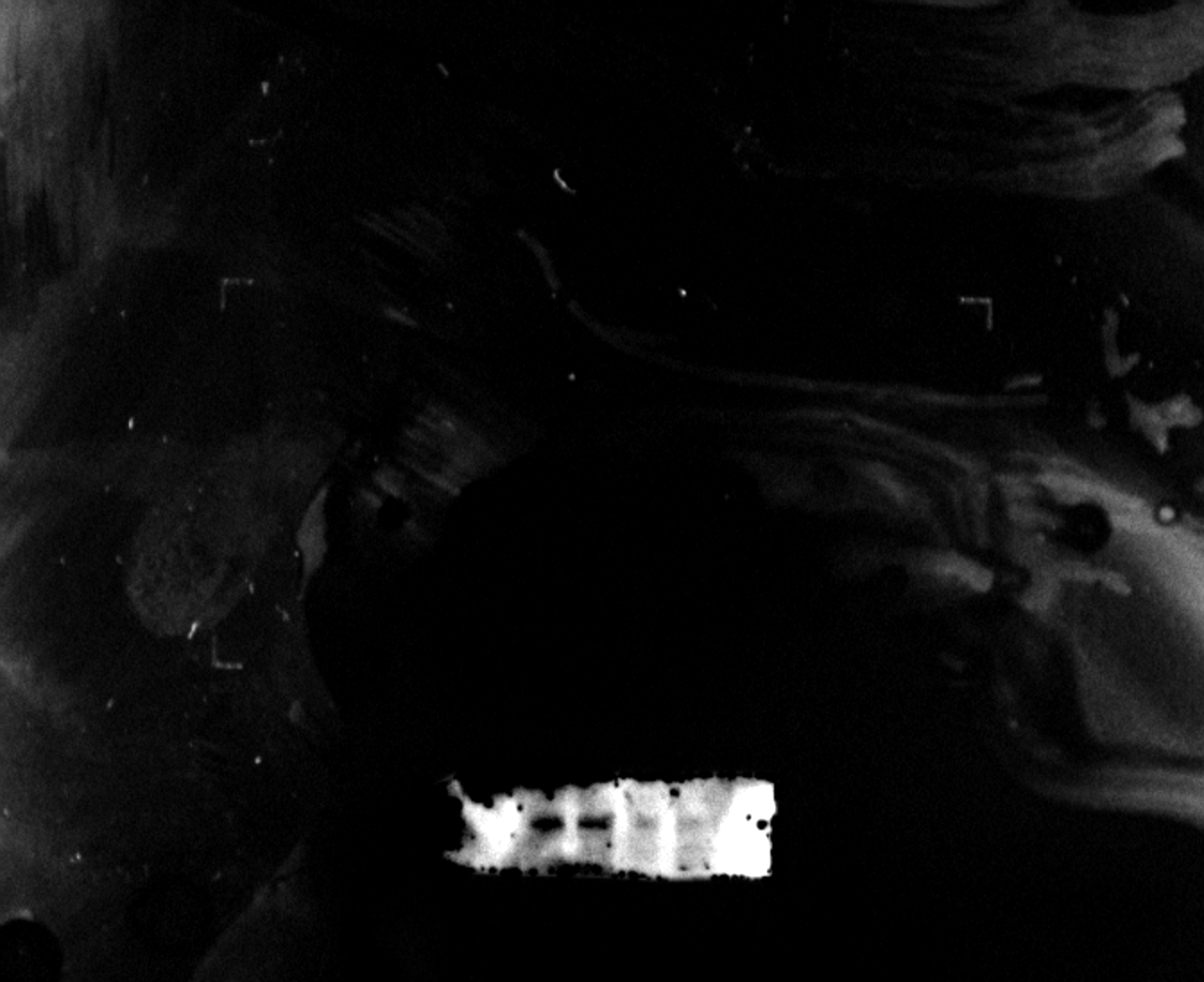

Supplement: Figure 4—source data 2. [file elife-104060-fig4-data2.zip › Figure 4-source data 2/p-SHP2/p-shp2 merge.Tif]

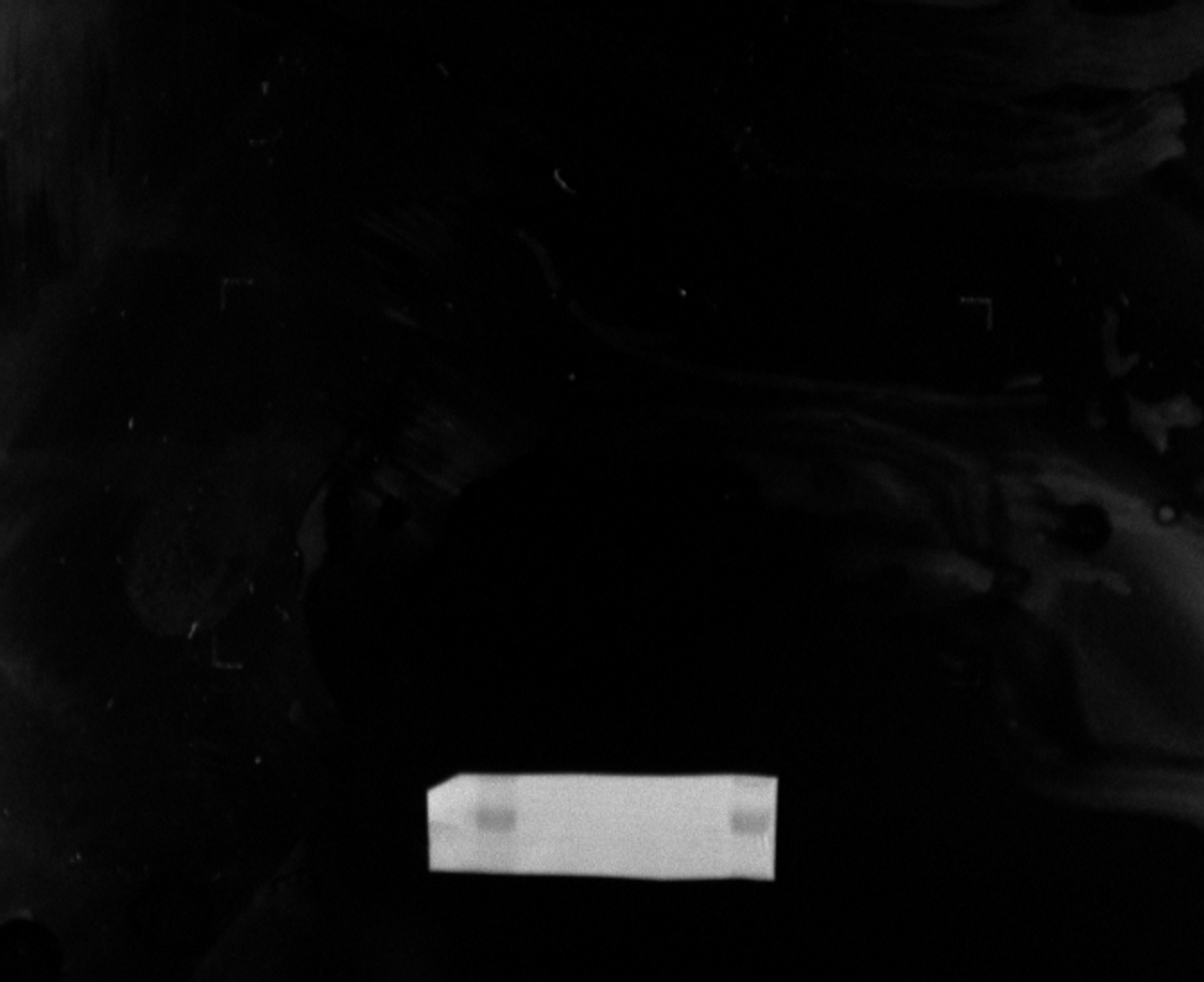

Supplement: Figure 4—source data 2. [file elife-104060-fig4-data2.zip › Figure 4-source data 2/p-SHP2/p-shp2 white.Tif]

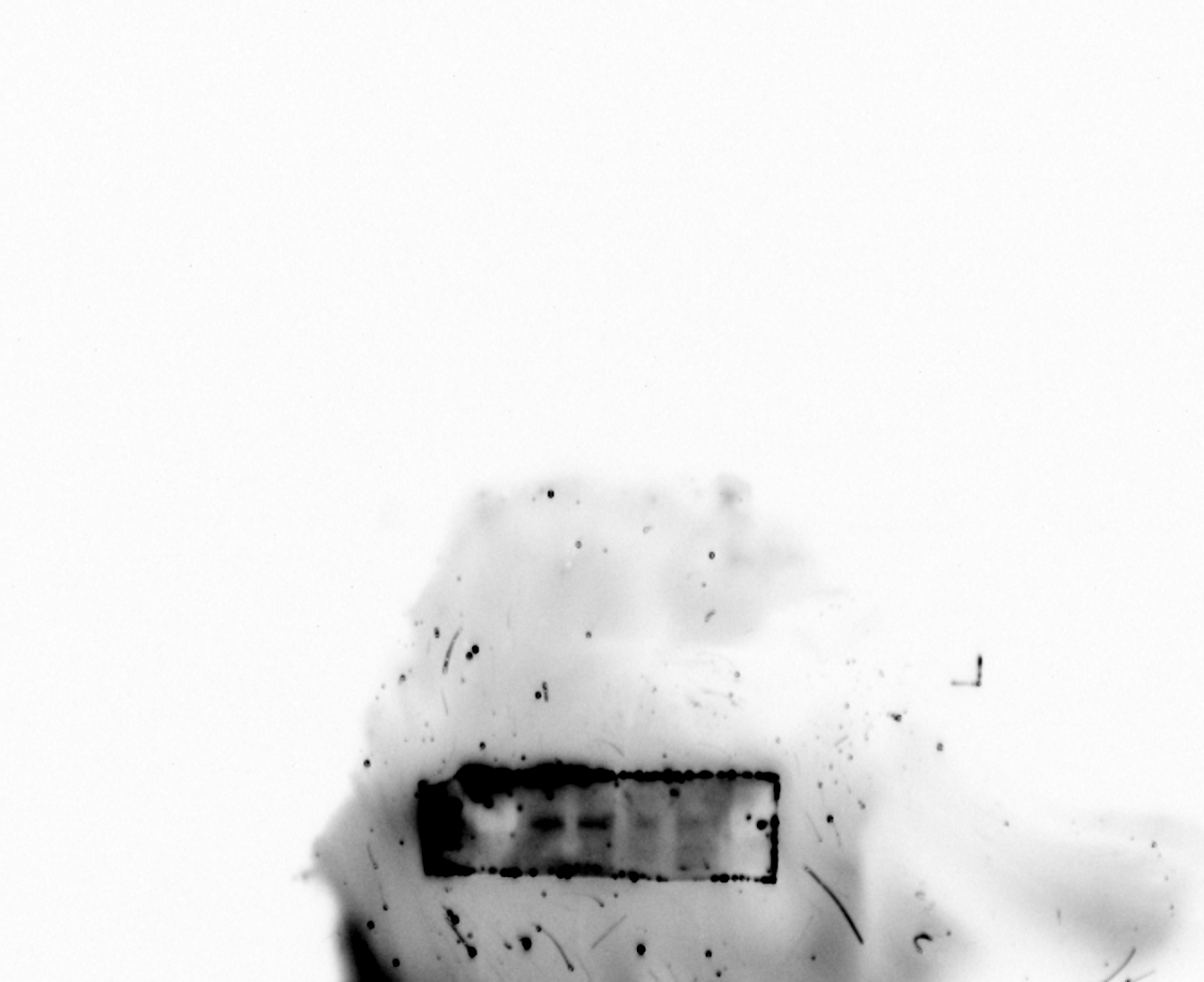

Supplement: Figure 4—source data 2. [file elife-104060-fig4-data2.zip › Figure 4-source data 2/p-SHP2/p-shp2.Tif]

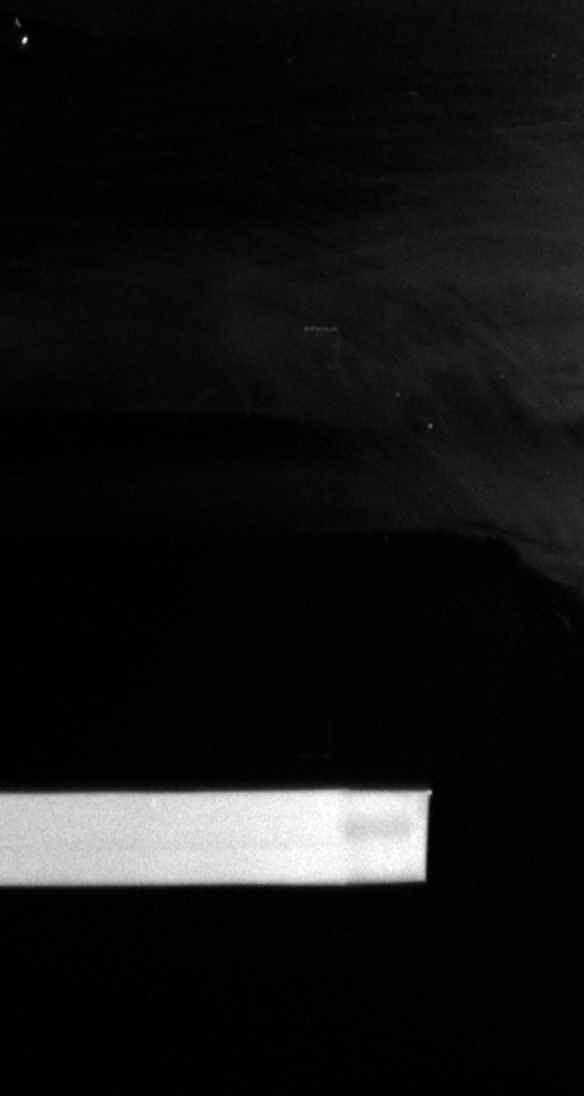

Supplement: Figure 4—source data 2. [file elife-104060-fig4-data2.zip › Figure 4-source data 2/SHP2/p-shp2 white.Tif]

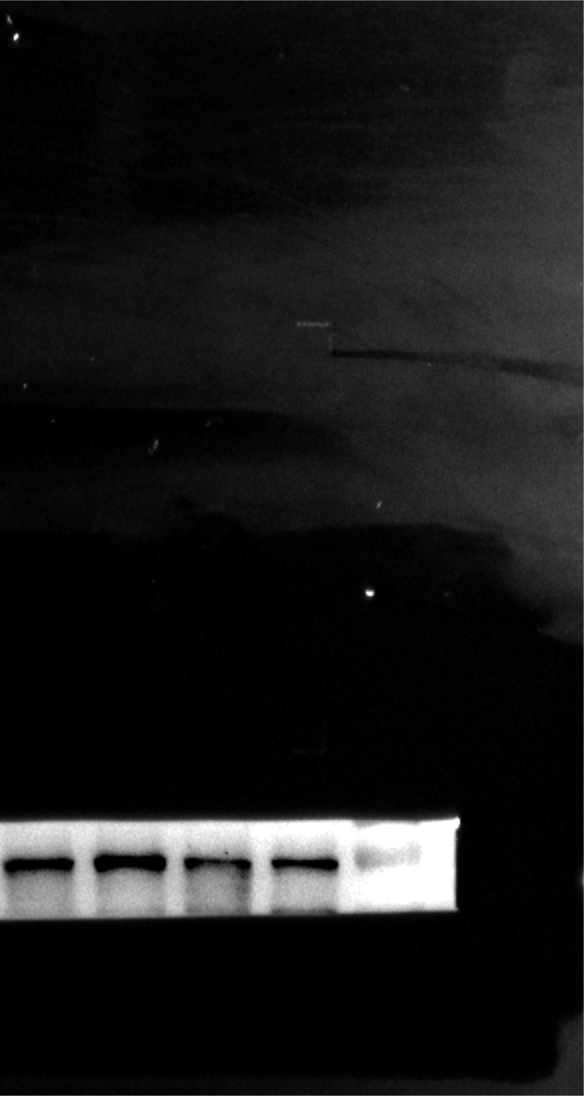

Supplement: Figure 4—source data 2. [file elife-104060-fig4-data2.zip › Figure 4-source data 2/SHP2/shp2 merge=.png]

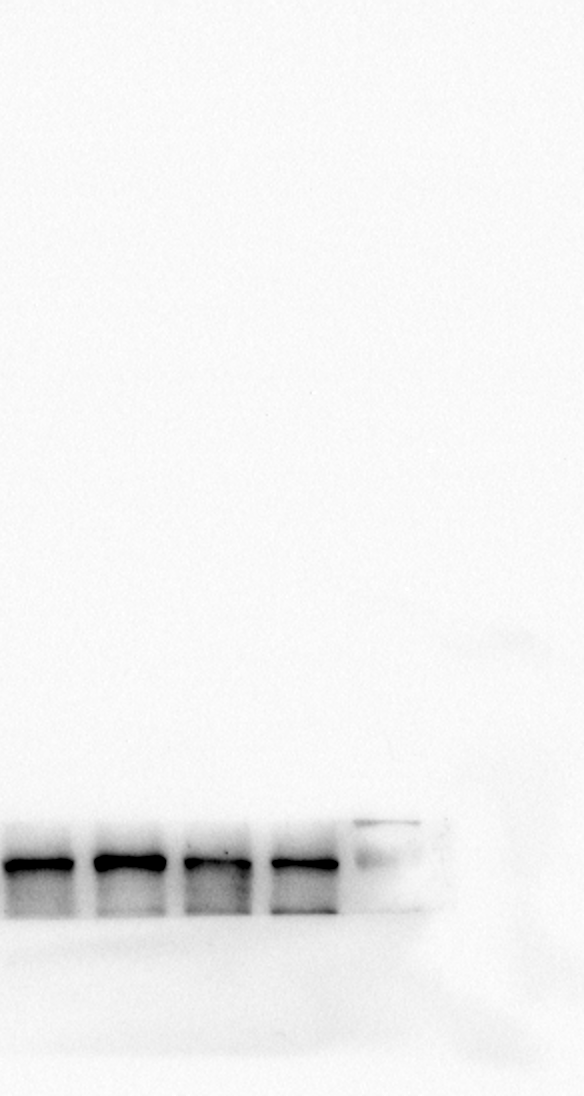

Supplement: Figure 4—source data 2. [file elife-104060-fig4-data2.zip › Figure 4-source data 2/SHP2/shp2=.Tif]
