# Supplementary material for: Blocking SHP2 benefits FGFR2 inhibitor and overcomes its resistance in FGFR2-amplified gastric cancer
Source: eLife. 2026 Mar 23;14:RP104060. doi: 10.7554/eLife.104060 (PMC13008354; doi:10.7554/eLife.104060)
Supplement: Supplementary file 1. [file elife-104060-supp1.pptx]

## Slide 1
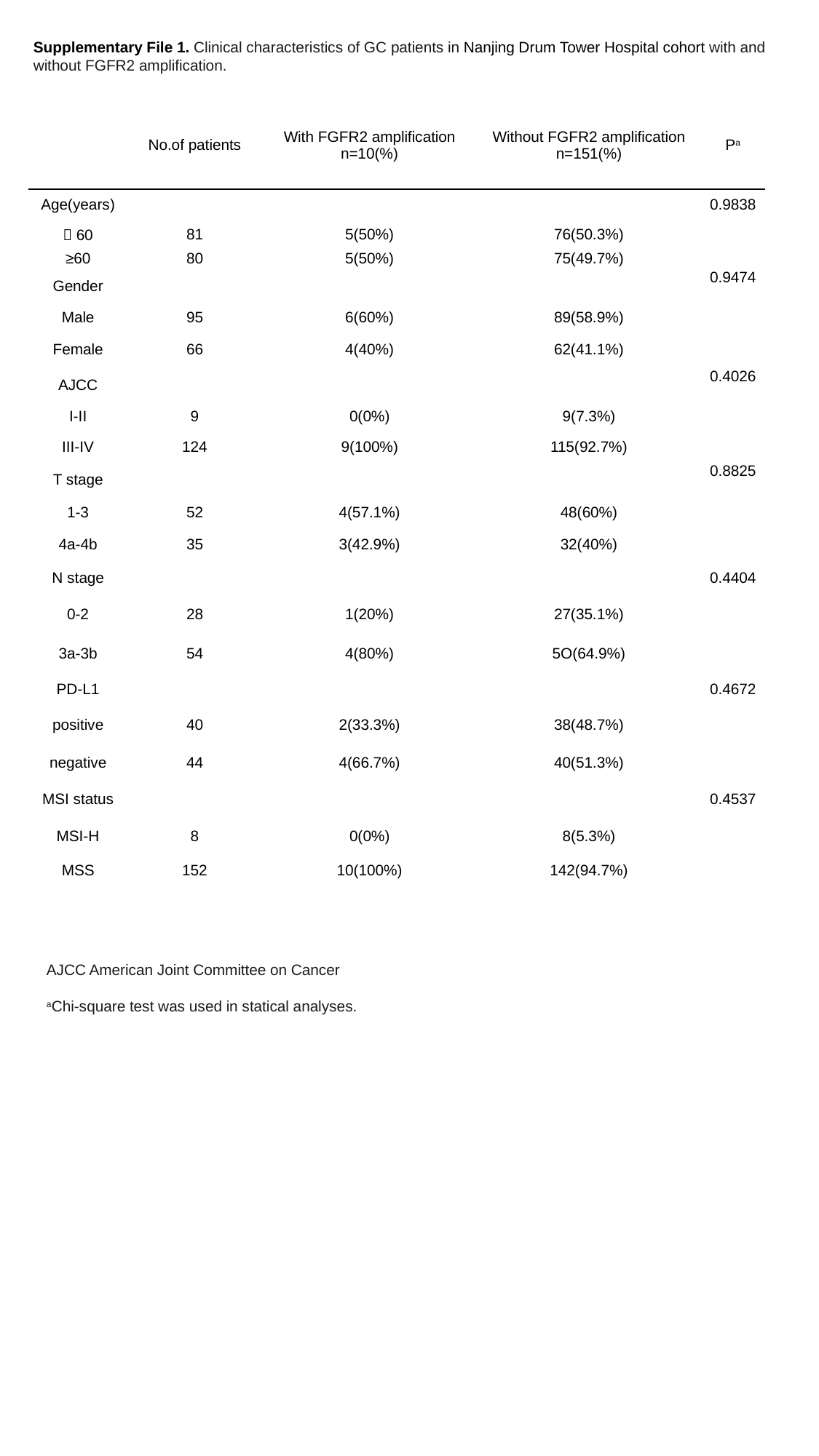

Supplementary File 1. Clinical characteristics of GC patients in Nanjing Drum Tower Hospital cohort with and without FGFR2 amplification.
| | No.of patients | With FGFR2 amplification n=10(%) | Without FGFR2 amplification n=151(%) | Pa |
| --- | --- | --- | --- | --- |
| Age(years) | | | | 0.9838 |
| ＜60 | 81 | 5(50%) | 76(50.3%) | |
| ≥60 | 80 | 5(50%) | 75(49.7%) | |
| Gender | | | | 0.9474 |
| Male | 95 | 6(60%) | 89(58.9%) | |
| Female | 66 | 4(40%) | 62(41.1%) | |
| AJCC | | | | 0.4026 |
| I-II | 9 | 0(0%) | 9(7.3%) | |
| III-IV | 124 | 9(100%) | 115(92.7%) | |
| T stage | | | | 0.8825 |
| 1-3 | 52 | 4(57.1%) | 48(60%) | |
| 4a-4b | 35 | 3(42.9%) | 32(40%) | |
| N stage | | | | 0.4404 |
| 0-2 | 28 | 1(20%) | 27(35.1%) | |
| 3a-3b | 54 | 4(80%) | 5O(64.9%) | |
| PD-L1 | | | | 0.4672 |
| positive | 40 | 2(33.3%) | 38(48.7%) | |
| negative | 44 | 4(66.7%) | 40(51.3%) | |
| MSI status | | | | 0.4537 |
| MSI-H | 8 | 0(0%) | 8(5.3%) | |
| MSS | 152 | 10(100%) | 142(94.7%) | |
AJCC American Joint Committee on Cancer
aChi-square test was used in statical analyses.
